# Living growth of ultra-bright 2D perovskites with long-lived carriers

**DOI:** 10.1038/s41467-026-75434-6

**Published:** 2026-07-14

**Authors:** Yanxin Han, Yahui Li, Qingyang Wei, Hongzhi Zhou, Hongzhi Shen, Wenbo Ma, Zhongpu Wang, Ming Xia, Yanan Wang, Lijun Chai, Xin Sheng, Yiling Zhang, Xiaohe Miao, Yunfan Guo, Zexin Jin, Yang Michael Yang, Juan Du, Long Yuan, Enzheng Shi

**Affiliations:** 1 School of Materials Science & Engineering, Zhejiang University, Hangzhou, China; 2 Department of Materials Science & Engineering, School of Engineering, Westlake University, Hangzhou, China; 3 School of Chemistry and Materials Science, University of Science and Technology of China, Hefei, China; 4 School of Physics and Optoelectronic Engineering, Hangzhou Institute for Advanced Study, University of Chinese Academy of Sciences, Hangzhou, China; 5 State Key Laboratory of Extreme Photonics and Instrumentation, College of Optical Science and Engineering, Zhejiang University, Hangzhou, China; 6 Department of Chemistry, Zhejiang University, Hangzhou, China; 7 Zhejiang Key Laboratory of Precise Synthesis of Functional Molecules, Instrumentation and Service Center for Molecular Sciences and Research Center for Industries of the Future, Westlake University, Hangzhou, China; 8 Jiaxing Key Laboratory of Photonic Sensing & Intelligent Imaging, Intelligent Optics & Photonics Research Center, Jiaxing Research Institute of Zhejiang University, Jiaxing, China; 9 State Key Laboratory of Precision and Intelligent Chemistry, School of Chemistry and Materials Science, University of Science and Technology of China, Hefei, China; 10 Division of Solar Energy Conversion and Catalysis at Westlake University, Zhejiang Baima Lake Laboratory Co., Ltd., Hangzhou, China; 11 Zhejiang Provincial Key Laboratory of Intelligent Low-Carbon Biosynthesis, Westlake University, Hangzhou, China

**Keywords:** Semiconductor lasers, Optical materials

## Abstract

Growth-induced defects and strain in two-dimensional (2D) perovskites severely limit carrier transport and suppress radiative efficiency, thereby sacrificing the carrier lifetime and obscuring the advantages from quantum confinement. Here, we report a near-equilibrium isothermal (NEIT) growth paradigm of 2D perovskite single crystals that exhibits living growth characteristics analogous to living polymerization. This approach confines crystallization primarily to initial nuclei, resulting in ultra-low nucleation density and yielding centimeter-scale single crystals of PEA_2_PbI_4_ (Pb-n1), PEA_2_MAPb_2_I_7_ (Pb-n2), and PEA_2_MA_2_Pb_3_I_10_ (Pb-n3) with enhanced crystallographic perfection. This process achieves ~89% utilization of Pb precursor for Pb-n1, significantly surpassing ~13% from conventional methods and aligning with green-chemistry and sustainable synthetic principles. Additionally, the trap density gets suppressed by one order of magnitude. This unlocks high photoluminescence quantum yields (PLQYs, 77 ± 2% for Pb-n1), first cavity-free lasing in 2D Pb-n1 perovskite flakes, and long carrier lifetimes with diffusion lengths up to ~1.92 μm rivaling 3D perovskites. Critically, the living NEIT growth successfully enables the epitaxy of 2D bulk perovskite heterostructures, driving 60-fold accelerated photocarrier separation in lateral photodetectors. This near-equilibrium living growth paradigm establishes defect-minimized 2D perovskites as a versatile and active platform for high-performance quantum-well optoelectronics.

## Abstract (web summary)

Growth defects and strain in two-dimensional perovskites limit transport and light emission. Han et al. develop a near- equilibrium growth method that yields large, low defect crystals with improved photoluminescence quantum yields, longer lifetimes, and enhanced device performance.

## Introduction

Dimensionality engineering has created new opportunities for tailoring optoelectronic properties in metal halide perovskites. Confining charge carriers into zero-, one-, and two-dimensional (0D/1D/2D) architectures enables strong quantum confinement effects^1–5^, and suppressed ion migration^6–9^. This has enabled near-unity photoluminescence quantum yields (PLQYs > 80%) in 0D quantum dots and 1D nanowires^10–13^, as well as efficient carrier funneling in quasi-2D perovskite films^14^, hereby establishing them as ideal materials for bright light-emitting devices^14–16^. In addition, pure-phase 2D Ruddlesden-Popper perovskites (L_2_A_n-1_M_n_X_3n+1_, A: monovalent cation; B: divalent metal cation; X: halide anion; L: alkyl chain or aromatic ligand; n: number of metal-halide octahedral layer in each layer of 2D perovskite, also denoted as quantum-well thickness) also hold exceptional promise for stable, bandgap-tunable optoelectronics due to their highly ordered ligand passivation and engineered quantum-well structures^3,4,6,17–23^. However, the remarkable potential of pure-phase 2D perovskites has yet to be fully exploited. They consistently exhibit lower PLQYs ( < 80%) than their 0D/1D counterparts and severely limited carrier diffusion lengths ( < 1 μm) compared to 3D analogues^24–26^, directly limiting their application in high-efficiency solar cells and efficient LEDs. More importantly, high PLQYs and long carrier diffusion lengths are rarely achieved simultaneously. This performance deficit originates from growth-induced microstructural imperfections inherent to conventional syntheses (*e.g*. temperature-variant methods, antisolvent precipitation, vapor-assisted crystallization), which introduce thermodynamic and kinetic disturbances. Thermal gradients cause lattice strain and defects, while fluid turbulence promotes high-density homogeneous nucleation and disrupts long-range layer stacking^20,23,27,28^.

Therefore, achieving high-fidelity 2D perovskites demands a fundamental paradigm shift toward crystallization processes that offer unparalleled control over nucleation and growth kinetics. Inspired by living polymerization where chain growth proceeds selectively at active ends without termination or spontaneous initiation^29,30^, in this work, we conceptualize a living crystallization framework of 2D halide perovskite crystals. Our near-equilibrium isothermal (NEIT) strategy maintains crystal growth under strictly controlled conditions, confining propagation to active sites while suppressing parasitic nucleation, minimizing structural disorder and leading to ultra-low nucleation density^31^. By maintaining a steady temperature environment under strictly isothermal conditions, the NEIT growth circumvents the thermally induced lattice strain and defect proliferation inherent to conventional solvent-mediated and temperature-variant methods, eliminating extrinsic perturbations that compromise crystallographic coherence. This breakthrough enables the synthesis of centimeter-scale 2D Ruddlesden-Popper perovskite single crystals (Pb-n1, Pb-n2, and Pb-n3) with improved crystallographic perfection, an order-of-magnitude suppression of trap densities (1.27 × 10^9 ^cm^−3^ *vs*. 1.49 × 10^10^ cm^−3^), and record-narrow XRD rocking curves. In addition, this represents a giant leap in terms of green-chemistry and sustainable synthesis, achieving ~89% utilization of Pb precursor for Pb-n1, significantly surpassing ~13% from conventional method (Supplementary Methods and Supplementary Tables 1-2). Consequently, the NEIT growth unlocks unprecedented optoelectronic benchmarks. Firstly, we achieved a high PLQY of 77 ± 2% for Pb-n1 crystals, alongside the first demonstration of cavity-free lasing in exfoliated 2D n1 lead halide perovskite monolayers. Simultaneously, the carrier lifetime exceeds 200 ns and the carrier diffusion length reaches 1.92 μm (Pb-n3), approaching 3D perovskite performance while retaining quantum confinement advantages. Moreover, the living NEIT growth enables the epitaxial fabrication of diverse single-crystal bulk heterostructures with vertical van der Waals (vdW) and lateral epitaxial configurations, all of which exhibit abrupt interfaces. This capability bridges the gap between interfacial precision and structural scalability, enabling heterostructure photodetectors with 60-fold-accelerated response kinetics (492/340 μs rise/decay) compared with single-component devices.

## Results

**Fig. 1:**
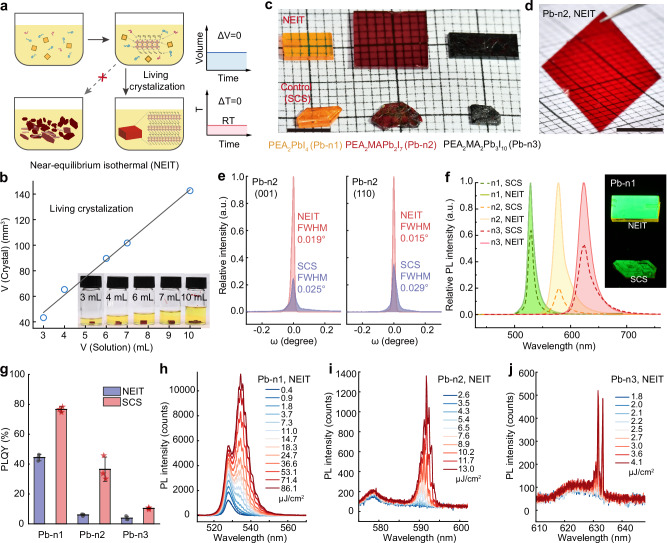
Structural and spectroscopic characterizations of 2D perovskite single crystals synthesized via NEIT and SCS approaches. **a** Schematic diagram of living NEIT growth process. The right two plots indicate no temperature gradient and no solvent exchange during the growth. **b** Linear relationship between as-synthesized crystal volume and the added precursor solution volume. Insets show optical images of as-synthesized Pb-n2 single crystals in 3 mL, 4 mL, 6 mL, 7 mL and 10 mL of precursor solutions. **c,** **d** Optical images of Pb-n1, Pb-n2, and Pb-n3 bulk single crystals synthesized from NEIT and SCS approaches. Scale bar: 5 mm. **e** Rocking curves of the (001) and (110) diffraction peaks of Pb-n2 synthesized from NEIT and SCS approaches. **f,** **g** PL spectra (**f**) and PLQYs (**g**) of Pb-n1, n2 and n3 single crystals synthesized from NEIT and SCS approaches. The inset in f shows fluorescence images of Pb-n1 single crystals synthesized from NEIT and SCS approaches under UV flashlight excitation. For PLQYs, error bars are presented as standard deviation (SD). **h–j** Power-dependent lasing spectra at 80 K for exfoliated flakes synthesized from NEIT growth: Pb-n1 (pump fluence from 0.4 to 86.1 μJ/cm^2^), Pb-n2 (2.6 to 13.0 μJ/cm^2^) and Pb-n3 (1.8 to 4.1 μJ/cm^2^).

Conventional perovskite crystal growth methods, such as temperature-variant protocols (*e.g*. slow-cooling synthesis (SCS) utilizing aqueous HI/H_3_PO_2_ mixtures and inverse temperature crystallization)^32^, and solvent-mediated approaches (*e.g*. solvent evaporation or antisolvent crystallization methods)^28,33,34^, inevitably introduce extrinsic thermodynamic perturbations. Specifically, the temperature gradient in temperature-variant growth tends to induce simultaneous energy fluctuation to increase nucleation probability and thermal strain to increase defect density. In the SCS (HI/H_3_PO_2_) system, dynamic light scattering (DLS) spectra reveal that minor cooling triggers the rapid emergence of aggregates. This is likely due to the rapid hydrophobic aggregation of bulky PEA^+^ cations. Consequently, abundant heterogeneous nucleation sites are generated and the metastable zone is narrowed, driving uncontrolled multi-nucleation^35^ (Supplementary Figs. 1–3). Meanwhile, dynamic solvent exchange in solvent-mediated techniques triggers uncontrolled supersaturation spikes, driving stochastic nucleation and defect proliferation. In stark contrast, our NEIT strategy operates within a rationally selected MeOH environment. Strong Lewis base solvents (e.g., DMF, DMSO) are excluded as their stable coordination with Pb^2+^ suppresses the formation of critical precursor clusters^36–38^. Among weakly coordinating alcohols, the decreasing dielectric constant from MeOH to EtOH and IPA progressively restricts the dissolution of PbI_2_ precursors^37^ (Supplementary Fig. 4). The NEIT strategy operates under strictly isothermal conditions (room temperature, RT) within a sealed, mono-solvent (MeOH) environment, establishing a steady supersaturation profile that enables diffusion-limited growth kinetics. This meticulously controlled, turbulence-free growth paradigm effectively eliminates convective disturbances and thermal transients, thereby enabling near-equilibrium crystallization process. DLS tracking confirms that the precursor maintains a stable molecular state (1-2 nm) for over 22 h at RT (Supplementary Figs. 5–8). This stability is likely because MeOH keeps the organic PEA^+^ cations extended, which minimizes heterogeneous nucleation sites and sustains a broad metastable zone. Once a primary nucleus forms, precursor ions preferentially add to this active center due to a lower energetic barrier, effectively inhibiting secondary nucleation to yield a single crystal, demonstrating strong living characteristics (Fig. 1a)^39^. To further illustrate these living features, we investigated the correlation between the as-grown single crystal and the growth solution at a fixed concentration. The crystal volume scales linearly with the added solution volume, while the solution remains free of parasitic nucleation, typically yielding a single dominant crystal. This result quantitatively confirms that growth is governed by active units, with the crystal volume precisely determined by the total precursor mass, a hallmark of a living and controlled process (Fig. 1b and Supplementary Table 3). Moreover, adjusting the initial precursor concentration correspondingly modulates the final crystal size, offering two viable strategies for dimensional customization (Fig. 1b and Supplementary Fig. 9). We further demonstrated the persistent reactivity of crystal surfaces and edges by reintroducing two distinct Pb-n2 single crystals, including one well-defined cuboid and another with a deliberately removed corner (Supplementary Fig. 10), into fresh NEIT growth solution. Both crystals grew into larger, well-defined cuboids along all three dimensions. The restoration of the damaged corner reflects kinetic Wulff-shaped growth driven by facet-dependent surface energies^40^. More importantly, the sustained growth from all exposed facets, including irregular regions, indicates that crystal terminations remain active and capable of epitaxial extension upon nutrient supply—a definitive characteristic of a living system.

Consequently, NEIT growth produces centimeter-scale single crystals of Pb-n1, Pb-n2, and Pb-n3 with geometrically defined facets and exceptional optical clarity, visual hallmarks of minimized defect densities and structural homogeneity (Fig. 1c, d and Supplementary Fig. 11). Quantitative transmittance spectroscopy confirms the superior macroscopic quality of the NEIT-grown crystals. The NEIT-grown Pb-n2 crystal maintains consistent transmittance of ~85% with good spatial uniformity (thickness of 619 μm), compared to highly variable transmittance (47%–79%) for the SCS-grown counterpart (thickness of 68 μm) (Supplementary Fig. 12). Comprehensive structural validation confirms crystallographic fidelity: single-crystal XRD identifies identical space groups (P1 for Pb-n1, n2 and n3) compared to SCS references (Supplementary Tables 4–6), while powder XRD (PXRD) patterns exhibit congruent (00k) periodicities (16.3 Å for Pb-n1, 22.6 Å for Pb-n2, 28.5 Å for Pb-n3) free of impurity signatures, unambiguously verifying phase purity and quantum-well periodicity alignment with single-crystal structures (Supplementary Fig. 13).

The crystallographic superiority imparted from NEIT growth is quantitatively resolved through high-resolution XRD (HRXRD) characterizations. Rocking curve analyses of NEIT-grown Pb-n2 single crystals reveal dramatically narrowed (001) and (110) diffraction peaks, exhibiting full width at half maximum (FWHM) values of just 0.019° and 0.015° (Fig. 1e), 24 ~ 48% reduction versus SCS counterparts (0.025° and 0.029°) (Supplementary Fig. 14). This narrowing signifies a profound enhancement in long-range crystalline order, directly attributable to suppressed defect formation during NEIT growth^41^. Aside from structural optimization, the crystallographic superiority manifests in optoelectronic performance. NEIT-grown crystals exhibit much brighter PL emissions compared to SCS grown crystals at identical emission peaks ( ~ 530 nm for Pb-n1, ~580 nm for Pb-n2, ~620 nm for Pb-n3; Fig. 1f), culminating in a high PLQY of 77 ± 2% for NEIT-grown Pb-n1 crystal—surpassing SCS counterpart (44 ± 2%) and establishing a state-of-the-art level for all reported Ruddlesden-Popper perovskite single crystals^20,24,42–44^. Similarly, PLQYs for Pb-n2 and Pb-n3 reach 37 ± 8% and 10 ± 1%, representing 6.3-fold and 2.6-fold enhancement over conventional SCS methods (6 ± 1% and 4 ± 1% for SCS-grown Pb-n2 and Pb-n3 crystals, Fig. 1g and Supplementary Figs. 15, 16). To ensure a fair comparison, these measurements were performed on mechanically exfoliated nanoflakes with similar thicknesses. Furthermore, both polarization-dependent and low-angle (0–62°) angle-dependent PL spectroscopy confirmed the isotropic out-of-plane emission profile of these 2D perovskite flakes. However, high-angle (62-90°) angle-dependent PL revealed that the in-plane emission is weaker than the out-of-plane emission. By combining the PL profiles from both angular ranges, the final PLQY values were recalibrated (Supplementary Figs. 17–25). This substantial increase in PLQY indicates the improved crystal quality substantially suppresses non-radiative recombination in the single crystals, confirming NEIT’s efficacy in passivating deep-level traps associated with halide vacancies and organic spacer disorder^45^.

Based on this defect-minimized and highly bright 2D perovskite single crystals, we demonstrate a previously unattainable milestone: cavity-free optically pumped lasing in exfoliated 2D n1 lead perovskite (PEA_2_PbI_4_) thin flakes. Under optical excitation, NEIT-grown Pb-n1 exhibits sharp lasing at 534 nm (FWHM ~ 0.53 nm) upon exceeding the lasing threshold fluence of 14.7 μJ/cm^2^, spectrally distinct from spontaneous excitonic emission at 528 nm (FWHM ~ 5 nm, Fig. 1h, i). Under the high optical excitation fluences required for lasing, trap states are generally saturated, and Auger recombination dominates the carrier loss^46,47^. Nevertheless, the reduced trap density in NEIT-grown crystals inherently could lower the defect-mediated Auger recombination rate and minimizes non-radiative thermal degradation, effectively facilitating population inversion^48^. We conducted measurements across randomly selected regions with varying flake thicknesses (Supplementary Fig. 26). The NEIT-grown flakes exhibited multimode lasing with threshold of ~15 μJ/cm^2^ (Fig. 1h and Supplementary Fig. 27). In contrast, exfoliated flakes from SCS-grown crystals tested under identical conditions only displayed amplified spontaneous emission (ASE) with a significantly higher threshold ( ~ 100 μJ/cm^2^) (Supplementary Fig. 28). Importantly, systematic quantum-well engineering via increasing n value progressively attenuates carrier-phonon coupling and Auger annihilation rates^46,49^, thereby enabling further reduction in lasing thresholds to 5.4 μJ/cm^2^ (Pb-n2, 591 nm) and 2.1 μJ/cm^2^ (Pb-n3, 632 nm) (Supplementary Fig. 27). Polarization-resolved spectroscopy confirms coherent lasing emission in all systems, conclusively distinguishing it from amplified spontaneous emission (Supplementary Fig. 29). Furthermore, we observed distinct interference fringes and extracted coherence times (τ_c_) of 73.8 fs, 332.2 fs, and 424.8 fs for the Pb-n1, n2, and n3 perovskites, respectively (Supplementary Fig. 30). The temporal coherence signatures, combined with linear polarization above the threshold, confirm the lasing emission. Collectively, these results establish a clear structure-property correlation: NEIT-driven crystallographic perfection directly enables record-high PLQYs and tunable optically pumped lasing across a 90-nm spectral range (Pb-n1, n2 and n3), validating NEIT growth as a universal strategy for synthesizing high-quality quantum-confined semiconductors.

**Fig. 2:**
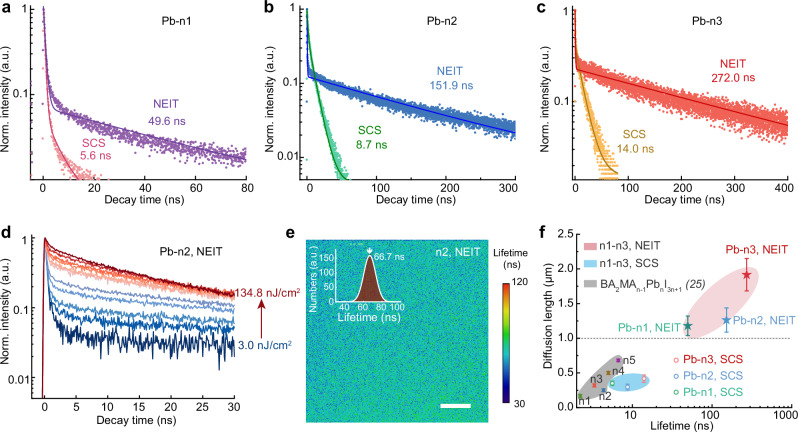
Prolonged carrier lifetime and diffusion length of NEIT-grown 2D halide perovskites. **a**–**c** TRPL spectra of the Pb-n1, n2 and n3 single crystals synthesized from NEIT and SCS approaches, respectively. **d** Power-dependent TRPL spectra of Pb-n2 synthesized from NEIT growth with pump power increasing from 3.0 to 134.8 nJ/cm^2^. **e** FLIM pseudo-color image of Pb-n2 synthesized from NEIT growth. Scale bar, 10 µm. The inset represents the carrier lifetime distribution. **f** Carrier diffusion length versus lifetime of 2D perovskite synthesized from NEIT, SCS approaches and literature reports^25^. Error bars are presented as standard error (SE).

Long-range carrier diffusion is highly desired in halide perovskite solar cells, photo detectors and X-ray detectors^50,51^. The promise of 2D perovskites as next-generation optoelectronic materials has long been constrained by a fundamental trade-off: quantum confinement enhances exciton stability but simultaneously amplifies non-radiative losses through defect-mediated recombination, restricting carrier diffusion lengths to sub-micrometer scales ( < 1 μm)^25^. Our NEIT strategy overcomes this limitation by achieving optimized lattice perfection, which dramatically extends carrier lifetimes through the suppression of atomic defects and disorders that traditionally dominate recombination pathways. Time-resolved photoluminescence (TRPL) analyses of NEIT-grown crystals reveal characteristic biexponential decay kinetics (Fig. 2a–c and Supplementary Figs. 31–33), comprising a fast decay component (τ_1_ ~ 0.3–0.8 ns) attributed to surface trap-assisted recombination where carriers are captured by unsaturated defect states at surface terminations, and a slow decay component (τ_2_) representing intrinsic radiative decay and the bulk recombination lifetime in defect-minimized regions^52^. Crucially, τ_2_ values reach 49.6 ns for Pb-n1, 151.9 ns for Pb-n2, and 272.0 ns for Pb-n3, representing 8.9-fold, 17.5-fold, and 19.4-fold enhancements over SCS controls (5.6 ns, 8.7 ns, and 14.0 ns, respectively). The large magnitude of enhancement underscores NEIT’s efficacy in defect passivation. As evidenced by power-dependent TRPL measurements: increasing pump fluence from 3.0 to 134.8 nJ/cm^2^ for Pb-n2 (and 2.4 to 104.9 nJ/cm^2^ for Pb-n3) progressively diminishes the amplitude of τ_1_ due to trap state filling, while τ_2_ experiences a moderate reduction owing to Auger recombination at elevated carrier densities^53^ (Fig. 2d and Supplementary Fig. 34). This fluence-dependent behavior conclusively establishes τ_1_ as a defect-mediated process and τ_2_ as the fundamental bulk lifetime. Fluorescence lifetime imaging microscopy (FLIM) of exfoliated Pb-n2 flakes synthesized from NEIT growth further confirms exceptional spatial homogeneity, with carrier lifetimes concentrated at 66.7 ns across a 60 × 60 μm^2^ region (Fig. 2e), demonstrating large-area uniformity in NEIT-grown 2D perovskites. This spatial consistency highlights NEIT’s capacity for bulk-like defect suppression. It is worth noting that the shorter lifetime measured by FLIM ( ~ 66.7 ns) compared to TRPL ( ~ 151.9 ns) arises from the different excitation power densities ( ~ 900 nJ/cm^2^ in FLIM versus ~5 nJ/cm^2^ in TRPL).

To resolve the compromise between quantum confinement advantages and carrier transport limitations, we employed TRPL imaging microscopy, a spatio-temporally resolved technique that quantifies diffusion constants (D). As a result, NEIT-grown crystals exhibit D values of 0.281 ± 0.004 cm^2^/s (Pb-n1), 0.105 ± 0.002 cm^2^/s (Pb-n2), and 0.135 ± 0.002 cm^2^/s (Pb-n3) (Supplementary Figs. 35–40), surpassing SCS controls by 4–28% and comparable with prior reports on (BA)_2_MA_n-1_Pb_n_I_3n+1_ homologs^25^. This non-monotonic dependence of D on n value of Pb-n1, n2 and n3 originates from the competing modulation by exciton–phonon coupling and octahedral tilting (in-/out-of-plane)^26^. This intricate interplay between dimensionality and lattice dynamics highlights the challenge of optimizing transport in layered perovskites. Remarkably, the synergy between prolonged τ_2_ and optimized D in our NEIT-grown crystals yields record-long carrier diffusion lengths ($L=\sqrt{D{\tau }_{2}}$): 1.18 ± 0.14 μm for Pb-n1, 1.26 ± 0.17 μm for Pb-n2, and 1.92 ± 0.23 μm for Pb-n3 (Fig. 2f). These values surpass SCS references by 3.4–4.6 times (0.35 ± 0.03 μm, 0.30 ± 0.04 μm, 0.42 ± 0.05 μm) and 5 times of the prior records for (BA)_2_MA_n-1_Pb_n_I_3n+1_ (*n* = 1 ~ 3, <0.35 μm), with the 1.92 μm diffusion length for Pb-n3 approaching benchmarks set by 3D perovskites (L ~ 1–5 μm)^50,54^ (Supplementary Table 7). This breakthrough demonstrates NEIT’s unique capacity to decouple quantum confinement benefits from traditional transport barriers, enabling 2D systems to retain excitonic advantages while achieving bulk-like diffusion.

**Fig. 3:**
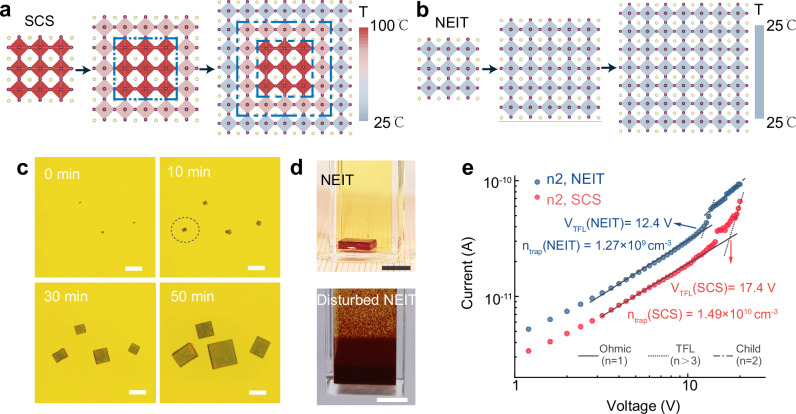
Suppressed strain and trap density in 2D perovskite via NEIT growth. **a, b**, Schematic illustration of perovskite octahedra growth during NEIT and SCS approaches. **c** In-situ monitoring of crystal growth at constant room temperature in NEIT growth, showing optical images during the crystal growth process captured at 0 min (top left), 10 min (top right), 30 min (bottom left), and 50 min (bottom right). Scale bars, 100 µm. **d** Pb-n2 crystals synthesized in undisturbed NEIT solution and in disturbed NEIT solution (ultrasonication for 2 min). Scale bar, 5 mm. **e** SCLC measurements on electron-only devices (Ag/BCP/C_60_/perovskite/C_60_/BCP/Ag) for Pb-n2 synthesized from SCS and NEIT approaches, respectively.

To elucidate the microstructural origin of the superior optoelectronic properties in NEIT-grown 2D perovskite single crystals, we systematically investigated their internal strain profiles and defect concentrations. Crucially, conventional SCS fundamentally compromises crystal integrity: nucleation and growth occur across a substantial thermal gradient ( ~ 100 °C to room temperature)^23,32^. This temperature variation induces non-uniform lattice expansion/contraction, generating pronounced internal strain due to mismatched thermal expansion between adjacent domains (Fig. 3a, b). Such strain inevitably initiates point defects and dislocations as strain-relief mechanisms, ultimately resulting in SCS crystals exhibiting both significant residual tensile strain and elevated defect densities. In contrast, our NEIT strategy operates entirely under constant ambient conditions (room temperature), The growth of Pb-n2, for instance, follows an ultra-slow near-equilibrium process: after cooling to room temperature, the growth solution remains clear for > 36 h before any observable nucleation (Supplementary Fig. 41), ultimately yielding millimeter-scale crystals in the absence of thermal transients. Consequently, lattice coherence is intrinsically preserved in NEIT-grown crystals.

Complementary to suppressed strain formation, NEIT growth concurrently achieves ultra-low nucleation densities—another critical factor governing crystal quality. Real-time optical microscopy tracked growth dynamics within a pre-vibrated (to accelerate the nucleation and enable observation) NEIT quartz cell over 50 min (Fig. 3c and Supplementary Movie 1). Initial observation revealed only three sub-10 μm crystals. Strikingly, merely one additional nucleus formed during the entire 50-minute window, while existing crystals grew steadily to 100–200 μm lateral dimensions. This corresponds to a growth rate of ~1.6-2.9 μm/min, orders of magnitude slower than conventional methods ( ~ 100 μm/min^20^), confirming kinetic control suppressing homogeneous nucleation (Supplementary Fig. 42). Practical large-scale growth batches consistently yielded extremely sparse crystallite populations (often 1–2 crystals per vial; Fig. 3d), directly attributable to the absence of external turbulence, mechanical agitation, and thermal gradients throughout the NEIT growth process. To explicitly demonstrate the necessity of this quiescent environment, we introduced ultrasonic pulses into a Pb-n2 growth solution prior to nucleation. This perturbation triggered the instantaneous formation of numerous fine crystallites, clearly proving that sustained near-equilibrium conditions free of external disturbances are imperative for achieving critically low nucleation densities.

Critically, the synergy between suppressed residual strain and ultra-low nucleation density results in a profoundly reduced trap density. Space-charge-limited current (SCLC) measurements on electron-only devices (Ag/BCP/C_60_/perovskite/C_60_/BCP/Ag) revealed that NEIT-grown n2 possesses an electron-trap density of just 1.27 × 10^9 ^cm^-3^, an order of magnitude lower than the 1.49 × 10^10^ cm^-3^ found in SCS crystals (Fig. 3e and Supplementary Fig. 43). This drastic reduction in bulk trap states underscores the direct causal link between NEIT-controlled microstructure and enhanced electrical performance. Significantly, the universality of this approach was demonstrated by extending NEIT growth to formamidinium (FA)-based system, yielding PEA_2_FAPb_2_I_7_ crystals with ultralong carrier lifetimes reaching 281 ns, the longest reported for pure-phase 2D Ruddlesden-Popper perovskite single crystals (Supplementary Fig. 44 and Supplementary Table 8). Consequently, the NEIT strategy not only delivers macroscopic high-purity crystals but fundamentally rewrites the structural optimization for achieving low-dimensional perovskites with simultaneously minimized strain, defects, and carrier recombination losses.

Epitaxial halide perovskite heterostructures are indispensable platforms for high-performance optoelectronics, yet a persistent compromise exists between interfacial precision and structural scalability. Conventional approaches yield either microscale heterostructures with sharp interfaces or bulk crystals with compositionally diffused interfaces, leaving macroscopic single-crystal heterostructures ( > 1 mm) with abrupt, defect-minimized interfaces unrealized^6,55–57^. Our living NEIT growth strategy overcomes this trade-off by leveraging suppressed homogeneous nucleation and controlled crystallization kinetics, enabling the fabrication of precisely defined, large-scale heterostructures.

**Fig. 4:**
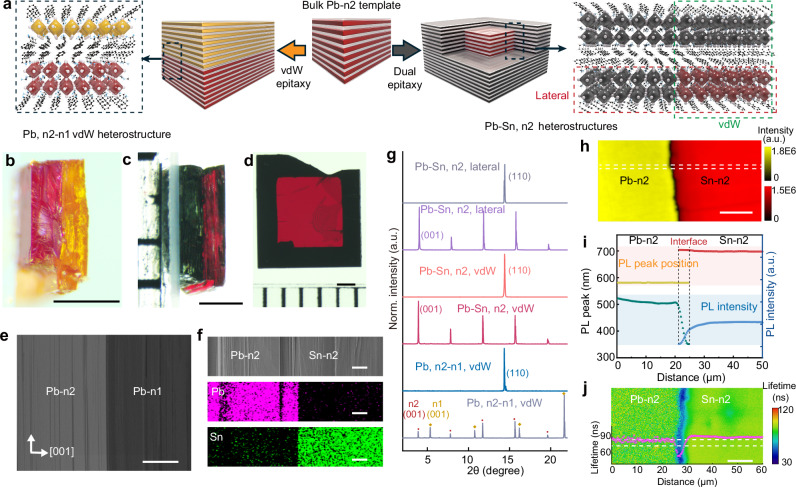
Living NEIT growth of 2D bulk perovskite single-crystal heterostructures. **a** Schematic illustration of three types of bulk heterostructures starting from Pb-n2 single crystals (middle), including two schematics of Pb, n2-n1 vdW heterostructure on the left, and two schematics of Pb-Sn, n2 vdW heterostructure and lateral epitaxial heterostructure on the right. **b–d** Optical images of bulk Pb, n2-n1 vdW (**b**), Pb-Sn, n2 vdW (**c**) and Pb-Sn, n2 lateral (**d**) epitaxial heterostructures. Scale bars, 1 mm. **e** SEM image of Pb, n2-n1 vdW heterostructure. Scale bar, 10 µm. **f** SEM image (top) and corresponding EDS elemental mappings of Pb (middle) and Sn (bottom) for the Pb-Sn, n2 vdW heterostructure. Scale bar, 10 µm. **g** PXRD patterns of Pb, n2-n1 vdW heterostructure, Pb-Sn, n2 vdW and lateral heterostructures in (00k) plane, and 2θ-ω scans from HRXRD characterizations of Pb, n2-n1 vdW heterostructure, Pb-Sn, n2 vdW and lateral heterostructure in (110) plane. **h** The confocal PL mapping of Pb-Sn, n2 lateral epitaxial heterostructure. Scale bar, 10 µm. The left side (yellow) corresponds to Pb-n2 (collected between 530 nm and 630 nm), and the right side (red) corresponds to Sn-n2 (collected between 648 nm and 748 nm). **i** Spatial evolution of PL peak position and PL intensity of Pb-Sn, n2 lateral epitaxial heterostructure. The data are extracted from the dashed white rectangle in (**h**). **j** FLIM pseudo-color image of Pb-Sn, n2 lateral epitaxial heterostructure. The left side corresponds to Pb-n2, and the right side corresponds to Sn-n2. Inset denotes the spatial evolution of carrier lifetime of Pb-Sn, n2 lateral epitaxial heterostructure. Scale bar, 10 µm.

Starting from bulk Pb-n2 templates, the living growth endows two distinct heteroepitaxial growth pathways: 1) van der Waals (vdW) epitaxy in perovskite solution with increased PEAI concentration produces PEA_2_MAPb_2_I_7_-PEA_2_PbI_4_ (Pb, n2-n1) vdW heterostructures, in which two distinct components possess large lattice mismatch (26.8%) along the out-of-plane direction but small lattice mismatch ( < 1.6%) along in-plane direction (Fig. 4a, b, and Supplementary Fig. 45); 2) Seeded epitaxy in PEA_2_MASn_2_I_7_ (Sn-n2) solution generates both vdW-stacked and lateral heterostructures of PEA_2_MAPb_2_I_7_-PEA_2_MASn_2_I_7_ (Pb-Sn, n2), enabled by minimal lattice mismatch ( < 1%) along the (110) crystallographic orientation (Fig. 4a, c, d and Supplementary Figs. 46, 47). Benefiting from the soft ionic lattice and flexible organic spacers of 2D halide perovskites, the <1% lattice mismatch is accommodated without observable macroscopic cracking, while the localized stress is likely released through the formation of interfacial dislocations^6^. Crucially, all heterostructures exhibit lateral dimensions exceeding 1 mm while maintaining structural coherence, evidenced by the corresponding SEM images revealing abrupt interfaces and lamellar stacking continuity (Fig. 4e, f and Supplementary Fig. 47). With Pb-n2 and Pb-n1 possessing different average atomic weight and electronic properties, there is an apparent contrast between two materials and a sharp interface was clearly observed from the SEM image (Fig. 4e). In addition, the shared halide anion and absence of intermediate phases ensure unshifted PL emission at the heterointerface compared to the individual components (Supplementary Fig. 45). For Pb-Sn, n2 heterostructures, although surface roughness in vdW structures somewhat obscures domain contrast in SEM imaging, the sharpness of the interfaces is clearly demonstrated through EDS mapping, which shows spatially confined distributions of Pb and Sn (Fig. 4f). In addition, the sharp interface was corroborated in lateral heterostructures by clear SEM domain contrast and energy-dispersive X-ray spectroscopy (EDS) line scans. The EDS profiles show an abrupt transition between Pb and Sn domains, with no discernible intermixing zone, definitively ruling out interfacial alloying (Supplementary Fig. 47).

Having established these diverse heterostructures, we subsequently validated their epitaxial mechanism and structural coherence. XRD characterizations provide definitive proof of the growth mechanism. For Pb, n2-n1 system, a singular (110) peak at ~14.4° in high-resolution 2θ-ω scans (Fig. 4g) confirms identical in-plane alignment, while distinct (00k) periodicities in PXRD affirm vdW epitaxy without phase mixing. Similarly, in the Pb-Sn, n2 heterostructures, equally spaced (00k) peaks (d = 22.6 Å) observed in both vdW and lateral configurations confirm co-aligned quantum-well stacking. Crucially, a singular (110) peak at 14.4° in the vdW heterostructure cross section (Fig. 4g) further indicates identical in-plane alignment of the Pb and Sn domains, collectively validating the epitaxial growth mechanism across all structures. Furthermore, HRXRD 1D scan and 2D reciprocal space mapping (RSM) of the (110) plane reveal that the diffraction peaks of the pure Pb-n2 and Sn-n2 single crystals align with those observed in the heterostructure, verifying the aligned in-plane orientation of the Pb-Sn, n2 lateral heterostructure (Fig. 4g and Supplementary Figs. 48–51).

Beyond structural epitaxy, the carrier dynamics and charge transport of these heterostructures critically depend on the interface quality, which hinges on suppressed ion interdiffusion. In lateral Pb-Sn, n2 heterostructures, confocal PL mapping (Fig. 4h) reveals negligible PL shift ( < 0.2 nm) in Pb region and minor redshift ( < 4 nm) in Sn region across the interface (Fig. 4i), indicative of minimal Pb/Sn alloying. This compositional sharpness arises from the slow dissolution kinetics of Pb-n2 in MeOH versus the rapid epitaxial growth of Sn-n2 (Supplementary Movie 2 and Supplementary Fig. 52). The kinetic disparity minimizes etching of the template while accelerating heterolayer formation, as further evidenced by EDS elemental confinement of Pb and Sn. Spectroscopically, significant PL quenching at heterointerfaces (Fig. 4i) signals efficient charge separation, characteristic of type-II band alignment where electrons and holes are localized in separate sides. This effect is further quantified by fluorescence lifetime imaging (FLIM, Fig. 4j), which shows a > 35% reduction in carrier lifetime at interfaces (τ_interface_ ~ 54 ns vs. τ_bulk-Pb_ ~ 84 ns and τ_bulk-Sn_ ~ 90 ns), directly confirming accelerated carrier transfer at the heterointerface.

By taking advantage of the engineered band alignment enabled by NEIT-fabricated heterostructures, we demonstrate high-performance lateral Pb-Sn, n2 heterostructure photodetectors. Chemically bonded [PbI_6_]^4-^/[SnI_6_]^4-^ layers facilitate low-resistance interlayer transport. Under 500-nm illumination, these devices exhibit remarkable response speeds, with rise/decay times of 492 μs/340 μs (Supplementary Fig. 53), approximately 60-fold faster than single-component Pb-n2 detectors (29.8 ms/31.3 ms) and over 15000 times faster than Sn-n2 devices ( > 1 s) (Supplementary Figs. 54–56). This dramatic acceleration is assumed to stem from the synergy of type-II-heterostructure driven charge separation and the sharp, chemically bonded interfaces achieved by NEIT growth. Notably, the scalability of NEIT strategy extends beyond n = 2 systems, successfully demonstrated in n = 3 lateral heterostructures (PEA_2_MA_2_Pb_3_I_10_-PEA_2_MA_2_Sn_3_I_10_) possessing equally sharp interfaces (Supplementary Fig. 57). Collectively, these results highlight NEIT growth as a universal paradigm, bridging the critical gap between epitaxial precision and scalable fabrication for next-generation optoelectronics by enabling macroscopic perovskite heterostructures with unprecedented interfacial control.

In summary, the NEIT growth paradigm featuring strong living characteristics suppresses trap densities to ~10^9 ^cm^-3^, resulting in a high PLQY of 77% and long carrier diffusion lengths up to 1.92 μm in 2D lead-halide perovskite single crystals. This precise crystallographic control enables previously inaccessible phenomena, such as cavity-free lasing in 2D n1 lead perovskites and bulk single-crystal heterostructures with sharp interfaces. By resolving the fundamental trade-off between dimensionality control, structural perfection, and optoelectronic performance, the NEIT strategy establishes a versatile platform for engineering quantum-confined semiconductors. Furthermore, the NEIT strategy opens avenues for producing wafer-scale single crystals and heterostructures of lead, tin, and germanium halide perovskites with controlled thickness and interfacial coherence, which will extend their applications beyond optoelectronics into high-performance transistors and thermoelectrics.

## Methods

### Synthesis of PEA_2_PbI_4_ (Pb-n1), PEA_2_MAPb_2_I_7_ (Pb-n2) and PEA_2_MA_2_Pb_3_I_10_ (Pb-n3) single crystals via conventional slow-cooling synthesis (SCS) method

The slow-cooling synthesis (SCS) method is the most widely used methods for synthesizing two-dimensional (2D) Ruddlesden–Popper perovskite single crystals, making it a reliable reference for comparative studies. Perovskite precursor solutions were prepared by dissolving mixtures of phenylethylammonium iodide (PEAI, Greatcell Solar Materials), methylammonium iodide (MAI, Greatcell Solar Materials), and lead iodide (PbI_2_, Tokyo Chemical Industry) in a mixed acid solvent consisting of hydriodic acid (HI, 57 wt.%, Tokyo Chemical Industry) and hypophosphorous acid (H_3_PO_2_, 50 wt.%, J&K Scientific Ltd) at 100 °C until clear solutions were obtained. Specifically, for the synthesis of Pb-n1, 150 mg of PEAI and 850 mg of PbI_2_ were dissolved in 3.6 mL of HI and 0.36 mL of H_3_PO_2_ in a glass vial. For Pb-n2, the precursor comprised 110 mg of PEAI, 350 mg of MAI, and 820 mg of PbI_2_ in the same volumes of acids. Similarly, Pb-n3 was synthesized using 80 mg of PEAI, 430 mg of MAI, and 820 mg of PbI_2_. Each solution was then transferred to a muffle furnace and subjected to a controlled cooling process from 100 °C to room temperature (RT) at a cooling rate of 1.5 °C /h. Three batches of Pb-n1 crystals were grown following this recipe. The Pb-n1 crystals masses were measured as 458.6 mg, 479.4 mg, and 419.9 mg, respectively, with corresponding Pb precursor contents of 220.5 mg, 230.5 mg, and 201.8 mg. The resulting Pb precursor utilization rates were 13.0%, 13.6%, and 11.9%, with an average of 12.8%.

### Synthesis of Pb-n1, Pb-n2 and Pb-n3 single crystals via near-equilibrium isothermal (NEIT) route

Precursors, including PEAI, MAI, and PbI2, were dissolved in methanol (MeOH, Sigma-Aldrich and Innochem) at 60°C for approximately 12h. Specifically, for Pb-n1, 700 mg of PEAI and 461 mg of PbI_2_ were dissolved in MeOH in a glass vial. For Pb-n2, a mixture of 1000 mg PEAI, 260 mg MAI and 922 mg PbI_2_ were used. For Pb-n3, 300 mg PEAI, 170 mg MAI and 461 mg PbI_2_ were dissolved. Each solution was filtered through a 0.22 μm nylon membrane to remove particulates. The filtered clear solution was subsequently cooled from 60 °C to 25 °C at a controlled rate of 1.05 °C/h to minimize disturbances. The solution was then maintained at RT. It remained optically clear for the first 48 h or longer, followed by gradual crystallization. Following the NEIT route, two batches if Pb-n1 crystals were grown. The dissolved PbI_2_ in the precursor solutions was 191.1 mg and 179.0 mg, respectively (with corresponding undissolved PbI_2_ masses of 269.9 mg and 282.0 mg after filtration). The masses of the resulting Pb-n1 crystals were 356.4 mg and 327.2 mg, containing Pb precursor of 171.3 mg and 157.3 mg, respectively. The corresponding Pb utilization rates were 89.7% and 87.9%, averaging 88.8%, marking a 5.9‑fold improvement over the Pb utilization achieved via the SCS route.

### Synthesis of PEA_2_MAPb_2_I_7_-PEA_2_PbI_4_ (Pb, n2-n1) vdW heterostructures

A clear precursor solution was prepared by dissolving 2000 mg of PEAI, 260 mg of MAI, and 922 mg of PbI_2_ in MeOH, followed by filtration. Bulk Pb-n2 single crystals were then immersed in this solution to initiate vdW epitaxial growth of Pb, n2-n1 vdW heterostructures.

### Synthesis of PEA_2_MAPb_2_I_7_-PEA_2_MASn_2_I_7_ (Pb-Sn, n2) vdW and lateral heterostructures

The NEIT grown Pb-n2 bulk single crystals were immersed in a clear Sn-n2 precursor solution (SnI_2_, Shanghai Macklin Biochemical Technology Co., Ltd). Epitaxial growth of Sn-n2 proceeded on all six faces of the Pb-n2 cuboid, resulting in a core–shell architecture with a red Pb, n2 core encapsulated by a black Sn-n2 shell. In this structure, vdW heterostructure was realized along the (00k) plane, where Sn-n2 adheres to the top and bottom surfaces of the Pb-n2 crystal via vdW stacking. Lateral heterostructures were formed through chemical epitaxy along the (110) plane, and could be isolated via removal of the vdW stacked Sn-n2 by mechanical exfoliation. Similar methodologies were applied to synthesize Pb-Sn, n3 vdW and lateral epitaxial heterostructures, wherein Pb, n3 crystals were immersed in a clear Sn, n3 precursor solution to facilitate the epitaxial growth (PDMS, Shanghai Onway Technology Co., Ltd).

### Single-crystal and power XRD (PXRD) measurements

Single-crystal XRD data were collected at RT on a Bruker AXS D8 Venture diffractometer equipped with Mo K_α_ radiation, (λ = 0.71073 Å). During the initial structure determination, the XPREP processing system suggested the space group P-1 based on systematic absences. However, refinement in this space group resulted in symmetry-induced disorder within the organic moiety, manifesting as overlapping electron density with multiple occupancies for several atomic sites. Upon lowering the crystallographic symmetry to P1, the disorder was fully resolved, yielding a well-ordered model for the organic component. Given that structural disorder is undesirable for subsequent theoretical calculations and to maintain consistency with previously reported analogous structures, the structure was refined in the space group P1.

PXRD was performed on a Bruker D8 Advance instrument using Cu Kα radiation (λ = 1.5406 Å). For PXRD characterizations of lateral epitaxial heterostructure, the sample was mechanically exfoliated to reveal a fresh surface and mounted directly onto the sample holder. To analyze vdW heterostructure along the (001) direction, the heterostructure was carefully cleaved near the interface to partially expose the underlying perovskite layer while leaving part of the top layer, ensuring the X-ray beam penetrating both components.

### High-resolution X-ray diffraction (HRXRD) characterization

All high-resolution X-ray measurements, including rocking curve, 2θ/ω scan, reciprocal space mapping (RSM), and X-ray reflectivity (XRR), were conducted on a Bruker D8 Discover diffractometer equipped with a point-focused Cu Kα source (beam diameter: 0.5 mm). Rocking curve measurements of Pb- n2 crystals obtained from both NEIT and SCS systems were performed under consistent experimental conditions, including beam diameter, step size, and integration time.

### Optical and photoluminescence (PL) characterizations

Optical images were captured using a Nikon LV100ND microscope. PL images were obtained under excitation from a mercury lamp source (C-LHGFI HG Lamp). PL spectra and confocal PL mapping were performed on a WITec Alpha300RAS confocal Raman microscopy system. The PL spectra related to PL quantum yield (PLQY) were collected using Andor Shamrock SR-500I-B1. Spectral intensities were quantified using a CCD detector (Andor PU420A-OE). Angle-resolved PL measurements were using two complementary systems to cover the full emission profile. The low-angle emission (0 to ±62°) was collected on a home-built system consisting of a 100× objective (NA = 0.9), a Princeton Instruments spectrometer, and a PIXIS-1024B detector. The high-angle emission (62° to 90°) was acquired using a circularly polarized luminescence spectrometer equipped with a molecular orientation unit (JASCO CPL-300). Polarization-resolved PL spectroscopy was conducted on a WITec Alpha300RAS confocal Raman microscopy system. A linearly polarized light was used as the excitation source. A polarizer was inserted into the signal collection path. The emission signals were recorded at different polarization angles by rotating the polarizer with a step size of 15°.

### UV-Vis absorption spectra measurement

UV-Vis absorption spectra were collected by CRAIC 20/30PV Shimadzu UV-VIS-NIR microspectrophotometer.

### Time-resolved PL (TRPL) and fluorescence lifetime imaging microscopy (FLIM) measurements

TRPL decay spectra were acquired using a TPL 200 transient fluorescence spectrometer (Time-Tech Spectra LLC). Emitted photons were detected with a single-photon avalanche diode (PMA hybrid-04, PicoQuant), and their arrival times were recorded by a time-correlated single-photon counting module (CNT-91, Pendulum). The photon counting rate was kept below 5% of the excitation repetition rate to avoid pulse pile-up. The instrument response function (IRF) was characterized by measuring the full width at half maximum ( < 300 ps) of the reflected signal from a clean silicon surface at the excitation wavelength. All measurements were performed at a repetition rate of 100 kHz. FLIM was carried out on a Microtime200 system (PicoQuant) equipped with a 100× oil-immersion objective. The samples were excited using a 405 nm laser with a repetition rate of 4 MHz and laser power of 5.4 × 10^-3 ^µW. The diffraction-limited Airy disk radius was calculated to be 218.34 nm, yielding a high localized excitation fluence of ~900 nJ/cm^2^.

### SEM and EDS mapping

SEM imaging and EDS mapping were conducted using a Zeiss Gemini450 scanning electron microscope. SEM imaging was conducted at an accelerating voltage of 3 kV with a probe current of 200 pA, while EDS mapping was performed at a higher accelerating voltage of 10 kV and probe current of 500 pA to ensure sufficient X-ray excitation. For the cross-sectional analysis, heterostructures were mechanically cut perpendicular to the (00k) plane using a sharp blade.

### AFM and KPFM

AFM and KPFM measurements were performed at room temperature under a N_2_ atmosphere using a Cypher ES atomic force microscope (Oxford Instruments).

### Lasing measurement

Lasing spectroscopy measurements were carried out using a home-built microspectroscopy system. A mode-locked Yb: KGW laser (Pharos, Light Conversion Ltd.; 10 kHz repetition rate, 170 fs pulse width) operating at a fundamental wavelength of 1030 nm was coupled to an optical parametric amplifier (Orpheus-HP, Light Conversion Ltd.) to generate tunable excitation wavelengths. The pump beam was focused onto mechanically exfoliated samples through a 50× NIR objective (NA = 0.20). A plano-convex lens (f = 500 mm) was inserted to expand the beam spot, ensuring uniform illumination across the sample surface. Samples were mounted on SiO_2_/Si substrates affixed with cryogenic adhesive inside a vacuum chamber and dried for ~12 h to remove solvent residues. Freshly exfoliated flakes were transferred within a nitrogen glovebox onto the prepared substrates. All lasing characterizations were performed at 80 K under high vacuum ( < 10^-4 ^Pa). An excitation wavelength of 490nm was used for the Pb-n1, Pb-n2, and Pb-n3 samples (with a 500 nm long-pass filter, LBTEK). The coherence time of the lasing emission was measured using a custom-built Michelson interferometer optical setup. The resulting interferometric signals were collected and detected by a Princeton Instruments (PI) spectrometer.

### Device fabrication and photodetector characterization

Trap state densities were evaluated using SCLC measurements in electron-only devices with a device structure of Ag (120 nm) /BCP (6 nm) /C_60_ (35 nm) /Perovskite /C_60_ (35 nm) /BCP (6 nm) /Ag (120 nm). Dark current–voltage characteristics were acquired with a Keithley 2636 A source meter. To quantify the trap density and suppress potential artifacts from transient mobile charges or contact asymmetry, we employed a strictly controlled pulsed-voltage SCLC technique on symmetric electron-only devices^58^. The SCLC measurements were conducted at a low scan rate (step size 0.4 V, transient pulse integration time of ~200 ms, and a pulse delay of 2 s) inside a dark electromagnetic shielding box. Furthermore, the exact cross-sectional thickness of each measured single crystal was directly determined via optical microscopy.

Photodetectors were fabricated via dry transfer of Au electrode arrays (40 × 40 μm^2^ area, 10 μm channel width) onto Pb-Sn, n2 lateral heterostructures. Electrical characterizations were performed with a Keithley 2636B source meter and a Tektronix MDO34 oscilloscope. A 500 nm LED served as the excitation source. The Sn-n2 terminal was biased, and the Pb-n2 side was grounded. All measurements and device handling were conducted in a N_2_-filled glovebox to prevent oxidation.

### Reporting summary

Further information on research design is available in the Nature Portfolio Reporting Summary linked to this article.

## Supplementary information

Supplementary information

Description of additional Supplementary files

Supplementary Movie 1

Supplementary Movie 2

Reporting Summary

Transparent Peer Review file

## Source data

Source Data

## Data Availability

Crystallographic data for the single crystals reported in this article have been deposited at the Cambridge Crystallographic Data Center (CCDC), with deposition numbers 2487055 (PEA_2_PbI_4_ synthesized from NEIT growth), 2487054 (PEA_2_MAPb_2_I_7_ synthesized from NEIT growth), 2487053 (PEA_2_MA_2_Pb_3_I_10_ synthesized from NEIT growth), 2487052 (PEA_2_FAPb_2_I_7_ synthesized from NEIT growth). Source data are provided with this paper.
