# Supplementary material for: Living growth of ultra-bright 2D perovskites with long-lived carriers
Source: Nat Commun. 2026 Jul 14;17:8629. doi: 10.1038/s41467-026-75434-6 (PMC13487158; doi:10.1038/s41467-026-75434-6)
Supplement: Supplementary file 1 [file 41467_2026_75434_MOESM1_ESM.pdf]

Supplementary information for

Living growth of ultra-bright 2D perovskites with long-lived carriers

Table of Contents

1. Supplementary Characterizations
2. Supplementary Figures 1-57
3. Supplementary Tables 1-8
4. Supplementary Videos 1-2

Supplementary Methods

1. The PL quantum yield (PLQY) measurements

All PLQY measurements were performed on mechanically exfoliated nanoflakes. The thickness of each flake was fitted using an established thickness-transmittance calibration curve. Both NEIT and SCS flakes tested possessed comparable nanometer-scale thicknesses.

Differential transmission micro-spectroscopy was conducted at RT using a confocal microscope system. The absorptivity (A) was determined according to the following equation:

$$A = 1 - R - T = 1 - R_{quartz} \times \frac{IR'}{IR} - \frac{IT'}{IT}$$

R_{quartz}: Reflectance constant of transparent quartz substrate. IR': Laser intensity of reflected light on the sample. IR: Laser intensity of reflected light on quartz substrate. IT': Laser intensity of transmitted light on the sample. IT: Laser intensity of transmitted light on quartz substrate. The PLQY of Pb-n1, Pb-n2 and Pb-n3 flakes was estimated based on a referenced method [1], using a spin coating film of 8-hydroxyquinoline aluminum (Alq_3) in poly(methyl methacrylate) (PMMA) (500 μM, spin at 3000 rpm for 60 s) as a standard. Given the sensitivity of Alq_3 PLQY to film thickness and concentration, the reference value was independently calibrated as 13.23% using an Edinburgh Instruments FLS1000+FS5+Pico1000 system. The PLQY of Alq_3 was assumed to be independent of excitation wavelength and laser intensity. The PLQY of Pb-n1, Pb-n2 and Pb-n3 flakes was then calculated using the formula:

$$\Phi_{\text{sample}} = \Phi_{\text{reference}} \times \frac{I_{sample}^{pl}/A_{Sample}}{I_{reference}^{pl}/A_{reference}}$$

Where Φ, I, A are PLQY, PL intensity, and absorptivity of the sample and reference. The measurements related to PL and absorption were performed using Andor Shamrock SR-500I-B1. Spectral intensities were quantified using a CCD detector (Andor PU420A-OE).

To account for the intrinsic optical anisotropy of 2D layered perovskites, the initial PLQY values were recalibrated. Isotropic low-angle emission within the objective's

collection limit (-62° to +62°) was confirmed using a home-built angle-dependent PL system (100× objective, NA = 0.9, Princeton Instruments spectrometer, PIXIS-1024B detector). The high-angle (62° to 90°) emission profile was extracted on thin films fabricated by immediately spin-coating the rapidly dissolved single crystals using a JASCO CPL-300 spectrometer equipped with a molecular orientation unit [2-4]. Integrating these two sets of emission profiles (low-angle and high-angle) over the full angular range yielded specific anisotropy correction factors (0.804 for Pb-n1, 0.827 for Pb-n2, and 0.780 for Pb-n3). By multiplying the initial values by these respective factors, the PLQY values for all 2D perovskite single crystals were recalibrated, thereby correcting the overestimation induced by structural confinement.

2. Carrier diffusion constants via time-resolved photoluminescence microscopy (TRPLM)

Diffusion imaging setup: Carrier diffusion constants were quantified using a home-built TRPLM system. A near diffraction limited exciton population was obtained by a 454 nm laser (PicoQuant LDH-D-C-454, PDL 800-D) and focused through a ×60 dry objective (Nikon S Plan Fluor, NA = 0.7). A f = 1000 mm lens, placed one tube length (200 mm) away from the back focal plane of the objective. Fluorescence of the exciton population was magnified 60 × 1000/200 = 300 times, and then focused on the TCSPC with a detector active area of 50 × 50 mm^2. The detector was manipulated by an electric two-dimensional translation stage to facilitate scanning over the exciton population.

Carrier diffusion constants: Briefly, the dynamics of the exciton distribution over space and time can be modeled by the equation:

$$\frac{\partial n(x,y,t)}{\partial t} = k_1 n(x,y,t) - D\nabla^2 n(x,y,t) \qquad (1)$$

Where k_1 is the exciton recombination rate, and D is the diffusion constant. The initial exciton population profile is dictated by the Gaussian excitation beam $n_0 = A_0 \exp\left(\frac{(x-x_0)^2-(y-y_0)^2}{2\sigma_0^2}\right)$. Over time *t*, the exciton diffusion leads to a broader Gaussian distribution: $n_t = A_t \exp\left(\frac{(x-x_0)^2-(y-y_0)^2}{2\sigma_t^2}\right)$.

For isotropic materials, the diffusion simplifies to a one-dimensional model. Linear

regression of mean squared displacement (MSD) versus time delays (t) revealed a diffusion coefficient derived from:

$$D = \frac{\sigma_t^2 - \sigma_0^2}{2t} \qquad (2)$$

Values were averaged across multiple single-crystalline flakes. Measurements at low and high excitation densities confirmed the absence of exciton-exciton interactions, ensuring the reported values reflect intrinsic material properties.

3. Calculation method for PbI_2 utilization rates

This section clearly presents the calculation details for both the NEIT and SCS methods. The raw experimental data are summarized in Supplementary tables 1-2.

For the NEIT method, the PbI_2 utilization rate represents the conversion of the dissolved PbI_2 precursor into the final Pb-n1 crystal. Since PEAI is fully dissolved in MeOH and only partial PbI_2 remains undissolved, the dissolved PbI_2 mass is calculated as the total initial PbI_2 input mass minus the mass of filtered undissolved PbI_2. The equivalent mass of PbI_2 within the Pb-n1crystal is determined by its stoichiometric ratio (e.g., for PEA_2PbI_4, the PbI_2 mass is the final crystal mass multiplied by the molar mass ratio of 461/959). The formula is:

$$PbI_2\ \ Utilization_{NEIT}\ (\%) = \left(\frac{Mass\ of\ equivalent\ PbI_2\ in\ the\ grown\ crystal}{Mass\ of\ dissolved\ PbI_2\ in\ the\ solution}\right) \times 100$$

In the SCS method, all initially added PbI_2 is fully dissolved in the solution, so the utilization rate for SCS be calculated based on the total initial PbI_2 added. The equivalent mass of PbI_2 within Pb-n1 crystals is identical to that in the NEIT system. The formula is:

$$PbI_2\ \ Utilization_{SCS}\ (\%) = \left(\frac{Mass\ of\ equivalent\ PbI_2\ in\ the\ grown\ crystal}{Total\ initial\ mass\ of\ PbI_2\ added}\right) \times 100$$

Supplementary Figures 1-57

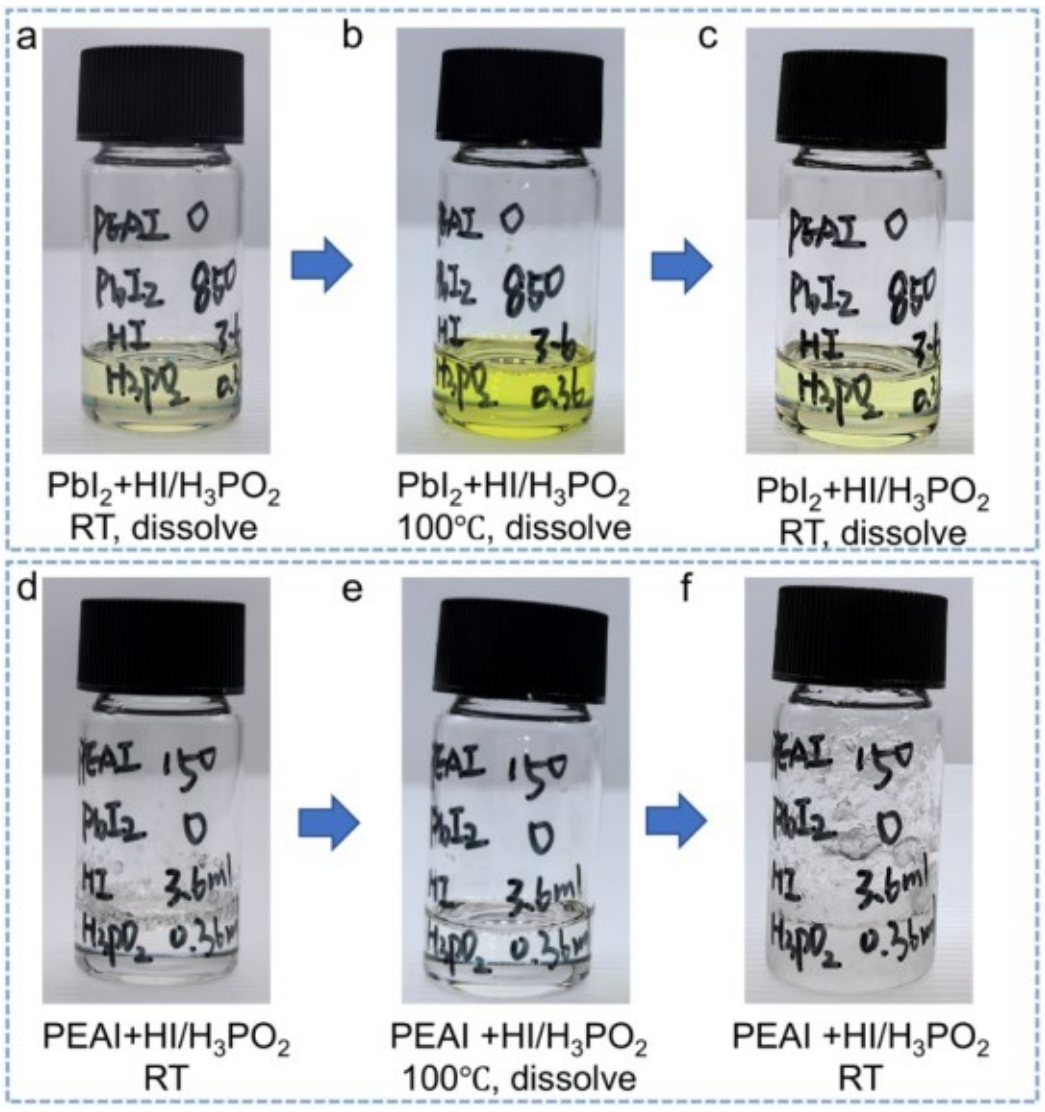

Supplementary Fig. 1. Dissolution and precipitation behaviors of precursors in the aqueous HI/H_3PO_2 system. a-c. PbI_2 dissolves in the HI/H_3PO_2 mixture at both RT and 100 ℃. **d-f**. PEAI dissolves at 100 ℃ (e), but forms precipitates upon cooling to RT (f).

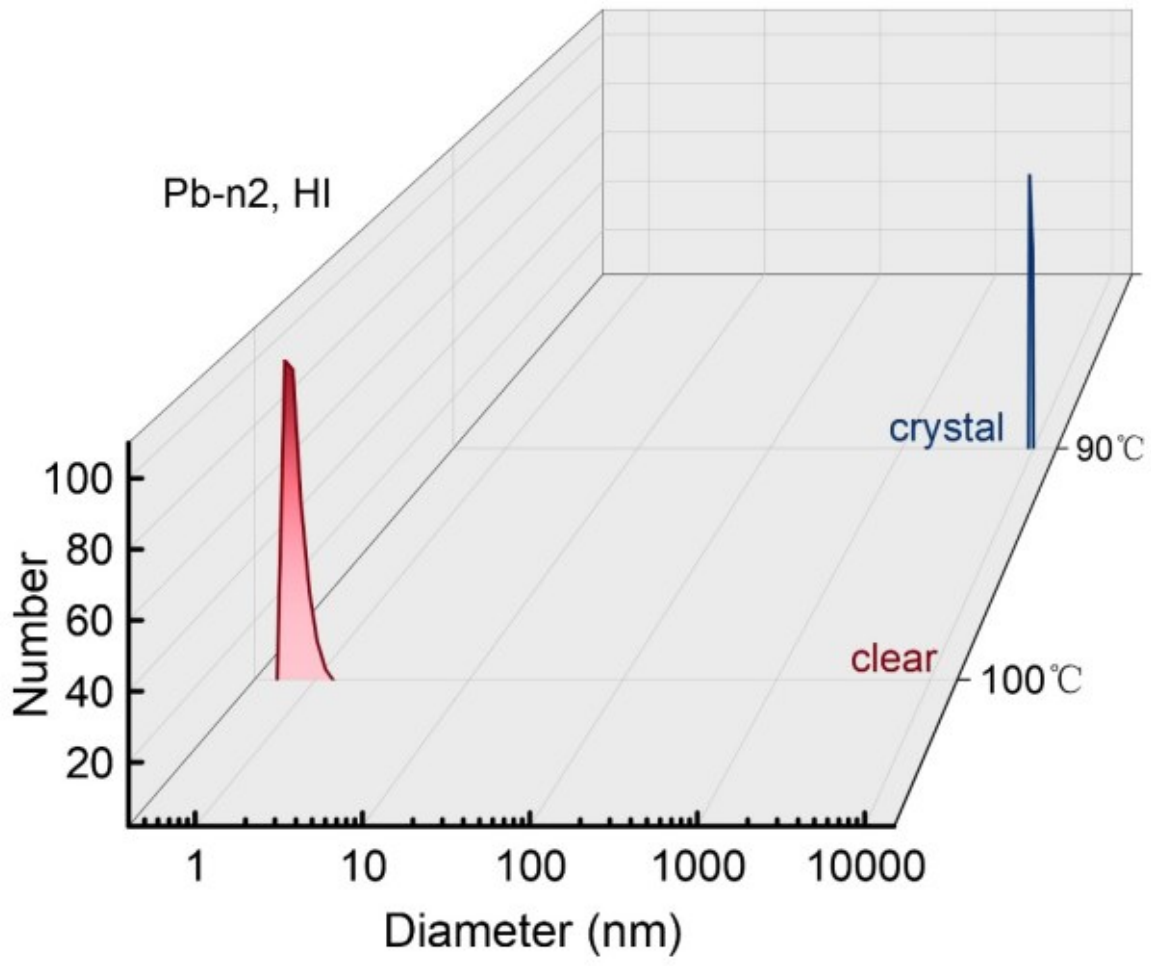

Supplementary Fig. 2. Quasi-in-situ dynamic light scattering (DLS) tracking of precursor solution in the HI system. Number distribution of the Pb-n2 precursor solution during cooling. A molecular-scale peak (~2 nm) is observed at 100 ℃, while cooling to 90 ℃ triggers the appearance of macroscopic scattering signals (>1000 nm).

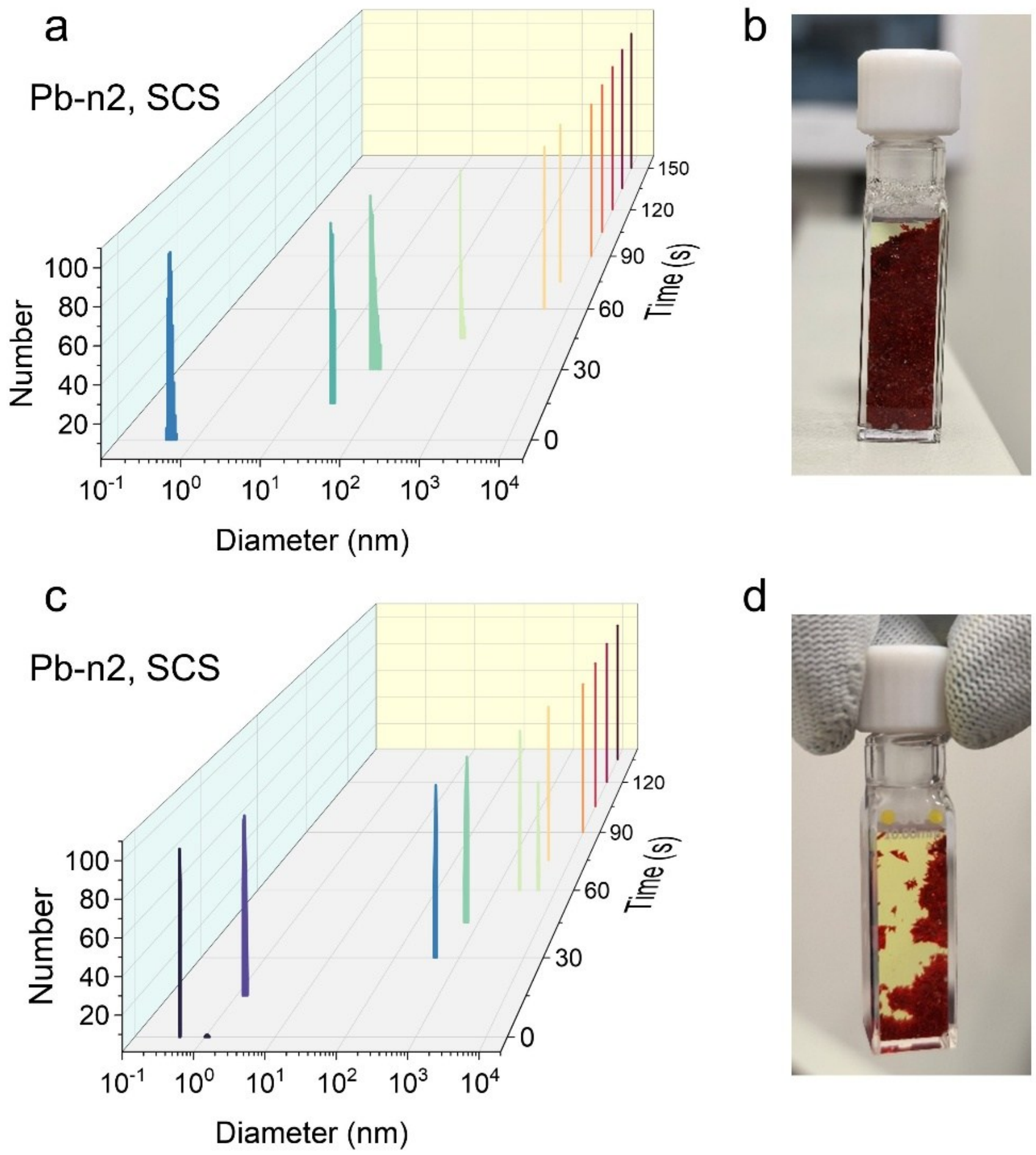

Supplementary Fig. 3. In-situ DLS tracking of the Pb-n2, SCS system. In situ DLS distribution of the Pb-n2, SCS precursor solution during natural cooling. Due to the rapid crystallization kinetics of the SCS method, measurements were initiated immediately upon transferring the hot, clear solution into the instrument (duration: 15 s; interval: 0 s). a, c. Two independent continuous in situ tracking profiles showing the rapid emergence of macroscopic scattering signals. b, d. Images of the corresponding solution immediately after the DLS measurements, revealing massive and rapid crystallization.

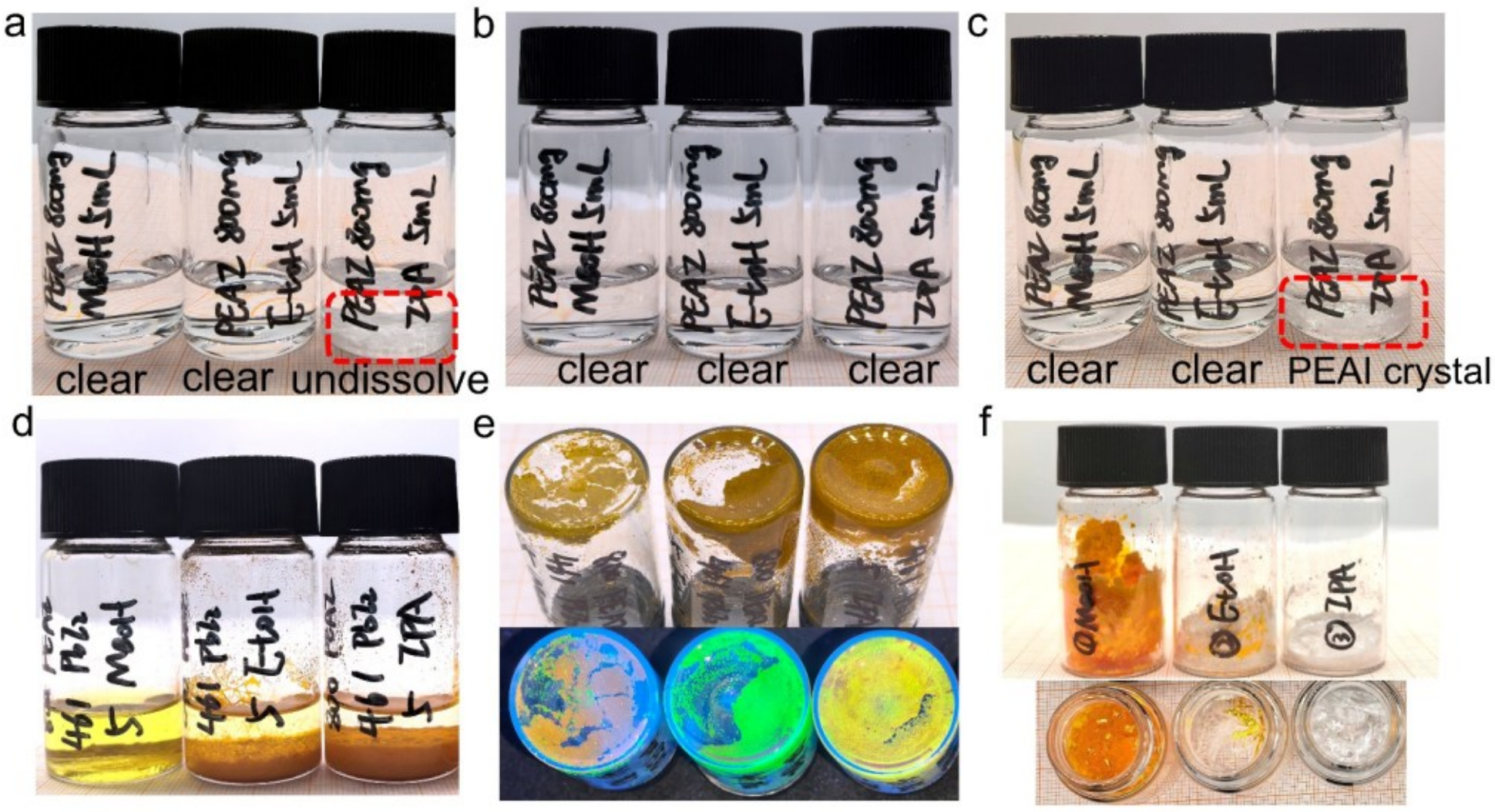

Supplementary Fig. 4. Solubility and complexation behaviors of perovskite precursors in different alcohol solvents. a. PEAI (800 mg) in 5 mL of MeOH, EtOH, and IPA after 5 minutes of stirring at RT. PEAI is readily dissolved in MeOH and EtOH to form clear solutions but remains partially undissolved in IPA. **b.** Complete dissolution of PEAI in all three solvents achieved after continuous heating at 60 °C for 12 hours. **c.** Precipitation behaviors after cooling these solutions to RT. PEAI crystals exclusively precipitate from the IPA solution. **d.** Dissolution states of PEAI (800 mg) and PbI_2 (461 mg) mixtures in 5 mL of MeOH, EtOH, and IPA after 12 hours of heating at 60 °C. **e.** Optical and UV-illuminated images of the filtered undissolved residues from the respective mixtures in (d). **f.** Solid products obtained by evaporating the filtered supernatants in a vacuum oven at 60 °C.

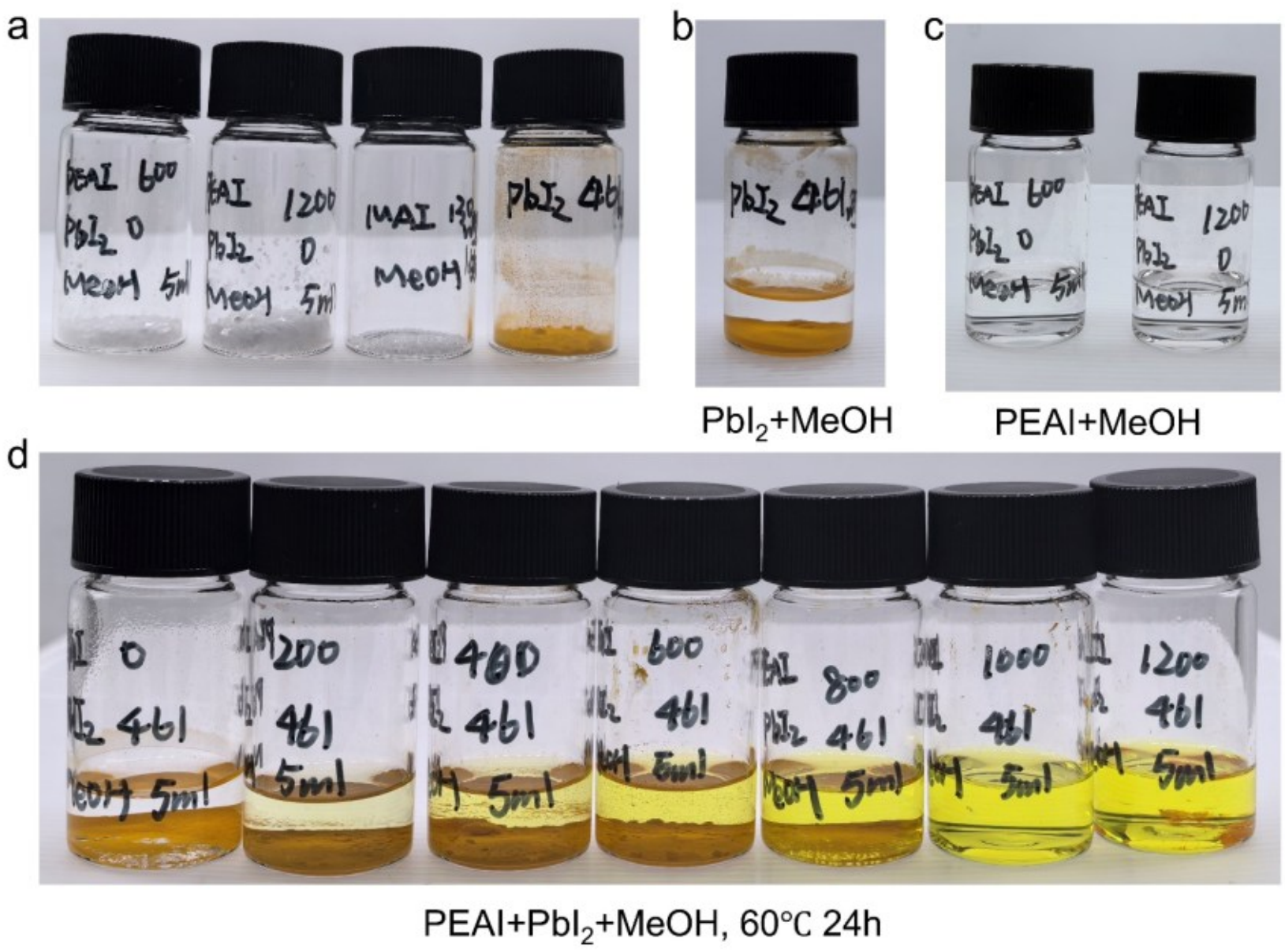

Supplementary Fig. 5. Complexation-driven dissolution of PbI_2 in MeOH system. **a, b,** Pure PbI_2 exhibits limited solubility in MeOH (5 mL) even at 60 ℃ for 24 h. **c.** The PEAI dissolves in MeOH at RT. **d.** With a fixed amount of PbI_2 (461 mg) in 5 mL MeOH at 60 ℃, increasing the PEAI (from 0 to 1200 mg) leads to the progressive dissolution of PbI_2.

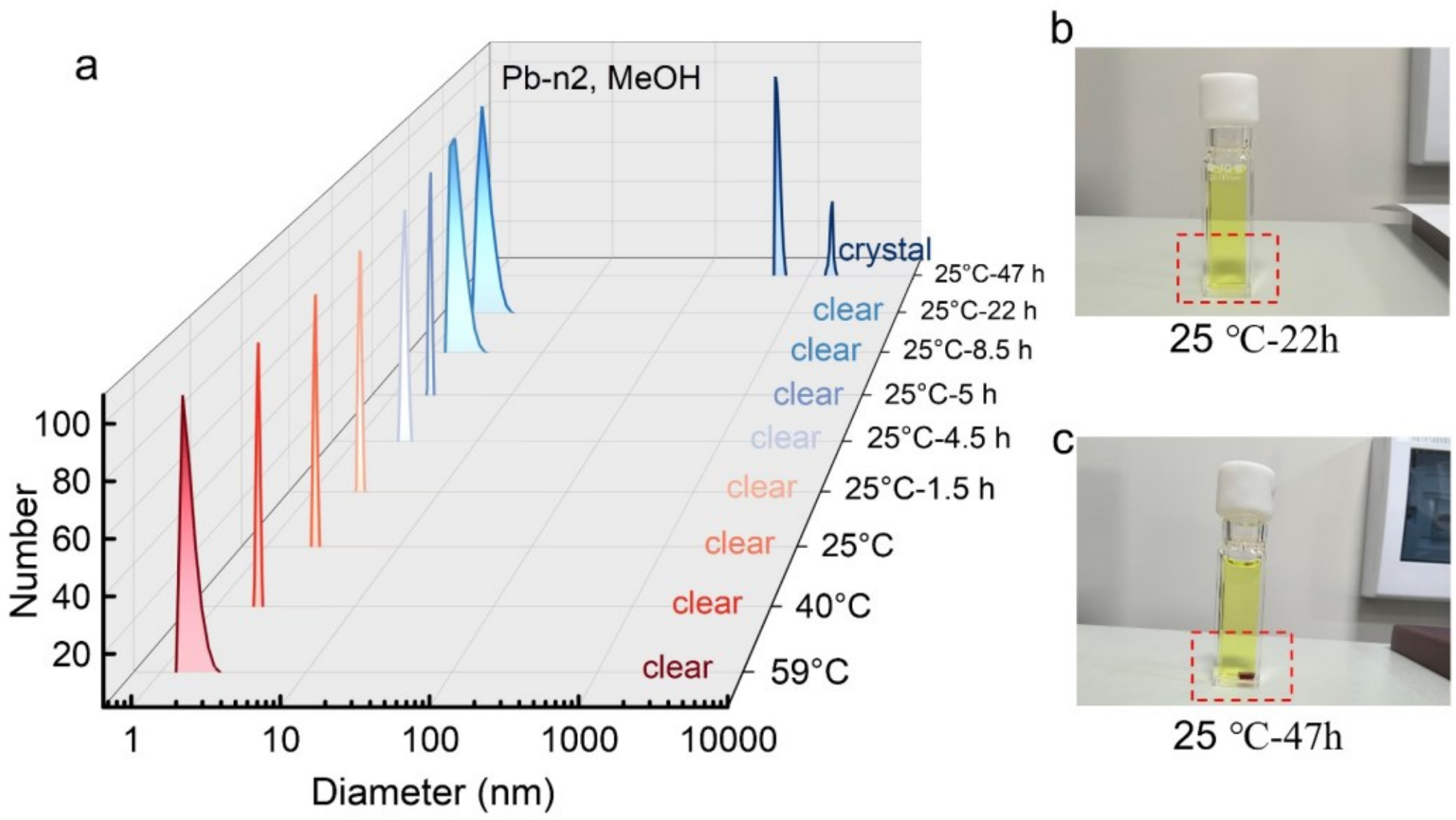

Supplementary Fig. 6. Quasi-in-situ DLS tracking of the MeOH system. a. DLS distribution of the Pb-n2 precursor solution during cooling (59 ℃ to 25 ℃) and RT aging at 25 ℃. The profile maintains a peak at ~1-2 nm for over 22 hours. (Note: The initial temperature was set to 59 ℃ due to the DLS instrument's safety restriction, which must remain below the boiling point of MeOH). **b,** The solution remains visually clear after 22 h at RT. **c,** After 47 h, a crystal appears at the bottom of the vial. The emergence of the macroscopic signal (>1000 nm) at 47 h in (a) may attributed to mechanical disturbances.

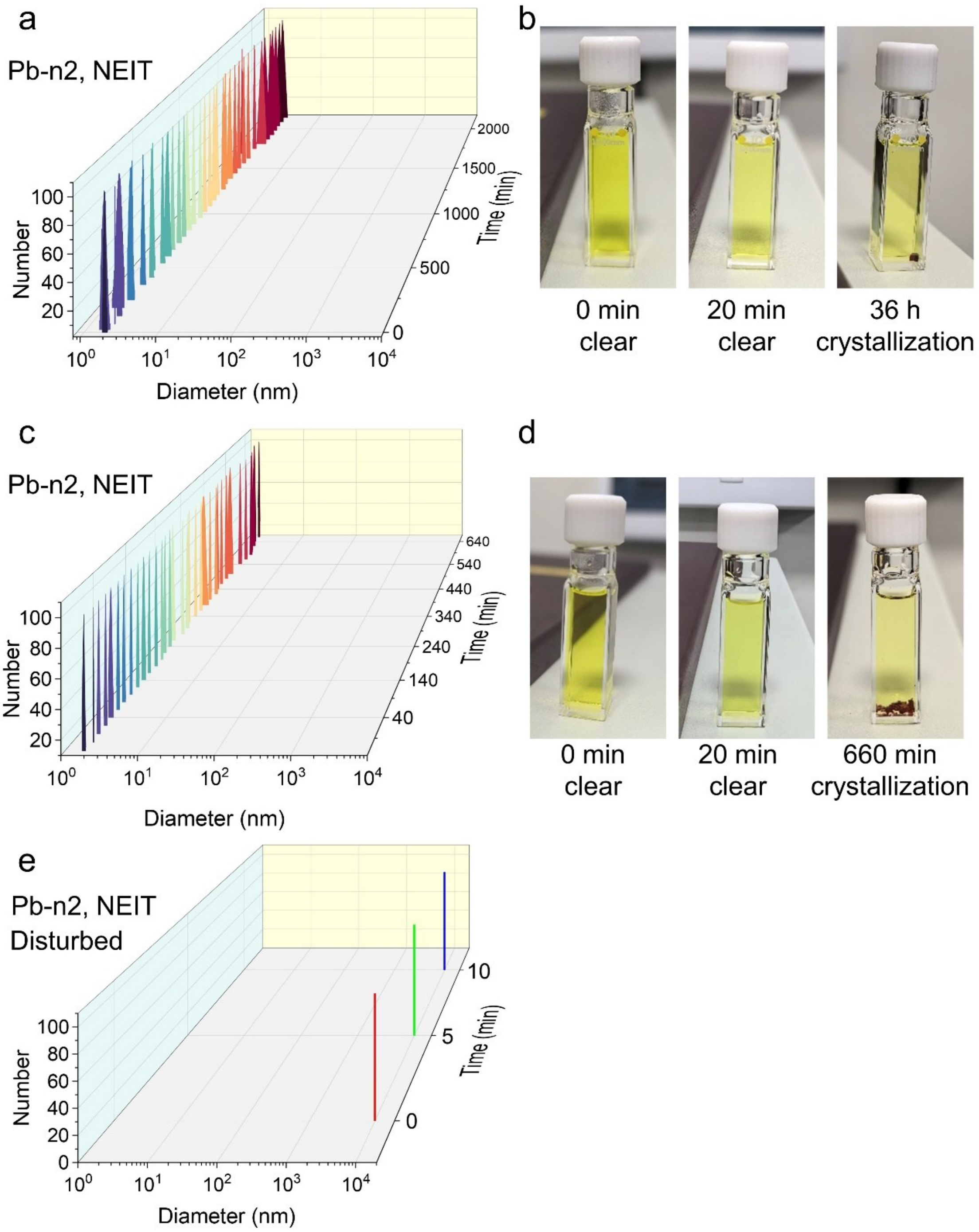

Supplementary Fig. 7. In-situ DLS tracking of the Pb-n2, NEIT system. a. Continuous tracking during the initial cooling (first 20 min) and subsequent RT (measured hourly). The profile maintains peak at ~1-2 nm throughout the entire 36 h process until crystallization. This may because the DLS laser path probes the mid-level supernatant, thus bypassing the bottom-nucleated crystal and detecting only the stable residual precursors. **b.** Images corresponding to (a) at 0 min (hot clear solution), 20 min (cooled to RT, clear), and 36 h (crystallization). **c.** DLS tracking of another sample to

monitor accelerated growth. After maintaining a ~1-2 nm peak during the initial 20 min cooling, the solution was intentionally shaken to accelerate nucleation and measured every 20 min. The signal persistently remained at ~1-2 nm up to 660 min. **d.** Images corresponding to (c) at 0 min (hot clear solution), 20 min (cooled to RT, clear), and 660 min (crystallization). **e.** DLS profile of the crystallized solution from (c) measured immediately after intentional mechanical disturbance (shaking), which instantly triggered the appearance of macroscopic scattering signals (~10000 nm). Note: Unlike previous quasi-in situ measurements where sample transfer caused mechanical disturbance, the steady-growth tracking in a and c was strictly in situ and undisturbed.

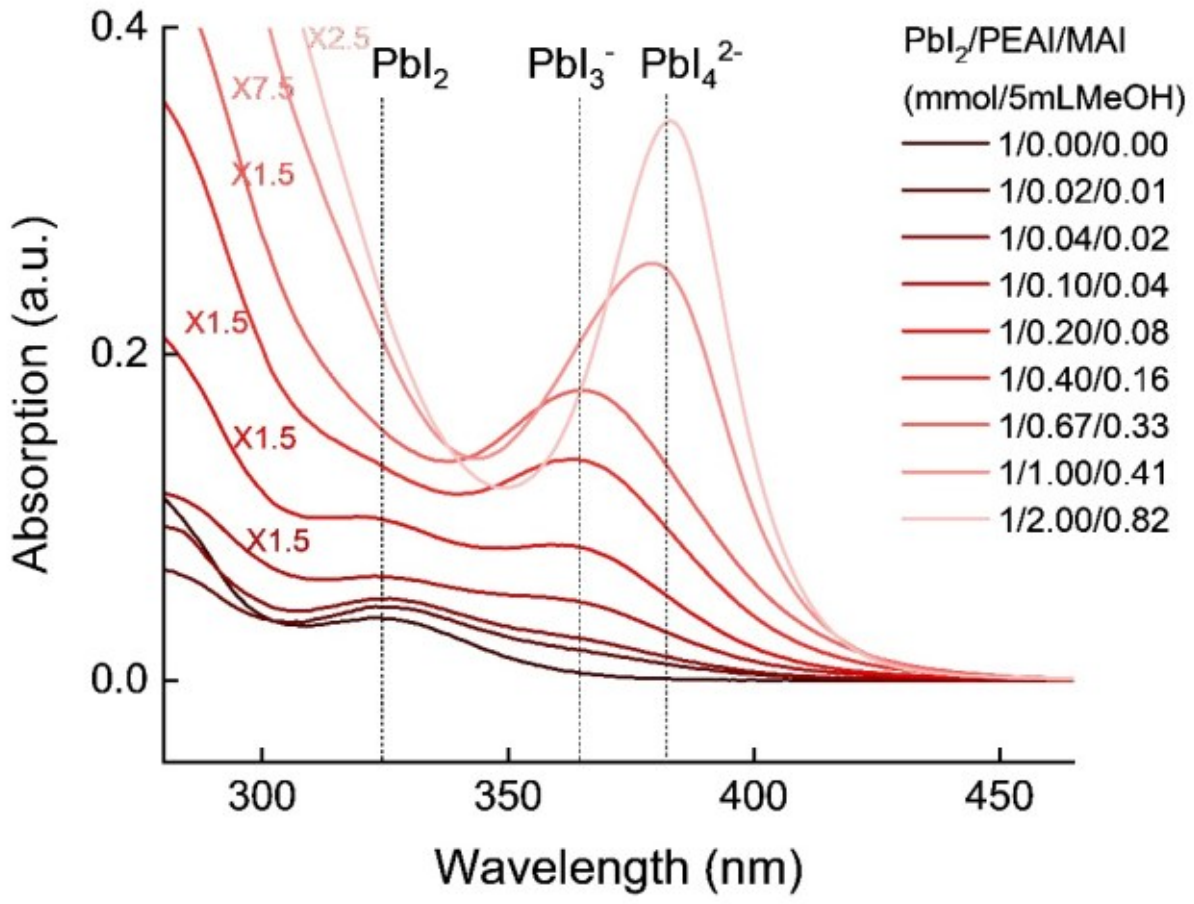

Supplementary Fig. 8. UV-Vis spectrum of the perovskite precursor solution in MeOH.

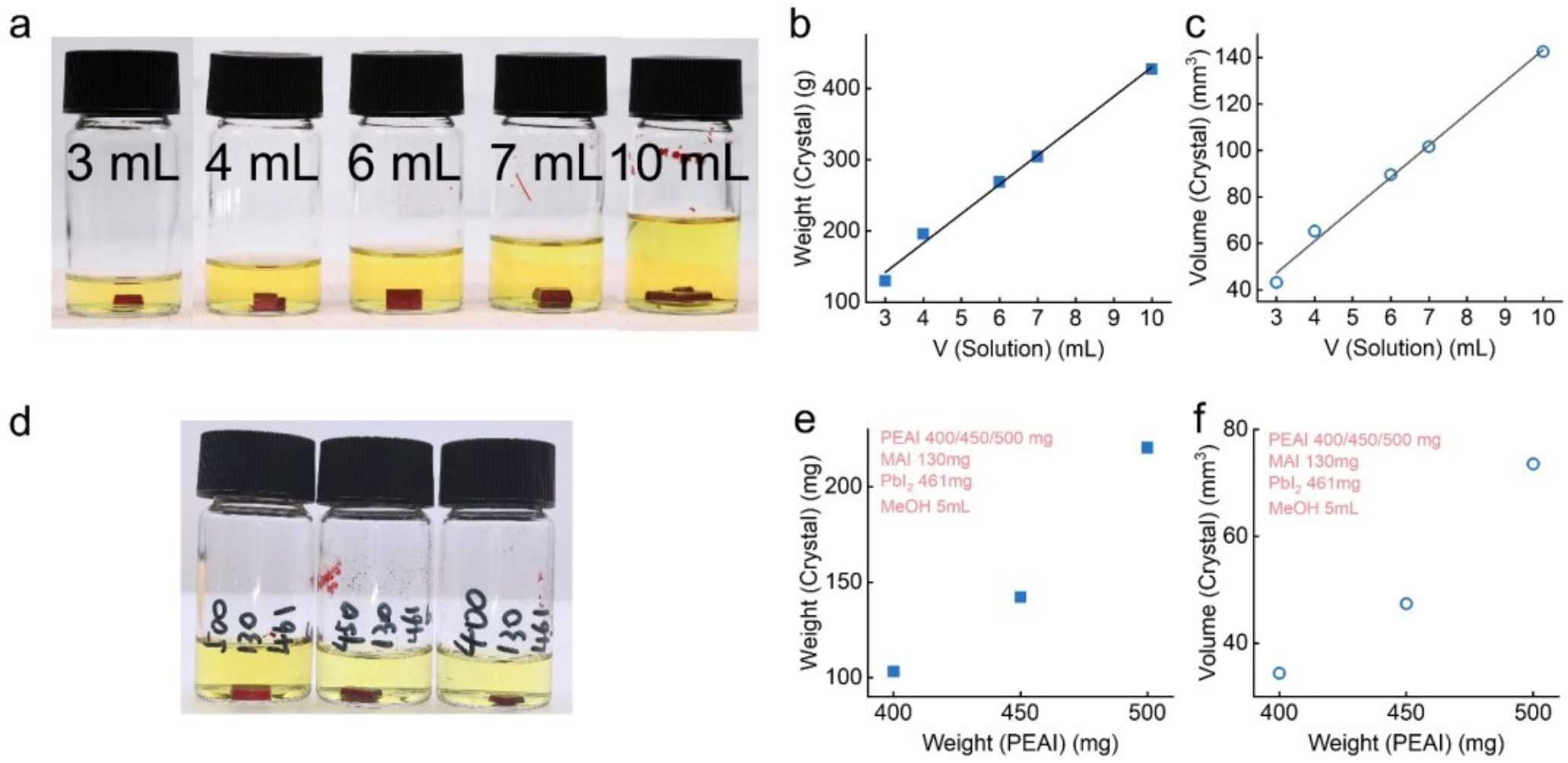

Supplementary Fig. 9. Tailoring the Pb-n2 crystal sizes in the NEIT system. a-c, Crystal size tailoring by solution volume: (**a**) Images of single crystals (Pb-n2) grown in varying precursor solution volumes (from 3 to 10 mL) with a fixed concentration. The final crystal weight (129.7, 195.8, 268.7, 304.9, and 427.5 mg) (b), and calculated volume (43.3, 65.3, 89.6, 101.7, and 142.6 mm^3) (c) scale linearly with the initial solution volume. **d-f,** Crystal size tailoring by initial concentration: Images of single crystals (Pb-n2) grown with varying PEAI masses (500, 450, and 400 mg) in 5 mL MeOH to modulate the initial dissolved solute concentration (d). The final crystal weight (e) and calculated volume (f) show a clear dependence on the PEAI mass.

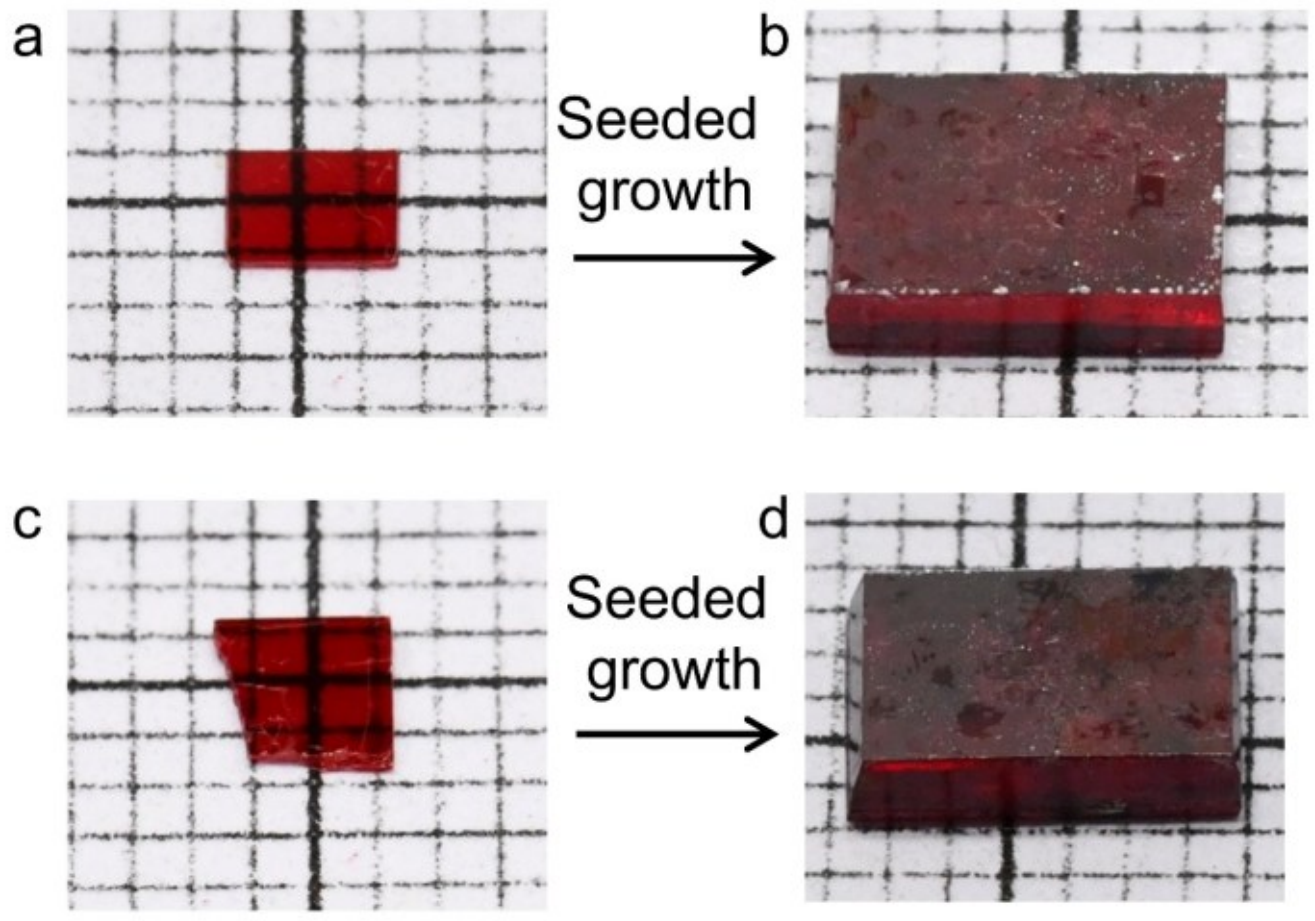

Supplementary Fig. 10. Living seeded growth of Pb-n2 crystals via the NEIT method. a, b. A well-defined cuboid Pb-n2 single crystal (a) subjected to living seeded growth within the NEIT system, resulting in a larger single crystal with well-preserved faceting and enhanced dimensions (b). **c, d.** A Pb-n2 crystal with a deliberately removed corner (c) undergoes seeded growth under NEIT conditions, recovering a regular single-crystal morphology (d). This demonstrates the persistent reactivity and inherent regenerative capability of crystal surfaces and edges under living crystallization conditions.

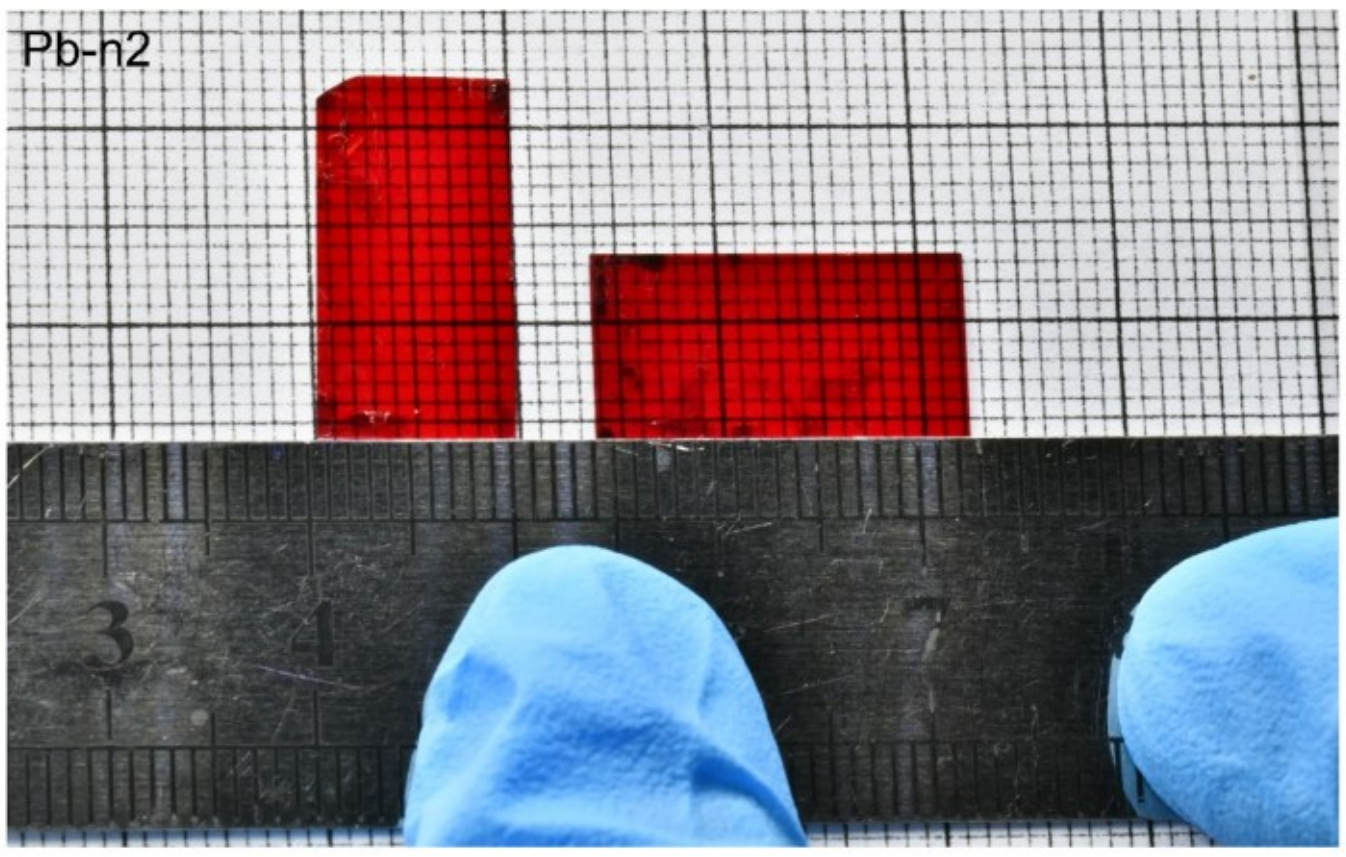

Supplementary Fig. 11. Optical images of Pb-n2 synthesized via NEIT growth. The sizes of the crystals are ~ 1.9 cm × 1 cm and 1.8 cm × 0.9 cm.

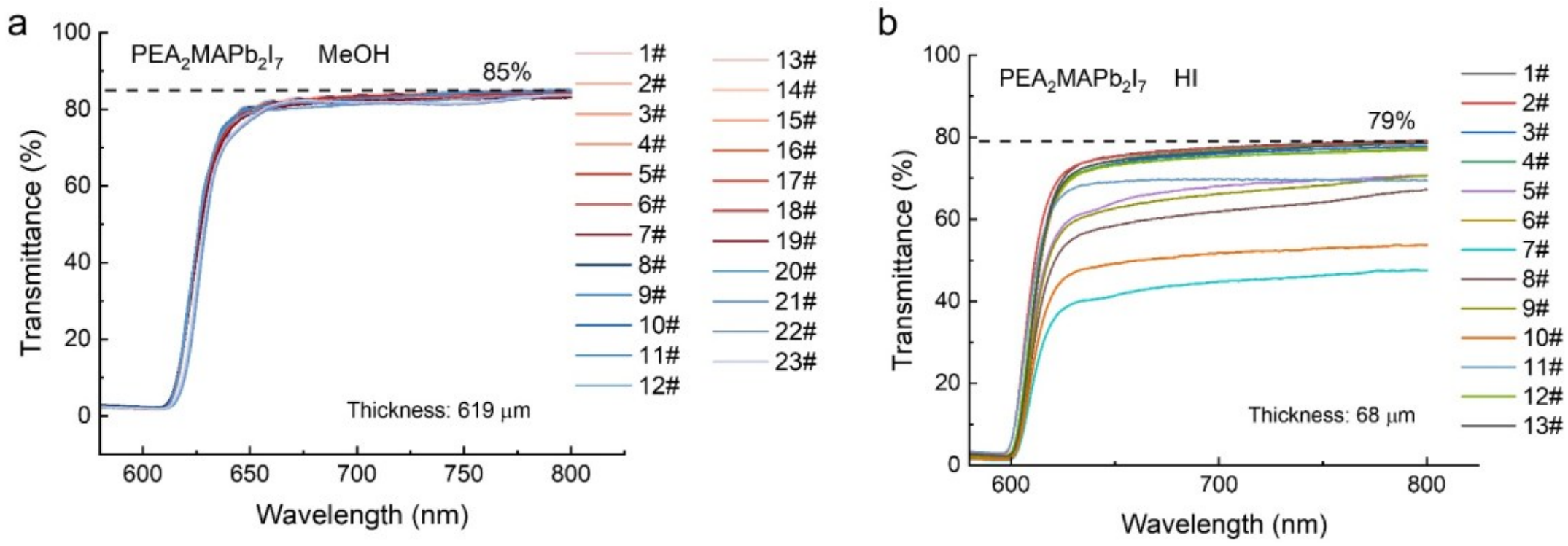

Supplementary Fig. 12. Optical transparency of NEIT- and SCS-grown Pb-n2 single crystals. a. Transmittance spectrum of a NEIT-grown Pb-n2 single crystal with a measured thickness of 619 μm. **b.** Transmittance spectrum of an SCS-grown Pb-n2 single crystal with a thickness of 68 μm.

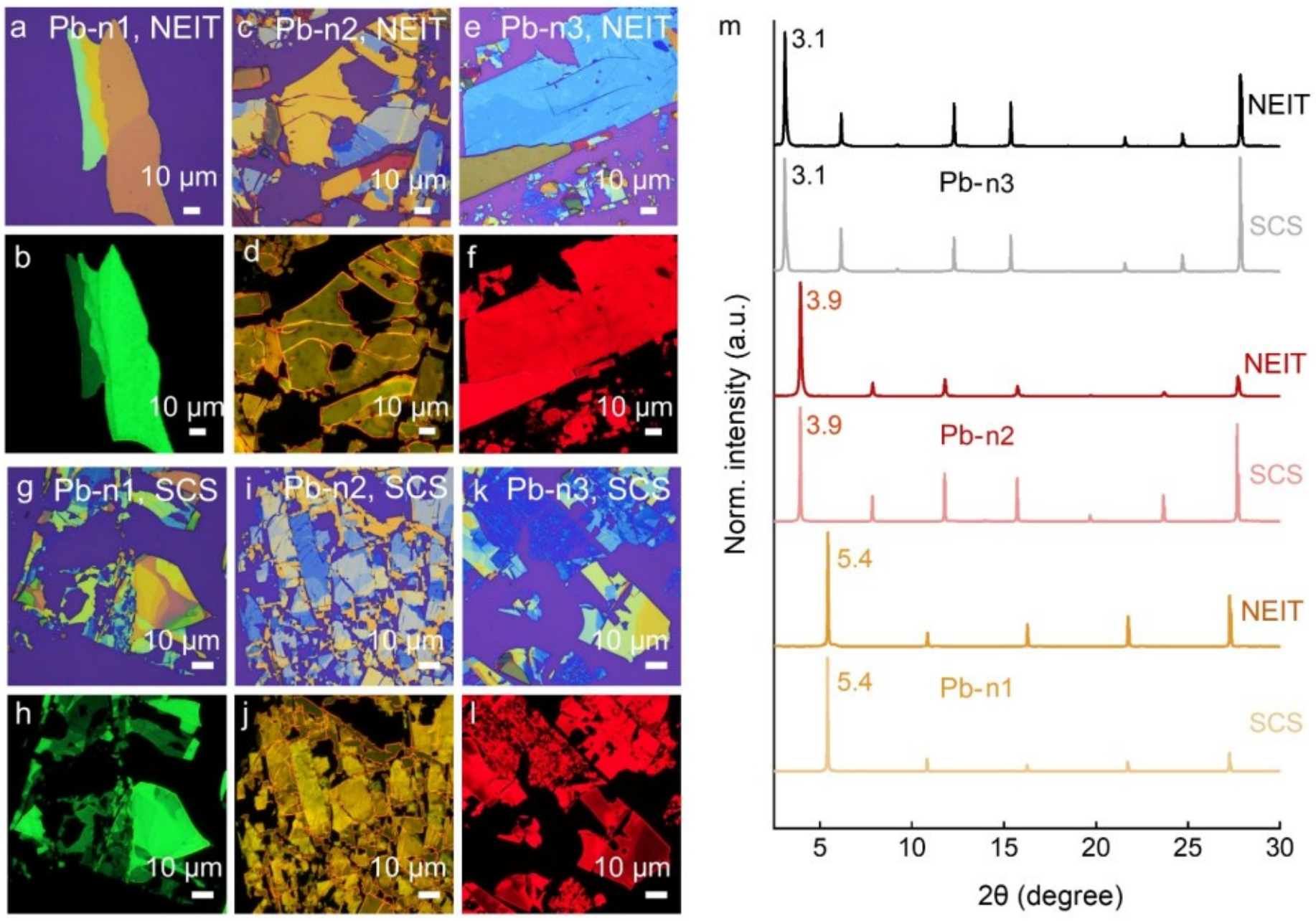

Supplementary Fig. 13. The optical images, PL images and PXRD patterns of exfoliated 2D perovskite synthesized via NEIT and SCS approaches. a-f. The optical and PL images of Pb-n1, Pb-n2 and Pb-n3 via NEIT growth. **g-l.** The optical and PL images of Pb-n1, Pb-n2 and Pb-n3 via SCS. **m.** PXRD patterns of Pb-n1, Pb-n2 and Pb-n3 synthesized via NEIT and SCS approaches.

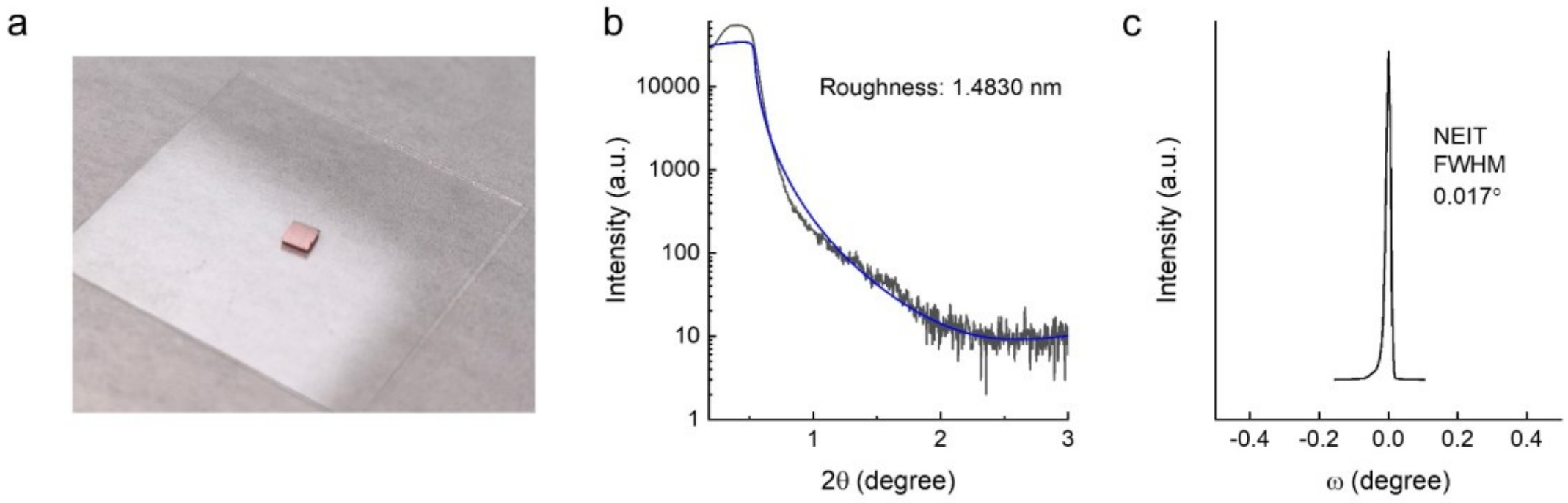

Supplementary Fig. 14. Structural characterization of NEIT-grown Pb-n2. a, Optical image of the exfoliated bulk single crystal, exposing a clear, mirror-like facet. **b,** X-ray reflectivity (XRR) profiles (gray lines) and corresponding fitting curves (blue lines) for NEIT-grown Pb-n2 sample. The extracted surface roughness value is 1.4830 nm. **c,** High-resolution X-ray rocking curve of the (001) reflection taken on the identical area, exhibiting an ultra-narrow full-width at half-maximum (FWHM) of 0.017°.

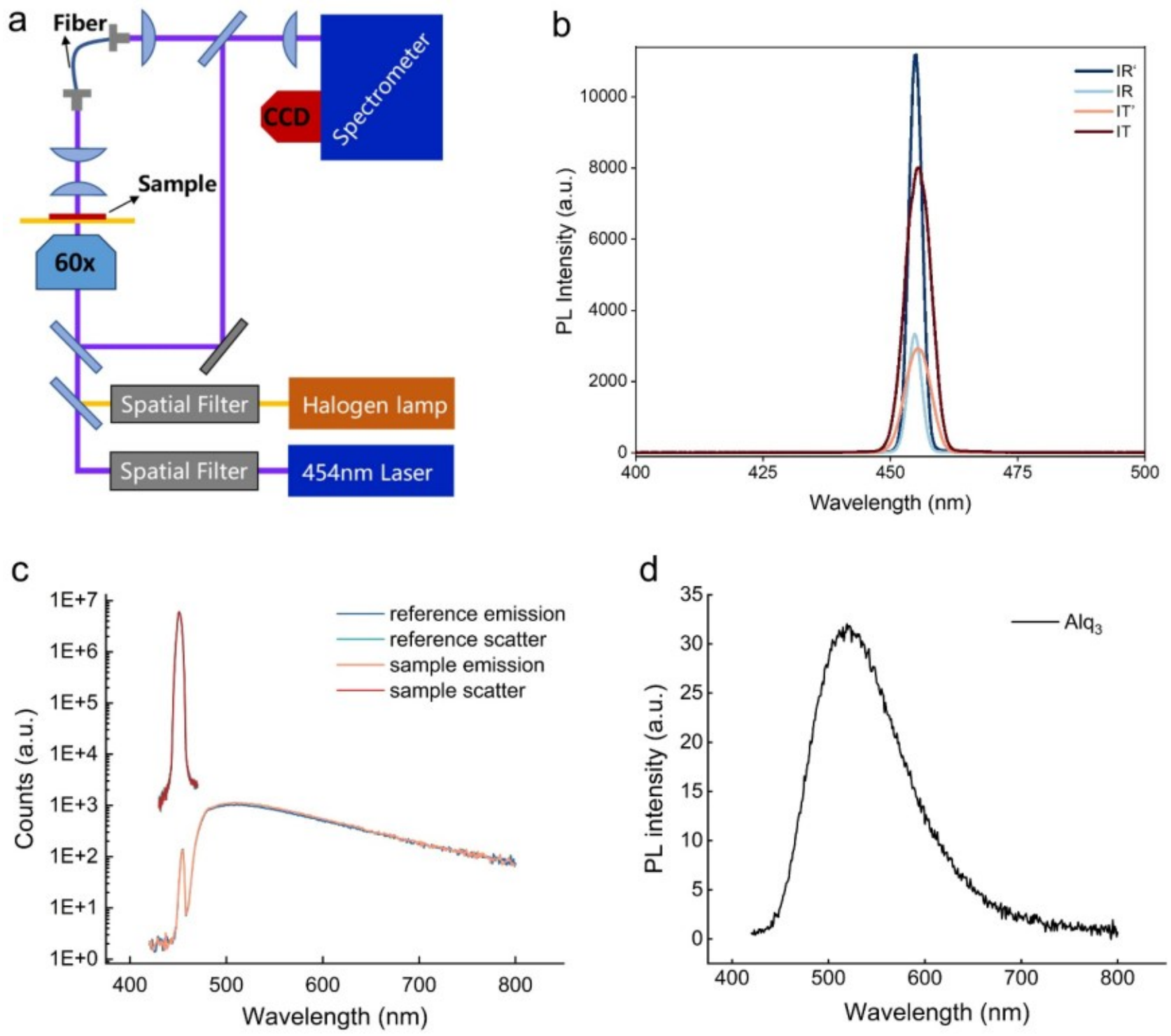

Supplementary Fig. 15. PLQY testing setup and PL testing of reference dyes (Alq_3). **a.** Schematic representation of the micro absorption spectroscopy setup. **b.** Absorption rate measurement. $A = 1 - R - T = 1 - R_{quartz} \times \frac{IR'}{IR} - \frac{IT'}{IT}$, R_{quartz}: Reflectance constant of transparent quartz substrate. IR': Laser intensity of reflected light on the sample. IR: Laser intensity of reflected light on quartz substrate. IT': Laser intensity of transmitted light on the sample. IT: Laser intensity of transmitted light on quartz substrate. **c.** Integral sphere test for absolute quantum yield of Alq_3 (PLQY: 13.23%, absorption rate: 0.26%). **d.** Fluorescence spectra of Alq_3.

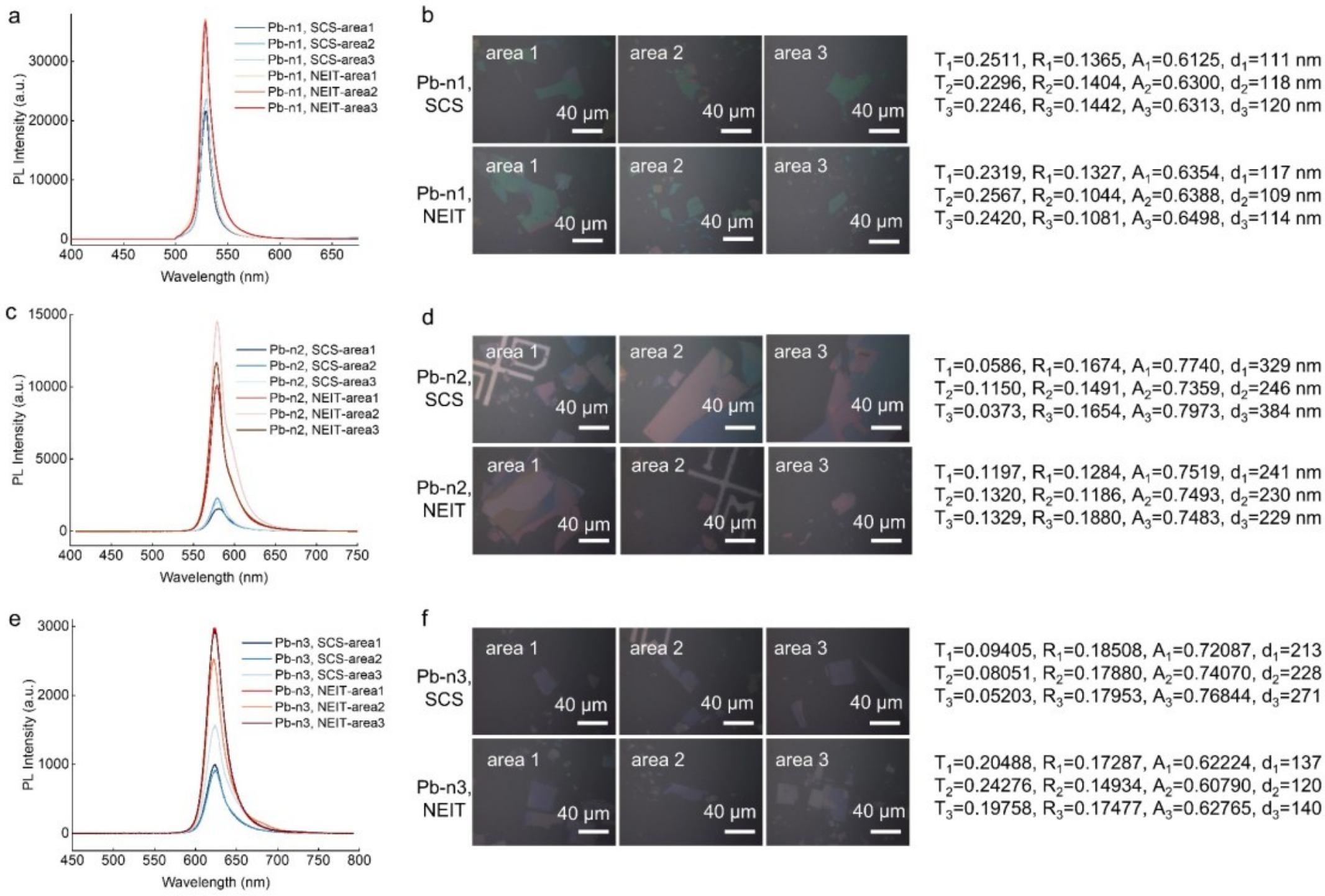

Supplementary Fig. 16. PL intensity of 2D perovskites excited under identical laser fluence (320 nW, 40 MHz, ~300 nJ/cm^2). a. PL spectra of Pb-n1 synthesized from SCS and NEIT approaches. **b.** Optical images of Pb-n1 samples synthesized from SCS and NEIT approaches for PL characterization. **c.** PL spectra of Pb-n2 synthesized from SCS and NEIT approaches. **d.** Optical images of Pb-n2 samples synthesized from SCS and NEIT approaches for PL characterization. **e.** PL spectra of Pb-n3 synthesized from SCS and NEIT approaches. **f.** Optical images of Pb-n3 samples synthesized from SCS and NEIT approaches for PL characterization. For each characterized region (area 1-3) in **b, d,** and **f**, the corresponding measured transmittance (T), reflectance (R), calculated absorptance (A) and calculated thickness (d) values are listed on the right, determined according to the relationship A=1-R-T. Substitute the transmittance at 454 nm into the formula established in Supplementary Figs. 17-19 to obtain the thickness.

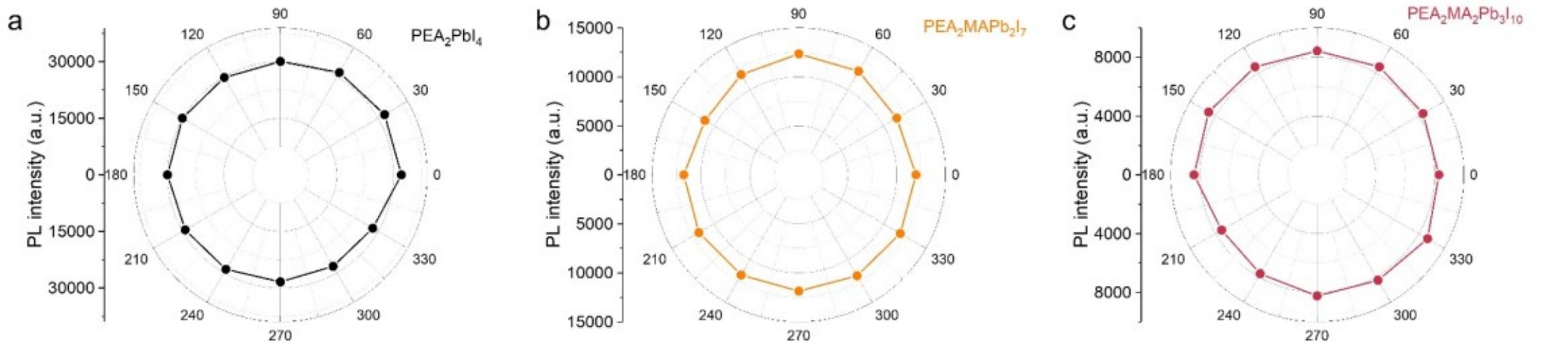

Supplementary Fig. 17. In-plane PL polarization of Pb-n1 (a), Pb-n2 (b) and Pb-n3 (c).

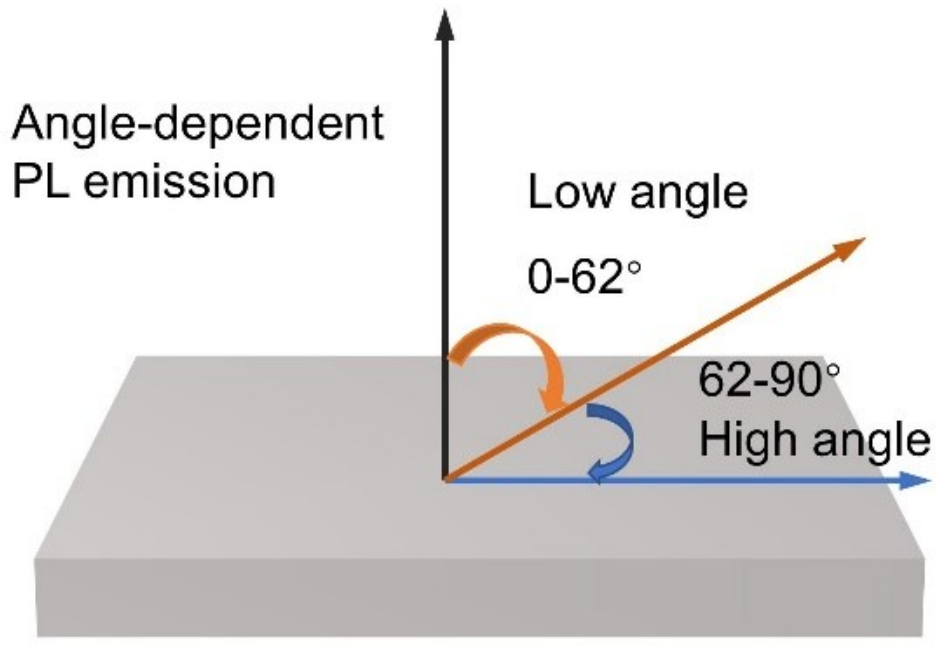

Supplementary Fig. 18. Schematic illustration of the angle-dependent PL measurements. The angles are defined relative to the surface normal, comprising a low-angle region (0° to 62°) and a complementary high-angle region (62° to 90°).

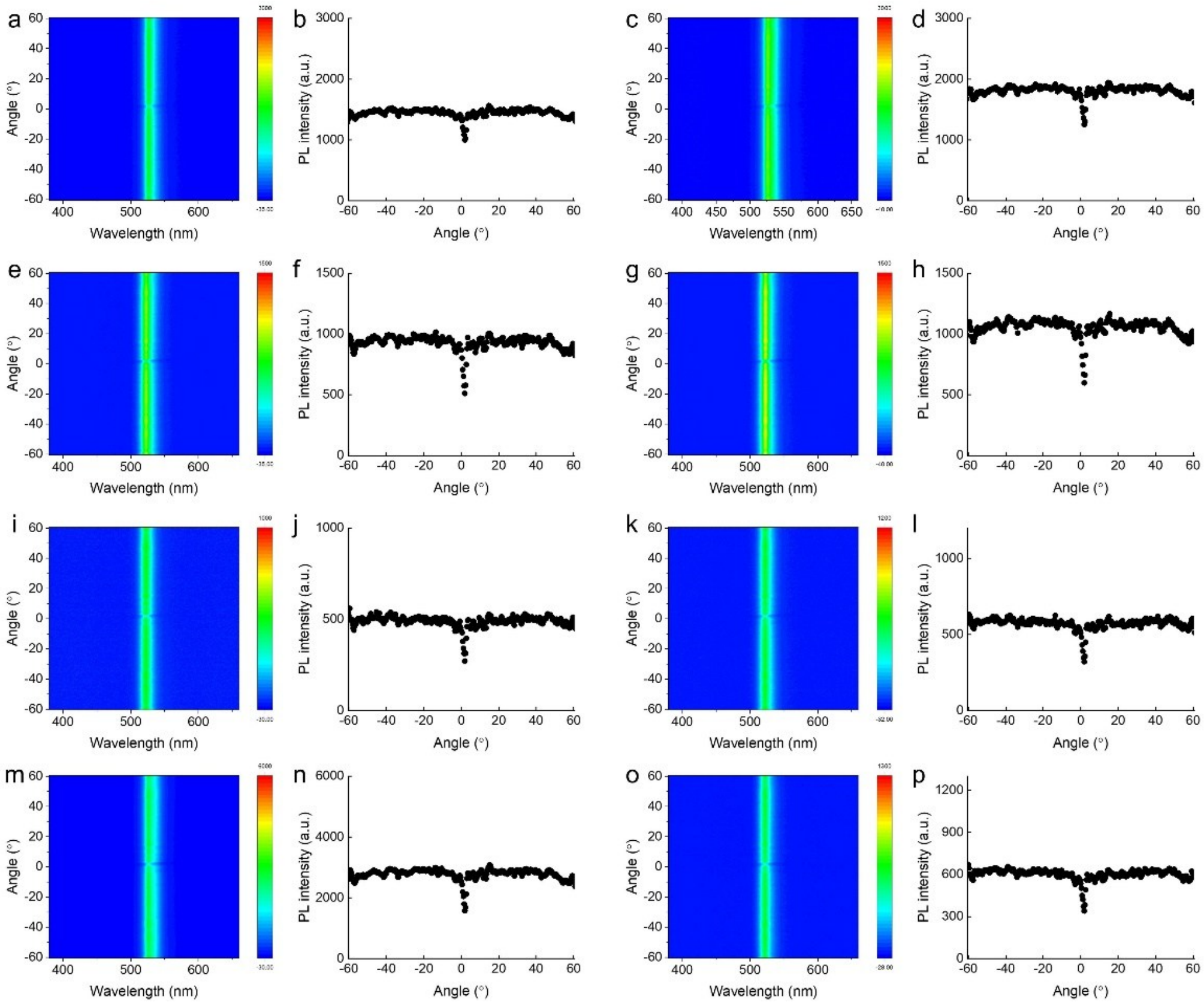

Supplementary Fig. 19. Low-angle angle-dependent PL of Pb-n1 single crystals. a, c, e, g, i, k, m, o, 2D pseudo-color maps of micro-area angle-resolved PL spectra collected from eight randomly selected regions on the Pb-n1 single crystal. **b, d, f, h, j, l, n, p,** Corresponding extracted peak PL intensities recorded at different emission angles. The sharp decrease in PL intensity observed near 0° is an instrumental artifact originating from the optical measurement setup.

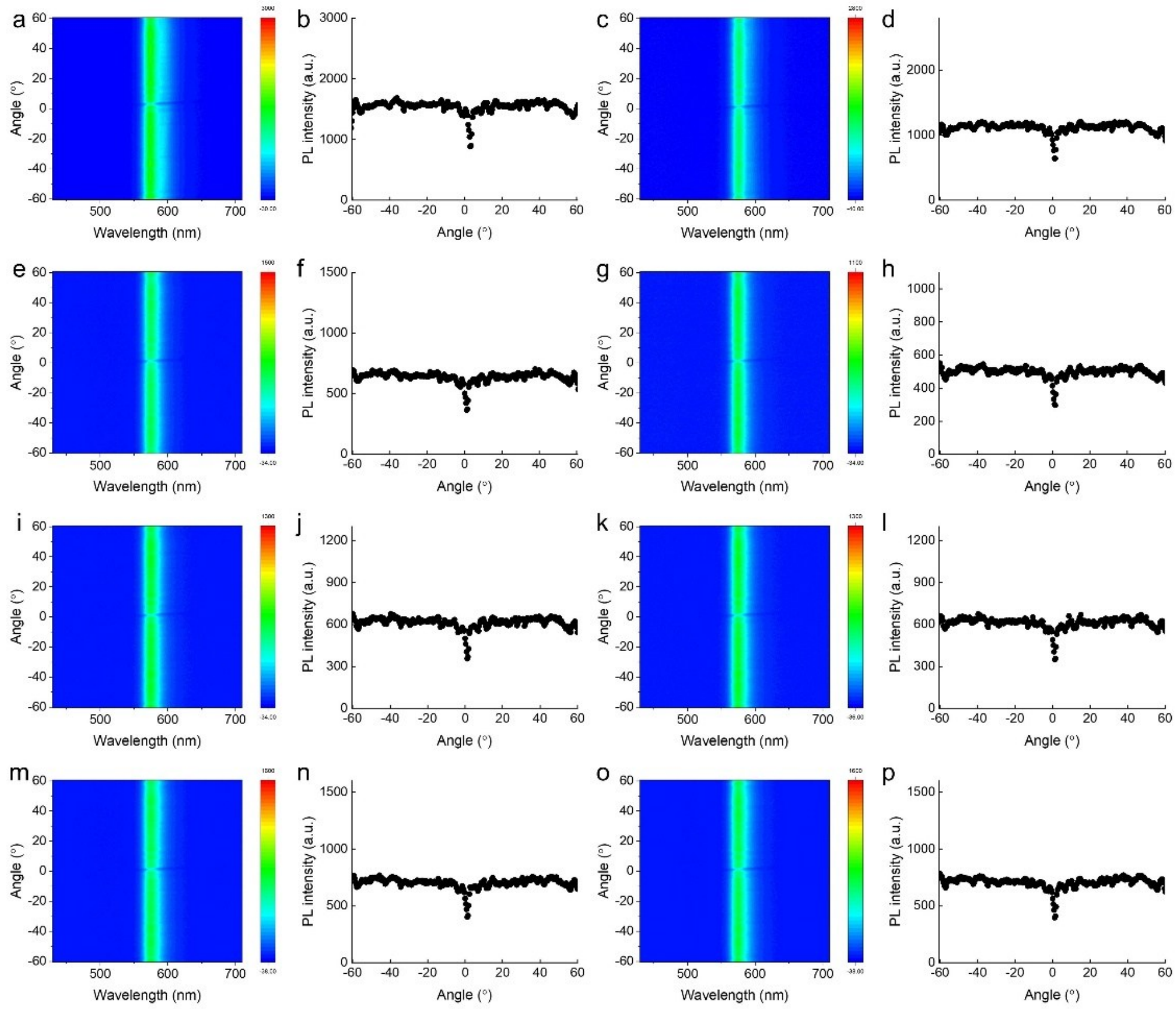

Supplementary Fig. 20. Low-angle angle-dependent PL of Pb-n2 single crystals. a, c, e, g, i, k, m, o, 2D pseudo-color maps of micro-area angle-resolved PL spectra collected from eight randomly selected regions on the Pb-n2 single crystal. **b, d, f, h, j, l, n, p,** Corresponding extracted peak PL intensities recorded at different emission angles. The sharp decrease in PL intensity observed near 0° is an instrumental artifact originating from the optical measurement setup.

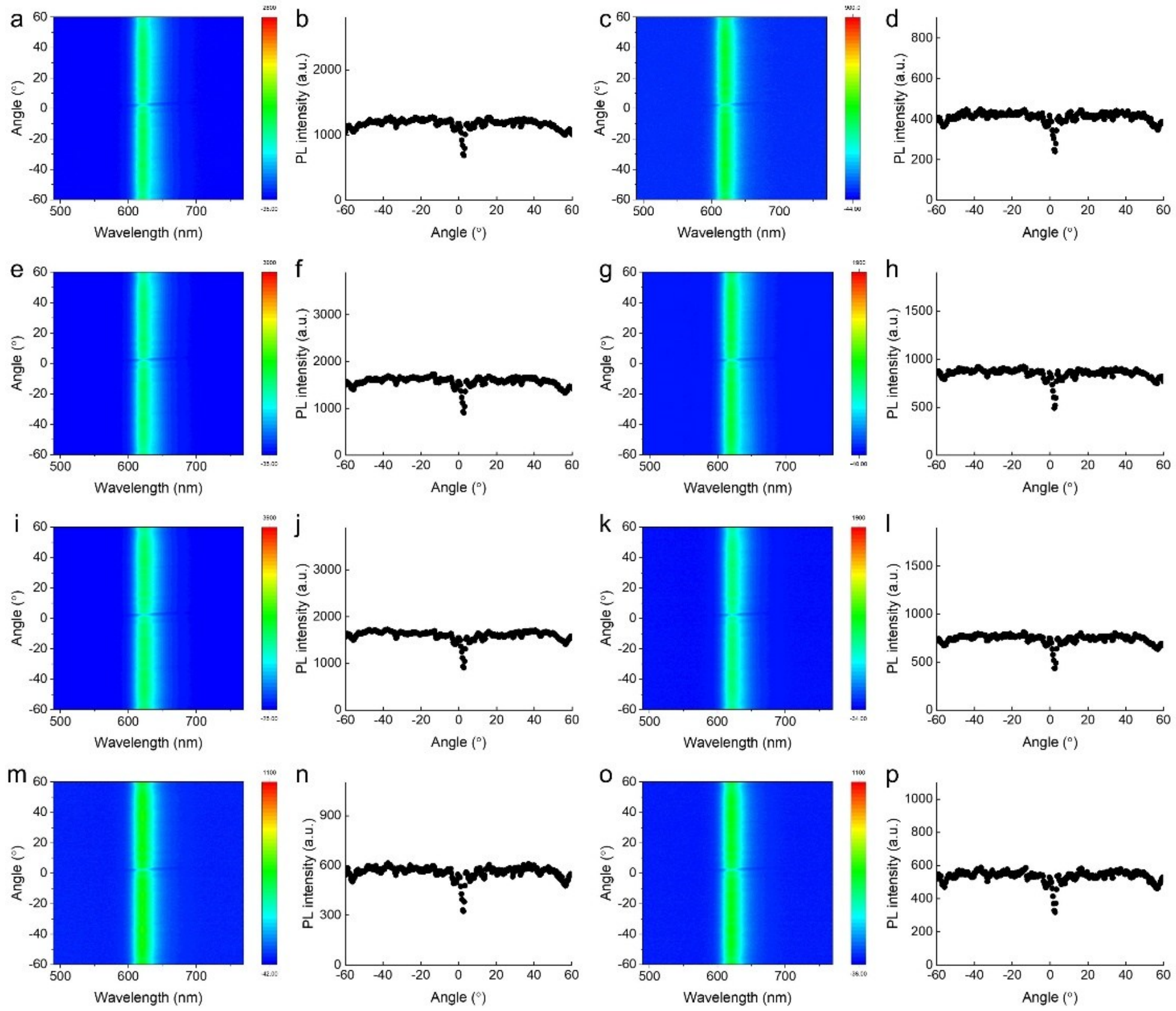

Supplementary Fig. 21. Low-angle angle-dependent PL of Pb-n3 single crystals. a, c, e, g, i, k, m, o, 2D pseudo-color maps of micro-area angle-resolved PL spectra collected from eight randomly selected regions on the Pb-n3 single crystal. **b, d, f, h, j, l, n, p,** Corresponding extracted peak PL intensities recorded at different emission angles. The sharp decrease in PL intensity observed near 0° is an instrumental artifact originating from the optical measurement setup.

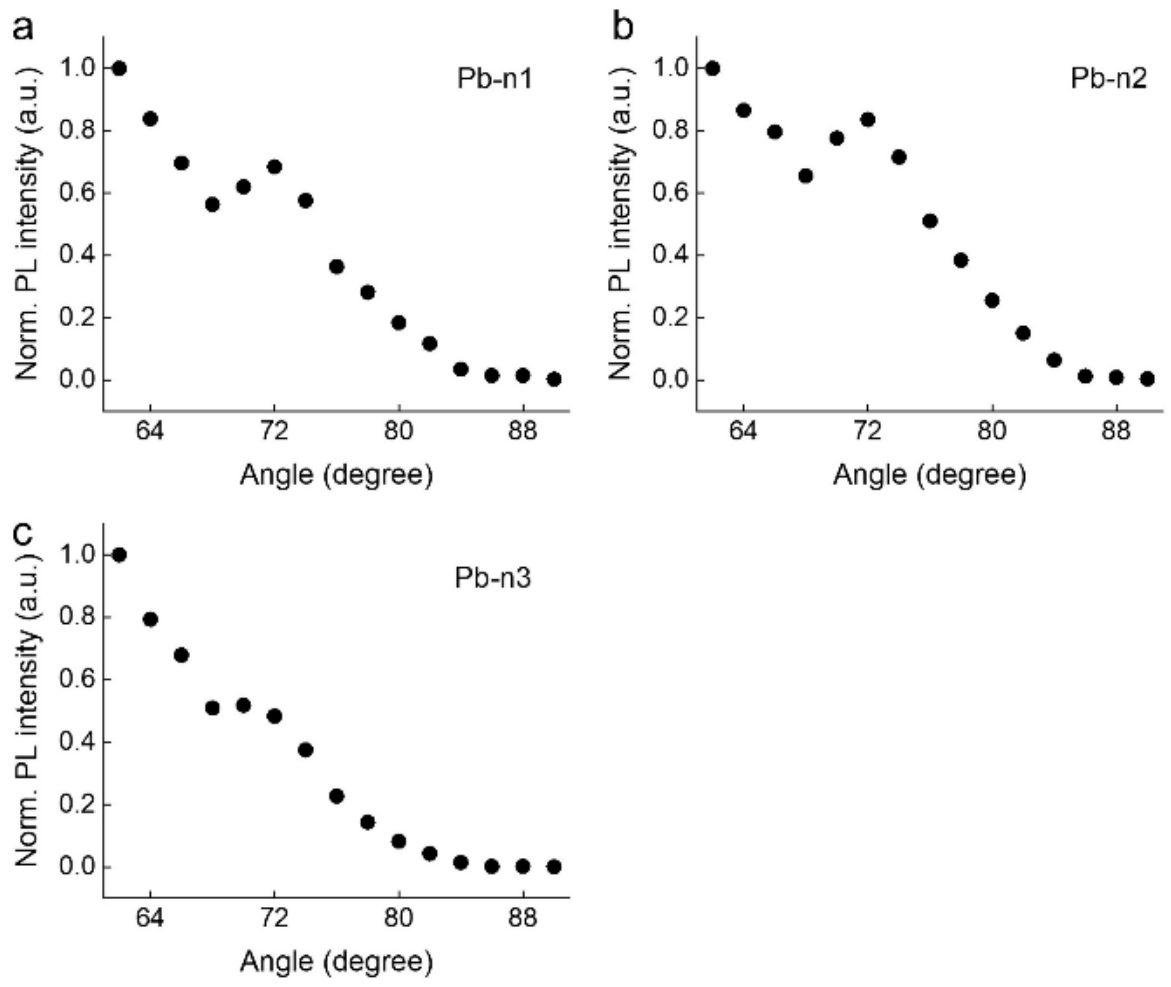

Supplementary Fig. 22. High-angle angle-dependent PL profiles of 2D perovskites. Normalized peak PL intensities recorded at high emission angles (ranging from 62° to 90°) for Pb-n1 (a), Pb-n2 (b), and Pb-n3 (c). Data were collected using a molecular orientation spectrometer on spin-coated thin films, which were prepared by dissolving the respective single crystals. In contrast to the out-of-plane isotropic emission, the pronounced attenuation of PL intensity towards 90° clearly demonstrates the in-plane optical anisotropy dictated by the 2D layered structure. These high-angle emission profiles were utilized to comprehensively recalibrate the PLQY values.

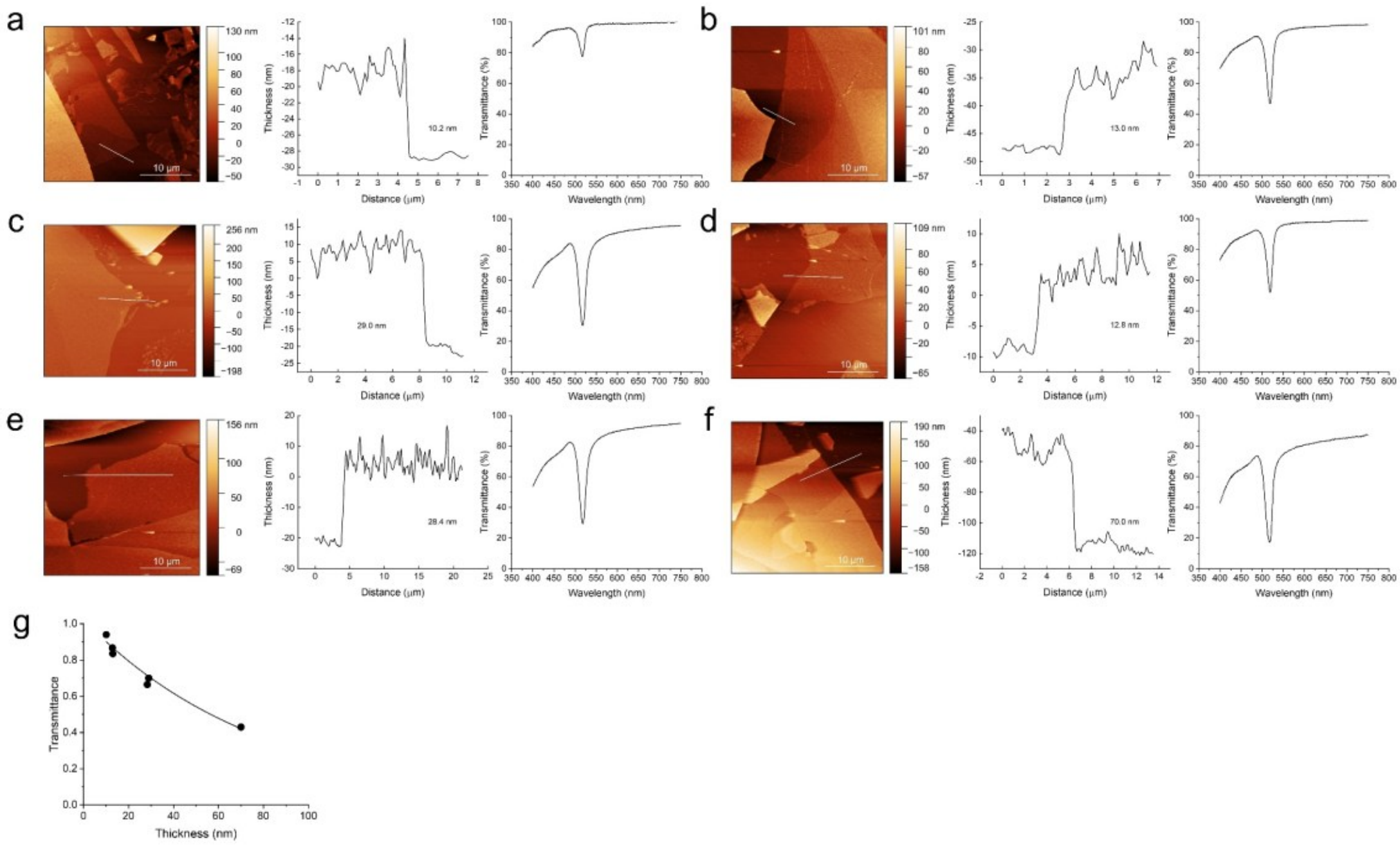

Supplementary Fig. 23. Establishment of the thickness-absorbance correlation for exfoliated Pb-n1 flakes. a-f. Atomic force microscopy (AFM) images (left), corresponding height profiles (middle), and transmittance (right) of mechanically exfoliated Pb-n1 thin flakes with varying thicknesses. The measured thicknesses range from ~10.2 nm to ~70.0 nm, with the corresponding transmittance values extracted at 454 nm. **g**, The extracted correlation between the thickness and transmittance (454 nm).

To quantify the thickness of the flakes utilized in our PLQY measurements, we established thickness-transmittance calibration. We performed AFM and transmittance measurements on a series of exfoliated Pb, n=1-3 flakes with varying thicknesses. In accordance with the Beer-Lambert law, the measured transmittance exhibits a correlation with the AFM-determined thickness. Utilizing this established relationship, the thickness of exfoliated flake can be fitted from its transmittance signal under 454 nm.

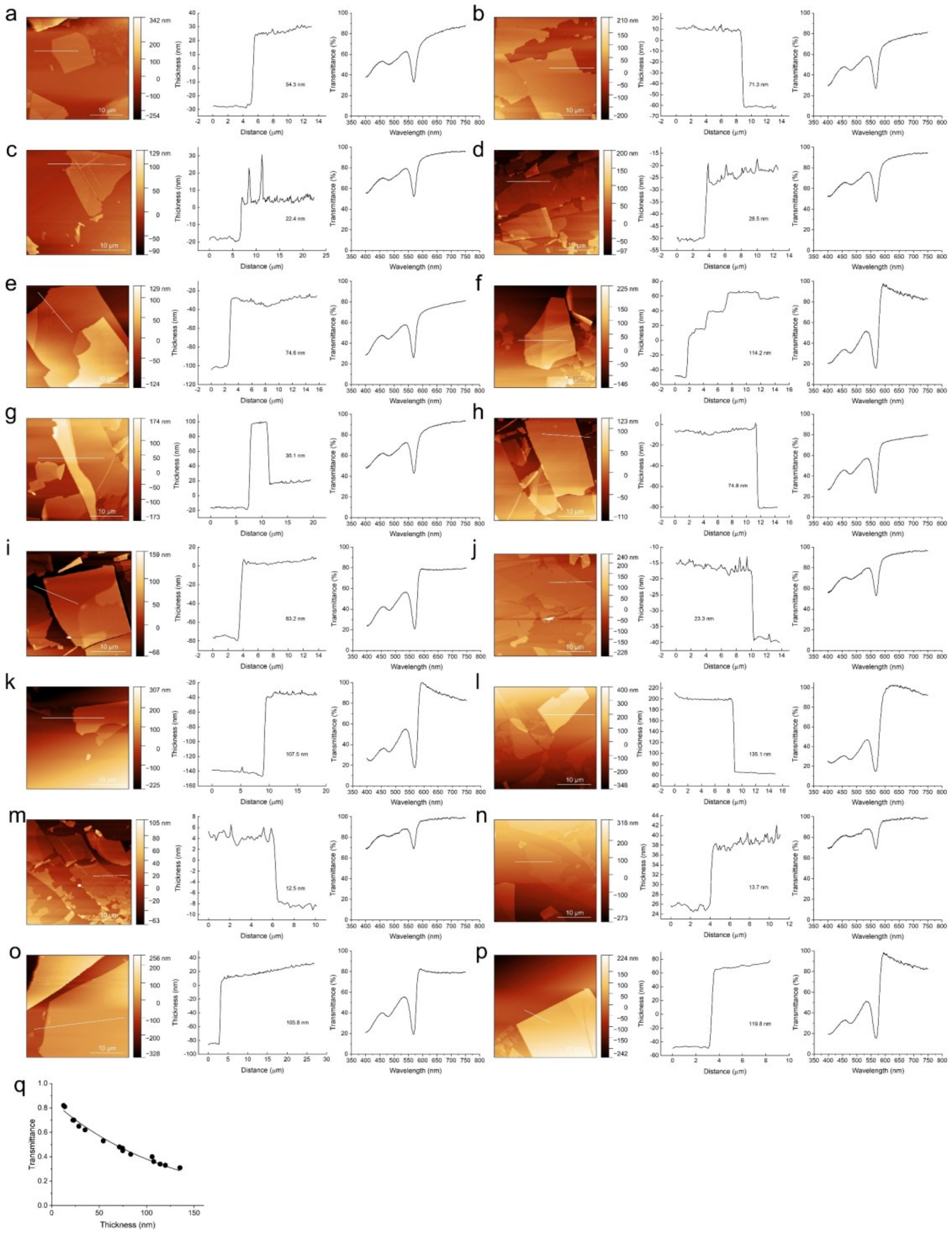

Supplementary Fig. 24. Establishment of the thickness-absorbance correlation for exfoliated Pb-n2 flakes. a-p. Atomic force microscopy (AFM) images (left), corresponding height profiles (middle), and transmittance (right) of mechanically exfoliated Pb-n2 thin flakes with varying thicknesses. The measured thicknesses range from ~12.5 nm to ~135.1 nm, with the corresponding transmittance values extracted at 454 nm. q, The extracted correlation between the thickness and transmittance (454 nm).

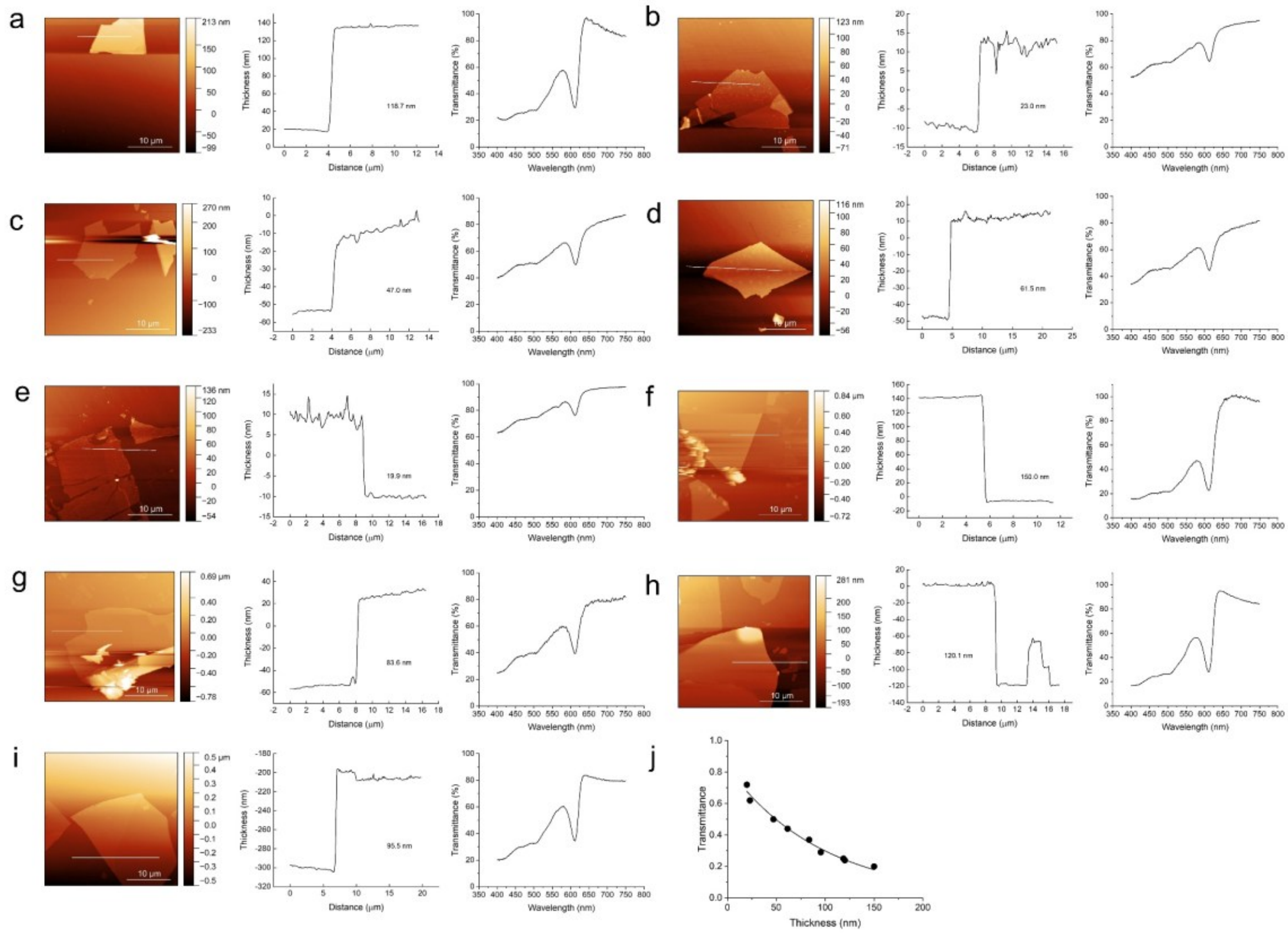

Supplementary Fig. 25. Establishment of the thickness-absorbance correlation for exfoliated Pb-n3 flakes. a-i. Atomic force microscopy (AFM) images (left), corresponding height profiles (middle), and transmittance (right) of mechanically exfoliated Pb-n3 thin flakes with varying thicknesses. The measured thicknesses range from ~19.9 nm to ~150.0 nm, with the corresponding transmittance values extracted at 454 nm. **j**, The extracted correlation between the thickness and transmittance (454 nm).

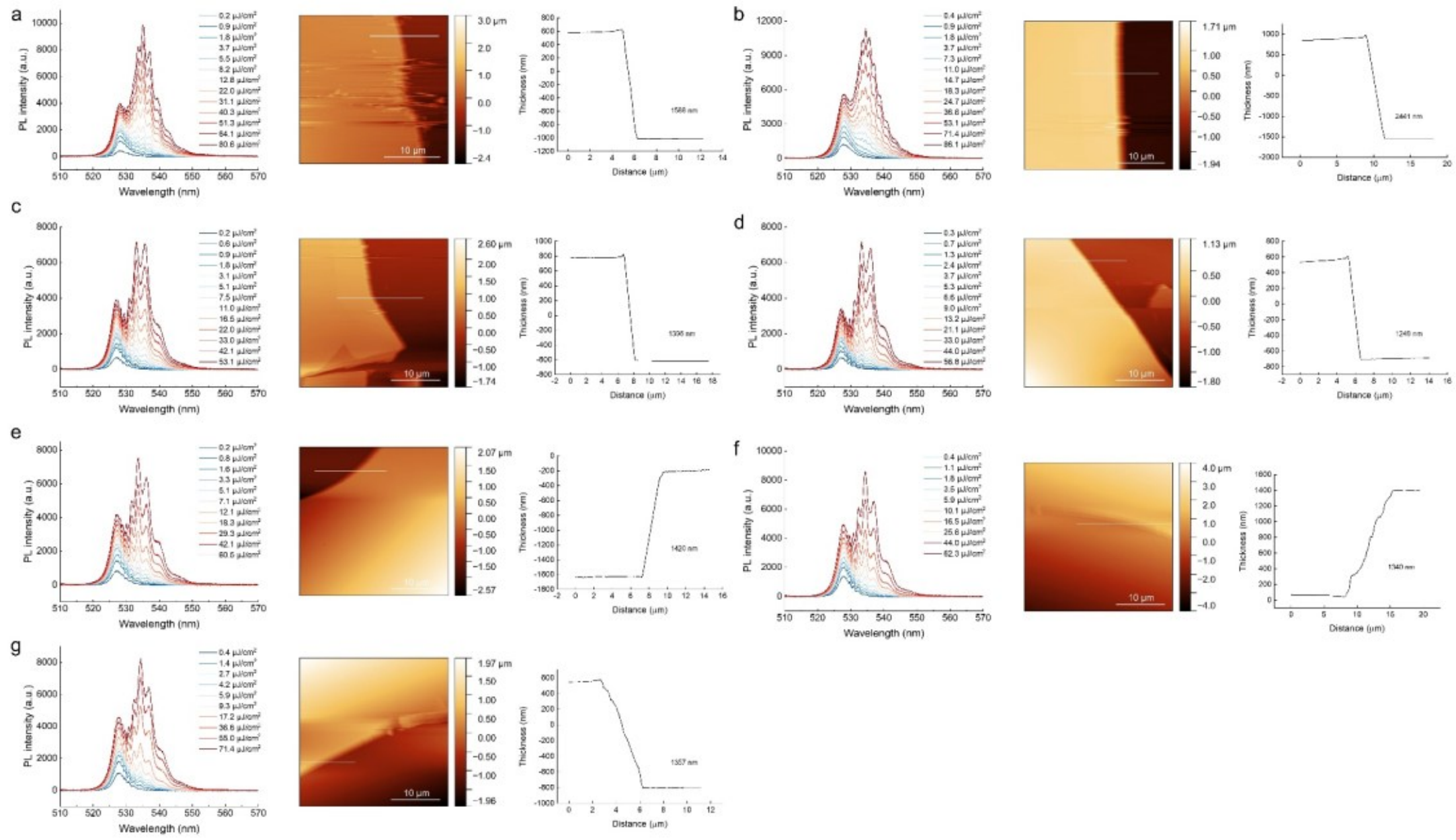

Supplementary Fig. 26. Lasing spectra and corresponding thickness of Pb-n1 exfoliated flakes synthesized from NEIT growth，including a. Sample 1# with the pump fluence increasing from 0.2 to 80.6 μJ/cm^2, the lasing threshold is ~12.8 μJ/cm^2 and the corresponding thickness is ~1588 nm. **b.** Sample 2# with the pump fluence increasing from 0.4 to 86.1 μJ/cm^2, the lasing threshold is ~14.7 μJ/cm^2 and the corresponding thickness is ~2441 nm. **c.** Sample 3# with the pump fluence increasing from 0.2 to 53.1 μJ/cm^2, the lasing threshold is ~ 11.0 μJ/cm^2 and the corresponding thickness is ~1396 nm. **d.** Sample 4# with the pump fluence increasing from 0.3 to 56.8 μJ/cm^2, the lasing threshold is ~9.0 μJ/cm^2 and the corresponding thickness is ~1249 nm. **e.** Sample 5# with the pump fluence increasing from 0.2 to 60.5 μJ/cm^2, the lasing threshold is ~ 12.1 μJ/cm^2 and the corresponding thickness is ~1420 nm. **f.** Sample 6# with the pump fluence increasing from 0.4 to 62.3 μJ/cm^2, the lasing threshold is ~16.5 μJ/cm^2 and the corresponding thickness is ~1340 nm. **g.** Sample 7# with the pump fluence increasing from 0.4 to 71.4 μJ/cm^2, the lasing threshold at ~17.2 μJ/cm^2 and the corresponding thickness is ~1357 nm.

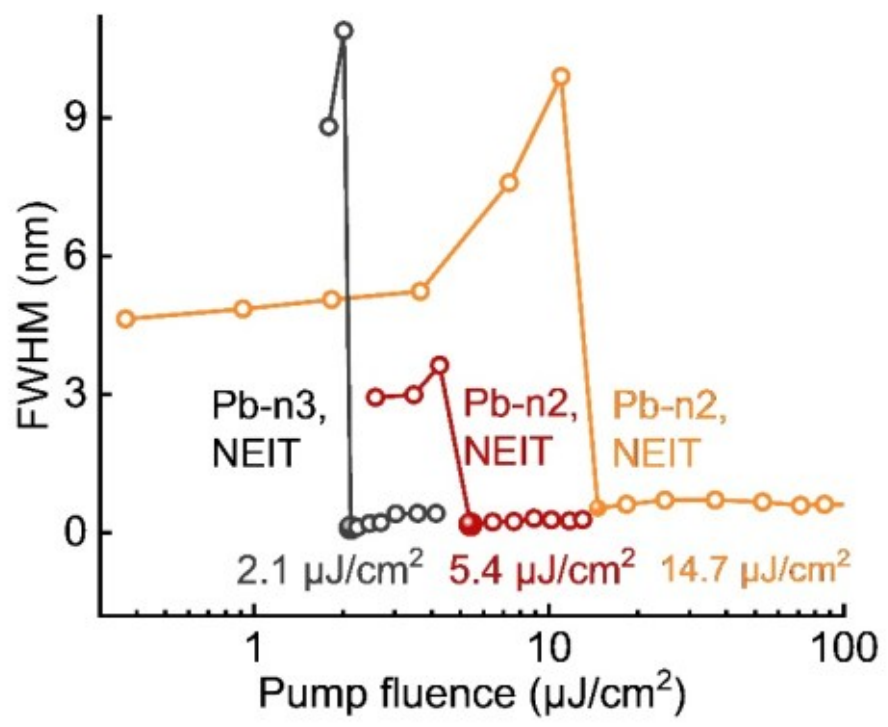

Supplementary Fig. 27. Pump fluence dependent FWHM (80 K) of Pb-n1, n2 and n3 synthesized from NEIT growth.

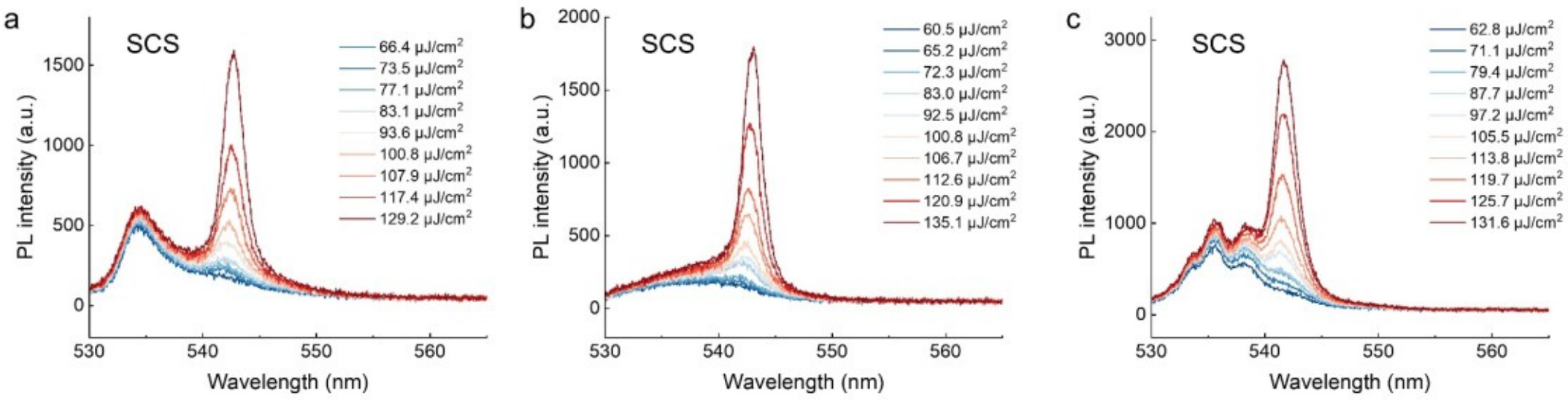

Supplementary Fig. 28. PL spectra of multiple Pb-n1 exfoliated flakes synthesized from SCS growth, including a. Sample 1# with the pump fluence increasing from 66.4 to 129.2 μJ/cm^2, the lasing threshold is ~93.6 μJ/cm^2. **b.** Sample 2# with the pump fluence increasing from 60.5 to 135.1 μJ/cm^2, the lasing threshold is ~100.8 μJ/cm^2. **c.** Sample 3# with the pump fluence increasing from 62.8 to 131.6 μJ/cm^2, the lasing threshold is ~97.2 μJ/cm^2.

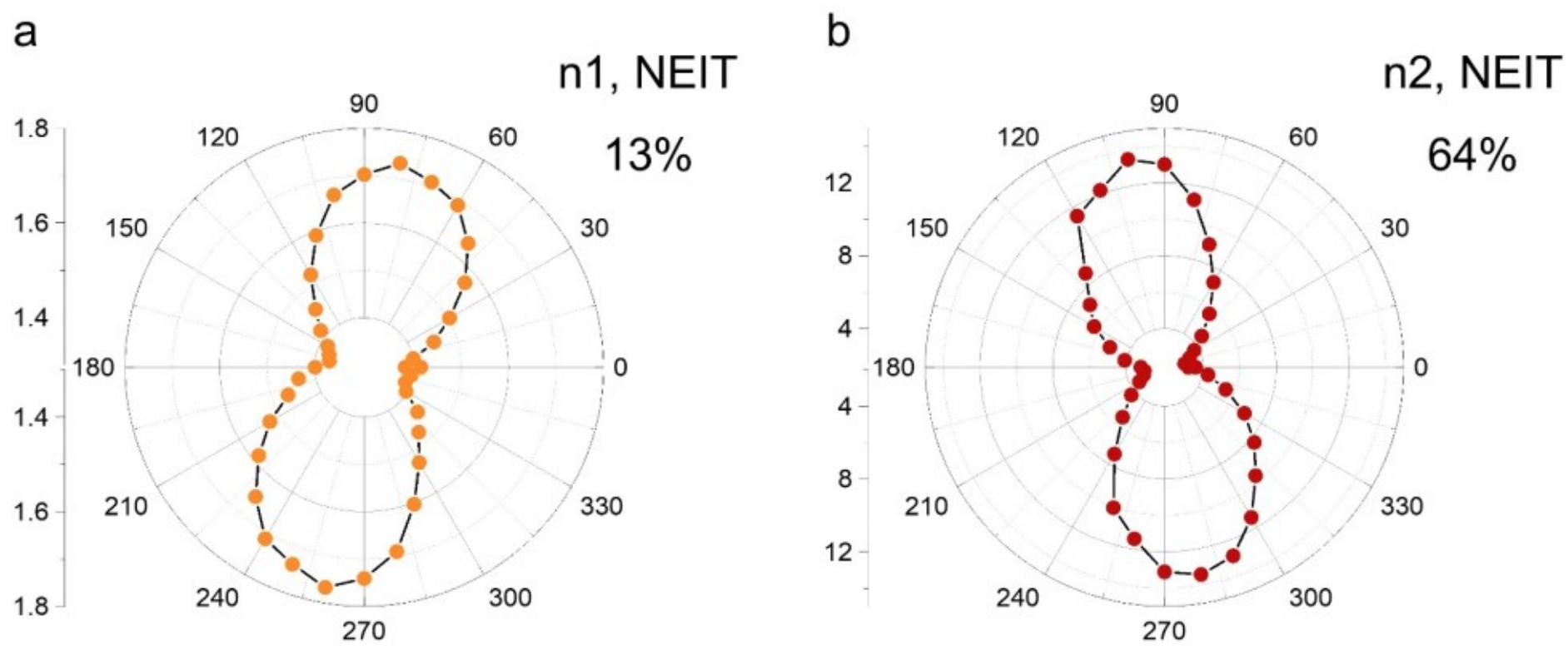

Supplementary Fig. 29. Polarization-resolved lasing spectra of 2D perovskites synthesized from NEIT growth at 80 K. a. Polarization-resolved lasing spectra of Pb-n1 from NEIT growth at 80 K. **b.** Polarization-resolved lasing spectra of Pb-n2 from NEIT growth at 80 K.

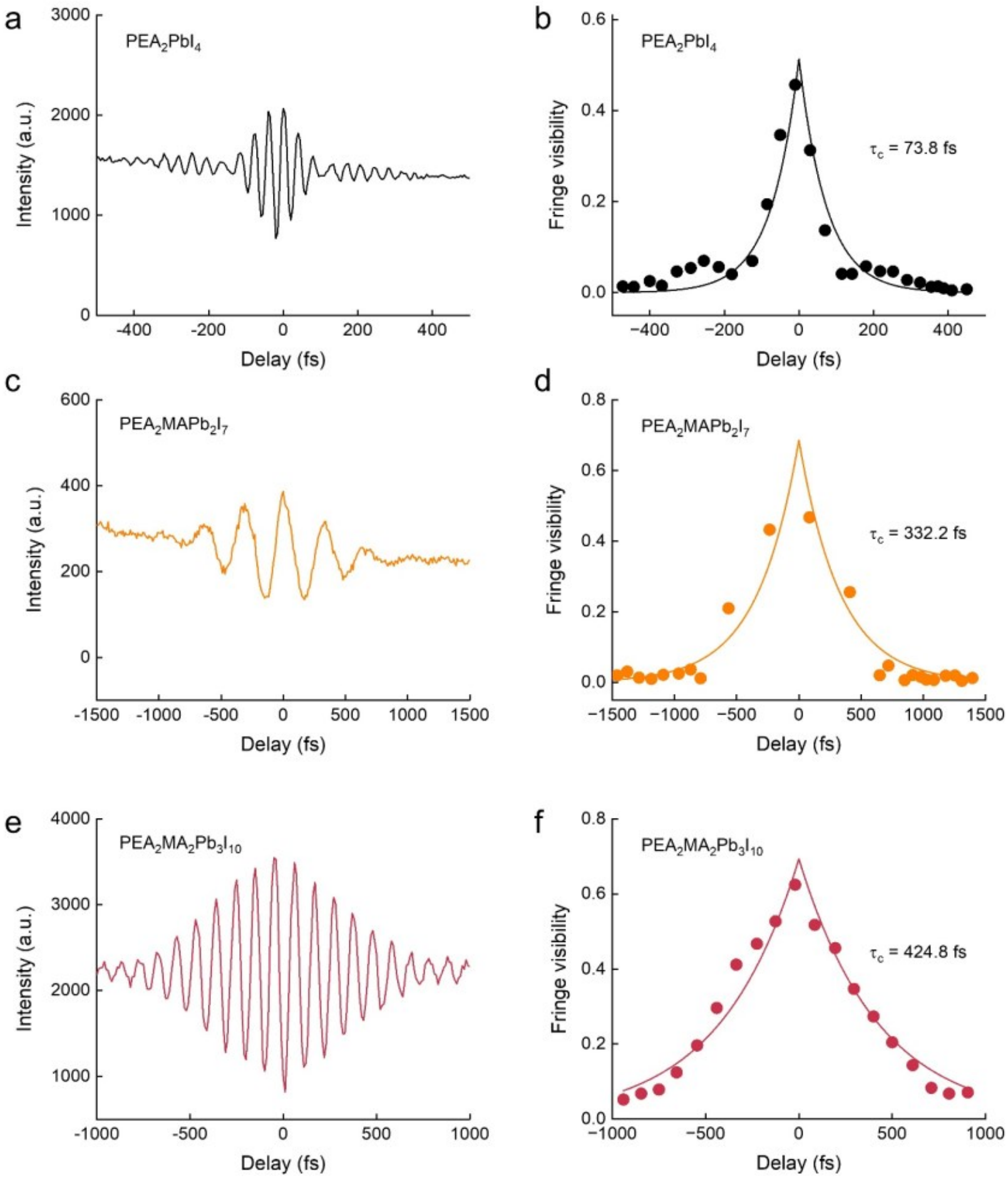

Supplementary Fig. 30. Coherence time measurements of the exfoliated Pb-n1, Pb-n2, and Pb-n3 flakes under lasing conditions. a, c, e, Interference fringe patterns (interferograms) of the emission from NEIT-grown Pb-n1 (a), Pb-n2 (c), and Pb-n3 (e) flakes measured by a Michelson interferometer above the lasing threshold at 80 K. The distinct oscillatory patterns indicate the strong phase correlation of the emitted photon wavepackets. **b, d, f,** The corresponding fringe visibility as a function of the time delay for Pb-n1 (b), Pb-n2 (d), and Pb-n3 (f). The solid lines represent the fitting curves used to extract the temporal coherence times (τ_c). The coherence times are quantified as 73.8 fs, 332.2 fs, and 424.8 fs for n=1, 2, and 3 perovskites, respectively. These extended coherence times and clear interference profiles provide unambiguous evidence of the temporal coherence of the optical emission, proving the true lasing.

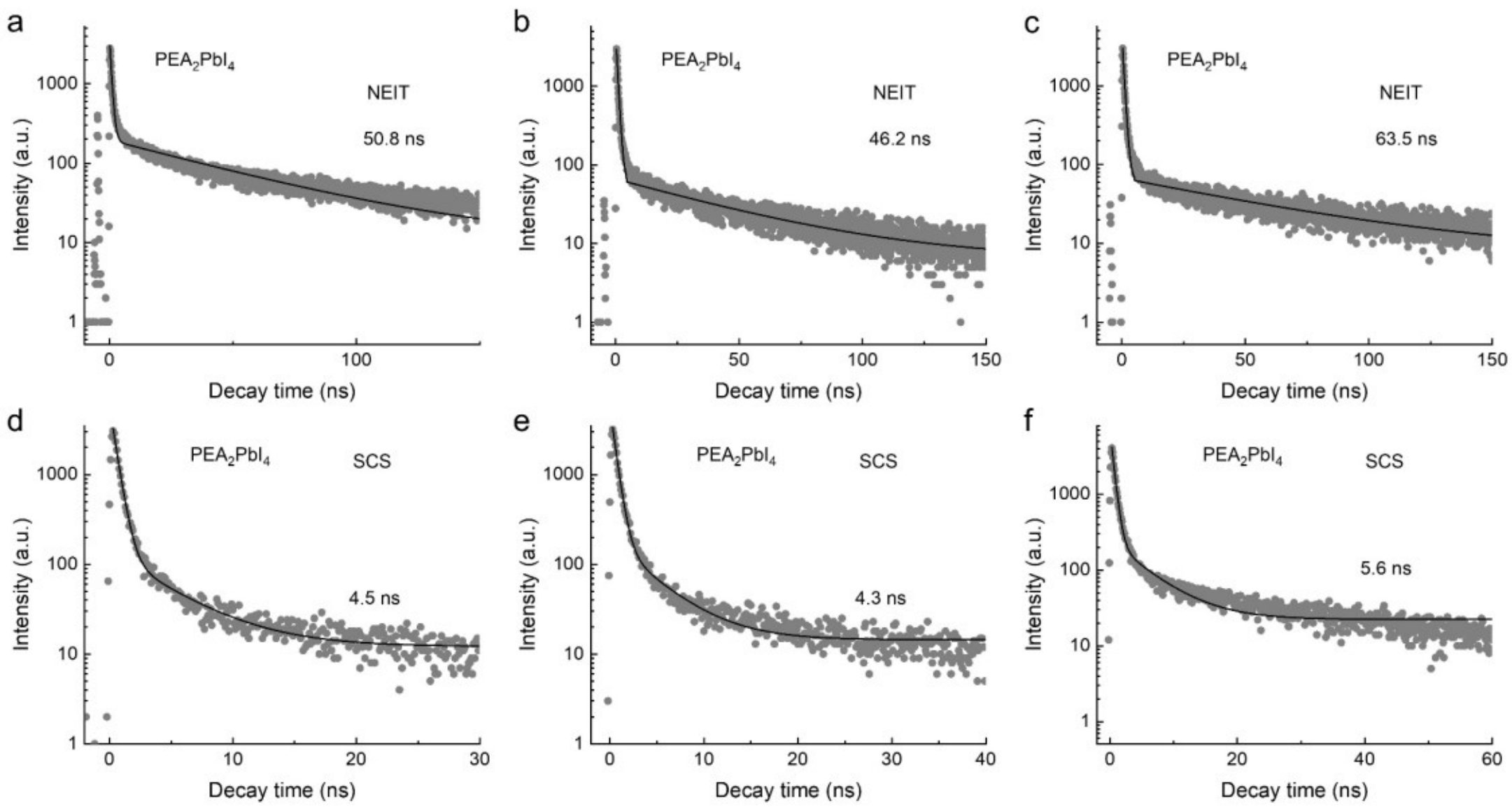

Supplementary Fig. 31. Reproducibility of carrier lifetime measurements for Pb-n1 single crystals. a-c. Repeated TRPL decay spectra and bi-exponential fitting curves of Pb-n1 flakes exfoliated from NEIT-grown single crystals, yielding consistently prolonged τ_2 lifetimes of 50.8 ns, 46.2 ns, and 63.5 ns. **d-f.** Repeated TRPL decay spectra of Pb-n1 flakes from conventional SCS-grown crystals under identical testing conditions, showing short lifetimes of 4.5 ns, 4.3 ns, and 5.6 ns.

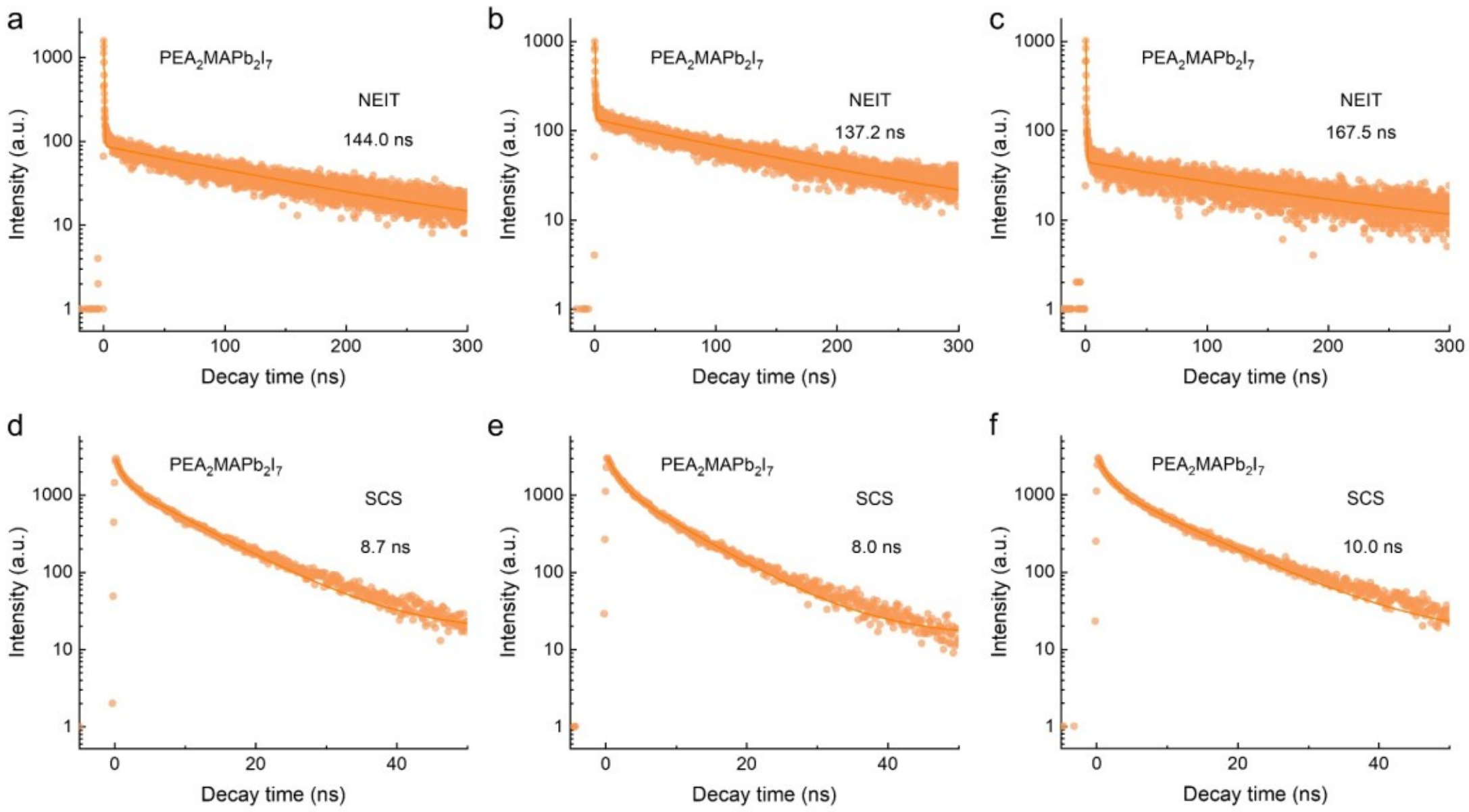

Supplementary Fig. 32. Reproducibility of carrier lifetime measurements for Pb-n2 single crystals. a-c. Repeated TRPL decay spectra and fitting curves of NEIT-grown Pb-n2 flakes, demonstrating long τ_2 lifetimes of 144.0 ns, 137.2 ns, and 167.5 ns. **d-f.** Repeated TRPL decay spectra of SCS-grown Pb-n2 flakes, showing consistently short lifetimes of 8.7 ns, 8.0 ns, and 10.0 ns.

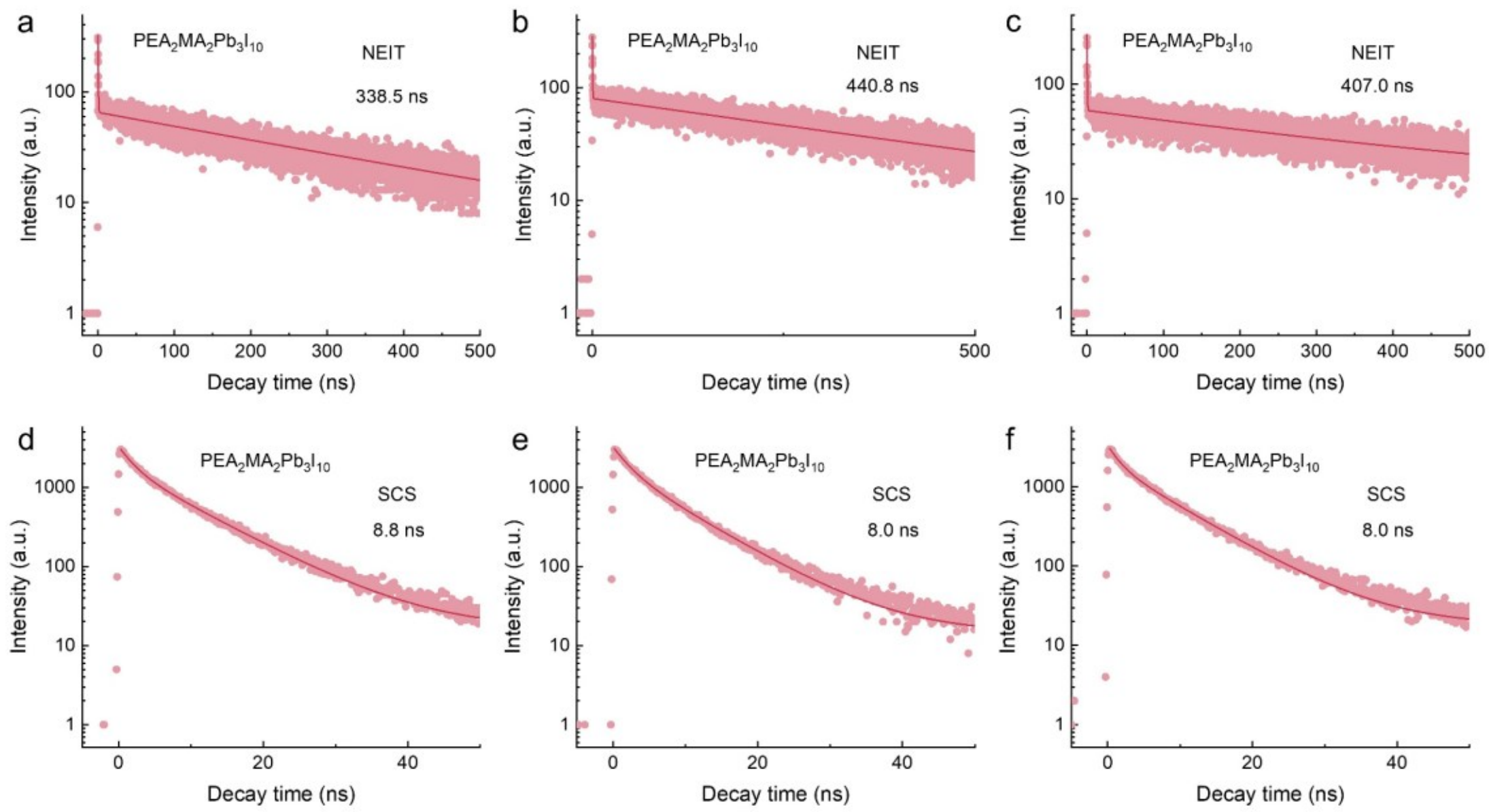

Supplementary Fig. 33. Reproducibility of carrier lifetime measurements for Pb-n3 single crystals. a-c. Repeated TRPL decay spectra and fitting curves of NEIT-grown Pb-n3 flakes, demonstrating long τ_2 lifetimes of 338.5 ns, 440.8 ns, and 407.0 ns. **d-f.** Repeated TRPL decay spectra of SCS-grown Pb-n3 flakes, showing short lifetimes of 8.8 ns, 8.0 ns, and 8.0 ns.

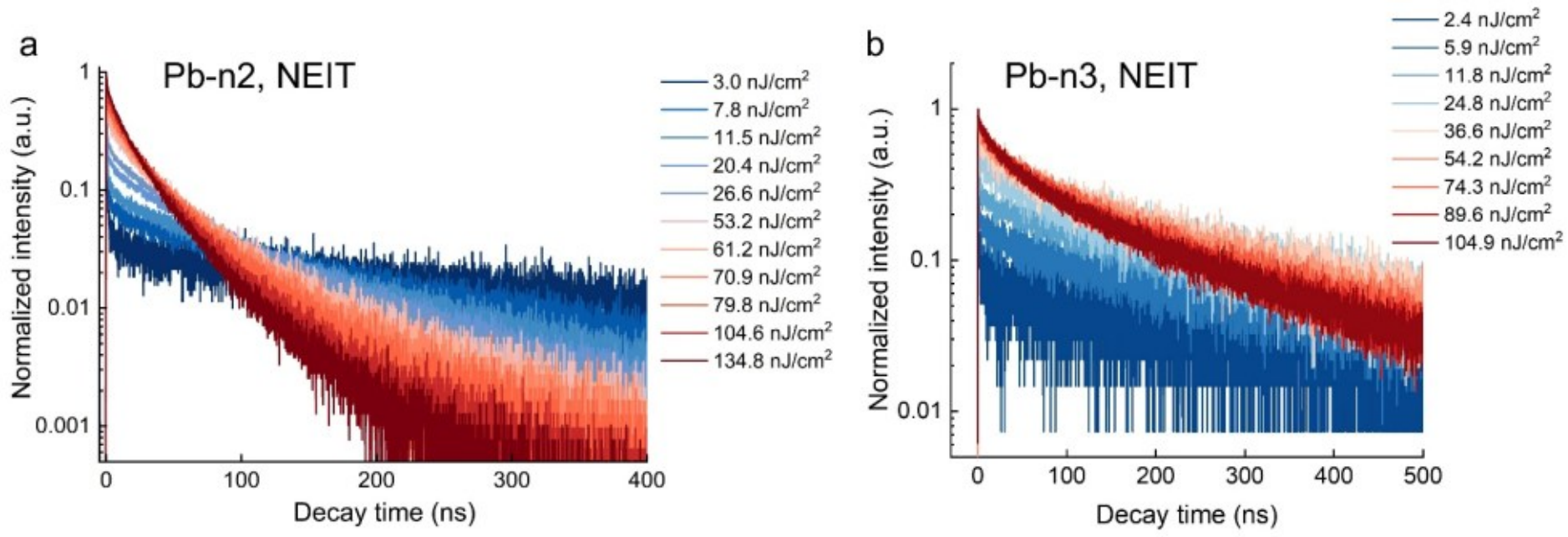

Supplementary Fig. 34. Power-dependent TRPL spectra of perovskites synthesized from NEIT growth. a. Power-dependent time-resolved PL spectra of Pb-n2 synthesized from NEIT growth with pump power increasing from 3.0 to 134.8 nJ/cm^2. **b.** Power-dependent time-resolved PL spectra of Pb-n3 synthesized from NEIT growth with pump power increasing from 2.4 to 104.9 nJ/cm^2.

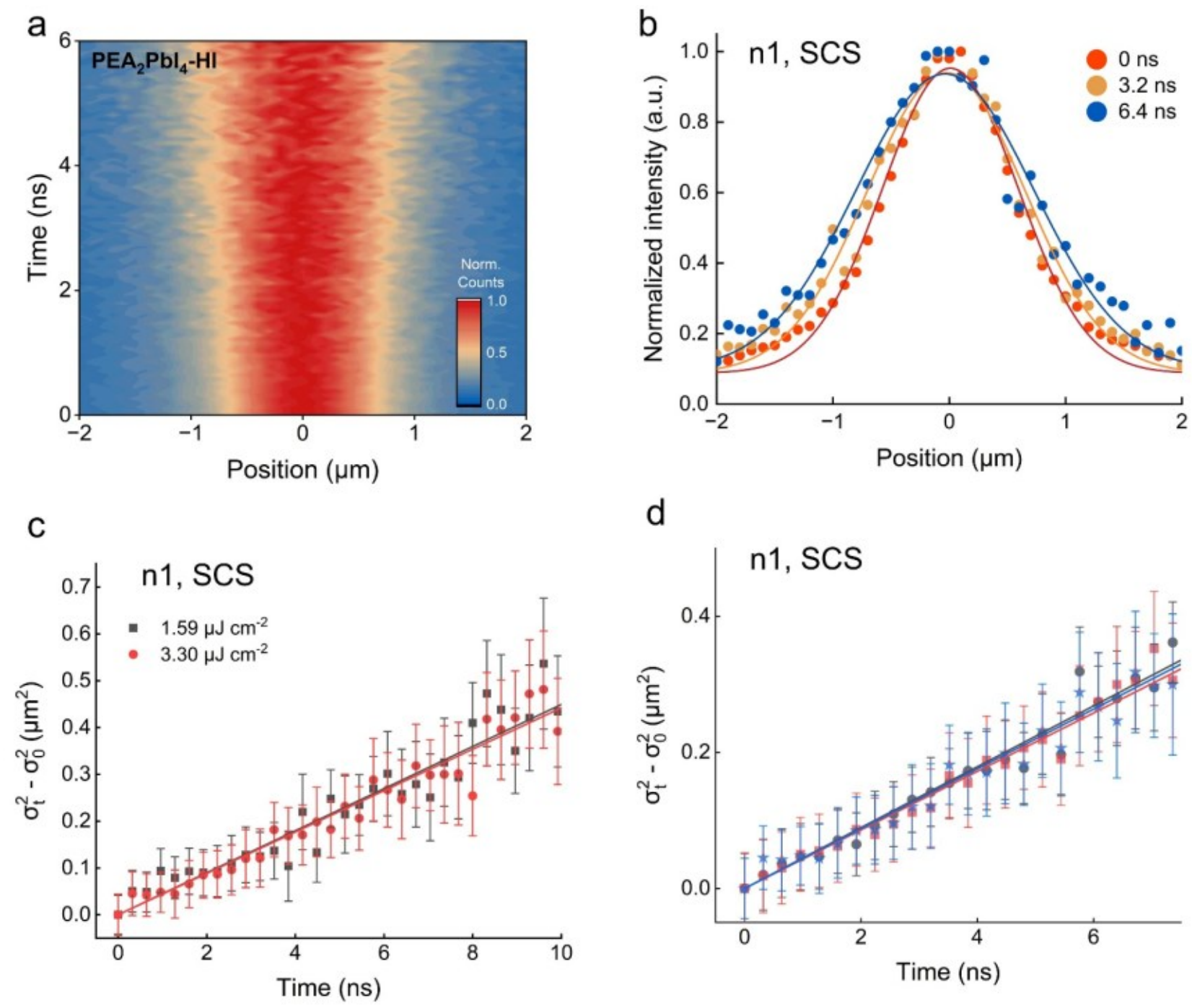

Supplementary Fig. 35. Carrier transport of Pb-n1 synthesized from SCS. a. Transient PL image of carrier diffusion in Pb-n1 synthesized from SCS. **b.** Spatial profiles at delay times of 0, 3.2, and 6.4 ns extracted from (**a**), with solid lines representing Gaussian fits. **c.** Comparison of carrier diffusion under low (1.59 μJ cm^{-2}) and high (3.30 μJ cm^{-2}) excitation power. The mean squared distribution (MSD, $\sigma_t^2 - \sigma_0^2$) exhibits a linear dependence on time (solid lines), with nearly identical diffusion constants (D): 0.224 ± 0.006 cm^2 s^{-1} at 1.59 μJ cm^{-2} and 0.221 ± 0.004 cm^2 s^{-1} at 3.30 μJ cm^{-2}. The MSD plots remains linear and the D values remain consistent, indicating negligible exciton–exciton interactions even at higher excitation powers and confirming the intrinsic nature of the measured carrier diffusion. **d.** Repeated carrier diffusion measurements on multiple exfoliated flakes yield D: 0.224 ± 0.003 cm^2 s^{-1}, 0.215 ± 0.003 cm^2 s^{-1}, 0.221 ± 0.004 cm^2 s^{-1}, further supporting the reproducibility of the intrinsic carrier diffusion. Error bars are presented as 95% confidence interval (CI).

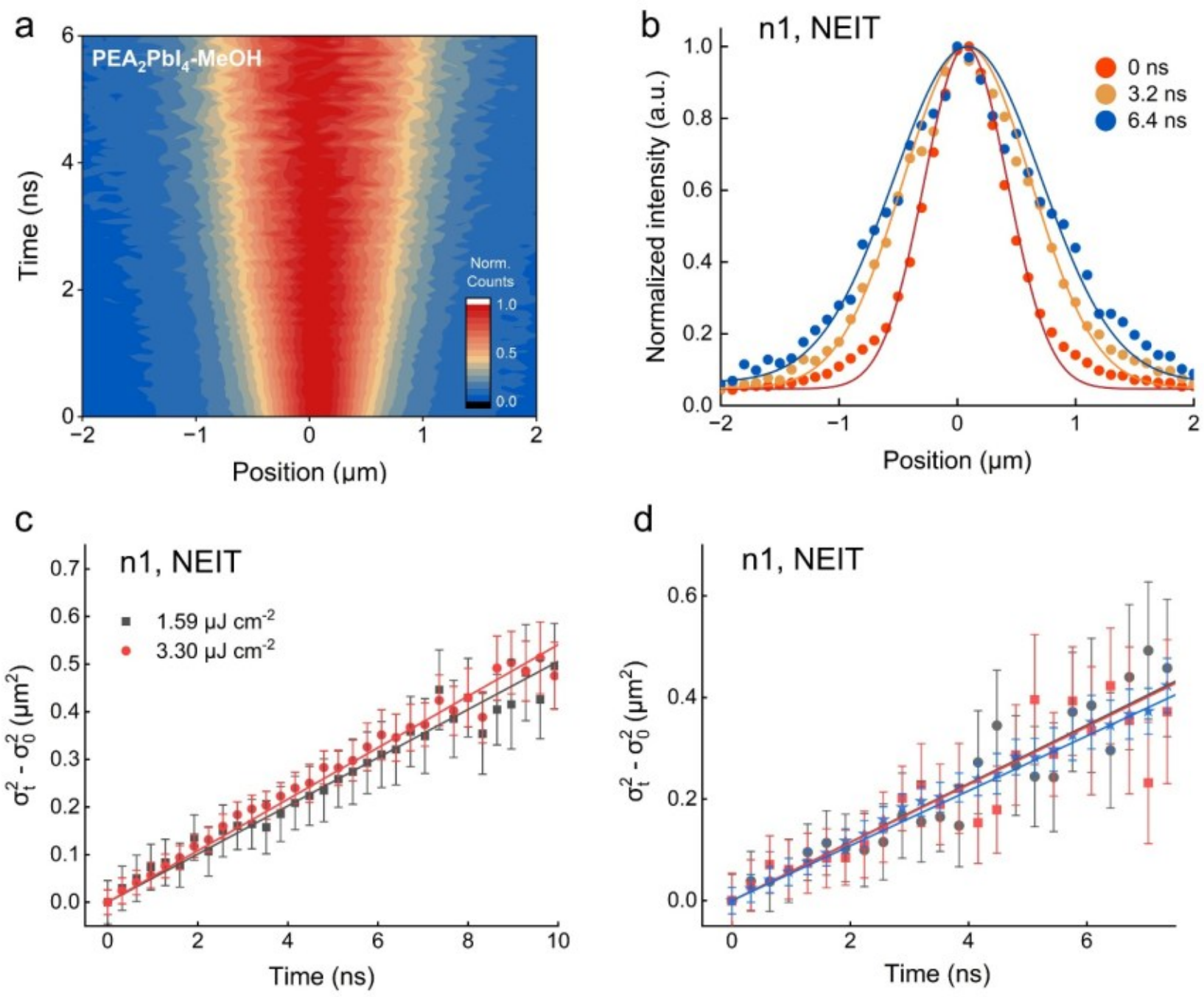

Supplementary Fig. 36. Carrier transport of Pb-n1 synthesized from NEIT growth. **a.** Transient PL image of carrier diffusion in Pb-n1 synthesized from NEIT growth. **b.** Spatial profiles at delay times of 0, 3.2, and 6.4 ns extracted from a, with solid lines representing Gaussian fits. **c.** Comparison of carrier diffusion under low (1.59 μJ cm^{-2}) and high (3.30 μJ cm^{-2}) excitation power. The mean squared distribution (MSD, $\sigma_t^2 - \sigma_0^2$) exhibits a linear dependence on time (solid lines), with nearly identical diffusion constants (D): 0.253 ± 0.004 cm^2 s^{-1} at 1.59 μJ cm^{-2} and 0.271 ± 0.003 cm^2 s^{-1} at 3.30 μJ cm^{-2}. The MSD plots remains linear and the D values remain consistent, indicating negligible exciton–exciton interactions even at higher excitation powers and confirming the intrinsic nature of the measured carrier diffusion. **d.** Repeated carrier diffusion measurements on multiple exfoliated flakes yield D: 0.288 ± 0.007 cm^2 s^{-1}, 0.285 ± 0.009 cm^2 s^{-1}, 0.271 ± 0.003 cm^2 s^{-1}, further supporting the reproducibility of the intrinsic carrier diffusion. Error bars are presented as 95% confidence interval (CI).

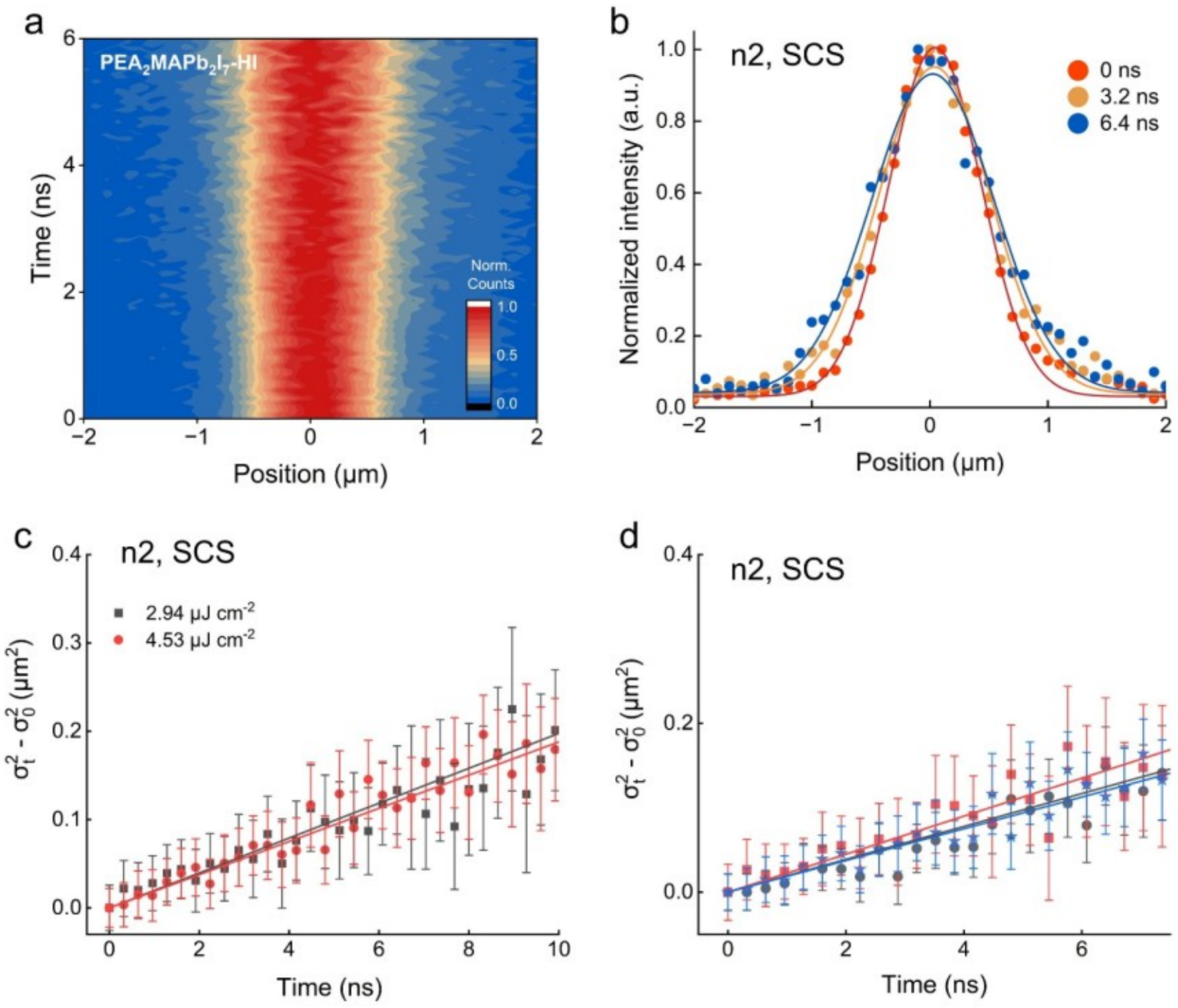

Supplementary Fig. 37. Carrier transport of Pb-n2 synthesized from SCS. a. Transient PL image of carrier diffusion in Pb-n2 synthesized from SCS. **b.** Spatial profiles at delay times of 0, 3.2, and 6.4 ns extracted from a, with solid lines representing Gaussian fits. **c.** Comparison of carrier diffusion under low (2.94 µJ cm^{-2}) and high (4.53 µJ cm^{-2}) excitation power. The mean squared distribution (MSD, $\sigma_t^2 - \sigma_0^2$) exhibits a linear dependence on time (solid lines), with nearly identical diffusion constants (D): 0.099 ± 0.003 cm^2 s^{-1} at 2.94 µJ cm^{-2} and 0.094 ± 0.003 cm^2 s^{-1} at 4.53 µJ cm^{-2}. The MSD plots remains linear and the D values remain consistent, indicating negligible exciton–exciton interactions even at higher excitation powers and confirming the intrinsic nature of the measured carrier diffusion. **d.** Repeated carrier diffusion measurements on multiple exfoliated flakes yield D: 0.097 ± 0.003 cm^2 s^{-1}, 0.113 ± 0.004 cm^2 s^{-1}, 0.094 ± 0.003 cm^2 s^{-1}, further supporting the reproducibility of the intrinsic carrier diffusion. Error bars are presented as 95% confidence interval (CI).

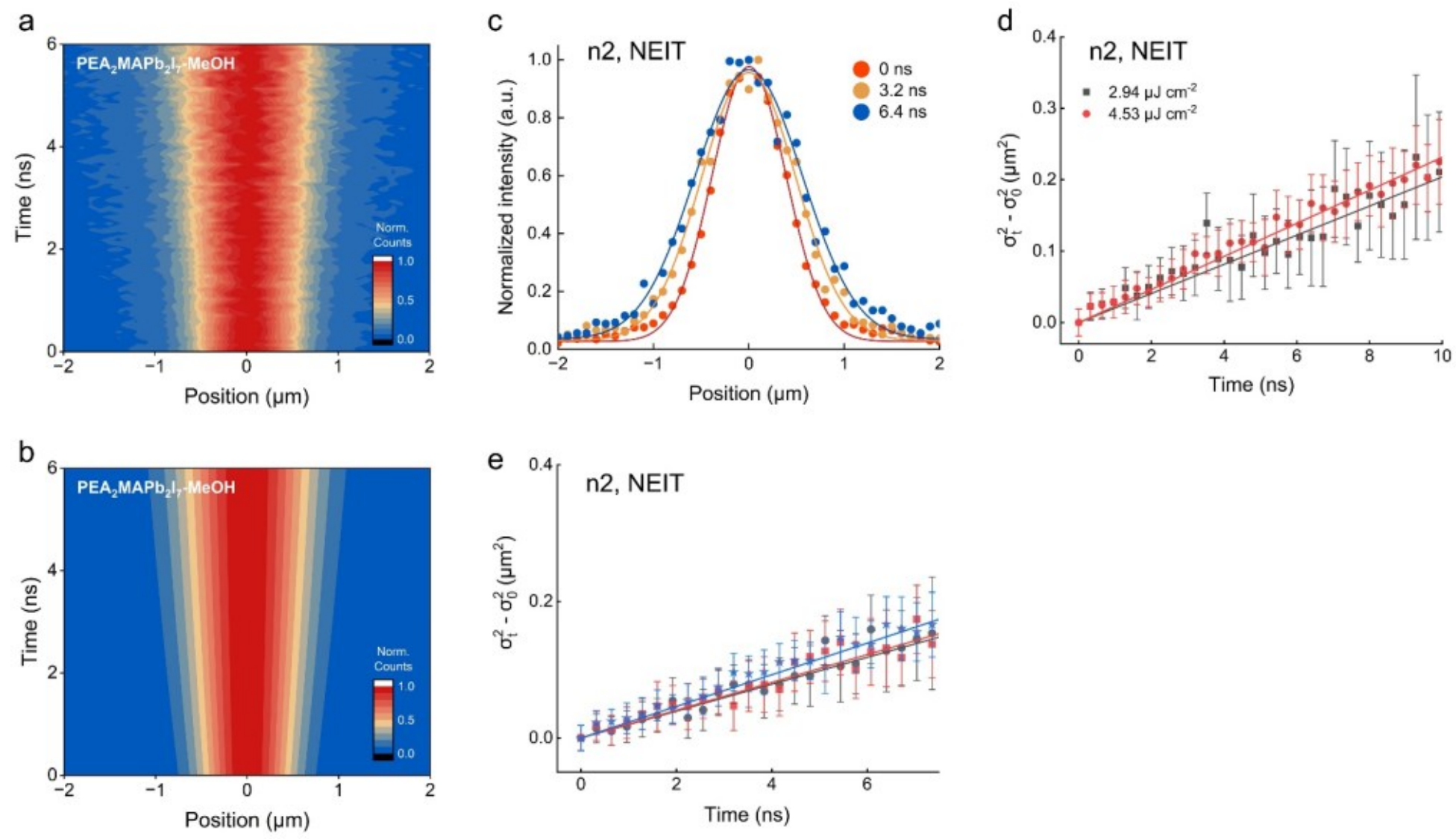

Supplementary Fig. 38. Carrier transport of Pb-n2 synthesized from NEIT growth. **a.** Transient PL image of carrier diffusion in Pb-n2 synthesized from NEIT growth. **b.** Fitting results for carrier diffusion in NEIT-synthesized Pb-n2. Experimental data a and fitting results b show excellent agreement. **c.** Spatial profiles at delay times of 0, 3.2, and 6.4 ns extracted from a, with solid lines representing Gaussian fits. **d.** Comparison of carrier diffusion under low (2.94 μJ cm^{-2}) and high (4.53 μJ cm^{-2}) excitation power. The mean squared distribution (MSD, $\sigma_t^2 - \sigma_0^2$) exhibits a linear dependence on time (solid lines), with nearly identical diffusion constants (D): 0.102 ± 0.003 $cm^2\,s^{-1}$ at 2.94 μJ cm^{-2} and 0.116 ± 0.002 $cm^2\,s^{-1}$ at 4.53 μJ cm^{-2}. The MSD plots remain linear and the D values remain consistent, indicating negligible exciton–exciton interactions even at higher excitation powers and confirming the intrinsic nature of the measured carrier diffusion. **e.** Repeated carrier diffusion measurements on multiple exfoliated flakes yield D: 0.098 ± 0.004 $cm^2\,s^{-1}$, 0.101 ± 0.003 $cm^2\,s^{-1}$, 0.116 ± 0.002 $cm^2\,s^{-1}$, further supporting the reproducibility of the intrinsic carrier diffusion. Error bars are presented as 95% confidence interval (CI).

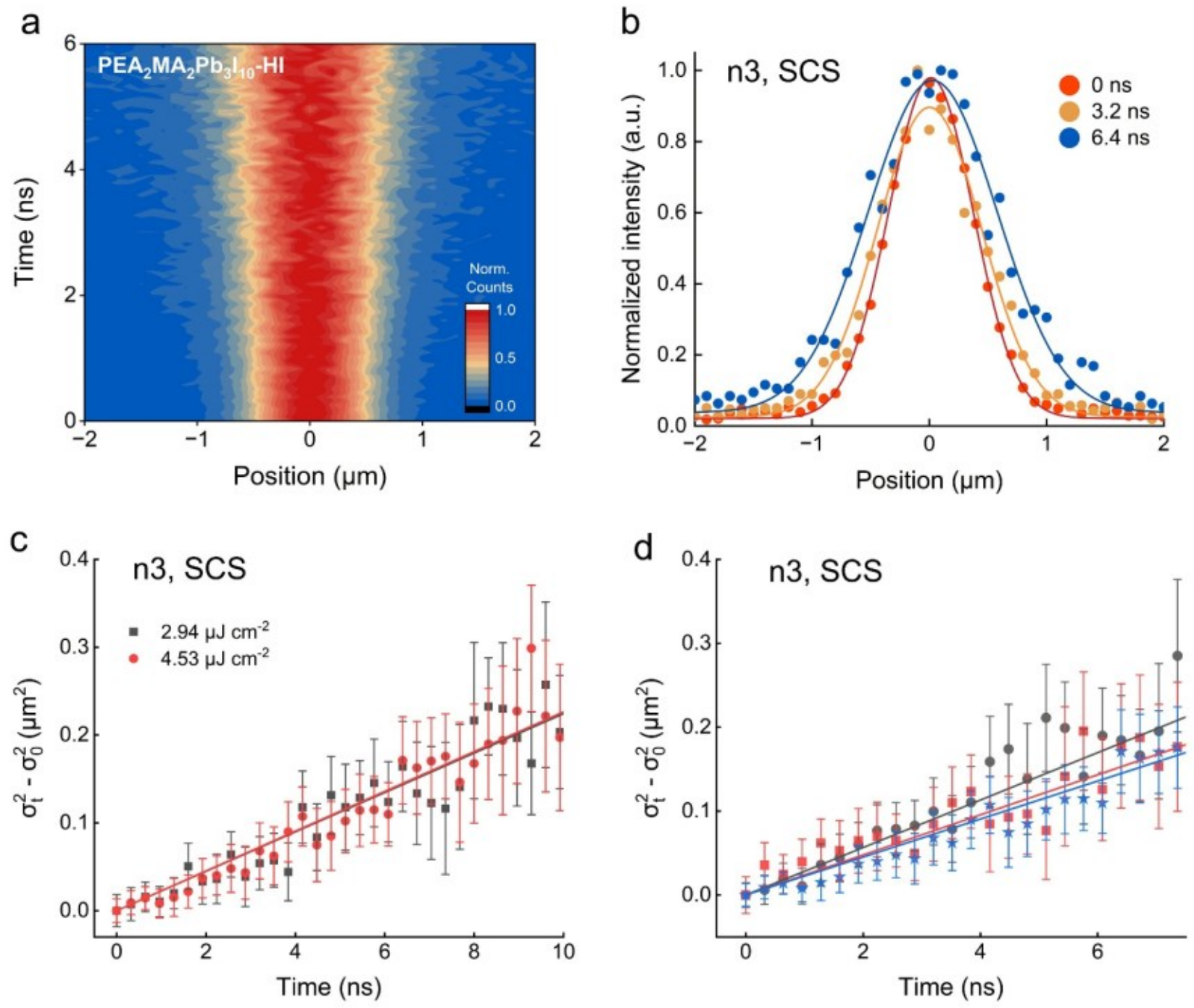

Supplementary Fig. 39. Carrier transport of Pb-n3 synthesized from SCS. a. Transient PL image of carrier diffusion in Pb-n3 synthesized from SCS. **b.** Spatial profiles at delay times of 0, 3.2, and 6.4 ns extracted from a, with solid lines representing Gaussian fits. **c.** Comparison of carrier diffusion under low (2.94 μJ cm^{-2}) and high (4.53 μJ cm^{-2}) excitation power. The mean squared distribution (MSD, $\sigma_t^2 - \sigma_0^2$) exhibits a linear dependence on time (solid lines), with nearly identical diffusion constants (D): 0.112 ± 0.003 cm^2 s^{-1} at 2.94 μJ cm^{-2} and 0.113 ± 0.003 cm^2 s^{-1} at 4.53 μJ cm^{-2}. The MSD plots remains linear and the D values remain consistent, indicating negligible exciton–exciton interactions even at higher excitation powers and confirming the intrinsic nature of the measured carrier diffusion. **d.** Repeated carrier diffusion measurements on multiple exfoliated flakes yield D: 0.142 ± 0.005 cm^2 s^{-1}, 0.119 ± 0.004 cm^2 s^{-1}, 0.113 ± 0.003 cm^2 s^{-1}, further supporting the reproducibility of the intrinsic carrier diffusion. Error bars are presented as 95% confidence interval (CI).

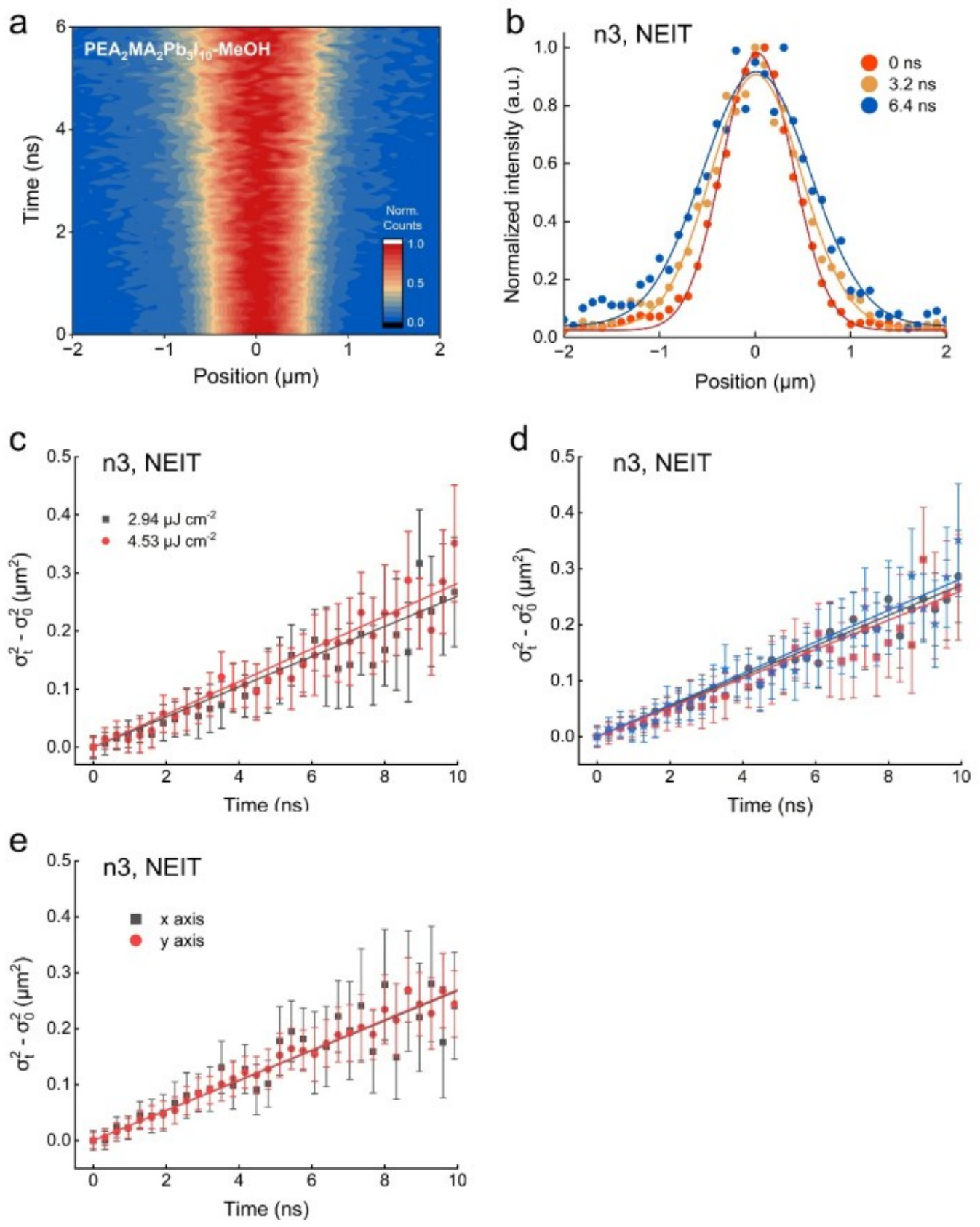

Supplementary Fig. 40. Carrier transport of Pb-n3 synthesized from NEIT growth. **a.** Transient PL image of carrier diffusion in Pb-n3 synthesized from NEIT growth. **b.** Spatial profiles at delay times of 0, 3.2, and 6.4 ns extracted from a, with solid lines representing Gaussian fits. **c.** Comparison of carrier diffusion under low (2.94 μJ cm^{-2}) and high (4.53 μJ cm^{-2}) excitation power. The mean squared distribution (MSD, $\sigma_t^2 - \sigma_0^2$) exhibits a linear dependence on time (solid lines), with nearly identical diffusion constants (D): 0.130 ± 0.004 cm^2 s^{-1} at 2.94 μJ cm^{-2} and 0.141 ± 0.004 cm^2 s^{-1} at 4.53 μJ cm^{-2}. The MSD plots remains linear and the D values remain consistent, indicating negligible exciton–exciton interactions even at higher excitation powers and confirming the intrinsic nature of the measured carrier diffusion. **d.** Repeated carrier diffusion measurements on multiple exfoliated flakes yield D: 0.135 ± 0.003 cm^2 s^{-1}, 0.130 ± 0.004 cm^2 s^{-1}, 0.141 ± 0.004 cm^2 s^{-1}, further supporting the reproducibility of the intrinsic carrier diffusion. **e.** Anisotropy testing of Pb-n3 samples. D: 0.134 ± 0.002 cm^2 s^{-1} (x axis), 0.135 ± 0.001 cm^2 s^{-1} (y axis). Error bars are presented as 95% confidence interval (CI).

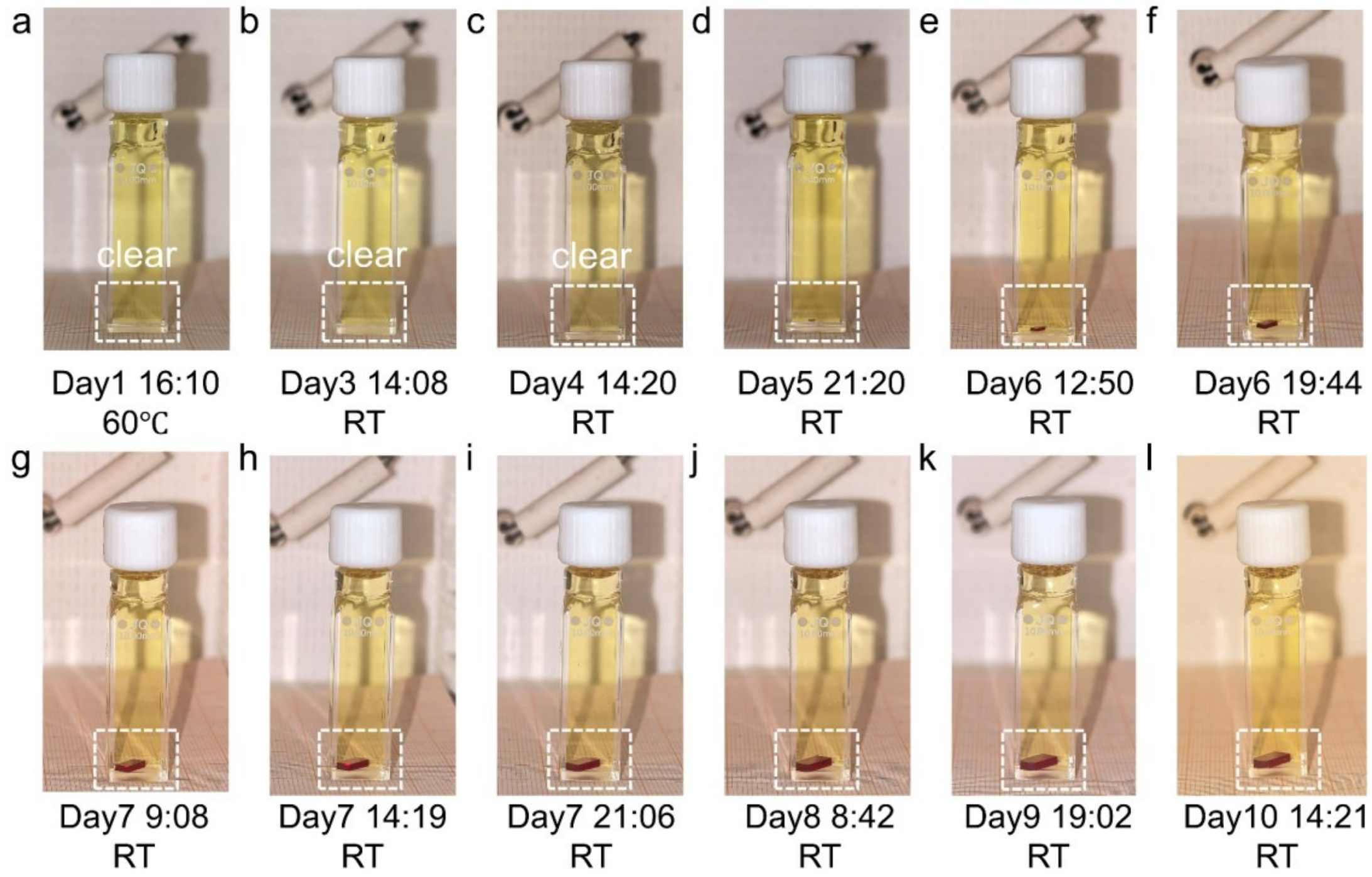

Supplementary Fig. 41. Continuous tracking of the living NEIT single crystal growth process of Pb-n2. a. The precursor solution initially placed in the muffle furnace at 60 °C. **b-c.** The solution cooled down and maintained at RT. The solution remained visibly clear. **d-l.** Continuous image records of the growth stages from Day 5 to Day 10.

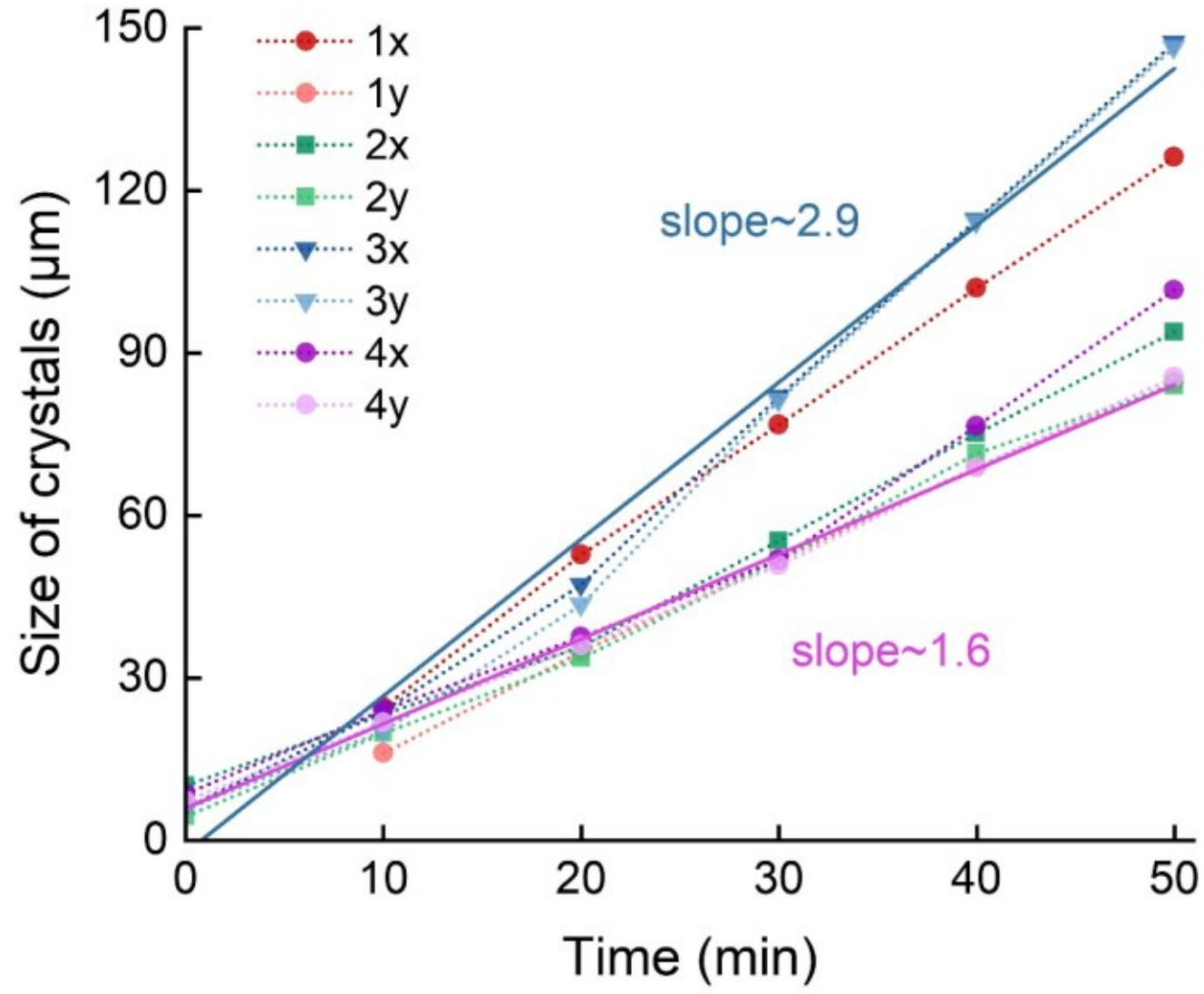

Supplementary Fig. 42. Evolution of crystal sizes and growth rates in the x- and y-directions of the four Pb-n2 crystals in Fig. 3e during 50 min of in-situ growth at constant RT. The data were extracted from video S1.

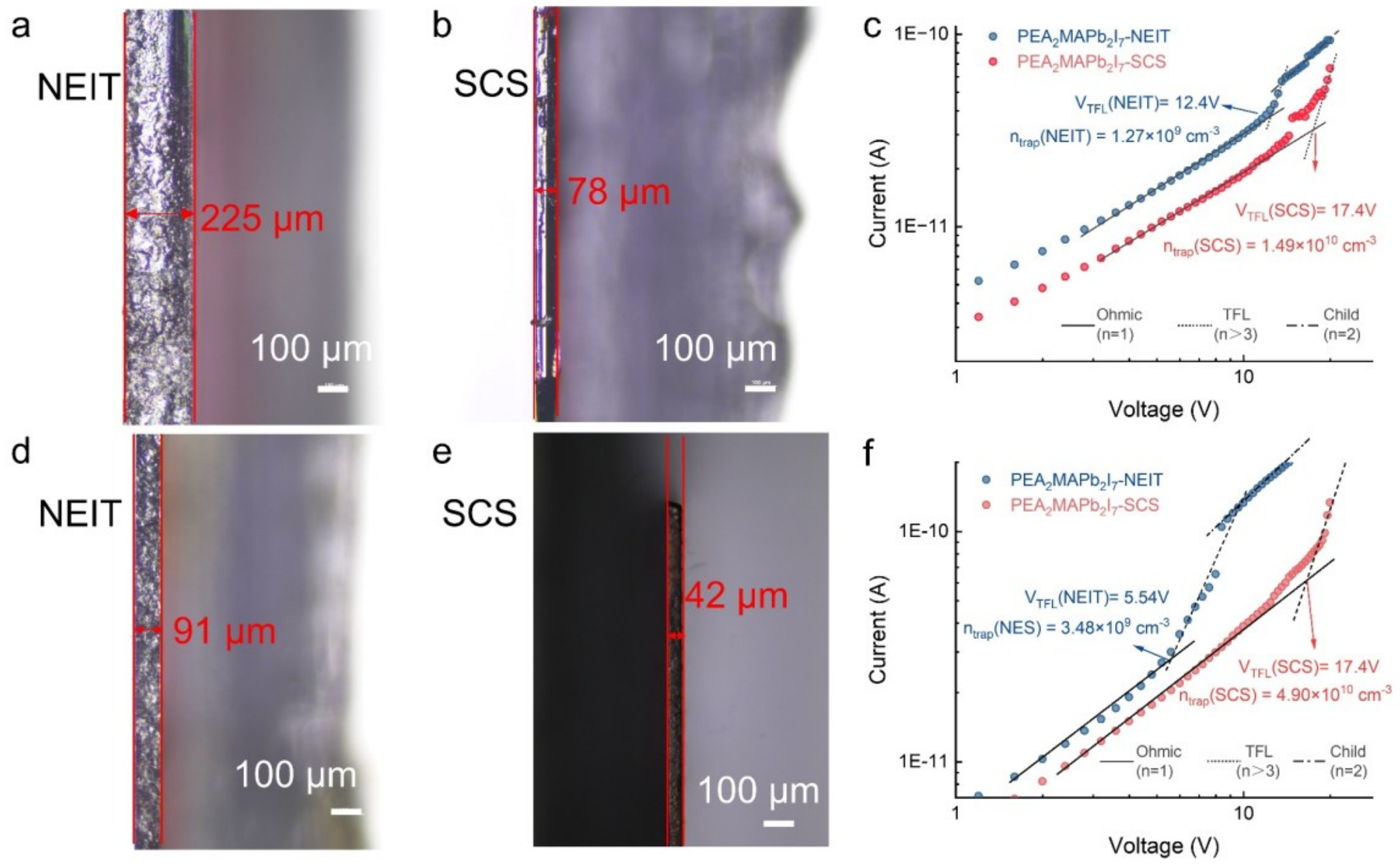

Supplementary Fig. 43. Reproducibility of SCLC measurements and cross-sectional thickness determination of $PEA_2MAPb_2I_7$ single crystals. a, b, Images and thicknesses of the NEIT (**a**, 225 μm) and SCS (**b**, 78 μm) samples in the manuscript. **c.** The corresponding dark current-voltage (I-V) characteristics of the SCLC measurements in **a** and **b**. **d, e,** Thickness of a newly fabricated batch of NEIT (**d**, 91 μm) and SCS (**e**, 42 μm) single crystals. **f.** The corresponding dark current-voltage (I-V) characteristics in **d** and **e**.

The accuracy of SCLC fundamentally depends on the precise determination of the active layer thickness (L), as the trap density (n_{trap}) is calculated by: $n_{trap} = \frac{2\varepsilon\epsilon_0 V_{TFL}}{eL^2}$.

To eliminate the geometrical uncertainties warned by Blakesley et al., we directly measured the exact cross-sectional thickness (L) of each single crystal using optical microscopy. To definitively prove reproducibility, we conducted SCLC measurements on a new batch of crystals with significantly reduced thicknesses (91 μm for NEIT and 42 μm for SCS, compared to the original 225 μm and 78 μm). Despite the substantial variations in L and the corresponding shifts in the trap-filled limit voltage (V_{TFL}), the derived n_{trap} values remained consistently within the identical order of magnitude (3.48×10^{9} for NEIT and 4.90×10^{10} for SCS).

For 2D hybrid perovskites, the average dielectric constant (ε_{ave}) is a volume-weighted

composite of the inorganic well (ω) and the organic barrier (b): $\varepsilon_{ave} = \frac{\varepsilon_\omega L_\omega + \varepsilon_b L_b}{L_\omega + L_b}$.

Based on structural data, we applied $\varepsilon_\omega = 6.1$ and $L_\omega = 12.8$ Å for the inorganic layer. Because the organic spacer comprises a bilayer, its effective thickness was doubled, yielding $\varepsilon_b = 3.3$ and $L_b = 6.7$ Å $\times 2$. This precise calculation yields an $\varepsilon_{ave} = 4.7$, which was strictly applied in our n_{trap} calculations.

The thermally evaporated C60 layer acts as a critical physical and chemical buffer. It ensures an ideal Ohmic contact for accurate SCLC extraction while preventing direct chemical reactions between the high-temperature Ag and the perovskite surface, mitigating surface defect generation.

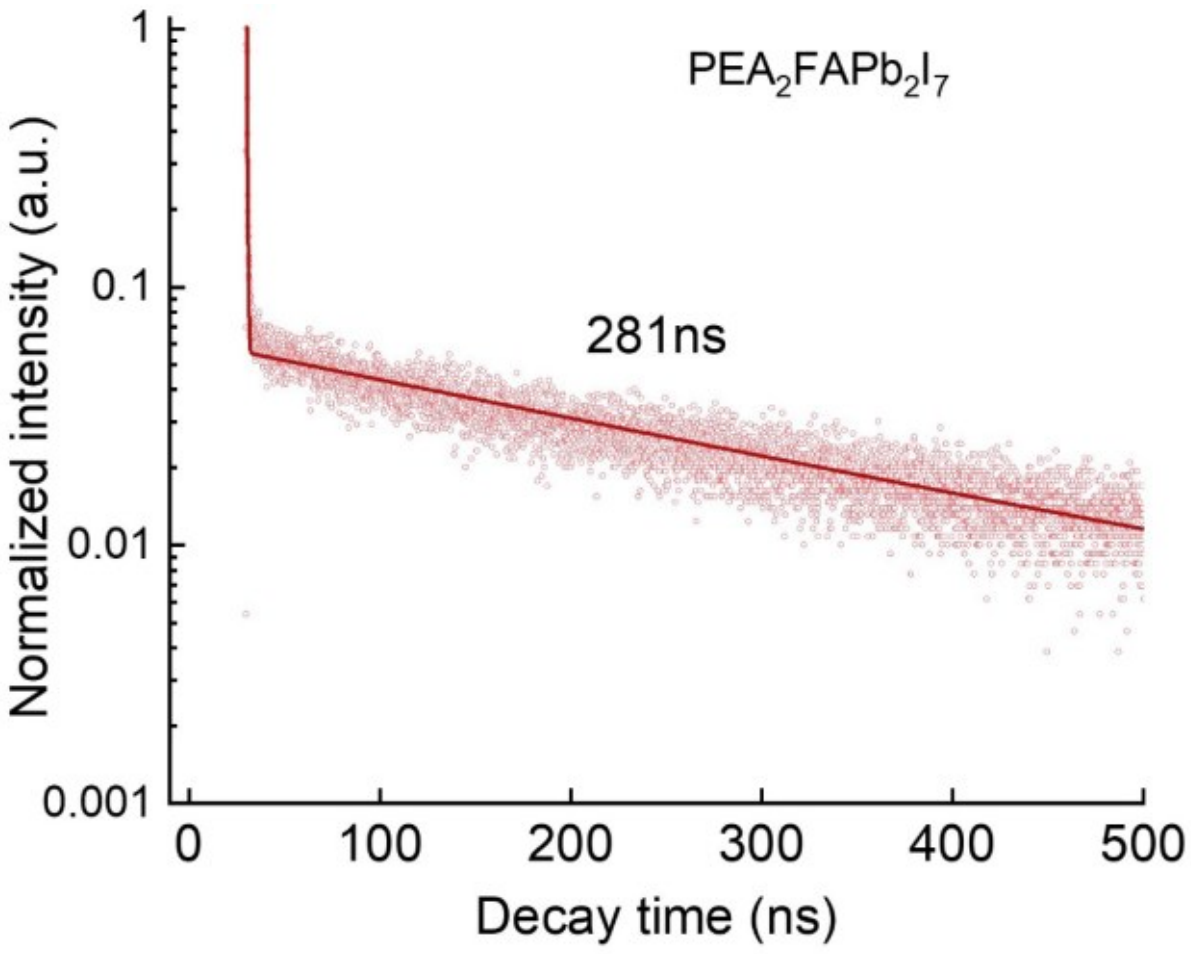

Supplementary Fig. 44. TRPL spectrum of $PEA_2FAPb_2I_7$ synthesized from NEIT growth.

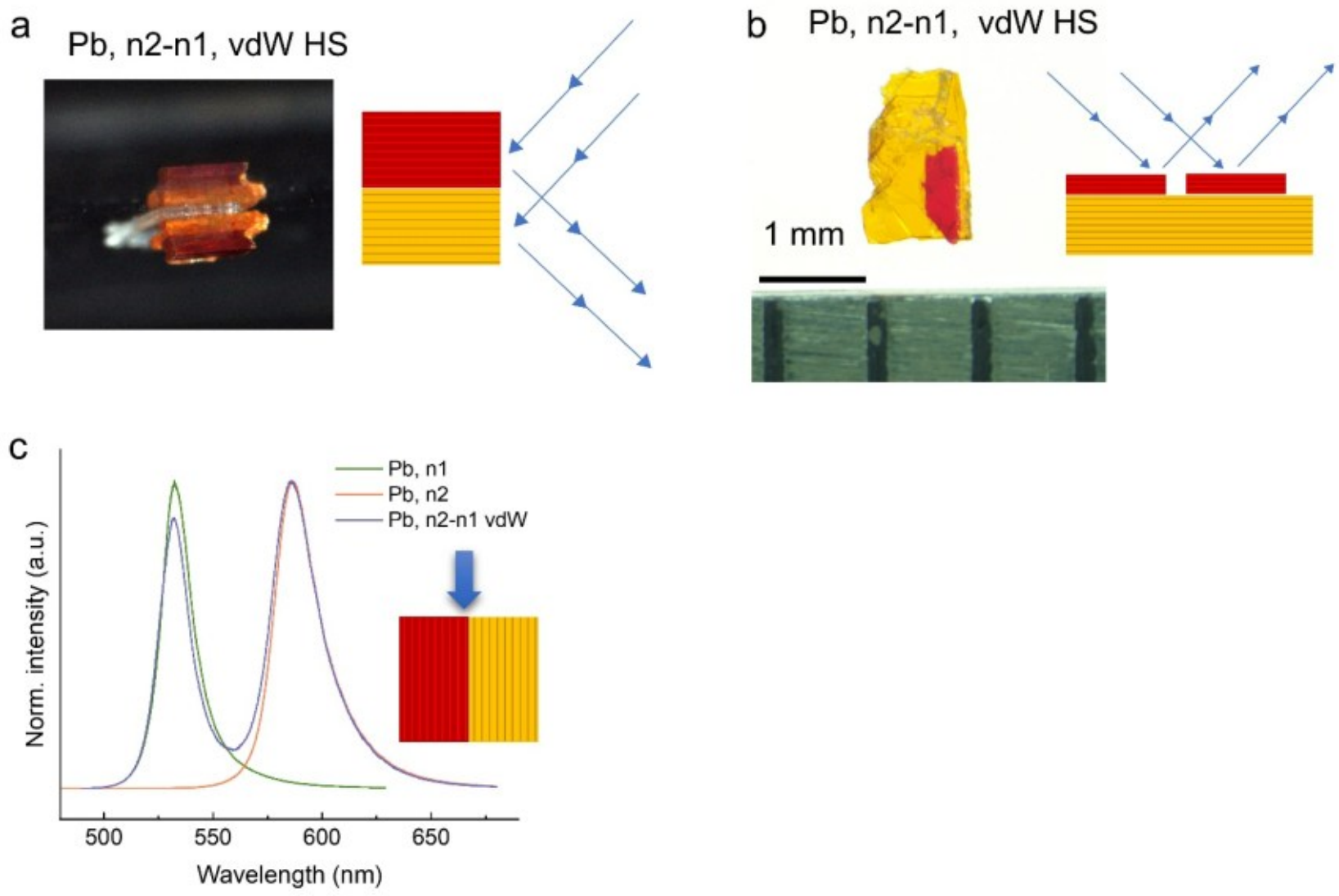

Supplementary Fig. 45. XRD characterizations and PL spectra of Pb, n2-n1 vdW heterostructure (HS). a. Optical image of Pb, n2-n1 vdW heterostructure for HRXRD measurement, along with a schematic of the Pb, n2-n1 vdW heterostructure orientation during HRXRD measurements. **b.** Sample image of the Pb, n2-n1 vdW heterostructure for PXRD measurement of the (00k) plane, with a schematic illustration of the heterostructure orientation during PXRD measurements. **c.** PL spectra of Pb, n2-n1 vdW heterostructure and a corresponding schematic of the crystal orientation during PL spectra measurements.

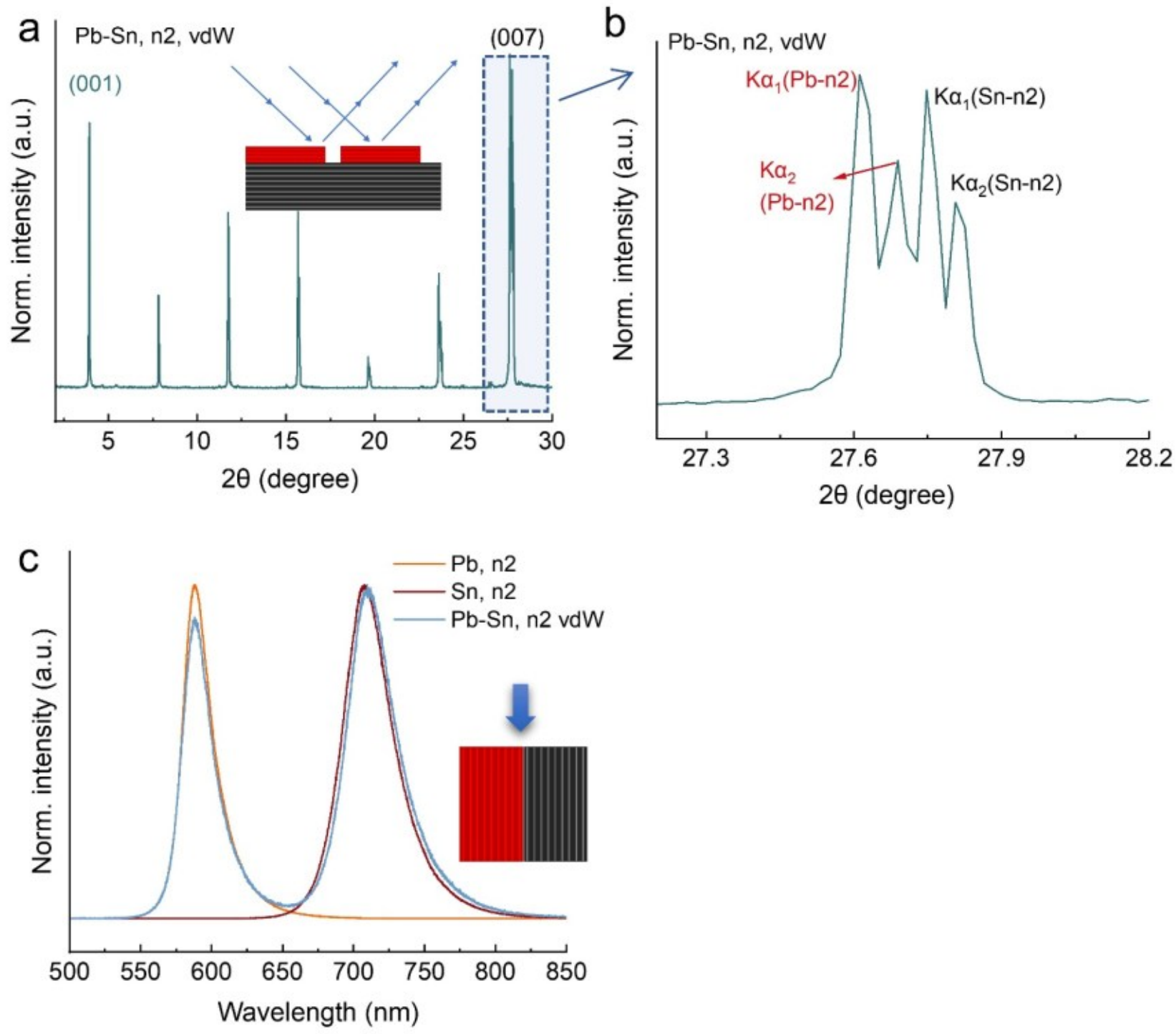

Supplementary Fig. 46. PXRD characterization and PL spectra of Pb-Sn, n2, vdW heterostructure. a. PXRD pattern of Pb-Sn, n2, vdW heterostructure. The inset represents a schematic of the Pb-Sn, n2 vdW heterostructure orientation during PXRD measurements. **b.** Magnified region around the (007) diffraction peak, showing four diffraction peaks corresponding to $K\alpha_1$ and $K\alpha_2$ for both Pb-n2 and Sn-n2. **c.** PL spectra of Pb-Sn, n2 vdW heterostructure, and the inset represents a schematic of the Pb-Sn, n2 vdW heterostructure orientation during PL spectra measurements.

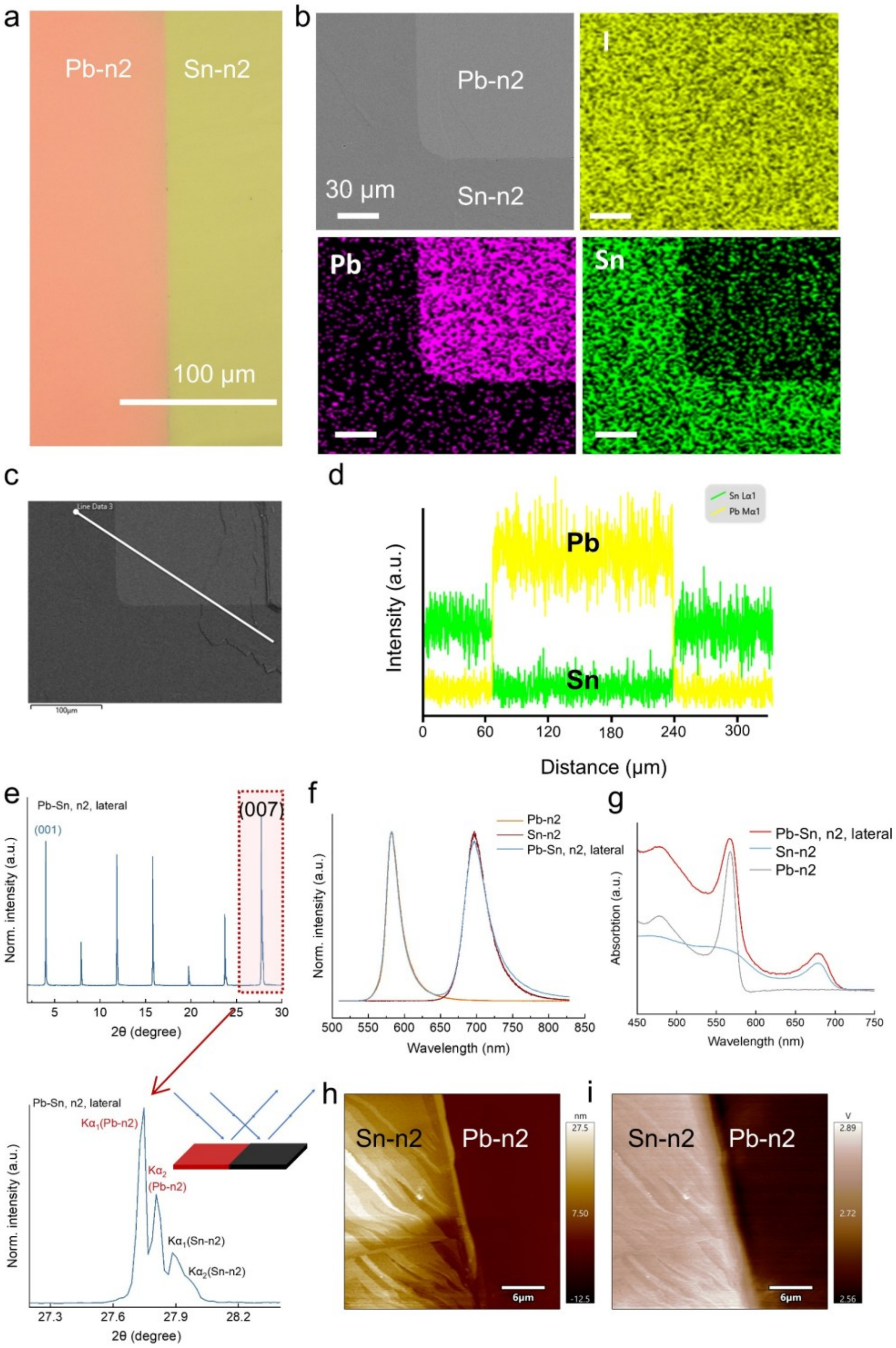

Supplementary Fig. 47. Detailed characterizations of Pb-Sn, n2 lateral heterostructures. a. Optical image of Pb-Sn, n2 lateral heterostructure. **b.** The SEM image and corresponding EDS elemental mappings (I, Pb and Sn) of Pb-Sn, n2 lateral heterostructure. **c, d.** SEM image of the Pb-Sn, n2 lateral heterostructure and the corresponding quantitative EDS line scan profiles of Pb and Sn elements along the path

indicated in (c). **e.** PXRD pattern of Pb-Sn, n2 lateral heterostructure. The magnified region around the (007) diffraction peak reveals four distinct peaks corresponding to $K\alpha_1$ and $K\alpha_2$ form both Pb-n2 and Sn-n2. The inset represents a schematic of the Pb-Sn, n2 lateral heterostructure orientation during PXRD measurements. **f**, **g.** PL spectra and UV-Vis spectra of Pb-Sn, n2 lateral heterostructure, along with reference spectra from individual Pb-n2 and Sn-n2 domains. **h**, **i.** AFM topography and KPFM surface potential images of Pb-Sn, n2 lateral heterostructure.

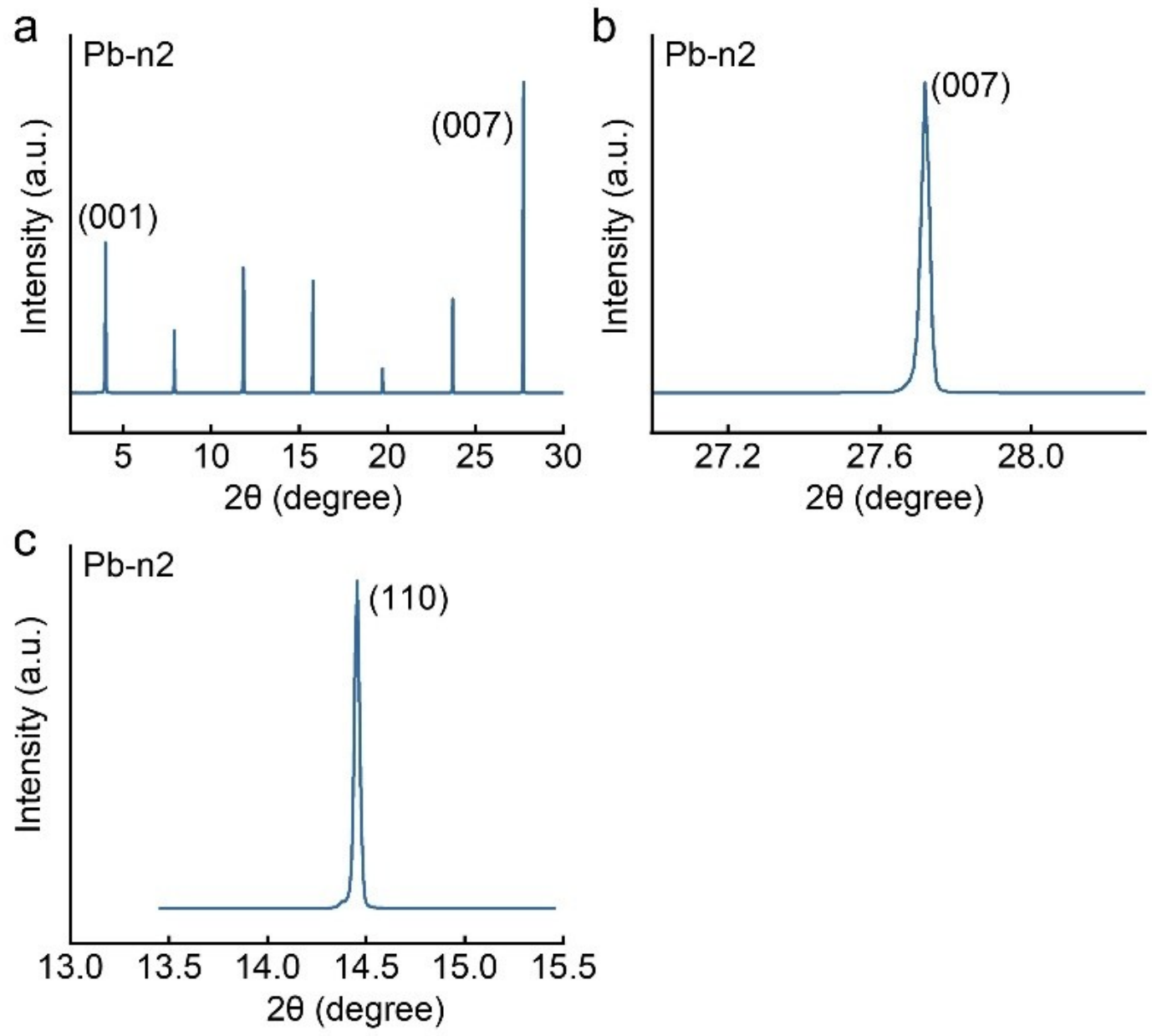

Supplementary Fig. 48. High-resolution 1D XRD pattern of Pb-n2 single crystal. a. High-resolution 1D XRD pattern of Pb-n2 single crystals in (00k) plane. **b.** The magnified region of (a) around the (007) diffraction peak. **c.** High-resolution 1D XRD pattern of Pb-n2 single crystals in (110) plane.

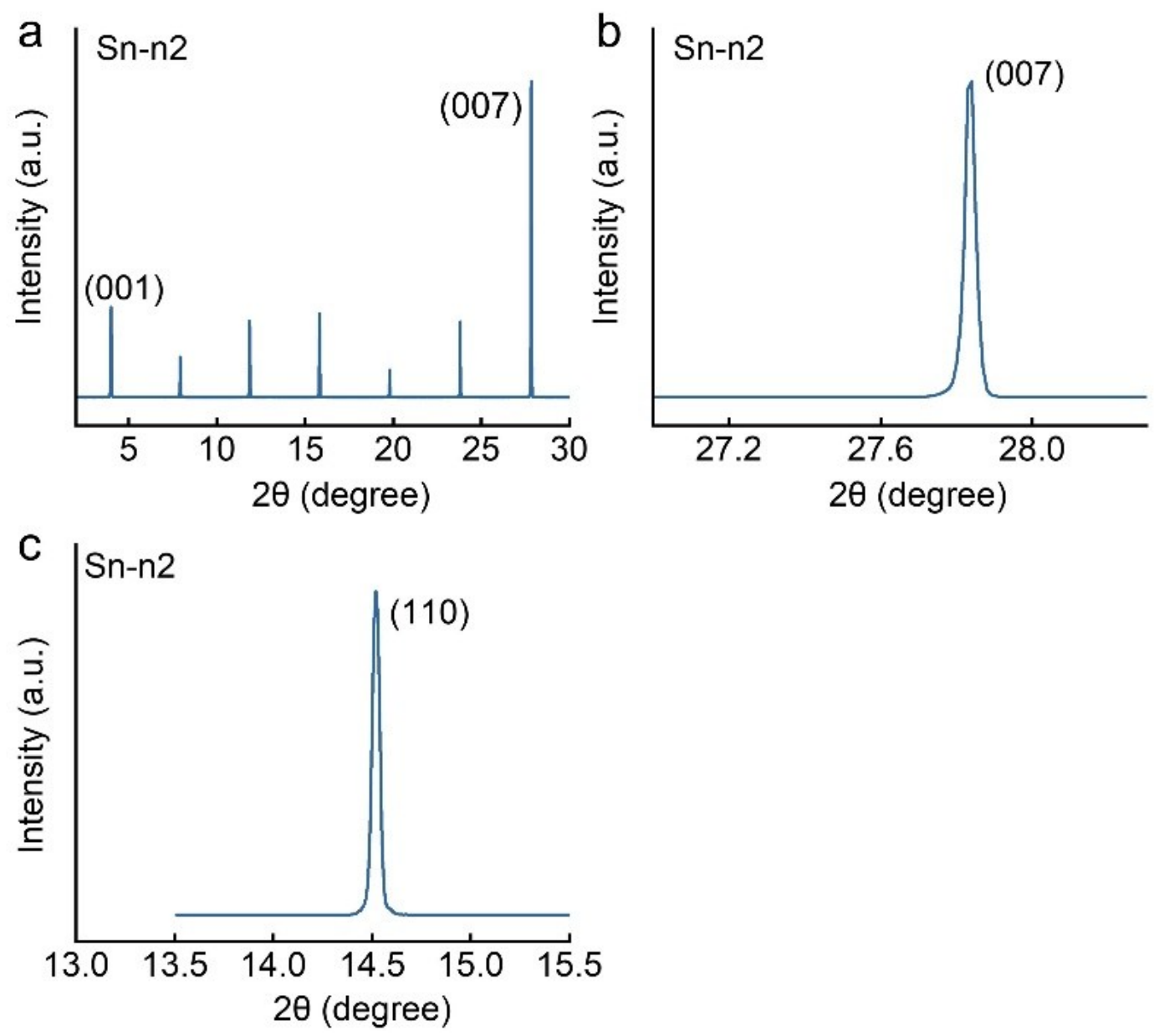

Supplementary Fig. 49. High-resolution 1D XRD pattern of Sn-n2 single crystal. **a.** High-resolution 1D XRD pattern of Sn-n2 single crystals in (00k) plane. **b.** The magnified region of (a) around the (007) diffraction peak. **c.** High-resolution 1D XRD pattern of Sn-n2 single crystals in (110) plane.

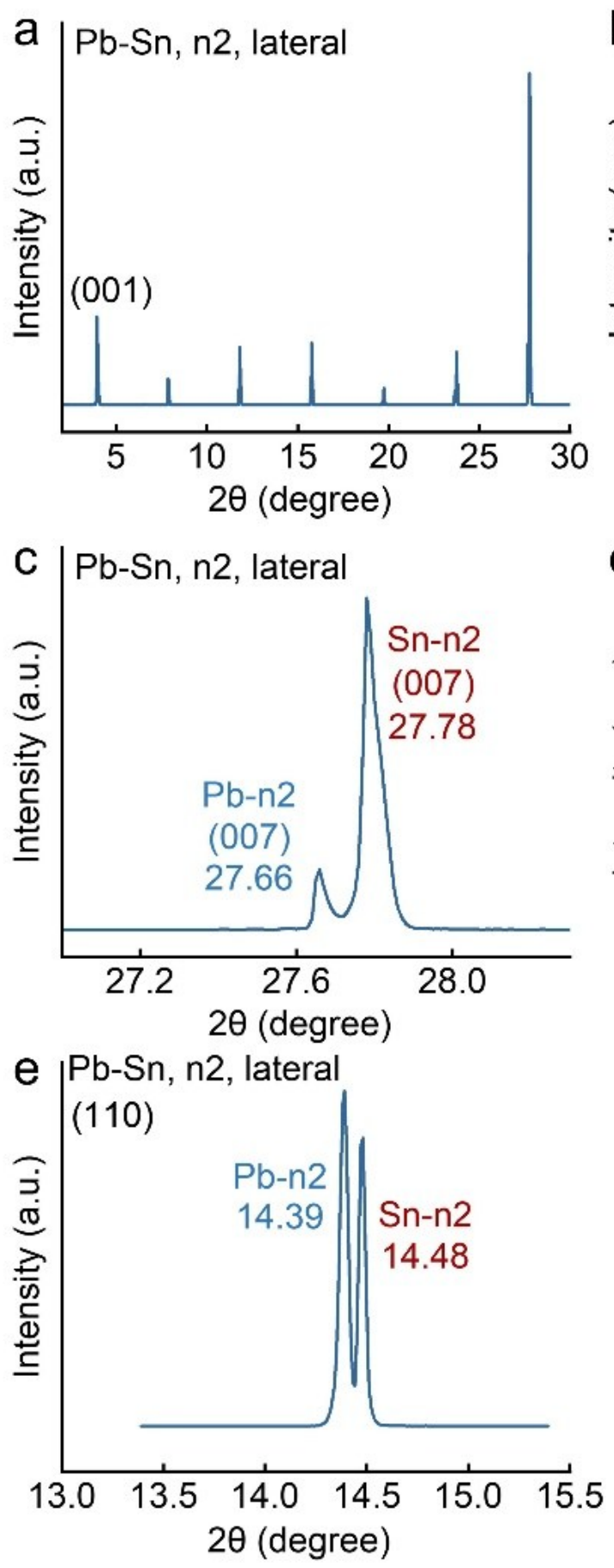

Supplementary Fig. 50. High-resolution 1D XRD pattern of Pb-Sn, n2 lateral heterostructure. a. High-resolution 1D XRD pattern of Pb-Sn, n2 lateral heterostructure in (00k) plane. **b, c.** The magnified region of (a) around the (004) and (007) diffraction peaks reveal 2 distinct peaks corresponding to Pb-n2 and Sn-n2. **d.** High-resolution 1D XRD pattern of Pb-n2 single crystals in (110) plane. **e.** The fine scan of (110) plane reveal 2 distinct peaks corresponding to Pb-n2 and Sn-n2.

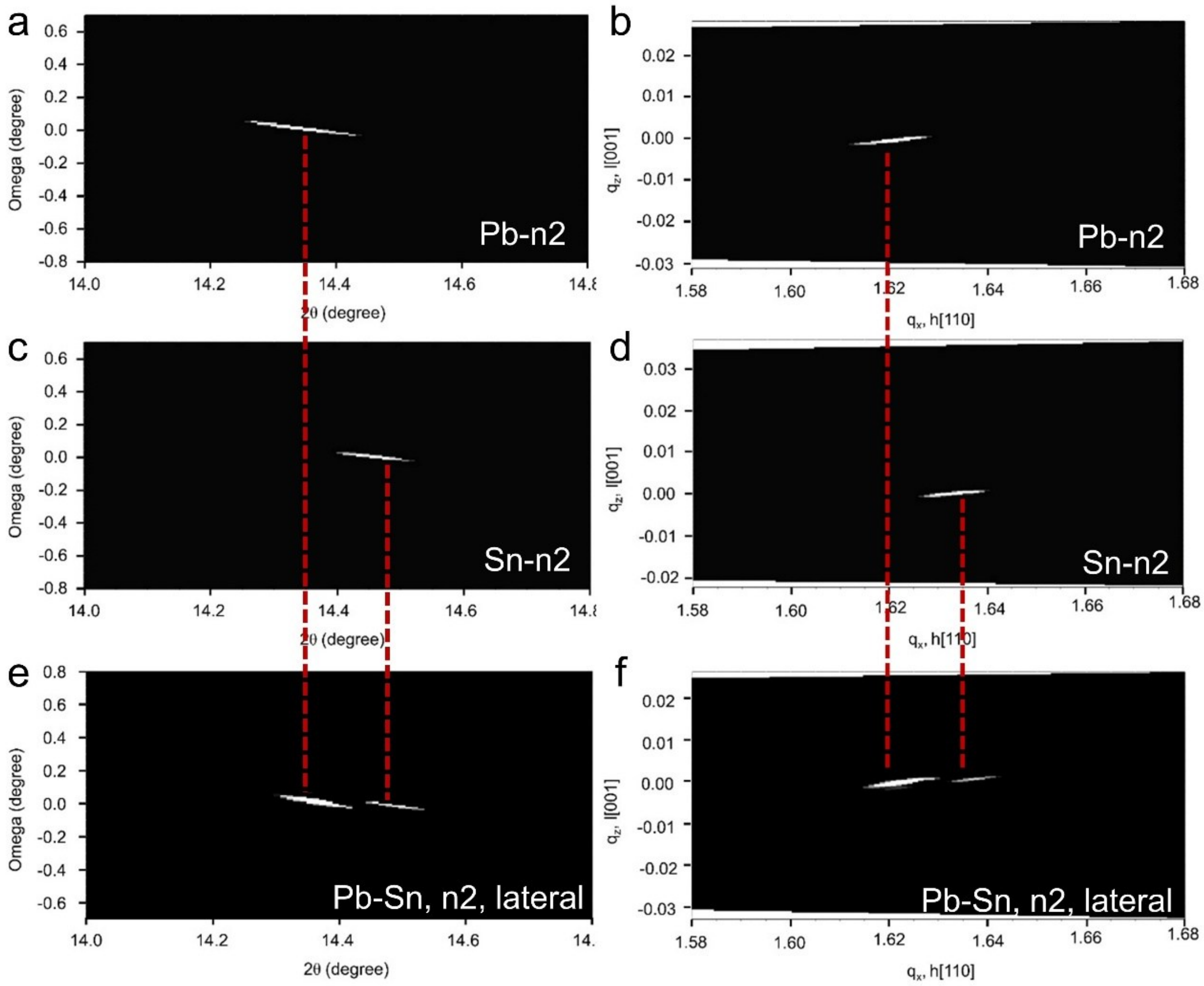

Supplementary Fig. 51. Crystallographic evidence for the in-plane orientational epitaxy of Pb-Sn, n2 lateral heterostructure. Raw RSM data of the (110) planes for pure Pb-n2 single crystal (**a**), pure Sn-n2 single crystal (**c**), and the Pb-Sn n2 lateral heterostructure (**e**). RSM of the (110) planes converted by the LEPTOS software for the pure Pb-n2 (**b**), pure Sn-n2 (**d**), and the heterostructure (**f**) samples. Both the raw data and RSM maps reveal that the diffraction peaks of the pure Pb-n2 and Sn-n2 single crystals align with those observed in the heterostructure.

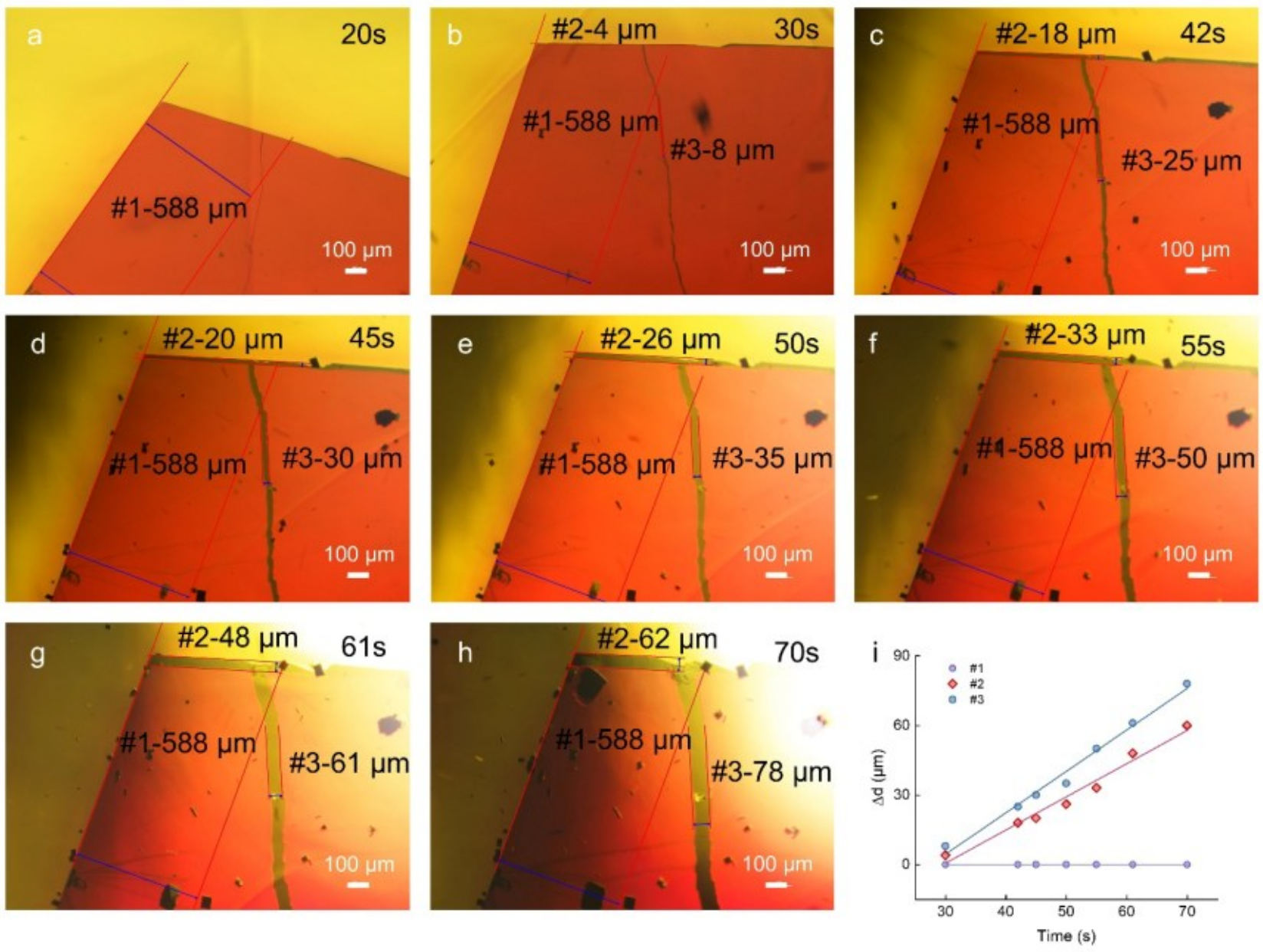

Supplementary Fig. 52. Epitaxial growth process of Pb-Sn, n2 heterostructures. a–h. Epitaxial growth process of Pb-Sn, n2 heterostructures during 20-70s. To visualize the epitaxial growth in real time under microscopy, a Pb-n2 single crystal was first deposited, followed by injection of a Sn-n2 precursor solution into the liquid cell. The subsequent morphological evolution of the crystal surface was monitored in situ after solution introduction. The left edge of the Pb-n2 crystal was intentionally modified to suppress epitaxial growth, providing a fixed reference line for tracking both the dissolution of Pb-n2 and the epitaxial deposition of Sn-n2. Initially, a distinct step was on the Pb-n2 surface, and a characteristic point was selected on this step. #1 was defined as the perpendicular distance between this characteristic point and the left Pb-n2 crystal edge. Throughout the observed period (20–70 s), the width of #1 remained constant. Meanwhile, along the upper edge of the Pb-n2 crystal and to the right of the step, black Sn-n2 crystals grew epitaxially along the red Pb-n2 substrate. The dimensions of these newly grown Sn-n2 regions, labeled #2 and #3, increased continuously over time. **i**. The change of Δd of #1, #2 and #3 with time. The constant value of #1, coupled with the progressive expansion of #2 and #3, indicates negligible dissolution of the Pb-n2 crystal during epitaxial growth—a critical factor for the formation of a high-quality heterointerface in the Pb–Sn, n2 heterostructure.

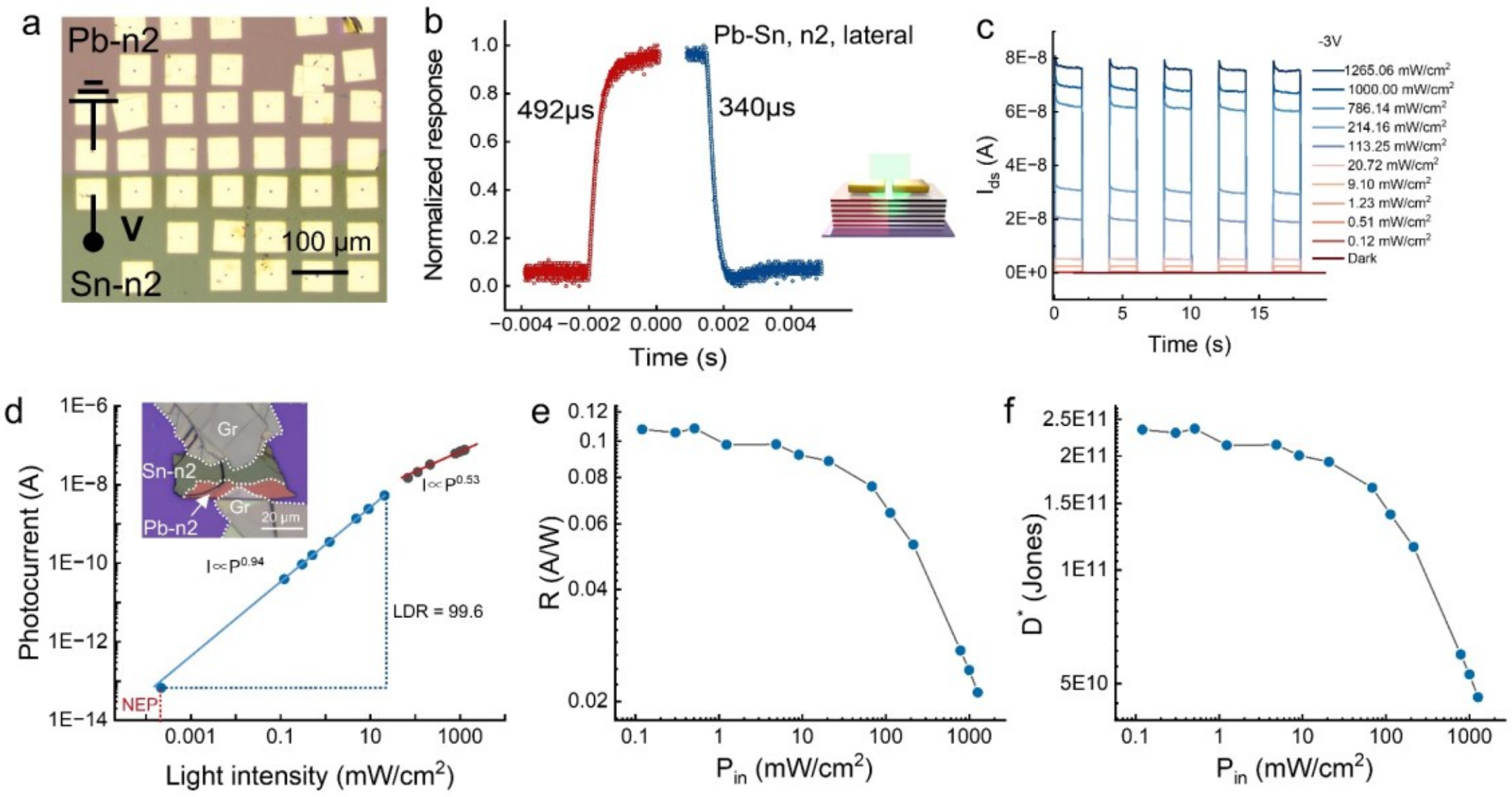

Supplementary Fig. 53. Photodetection performance of Pb-Sn, n2 lateral heterostructure under 500 nm illumination with Pb-n2 grounded and Sn-n2 biased. **a.** The optical image of the macroscopic two-terminal Pb-Sn, n2 lateral heterostructure photodetector. **b.** The response times of Pb-Sn, n2 lateral heterostructure macroscopic device measured at –3 V bias. The inset represents the schematic diagram of two-terminal photodetector. **c–f,** Measurements of a mechanically exfoliated micro- Pb-Sn, n2 lateral heterostructure. All data were recorded under 500 nm illumination at -3 V bias. **c,** Photocurrent switching behaviour under illumination power densities ranging from 0.12 to 1265.06 mW/cm^2, recorded at a bias voltage of –3 V (Pb-n2 grounded, Sn-n2 biased). **d,** Power-dependent photocurrent. The device exhibits a linear dynamic range (LDR) of 99.6 dB ($I \propto P^{0.94}$), followed by a sublinear regime ($I \propto P^{0.53}$) at high power intensities. **e, f,** Responsivity (R) and specific detectivity (D^*) as functions of irradiation power intensity (P_{in}) at –3 V bias.

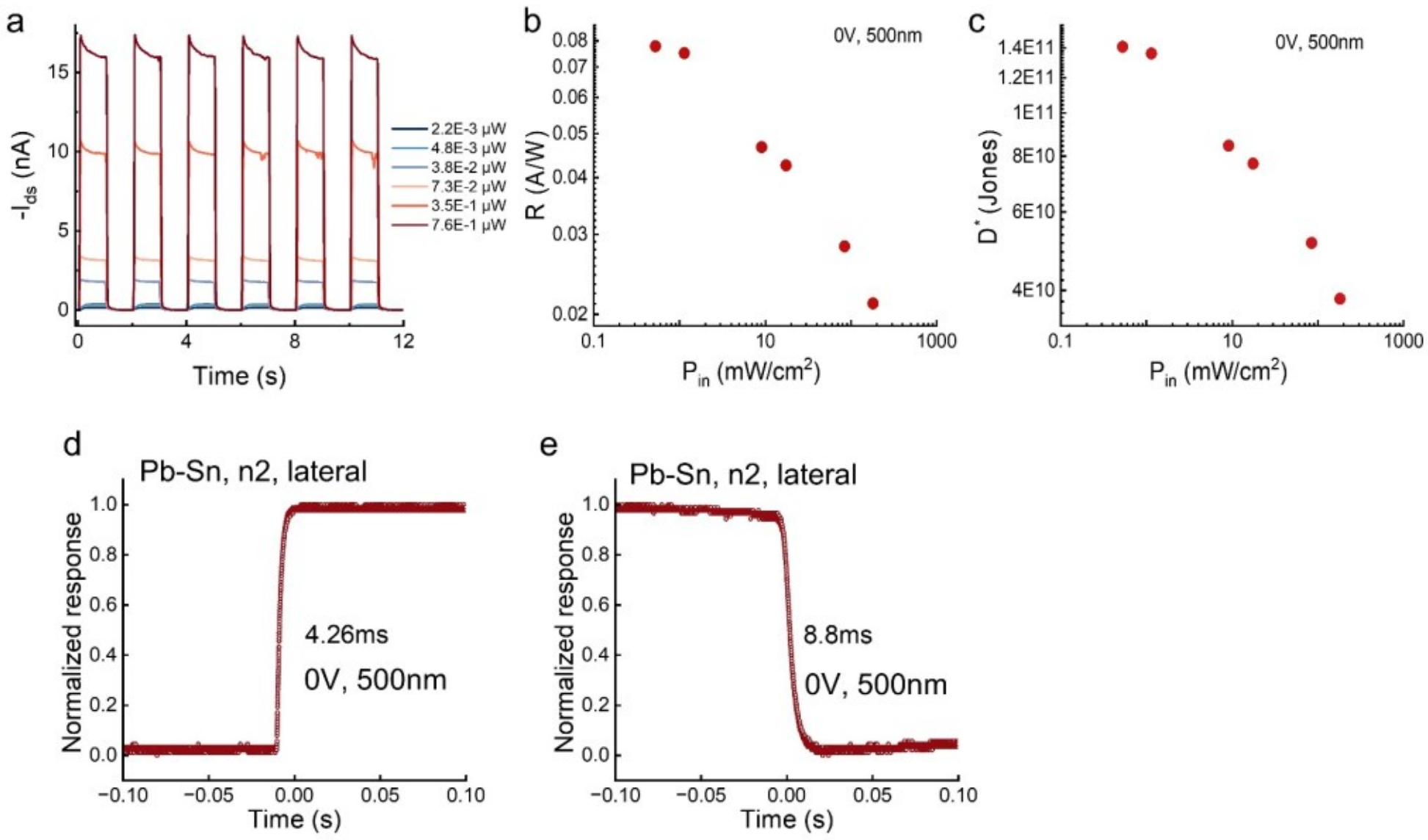

Supplementary Fig. 54. Photodetection performance of Pb-Sn, n2 lateral heterostructure under 500 nm illumination at zero bias. a. Photocurrent switching performance of the Pb-Sn, n2 lateral heterostructure photodetector under different light intensities (2.2×10^{-3} to 7.6×10^{-1} μW, device channel area: 420 μm^2). The bias voltage is 0 V. **b, c.** The dependence of R and D^* on irradiation power intensity (P_{in}) at bias voltage of 0 V. **d, e.** The response times of Pb-Sn, n2 lateral heterostructure. The bias voltage is 0 V.

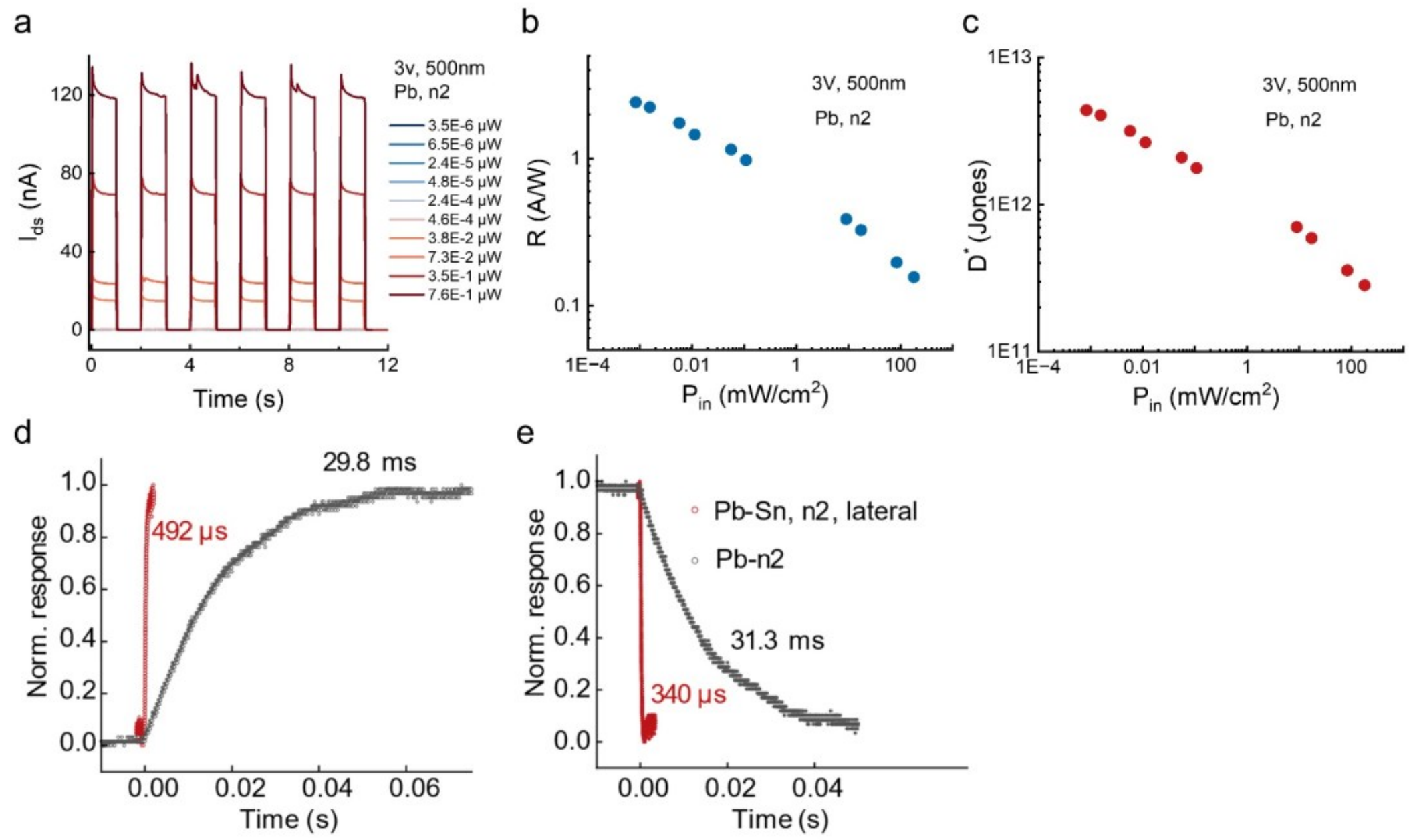

Supplementary Fig. 55. Photodetection performance of Pb-n2 under 500 nm illumination with bias voltage of 3 V. a. Photocurrent switching performance of the Pb-n2 photodetector under different light intensities (3.5×10^{-6} to 7.6×10^{-1} μW, device channel area: 420 μm^2). **b, c.** The dependence of R and D^* on P_{in} at bias voltage of 3 V. **d, e.** Response times of two-terminal photodetectors based on Pb-Sn, n2 lateral heterostructure compared with that from Pb-n2 single crystal.

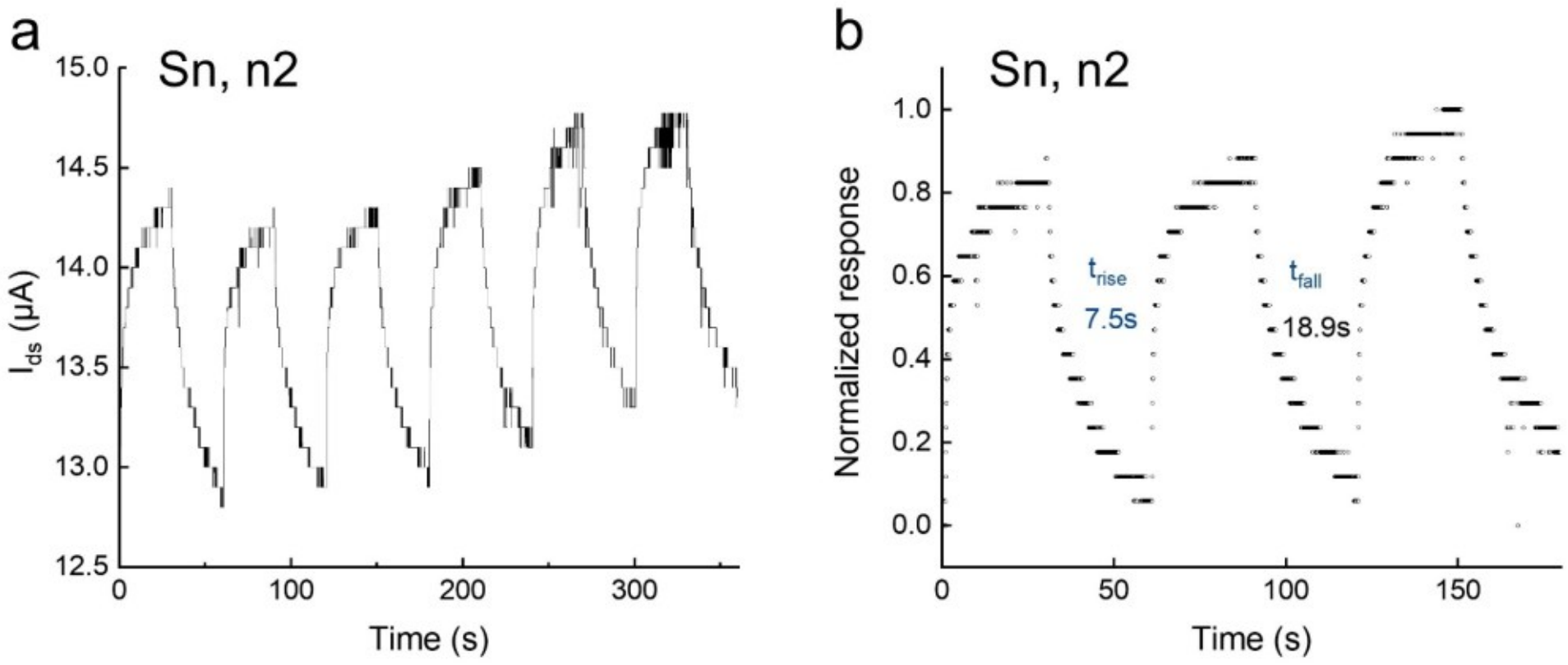

Supplementary Fig. 56. Photodetection performance of Sn-n2 under 500 nm illumination with bias voltage of 3 V. a. Photocurrent switching performance of the Sn-n2 photodetector with light intensity at 4.8×10^{-3} μW (device channel area: 420 μm^2). **b.** The response times of Sn-n2 photodetector.

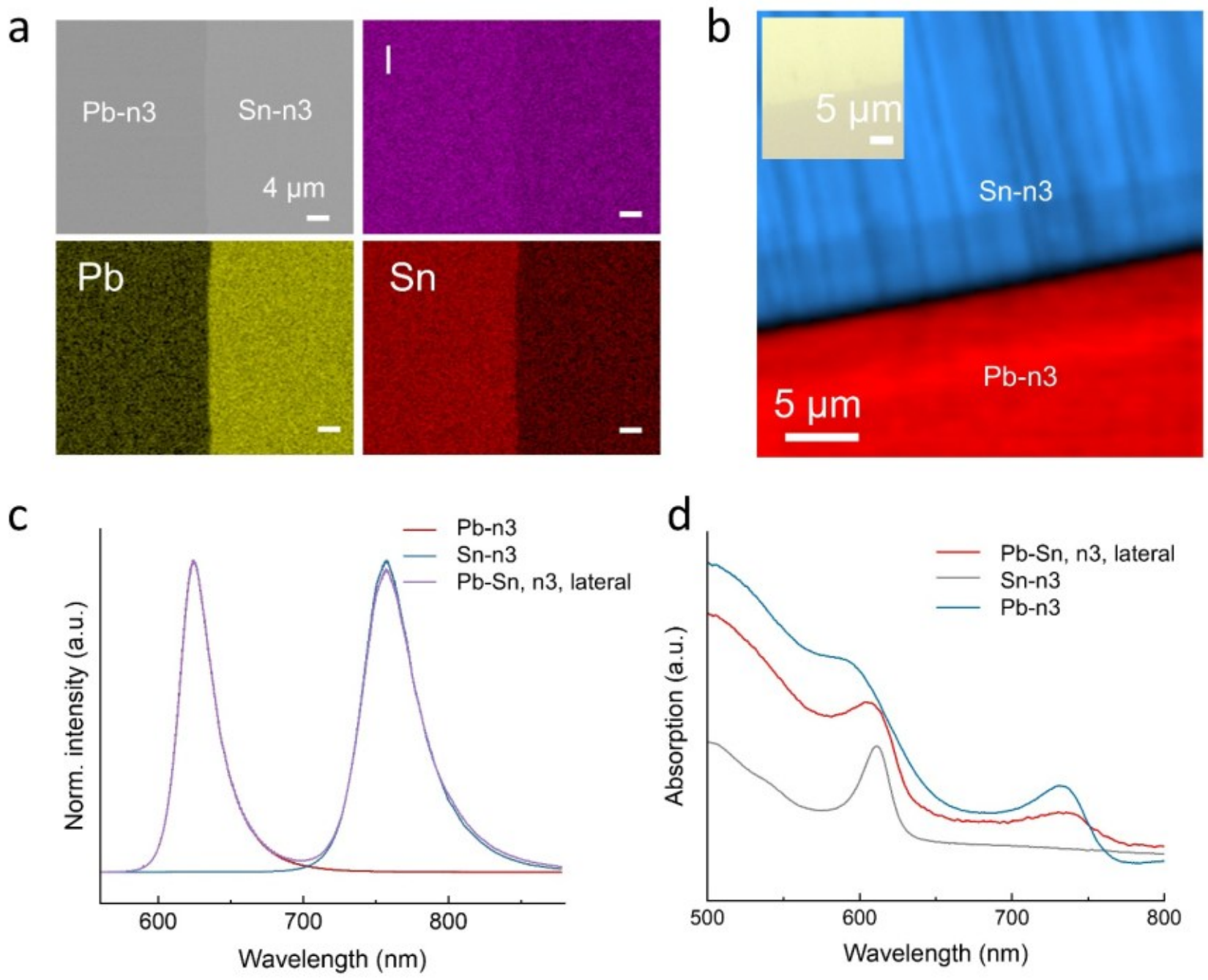

Supplementary Fig. 57. The interface characterization of Pb-Sn, n3 lateral heterostructure. a. The SEM image and EDS elemental mappings (I, Pb and Sn) of Pb-Sn, n3 lateral heterostructure. **b.** The confocal PL mapping of the lateral epitaxial heterostructure. The top side (blue) corresponds to Sn-n3 (collected between 708 nm and 808 nm), and the bottom side (red) corresponds to Pb-n3 (collected between 575 nm and 675 nm). Inset represents the optical image. **c-d.** PL and UV-Vis spectra of Pb-Sn, n3 lateral heterostructure.

Supplementary Tables 1-8

Supplementary Table 1. | Raw mass data and PbI_2 utilization calculations for PEA_2PbI_4 crystal growth through NEIT method.

	Precursors of Pb-n1, NEIT			Undissolved PbI_2 (mg)	Dissolved PbI_2 (mg)	PEA_2PbI_4 mass (mg)	Equivalent PbI_2 in crystal (mg)	PbI_2 Utilization (%)
	PEAI (mg)	PbI_2 (mg)	MeOH (mL)					
1	700	461	5	269.9	191.1	356.4	171.3	89.7
2	700	461	5	282	179	327.2	157.3	87.9

Supplementary Table 2. | Raw mass data and PbI_2 utilization calculations for PEA_2PbI_4 crystal growth through SCS method.

	Precursors of Pb-n1, SCS			PEA_2PbI_4 mass (mg)	Equivalent PbI_2 in crystal (mg)	PbI_2 Utilization (%)
	PEAI (mg)	PbI_2 (mg)	$HI+H_3PO_2$ (mL)			
1	300	1700	9+1	458.6	220.5	13.0
2	300	1700	9+1	479.4	230.5	13.6
3	300	1700	9+1	419.9	201.8	11.9

Supplementary Table 3. Weight and volume of individual crystals (Pb-n2) grown from NEIT solutions of varying volumes.

Volume of solution (mL)	Weight of crystal (g)	Density of crystal (g/cm^3)	Volume of crystal (mm^3)
3	0.1297	2.998	43.3
4	0.1958	2.998	65.3
6	0.2687	2.998	89.6
7	0.3049	2.998	101.7
10	0.4275	2.998	142.6

Supplementary Table 4. Crystal data and structure refinement for PEA_2PbI_4 synthesized via NEIT growth.

	PEA_2PbI_4
Crystal data	
Chemical formula	C16 H24 I4 N2 Pb
M_r	959.16
Crystal system, space group	Triclinic, P1
Temperature (K)	300.00 K
a, b, c (Å)	8.7469(8), 8.7497(7), 16.6843(14)
α, β, γ (°)	95.205(3), 99.759(3), 90.388(3)
V (Å^3)	1252.91(19)
Z	2
F (000)	856
D_x (Mg m^{-3})	2.542
Radiation type	Mo $K\alpha$
No. of reflections for cell measurement	9911
θ range (°) for cell measurement	2.48 to 27.52
μ (mm^{-1})	11.662
Crystal shape	Plate
Colour	Yellow
Data collection	
Diffractometer	Bruker AXS D8 Venture<br>diffractometer with Photon III charge-integrating pixel array detector (CPAD)
Radiation source	High-intensity diamond-Cu/Mo hybrid dual-microfocal X-ray light source
Photon III charge-integrating pixel array detector	10 * 14 cm^2
Scan method	Omega and phi scans
Absorption correction	Multi-scan

	SADABS 2016/2: Krause, L., Herbst-Irmer, R., Sheldrick G.M. & Stalke D. (2015). J. Appl. Cryst. 48, 3-10.
T_{min}, T_{max}	0.3565, 0.7456
No. of measured, independent and observed [$I > 2s(I)$] reflections	36248, 11191, 10509
R_{int}	0.0430
θ values (°)	θ_{max} = 27.544, θ_{min} = 2.338
$(\sin \theta/\lambda)_{max}$ (Å^{-1})	0.714
Range of *h*, *k*, *l*	-11<=h<=11, -11<=k<=11, -21<=l<=21
Refinement	
Refinement on	F^2
$R[F^2 > 2\sigma(F^2)]$, $wR(F^2)$, S	0.0322,0.0808, 1.094
No. of reflections	11191
No. of parameters	372
No. of restraints	230
H-atom treatment	H-atom parameters constrained
Weighting scheme	$w = 1/[\sigma^2(F_o^2) +(0.0425P)^2+ 0.6386P]$ where $P = (F_o^2 + 2F_c^2)/3$
$(\Delta/\sigma)_{max}$	0.001
$\Delta\rho_{max}$, $\Delta\rho_{min}$ (e Å^{-3})	1.557 and -1.271
Absolute structure parameter	0.502(7)

The crystal data and structure refinement for PEA_2PbI_4 synthesized via NEIT and SCS methods exhibit negligible differences.

Supplementary Table 5. Crystal data and structure refinement for $PEA_2MAPb_2I_7$ synthesized via NEIT growth.

	$PEA_2MAPb_2I_7$
Crystal data	
Chemical formula	C17 H30 I7 N3 Pb2
M_r	1579.12
Crystal system, space group	Triclinic, P1
Temperature (K)	303.00 K
a, *b*, *c* (Å)	8.8036(4), 8.8033(3), 22.8002(10)
α, β, γ (°)	94.0225(16), 97.0346(17), 90.1994(15)
V (Å^3)	1749.28(13)
Z	2
F (000)	1376
D_x (Mg m^{-3})	2.998
Radiation type	Mo $K\alpha$
No. of reflections for cell measurement	9841
θ range (°) for cell measurement	2.71 to 27.47
μ (mm^{-1})	15.799
Crystal shape	Plate
Colour	Red
Data collection	
Diffractometer	Bruker AXS D8 Venture diffractometer with Photon III charge-integrating pixel array detector (CPAD)
Radiation source	High-intensity diamond-Cu/Mo hybrid dual-microfocal X-ray light source
Photon III charge-integrating pixel array detector	10 * 14 cm^2
Scan method	Omega and phi scans
Absorption correction	Multi-scan

	SADABS 2016/2: Krause, L., Herbst-Irmer, R., Sheldrick G.M. & Stalke D. (2015). J. Appl. Cryst. 48, 3-10.
T_{min}, T_{max}	0.4976, 0.7456
No. of measured, independent and observed [$I > 2s(I)$] reflections	88038, 16010, 12662
R_{int}	0.0634
θ values (°)	θ_{max} = 27.496, θ_{min} =2.319
$(\sin \theta/\lambda)_{max}$ ($Å^{-1}$)	0.714
Range of *h*, *k*, *l*	-11<=h<=11, -11<=k<=11, -29<=l<=29
Refinement	
Refinement on	F^2
$R[F^2 > 2\sigma(F^2)]$, $wR(F^2)$, S	0.0344, 0.0884, 1.072
No. of reflections	16010
No. of parameters	484
No. of restraints	461
H-atom treatment	H-atom parameters constrained
Weighting scheme	$w = 1/[\sigma^2(F_o^2) +(0.0558P)^2+37.5978P]$<br>where $P = (F_o^2 + 2F_c^2)/3$
$(\Delta/\sigma)_{max}$	0.001
$\Delta\rho_{max}$, $\Delta\rho_{min}$ (e $Å^{-3}$)	1.198 and -1.634
Absolute structure parameter	0.379(9)

The crystal data and structure refinement for $PEA_2MAPb_2I_7$ synthesized via NEIT and SCS methods exhibit negligible differences.

Supplementary Table 6. Crystal data and structure refinement for $PEA_2MA_2Pb_3I_{10}$ synthesized via NEIT growth.

	$PEA_2MA_2Pb_3I_{10}$
Crystal data	
Chemical formula	C18 H36 I10 N4 Pb3
M_r	2199.08
Crystal system, space group	Triclinic, P1
Temperature (K)	298.00 K
a, *b*, *c* (Å)	8.8180(5), 8.8206(5), 29.0416(16)
α, β, γ (°)	95.586(2), 93.207(2), 90.1422(19)
V (Å^3)	2244.5(2)
Z	2
F (000)	1896
D_x (Mg m^{-3})	3.254
Radiation type	Mo *K*α
No. of reflections for cell measurement	9639
θ range (°) for cell measurement	2.82 to 27.42
μ (mm^{-1})	18.116
Crystal shape	Plate
Colour	Black
Data collection	
Diffractometer	Bruker AXS D8 Venture<br>diffractometer with Photon III charge-integrating pixel array detector (CPAD)
Radiation source	High-intensity diamond-Cu/Mo hybrid dual-microfocal X-ray light source
Photon III charge-integrating pixel array detector	10 * 14 cm^2
Scan method	Omega and phi scans
Absorption correction	Multi-scan

	SADABS 2016/2: Krause, L., Herbst-Irmer, R., Sheldrick G.M. & Stalke D. (2015). J. Appl. Cryst. 48, 3-10.
T_{min}, T_{max}	0.5156, 0.7456
No. of measured, independent and observed [$I > 2s(I)$] reflections	104812, 14420, 10705
R_{int}	0.0810
θ values (°)	θ_{max} = 24.712, θ_{min} = 2.313
$(\sin \theta/\lambda)_{max}$ (Å^{-1})	0.714
Range of *h*, *k*, *l*	-10<=h<=10, -10<=k<=10, -34<=l<=34
Refinement	
Refinement on	F^2
$R[F^2 > 2\sigma(F^2)]$, $wR(F^2)$, S	0.0595, 0.1375, 1.202
No. of reflections	14420
No. of parameters	596
No. of restraints	359
H-atom treatment	H-atom parameters constrained
Weighting scheme	$w = 1/[\sigma^2(F_o^2) +(0.0558P)^2+37.5978P]$<br>where $P = (F_o^2 + 2F_c^2)/3$
$(\Delta/\sigma)_{max}$	0.001
$\Delta\rho_{max}$, $\Delta\rho_{min}$ (e Å^{-3})	2.548 and -1.200
Absolute structure parameter	0.407(14)

The crystal data and structure refinement for $PEA_2MA_2Pb_3I_{10}$ synthesized via NEIT and SCS methods exhibit negligible differences.

Supplementary Table 7. Lifetime and diffusion constant (D) of 2D perovskites.

	Lifetime (ns)	Diffusion length (μm)	Reference
BA_2PbI_4	2.2	0.17	[5]
$BA_2MAPb_2I_7$	4.3	0.25	
$BA_2MA_2Pb_3I_{10}$	3.3	0.32	
$BA_2MA_3Pb_4I_{13}$	5.0	0.5	
$BA_2MA_4Pb_5I_{16}$	6.7	0.68	
BA_2PbI_4	-	0.05	[6]
$BA_2MAPb_2I_7$	-	0.06	
$BA_2MA_2Pb_3I_{10}$	-	0.10	
PEA_2PbI_4	-	0.23	
$PEA_2MAPb_2I_7$	-	0.09	
$PEA_2MA_2Pb_3I_{10}$	-	0.14	
mF-PEA_2PbI_4	-	0.41	
PEA_2PbI_4	28±7	-	[7]
$PEA_2MAPb_2I_7$	35±4	-	
$PEA_2MA_2Pb_3I_{10}$	6.7±0.8	-	
$PEA_2MA_3Pb_4I_{13}$	80±10	-	

Supplementary Table 8. Crystal data and structure refinement for $PEA_2FAPb_2I_7$ synthesized via NEIT growth.

	$PEA_2FAPb_2I_7$
Crystal data	
Chemical formula	C17 H30 I7 N4 Pb2
M_r	1593.13
Crystal system, space group	Triclinic, P1
Temperature (K)	300.00 K
a, b, c (Å)	8.8041(6), 8.8052(5), 22.7997(14)
α, β, γ (°)	97.051(2), 94.010(3), 90.186(2)
V (Å^3)	1749.69(19)
Z	2
F (000)	1390
D_x (Mg m^{-3})	3.024
Radiation type	Mo $K\alpha$
No. of reflections for cell measurement	9972
θ range (°) for cell measurement	2.71 to 27.50
μ (mm^{-1})	15.797
Crystal shape	Plate
Colour	Red
Data collection	
Diffractometer	Bruker AXS D8 Venture<br>diffractometer with Photon III charge-integrating pixel array detector (CPAD)
Radiation source	High-intensity diamond-Cu/Mo hybrid dual-microfocal X-ray light source
Photon III charge-integrating pixel array detector	10 * 14 cm^2
Scan method	Omega and phi scans
Absorption correction	Multi-scan

	SADABS 2016/2: Krause, L., Herbst-Irmer, R., Sheldrick G.M. & Stalke D. (2015). J. Appl. Cryst. 48, 3-10.
T_{min}, T_{max}	0.4993, 0.7456
No. of measured, independent and observed [$I > 2s(I)$] reflections	44135, 11648, 9250
R_{int}	0.0583
θ values (°)	θ_{max} = 24.711, θ_{min} = 2.319
$(\sin \theta/\lambda)_{max}$ (Å^{-1})	0.714
Range of *h*, *k*, *l*	-10<=h<=10, -10<=k<=10, -26<=l<=26
Refinement	
Refinement on	F^2
$R[F^2 > 2\sigma(F^2)]$, $wR(F^2)$, S	0.0469, 0.1513, 0.969
No. of reflections	11648
No. of parameters	543
No. of restraints	542
H-atom treatment	H-atom parameters constrained
Weighting scheme	$w = 1/[\sigma^2(F_o^2) +(0.0558P)^2+37.5978P]$<br>where $P = (F_o^2 + 2F_c^2)/3$
$(\Delta/\sigma)_{max}$	0.001
$\Delta\rho_{max}$, $\Delta\rho_{min}$ (e Å^{-3})	2.063 and -2.166
Absolute structure parameter	0.498(14)

Supplementary References

1 Roy, S. *et al.* Measurement of quantum yields of monolayer TMDs using dye-dispersed PMMA thin films. *Nanomaterials* **10**, 1032 (2020).

2 Walters, G. *et al.* Directional light emission from layered metal halide perovskite crystals. *J. Phys. Chem. Lett.* **11**, 3458-3465 (2020).

3 Qi, Y. *et al.* Anisotropic emission of orientation-controlled mixed-dimensional perovskites for light-emitting devices. *J. Materiomics.* **9**, 762-767 (2023).

4 Sidhik, S. *et al.* Memory seeds enable high structural phase purity in 2D perovskite films for high-efficiency devices. *Adv. Mater.* **33**, 2007176 (2021).

5 Deng, S. *et al.* Long-range exciton transport and slow annihilation in two-dimensional hybrid perovskites. *Nat. Commun.* **11**, 664 (2020).

6 Xiao, X. *et al.* Ultrafast exciton transport with a long diffusion length in layered perovskites with organic cation functionalization. *Adv. Mater.* **32**, 2004080 (2020).

7 Delport, G. *et al.* Exciton–Exciton annihilation in two-dimensional halide perovskites at room temperature. *J. Phys. Chem. Lett.* **10**, 5153-5159 (2019).
