# Supplementary material for: Living growth of ultra-bright 2D perovskites with long-lived carriers
Source: Nat Commun. 2026 Jul 14;17:8629. doi: 10.1038/s41467-026-75434-6 (PMC13487158; doi:10.1038/s41467-026-75434-6)
Supplement: Supplementary file 2 [file 41467_2026_75434_MOESM2_ESM.pdf]

Description of additional Supplementary files

Supplementary Movie 1: In-situ monitoring of crystal growth at constant RT in NEIT, showing optical images captured over 50 minutes (recorded under a 5× objective, sped up 100×).

Supplementary Movie 2: In situ observation of the epitaxial growth process of Pb-Sn, n2 heterostructures under a 5× objective, sped up 10×.
