# Supplementary material for: Living growth of ultra-bright 2D perovskites with long-lived carriers
Source: Nat Commun. 2026 Jul 14;17:8629. doi: 10.1038/s41467-026-75434-6 (PMC13487158; doi:10.1038/s41467-026-75434-6)
Supplement: Supplementary file 3 [file 41467_2026_75434_MOESM5_ESM.pdf]

nature research

Corresponding author(s): *Double-blind peer review submissions: write DBPR and your manuscript number here instead of author names.*

Lasing Reporting Summary

Nature Research wishes to improve the reproducibility of the work that we publish. This form is intended for publication with all accepted papers reporting claims of lasing and provides structure for consistency and transparency in reporting. Some list items might not apply to an individual manuscript, but all fields must be completed for clarity.

For further information on Nature Research policies, including our data availability policy, see Authors & Referees.

Experimental design

Please check: are the following details reported in the manuscript?

1. Threshold

Plots of device output power versus pump power over a wide range of values indicating a clear threshold

☒ Yes ☐ No — Fig.1 h-j and Supplementary Fig. 27

2. Linewidth narrowing

Plots of spectral power density for the emission at pump powers below, around, and above the lasing threshold, indicating a clear linewidth narrowing at threshold

☒ Yes ☐ No — Fig.1 h-j and Supplementary Fig. 27

Resolution of the spectrometer used to make spectral measurements

☒ Yes ☐ No — 0.1141nm

3. Coherent emission

Measurements of the coherence and/or polarization of the emission

☒ Yes ☐ No — Supplementary Figs. 29, 30

4. Beam spatial profile

Image and/or measurement of the spatial shape and profile of the emission, showing a well-defined beam above threshold

☒ Yes ☐ No — Supplementary Fig. 30

5. Operating conditions

Description of the laser and pumping conditions
Continuous-wave, pulsed, temperature of operation

☒ Yes ☐ No — Methods-Lasing measurement

Threshold values provided as density values (e.g. W cm^{-2} or J cm^{-2}) taking into account the area of the device

☒ Yes ☐ No — μJ/cm2, Fig.1 h-j and Supplementary Fig. 27

6. Alternative explanations

Reasoning as to why alternative explanations have been ruled out as responsible for the emission characteristics
e.g. amplified spontaneous, directional scattering; modification of fluorescence spectrum by the cavity

☒ Yes ☐ No — Fig.1 h-j and Supplementary Figs. 26-30

7. Theoretical analysis

Theoretical analysis that ensures that the experimental values measured are realistic and reasonable
e.g. laser threshold, linewidth, cavity gain-loss, efficiency

☒ Yes ☐ No — Fig.1 h-j and Supplementary Figs. 26-30

8. Statistics

Number of devices fabricated and tested

☒ Yes ☐ No — Supplementary Fig. 26

Statistical analysis of the device performance and lifetime (time to failure)

☒ Yes ☐ No — Supplementary Fig. 26

March 2018
