# Supplementary material for: Living growth of ultra-bright 2D perovskites with long-lived carriers
Source: Nat Commun. 2026 Jul 14;17:8629. doi: 10.1038/s41467-026-75434-6 (PMC13487158; doi:10.1038/s41467-026-75434-6)
Supplement: Supplementary file 4 [file 41467_2026_75434_MOESM6_ESM.pdf]

nature portfolio

# Peer Review File

# Living growth of ultra-bright 2D perovskites with long-lived carriers

Corresponding Author: Professor Enzheng Shi

**This file contains all reviewer reports in order by version, followed by all author rebuttals in order by version.**

Version 0:

Reviewer comments:

Reviewer #1

(Remarks to the Author)
The paper describes a new method of crystal growth method capable of avoiding the pitfalls of traditional 2D perovskite crystal growth methods based on slow-cooling, such as low photoluminescence quantum yield (PLQY), short charge carrier lifetimes, and short diffusion lengths. The study characterizes properties which defines crystal quality produced using the authors' Near-Equilibrium Isothermal (NEIT) strategy, such as physical dimensions, photophysical properties and crystallographic fidelity, and compare these characteristics to crystals grown via slow-cooling synthesis. The crystal growth control is interesting for the synthesis of 2D perovskite large bulk crystals. However, the broad impact of the current results can be improved. A few directions are suggested below:

1. While the NEIT strategy can enhance the quality of 2D perovskites grown, the claim of its living character should be challenged. Crystal growth is always a living process, including the traditional SCS methods for 2D perovskite using mineral acids. While the NEIT method emphasizes supersaturation control, similar supersaturation control can also be implemented in slow-cooling. (i.e. as long as the crystal is not severely contaminated on its surface, one can always put it back into a supersaturated solution to promote the growth again).

2. However, the supersaturation control is indeed less explored for 2D perovskite crystal growth. There may be a few additional points worth investigating further:
a) Mechanistically, it is possible to achieve supersaturation control using either mineral acid system or methanol system. However, for the compositions that the authors studied (i.e. PEA, Pb, MA, I), is it much easier to reach supersaturation in methanol versus mineral acid, and what is the reason for that?
b) Using the NEIT method, is it possible to increase the reproducibility of the final crystal size? For instance, would it be possible to tailor the final dimensions of the crystal by controlling temperature gradient or supersaturation degree?

3. The demonstration of lasing is interesting from exfoliated flakes is interesting but has several limitations. First, while the overall improvement of carrier lifetime and diffusion length did support the much higher crystal quality grown by NEIT method, since exfoliation method is implemented, it is not impossible to find an exfoliated crystal from the SCS method that can exhibit optically pumped lasing. Second, the lasing experiments were conducted at 80K, while the possibility of room-temperature optically pumped lasing using n=1 2D perovskites bulk crystals has been demonstrated back in 2018 (Nano Letters, 2018, 18(5), 3221–3228). One angle to improve the authors' results is to study the RT lasing using as-grown bulk crystals since the NEIT-grown crystals seem to have much higher structural and cavity quality. If dimensional control is possible, then RT lasing can be controlled.

Additional, a few minor issues can be better clarified.
1. It would be a good idea to present shortly how the utilization rates of $PbI_2$ are calculated for both techniques used for comparison in the paper, as the numbers used for the SCS method seem lower than the NEIT method. In particular, the utilization rates of $PbI_2$ for NEIT synthesis can be accurately obtained by calculating the ratio of Pb precursor in the crystal and in the precursor down to the decimal place while that is not the case for the SCS method.

2. The SCLC measurement, data processing, and reproducibility should be better clarified. According to Blakesley et al. (2014), control factors such as film thickness and electrode quality can impact the measurement of trapped density, up to a

factor of 3. Corre (2024) also highlighted a variety of potential problems with SCLC measurements such as the presence of mobile charges and contact asymmetry in perovskites.
https://pubs.acs.org/doi/10.1021/acs.jpclett.4c02379
https://pubs.acs.org/doi/10.1021/acsenergylett.9b02720

Reviewer #2

(Remarks to the Author)
Han et al. described a near-equilibrium isothermal growth strategy to improve the quality of 2D metal halide perovskites. The crystals are cm in size and can be applied to several compositions. The crystals demonstrated improved optoelectronic properties, including high PLQY and reported cavity-free lasing. Furthermore, the near-equilibrium growth approach allows formation of vdWs or lateral epitaxial heterostructures. The new structure could broaden potential applications. The results are significant, but several claims and analysis require closer scrutiny and greater scientific rigorous before the manuscript can be considered for acceptance.

1. The authors described the single crystal growth process as near-equilibrium isothermal growth, but additional explanation is required. Why was MeOH chosen as the solvent instead of other candidates? The manuscript also lacks the key explanation for why there is only one nuclei per vial. From the methods section, only two batches of samples were grown using the NEIT route. More statistical evidence of the single nucleation behavior should be provided.
2. In Supplementary Fig.15, no crystals are visible in Day4 picture, but a centimeter sized crystal is shown in the next photo at Day9. The authors should provide more information on the intermediate stage.
3. The authors provided rocking curve and GIXRD results to demonstrate improved crystallinity:
-The GIXRD results appear significantly flawed and can be excluded, along with the claim of a 40% reduction in residue strain. Specifically, the Bragg peak at ~4deg decreased by 0.16deg when sampling from surface to bulk, which would correspond to an implausibly large (a few percent) strain for a bulk single crystal. Alternatively, the authors could exfoliate the surface layer and measured XRD directly of the bulk region to confirm this strain. A more likely explanation is measurement artefact due to misalignment when changing the tilt angle, that caused significant peak shifts at low angles. Also, such big residue strain is not consistent with the narrow FWHM from rocking curve measurements.
-The rocking curve results alone support improved crystallinity. However, the author should make sure that difference in surface roughness is accounted for rocking curve broadening. X-ray reflectivity should provide indication of the surface roughness and can be measured on the same sample using the same instrument. Ideally, exfoliated samples should be used to maintain a flat surface.
4. The measurement of 2D perovskite PLQY was not performed in an integration sphere.
-Did the authors consider the anisotropic emission commonly observed in 2D perovskites? Since the optic setup does not collect emission from all directions, assuming a Lambertian profile (which is unclear in the current manuscript) may introduce big error? Back focal plane imaging or angle-resolved PL measurement should confirm the emission profile.
-In addition, what is the sample thickness used for the PLQY measurement? For cm-thick crystals, how much transmission signal is still there for 450nm excitation? Thick crystals exhibit strong reabsorption effect, therefore, even the reference sample reported as 55% measured PLQY could have a much larger internal quantum efficiency, which will lower down the claimed difference.
5. On page 7: the authors attributed the claimed lasing to reduced trap density. However, as the authors stated, Auger recombination, rather than trap-mediated non-radiative recombination, is the main limitation. Under such high power excitation, traps are likely saturated, so attributing threshold behavior primarily to trap density is not justified. Instead, low lasing threshold would be strongly affected by unintentional cavity effects. Thickness of the sample measured should be reported.
-The authors claimed lasing, rather than alternative explanations such as ASE or random lasing, by narrowed peaks, polarization measurements. However, since the 2D perovskite is anisotropic material, polarization is not enough to conclusively establish lasing. Additional evidence of coherence and a feedback mechanism/mode structure is required to refer it as lasing.
-The authors reported FWHM of 0.18 nm. From Figure 1h, it seems the redder part has an overall Gaussian-like shape with FWHM ~2nm, and many periodic fringing features. Perhaps the fringes are where the authors derived the FWHM 0.18nm from. Are the fringes (similarly in Fig. 1h and Fig. S6) an artefact of the spectrometer when averaging over multiple pulses, or from interference in the optical path including windows of the cryostat? By removing the fringes, the feature could be more sensibly explained by ASE, since there was no designed cavity.
7. Figure 4e-f are hard to follow and the authors should label the crystal orientation. Figure 4e seems to be cross-section of the vdWs heterostructure. How was the cross-section section prepared? In line 388 the authors also mentioned SEM of lateral heterostructure but no figures are referred to. In figure S20b, the EDS spectra for the Pb and Sn regions should be shown to confirm that they are not alloyed.
8. There is a claimed lattice mismatch of <1.6% between the Pb-Sn lateral heterojunction. The author should explain how the heterostructure accommodate the high mismatch without cracking or significant defects that affect photodetector performance.
9. The authors reported vdWs epitaxy and lateral epitaxy in the heterostructure. More crystallographic evidence is needed to support the aligned in-plane orientation. A single peak of (110) is shown for the vdWs heterostructure but not for lateral heterostructure. Importantly, the single (110) peak cannot exclude certain misoriention when the Bragg peak from the second crystal could be rotated such that they are not accessible in the current 1D line scan geometry. Reciprocal space maps showing in-plane signals from both layers can confirm epitaxy since the two layers do have different lattice parameters.

Format and terminology:

The manuscript lacks sufficient methodological detail for reproducibility of characterizations. For example, rocking curve measurements including beam size, slit settings and beam optics; accelerating voltage for SEM and EDS; excitation power of PLQY, device geometry of the SCLC electron-only device; etc.
In Supplementary Fig. 1, enlarging a crystal should be described as seeded growth, not epitaxial growth.
On page 2 line 61, suggest change ‘bright’ to ‘efficient’. Confined perovskite systems are advantageous in efficient LEDs, but to achieve high brightness, these systems (and the reference cited) are less competitive in maintaining high efficiency at high current density.
Supplementary Fig.4b, the y-axis label should not be PL intensity if the measurement is reflection and transmission.
Supplementary Fig 22c, the reported linear dynamic range is not supported. In linear dynamic range, the photoresponsivity is constant against input power, instead here, photoresponsivity decreased from the very beginning.

Reviewer #3

(Remarks to the Author)
This work reports a near-equilibrium isothermal (NEIT) growth strategy for two-dimensional perovskite single crystals, which achieves ultra-low nucleation density and produces centimeter-sized single crystals of $PEA_2PbI_4$ (Pb-n1), $PEA_2MAPb_2I_7$ (Pb-n2), and $PEA_2MA_2Pb_3I_{10}$ (Pb-n3). The NEIT strategy establishes a versatile and scalable platform for synthesizing high-quality, large-area 2D perovskite single crystals and their heterostructures, representing a significant advance in the material foundation for quantum-confined semiconductor devices. The findings are compelling and the methodology is highly innovative. However, before the acceptance in Nature Communications, the authors are required to make minor revisions to address the following comments to further strengthen the rigor and impact of the work.

1. In Fig. 1g, the PLQY is plotted on a normalized scale (0–1), while the text reports values as percentages (e.g., 95 ± 2%). The y-axis of Fig. 1g should be relabeled to 0–100% to maintain consistency and avoid reader confusion.
2. The Pb-n2 crystals grown by the NEIT method exhibit high transparency in the presented images. As optical transparency correlates directly with crystalline quality and long-range uniformity, it is highly recommended to provide a quantitative comparison of optical clarity, for example via transmittance spectroscopy, between crystals synthesized using the NEIT and SCS approaches.
3. In Fig. 1e, the FWHM for the (001) diffraction peak is labeled, but the corresponding value for the (110) peak is absent. For completeness, the (110) peak FWHM should also be indicated in the figure or caption.
4. In Fig. 2, the carrier lifetime of the Pb-n1, n2 and n3 single crystals synthesized from NEIT and SCS approaches are presented as single measurements for each crystal. Reporting results from multiple samples or repeated measurements would enhance the statistical reliability of these data.
5. A discrepancy is noted between the lifetime of Pb-n2 reported in Fig. 2b (151.9 ns) and that from FLIM in Fig. 2e (66.7 ns). The origin of this difference, whether due to measurement conditions or regional variation, should be clarified and discussed.

Version 1:

Reviewer comments:

Reviewer #1

(Remarks to the Author)
The response letter is well-organized, scientifically detailed, and addresses most reviewer concerns substantively. Particularly, DLS tracking experiments are highly persuasive evidence for the metastable zone difference between HI and MeOH systems. One remaining issue to address is that authors should try to capture the gradual increase of particle size under DLS (during cooling in SLS method and during steady growth at RT in MeOH system.) The current DLS results only capture the beginning and the end.

Additionally, SI Fig 22 "ump fluence" should be "pump fluence"

Reviewer #2

(Remarks to the Author)
The authors have done a considerable effort to improve the manuscript to be published in the journal. I have three questions prior to recommend for publication:
(1) The authors showed angular-resolved PL is uniform for the 2D perovskite single crystal. I appreciate the experimental validation in Fig. S16, but could the author comment on why the strong structure anisotropy did not lead to optical anisotropy? It has been previously demonstrated both theoretically and experimentally that the emission intensity is angular dependent (stronger in the out-of-plane direction) in 2D perovskites (https://doi.org/10.1021/acs.jpclett.0c00901, https://doi.org/10.1016/j.jmat.2023.01.014). This discrepancy may affect an over-estimated PLQY value.
(2) The authors stated a mismatch value of 1.6% between lateral Pb-Sn epitaxial heterostructure. The claim of that mismatch value ‘is accommodated without cracking or significant defects’ is not substantiated with existing data. It is hard to validate whether small cracks form that are not yet being captured in the current resolution, and characterization of defects is not provided. Moreover, it is less likely that the 1.6% mismatch did not show any impact at all. In the 5% lattice mismatch report the author quoted, the strain can be seen relaxed through ripples and dislocations.
(3) While the mismatch value of 1.6% is quoted, in the (110) plane XRD data (Fig. S42) the two peaks should be expected to have q value difference of 1.6%. As the q value is ~1 A-1, the difference would be 0.01-0.02 A-1. The data in Fig.S42a

shows less than 0.004 A-1 and even less than 0.0005 A-1 q value difference in Fig. S42c. It is therefore unclear whether the second signal belongs to Sn-n2, which shed question on whether the epitaxial relationship is proven. Would the author provide reference pure Sn-n2 samples?

Reviewer #3

(Remarks to the Author)
The authors have adequately addressed my previous concerns and have made appropriate revisions to the manuscript. In my opinion, the manuscript is now suitable for publication in Nature Communications. I therefore recommend acceptance.

Version 2:

Reviewer comments:

Reviewer #1

(Remarks to the Author)
The authors have well address the previous questions.

Reviewer #2

(Remarks to the Author)
The authors have addressed previous questions and improved the manuscript accordingly. I am therefore pleased to recommend acceptance.

**Open Access** This Peer Review File is licensed under a Creative Commons Attribution 4.0 International License, which permits use, sharing, adaptation, distribution and reproduction in any medium or format, as long as you give appropriate credit to the original author(s) and the source, provide a link to the Creative Commons license, and indicate if changes were made.
In cases where reviewers are anonymous, credit should be given to 'Anonymous Referee' and the source.
The images or other third party material in this Peer Review File are included in the article's Creative Commons license, unless indicated otherwise in a credit line to the material. If material is not included in the article's Creative Commons license and your intended use is not permitted by statutory regulation or exceeds the permitted use, you will need to obtain permission directly from the copyright holder.
To view a copy of this license, visit https://creativecommons.org/licenses/by/4.0/

**A List of All Revisions and Responses to Referees' Comments for *Nature Communications* Manuscript: NCOMMS-26-000453-T**

**Note**: The revision is yellow highlighted in the main article. Response to the referees' comments is marked in blue.

## REVIEWER COMMENTS

Reviewer #1 (Remarks to the Author):

The paper describes a new method of crystal growth method capable of avoiding the pitfalls of traditional 2D perovskite crystal growth methods based on slow-cooling, such as low photoluminescence quantum yield (PLQY), short charge carrier lifetimes, and short diffusion lengths. The study characterizes properties which defines crystal quality produced using the authors' Near-Equilibrium Isothermal (NEIT) strategy, such as physical dimensions, photophysical properties and crystallographic fidelity, and compare these characteristics to crystals grown via slow-cooling synthesis. The crystal growth control is interesting for the synthesis of 2D perovskite large bulk crystals. However, the broad impact of the current results can be improved. A few directions are suggested below:

1. While the NEIT strategy can enhance the quality of 2D perovskites grown, the claim of its living character should be challenged. Crystal growth is always a living process, including the traditional SCS methods for 2D perovskite using mineral acids. While the NEIT method emphasizes supersaturation control, similar supersaturation control can also be implemented in slow-cooling. (i.e. as long as the crystal is not severely contaminated on its surface, one can always put it back into a supersaturated solution to promote the growth again).

***Response:*** We appreciate your insightful comment. While a clean crystal can resume growth when reintroduced into a supersaturated solution, it does not necessarily constitute a living process. Borrowing the definition from polymer chemistry, living growth requires continuous propagation with negligible secondary nucleation (*Nature* **1956**, 178, 1168-1169). Therefore, the system that spontaneously forms numerous new nuclei instead of adding to existing seeds is non-living.
Specifically, implementing supersaturation control in traditional slow cooling synthesis (SCS) using aqueous $HI/H_3PO_2$ mixtures is challenging. Dynamic light scattering (DLS) results reveal the high sensitivity of the SCS system to temperature (Fig. R1). Slight cooling (100 °C to 90 °C) triggers precursors to transition from a molecular state (~2

nm) into macroscopic scattering signals (>1000 nm). We attribute this rapid transition to the hydrophobic aggregation of bulky $PEA^+$ cations, which creates abundant heterogeneous nucleation sites, indicating a narrow metastable zone (defined as the concentration region where a solution is supersaturated but spontaneous nucleation does not occur) during dynamic cooling (Crystallization (Fourth Edition), *Elsevier*, **2001**). In addition, introducing a seed crystal into the supersaturated HI solution inevitably introduces thermal fluctuations that disrupt this metastable state, provoking rapid multi-nucleation (Fig. R2).

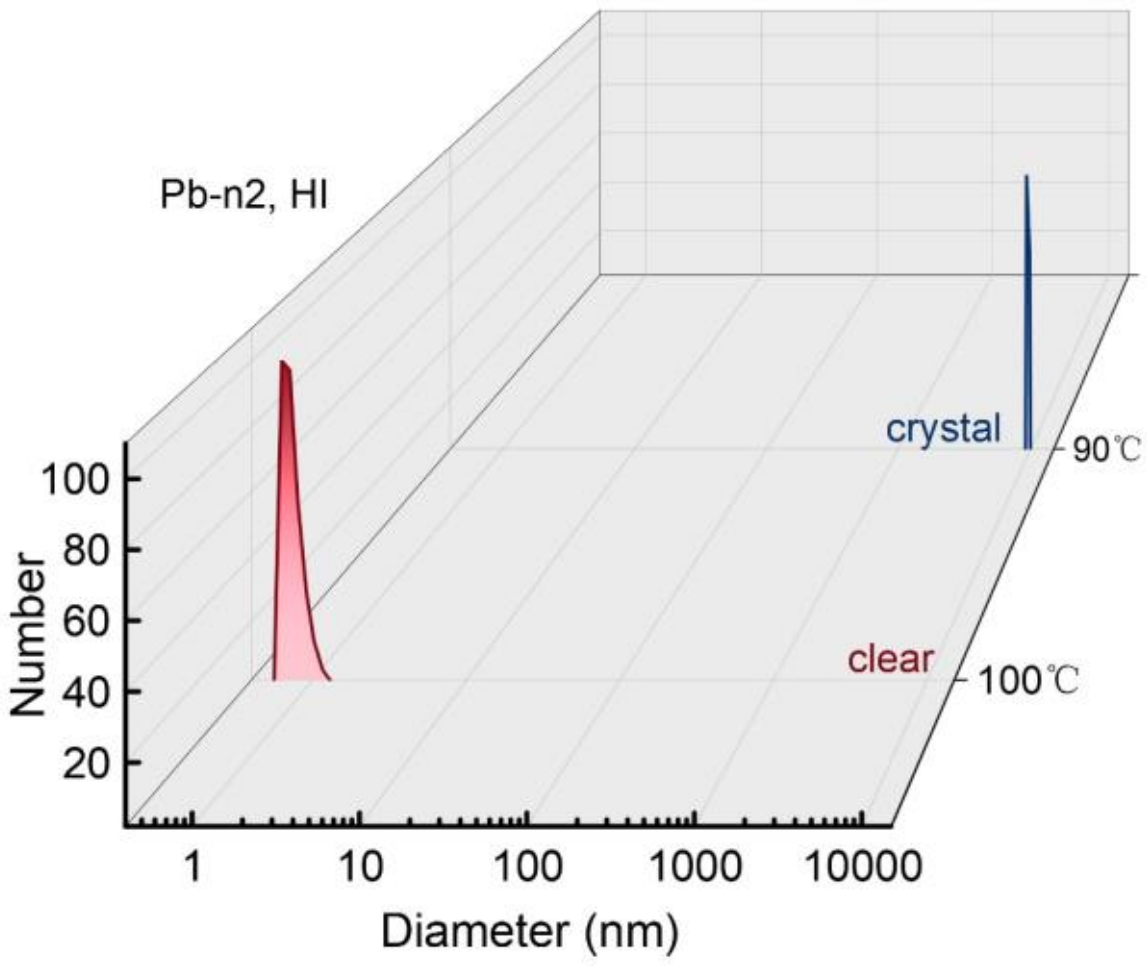


**Fig. R1. Dynamic light scattering (DLS) tracking of precursor solution in the HI system.** Number distribution of the Pb-n2 precursor solution during cooling. A molecular-scale peak (~2 nm) is observed at 100 °C, while cooling to 90 °C triggers the appearance of macroscopic scattering signals (>1000 nm).

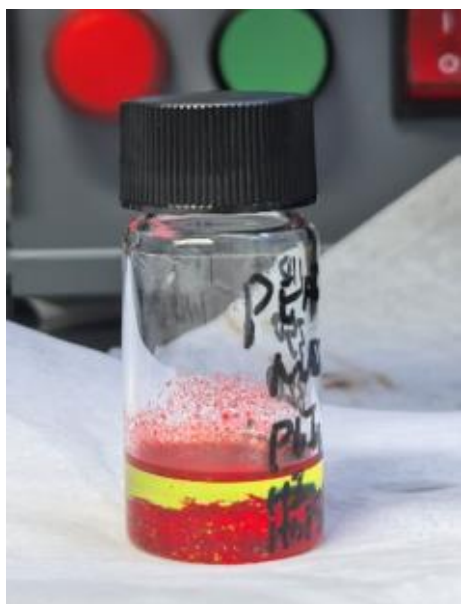

**Fig. R2. The explosive parasitic nucleation when implementing seeded growth in the HI/$H_3PO_2$ SCS system.**

In comparison, the NEIT strategy is operated under isothermal room-temperature (RT) conditions in MeOH. As evidenced by DLS tracking (Fig. R3), the precursor solution maintains a stable molecular state (1-2 nm) for over 22 hours at RT, confirming the absence of large aggregates. This is likely because MeOH solvent with moderate polarity keeps the $PEA^+$ cations extended, which effectively minimizes heterogeneous nucleation sites and directs the system toward a homogeneous nucleation. Since the energy barrier for homogeneous nucleation is significantly higher than that of

heterogeneous nucleation, spontaneous nucleation is effectively inhibited, thereby sustaining a broad metastable zone. Within this zone, once a primary nucleus forms, it acts as the main active growth center (*Science* **2015**, 348, aaa6760). Precursor ions preferentially add to this crystal due to the lower energy barrier, demonstrating living growth characteristics with negligible secondary nucleation.

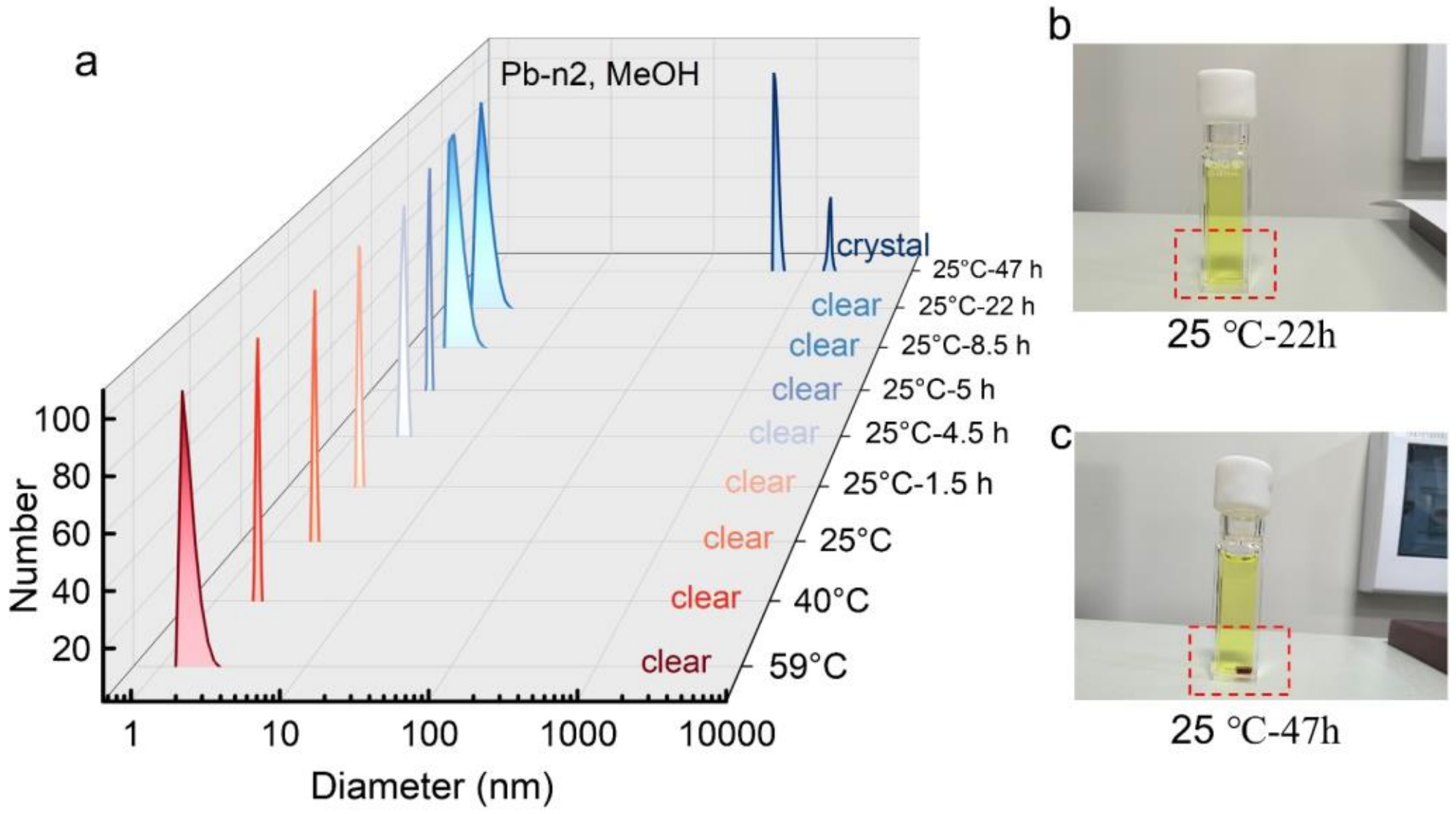


**Fig. R3. DLS tracking and macroscopic observation of the MeOH system. a.** DLS number distribution of the Pb-n2 precursor solution during cooling (59 °C to 25 °C) and RT aging at 25 °C. The profile maintains a peak at ~1-2 nm for over 22 hours. (Note: The initial temperature was set to 59 °C due to the DLS instrument's safety restriction, which must remain below the boiling point of MeOH). **b,** The solution remains visually clear after 22 h at RT. **c,** After 47 h, a crystal at the bottom of the vial.

***Revision:*** Figs. R1 and R3 are incorporated in the revised Supplementary Information as Supplementary Figs. 2 and 5.

The following texts (highlighted in yellow) are included in Line 136 of the revised main manuscript: In the SCS ($HI/H_3PO_2$) system, dynamic light scattering (DLS) spectra reveal that minor cooling triggers the rapid emergence of aggregates. This is likely due to the rapid hydrophobic aggregation of bulky $PEA^+$ cations. Consequently, abundant heterogeneous nucleation sites are generated and the metastable zone is narrowed, driving uncontrolled multi-nucleation[38] (Supplementary Figs. 1, 2).

38. Mullin, J. W. *Crystallization* (Elsevier, 2001).

2. However, the supersaturation control is indeed less explored for 2D perovskite crystal growth. There may be a few additional points worth investigating further:
a) Mechanistically, it is possible to achieve supersaturation control using either mineral acid system or MeOH system. However, for the compositions that the authors studied (i.e. PEA, Pb, MA, I), is it much easier to reach supersaturation in MeOH versus mineral acid, and what is the reason for that? b) Using the NEIT method, is it possible to increase

the reproducibility of the final crystal size? For instance, would it be possible to tailor the final dimensions of the crystal by controlling temperature gradient or supersaturation degree?

***Response:*** We deeply appreciate your insightful mechanistic inquiry.

**a)** Firstly, it is not easier for MeOH system to reach supersaturation than the HI system; rather, it sustains high supersaturation (as evidenced by high Pb utilization rate of ~89%) within a wide metastable zone. This difference originates from distinct solvation behaviors.

In the HI system (SCS method), while $PbI_2$ dissolves easily at room temperature (RT) (Fig. R4a-c), the bulky phenyl ring of $PEA^+$ is highly hydrophobic and exhibits limited solubility at RT (massive macroscopic precipitation at RT) (Fig. R4d-h). As detailed in the response to 1st comment as Fig. R1, the HI system suffers from the rapid hydrophobic aggregation of $PEA^+$ cations, which creates abundant heterogeneous nucleation sites that instantly collapse the metastable zone.

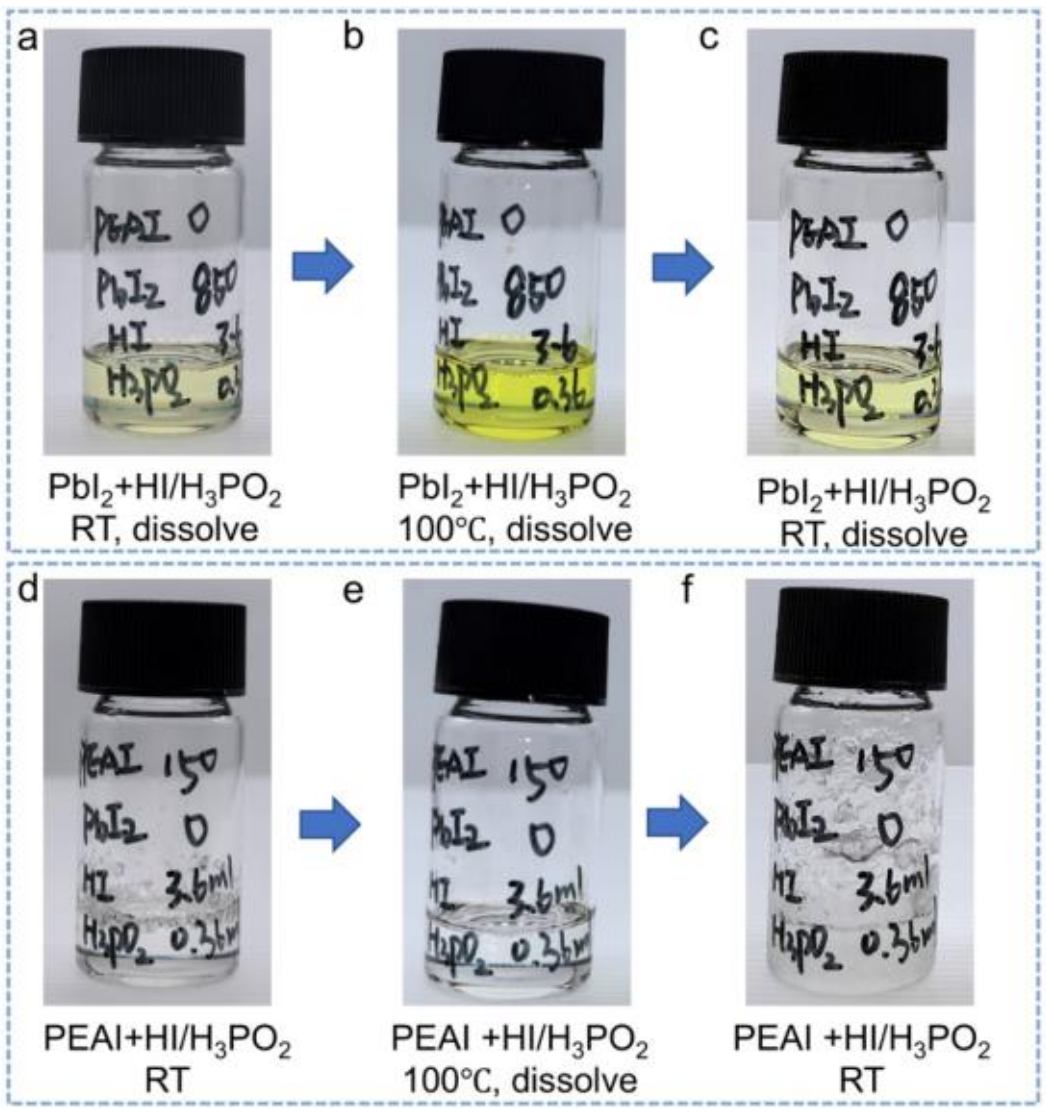


**Fig. R4. Dissolution and precipitation behaviors of precursors in the aqueous HI/$H_3PO_2$ system. a-c.** $PbI_2$ dissolves in the HI/$H_3PO_2$ mixture at both RT and 100 °C. **d-f.** PEAI dissolves at 100 °C (**e**), but forms precipitates upon cooling to RT (**f**).

In comparison, MeOH exhibits ultralow solubility for pure $PbI_2$ (Fig. R5a-b). Incrementally adding PEAI (Fig. R5c) into the MeOH/$PbI_2$ mixture causes the $PbI_2$ to gradually dissolve (Fig. R5d). This shows that dissolution of $PbI_2$ in MeOH is likely halide-complexation-driven ($PbI_2 + nI^- \rightleftharpoons [PbI_{2+n}]^{n-}$). To further investigate this, UV-Vis absorption spectroscopy were conducted (Fig. R6). When only pure $PbI_2$ is present in MeOH, the spectrum exhibits the peak corresponding to $PbI_2$. As the organic ligand increases, the absorption features of $[PbI_3]^-$ gradually emerge, followed by transition toward $[PbI_4]^{2-}$ complexes (Fig. R6). This spectral evolution substantiates the formation of higher-order iodoplumbate complexes. As previously discussed and supported by DLS tracking (Fig. R3), MeOH acts as a good solvent that keeps these complexes and $PEA^+$ extended, preventing aggregate formation. This drives the system toward

homogeneous nucleation (which inherently possesses a higher energy barrier than heterogeneous nucleation), thereby maintaining the broad metastable zone necessary to suppress secondary nucleation.

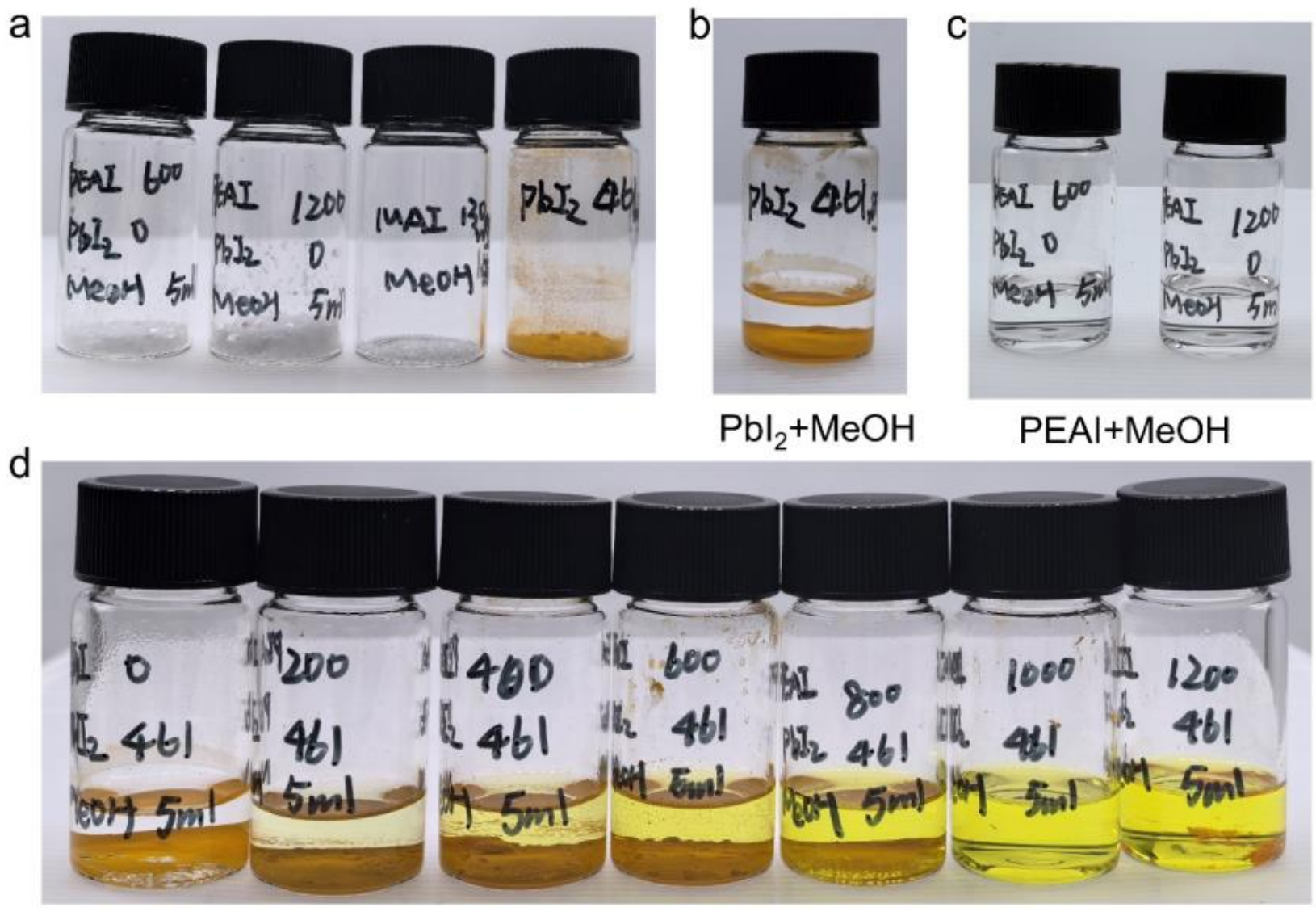


**Fig. R5. Complexation-driven dissolution of $PbI_2$ in MeOH system. a, b,** Pure $PbI_2$ exhibits limited solubility in MeOH (5 mL) even at 60 °C for 24 h. **c.** The PEAI dissolves in MeOH at RT. **d.** With a fixed amount of $PbI_2$ (461 mg) in 5 mL MeOH at 60 °C, increasing the PEAI (from 0 to 1200 mg) leads to the progressive dissolution of $PbI_2$.

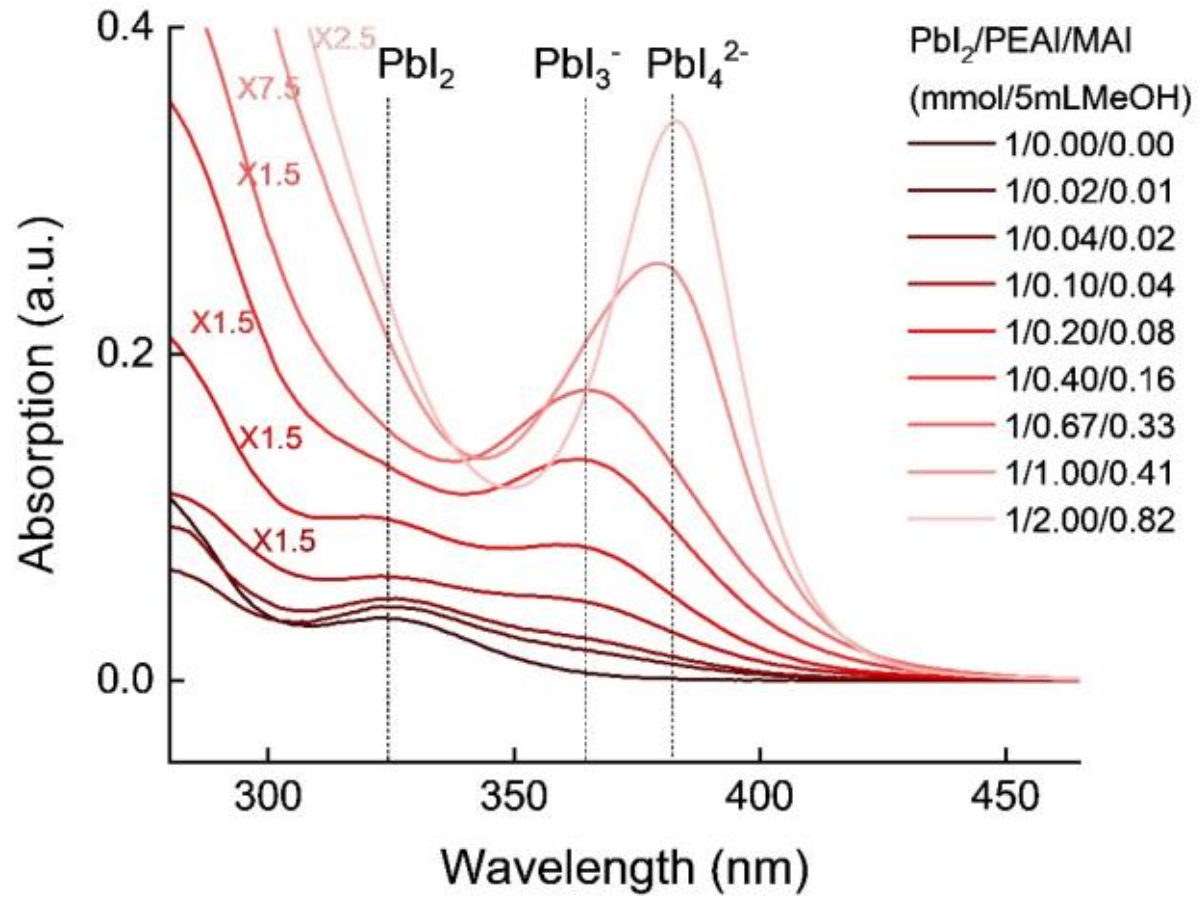


**Fig. R6. UV-Vis spectrum of the perovskite precursor solution in MeOH.**

**b)** The final crystal size in NEIT paradigm is reproducible and tunable. However, utilizing temperature gradients inevitably introduces disturbances that tend to provoke multi-nucleation. Under isothermal RT, crystal volumes can be modulated by tuning either the precursor solution volume or the initial concentration. Scaling the input solution volume from 3 to 10 mL at a fixed concentration, yielded proportionally larger crystals (129.7 to 427.5 mg). Using the theoretical density derived from the single-crystal structure (2.998 g/cm$^3$ for Pb-n2), the corresponding calculated crystal volumes are 43.3, 65.3, 89.6, 101.7, and 142.6 mm$^3$ (Fig. R7). This confirms a linear correlation

between solution volume and final crystal volume (Fig. R7a-c). Maintaining consistent inputs of MAI (130 mg), excess $PbI_2$ (461 mg), and MeOH (5 mL), we systematically varied the PEAI input (500, 450, and 400 mg). This variation adjusted the concentration of dissolved complexes (the concentration), yielding crystals weighing 220.5, 142.1, and 103.1 mg, respectively.

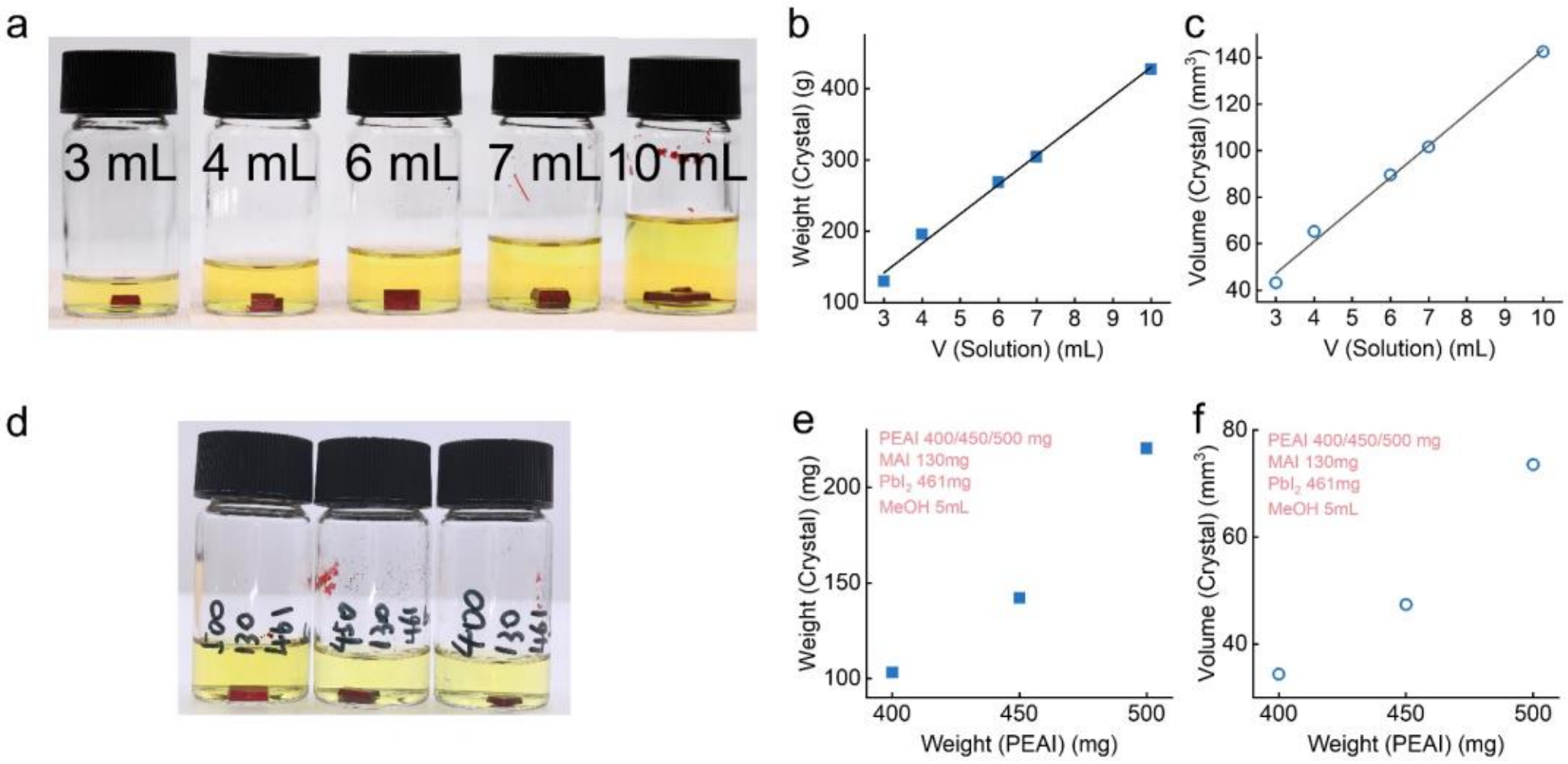


**Fig. R7. Tailoring the Pb-n2 crystal sizes in the NEIT system. a-c,** Crystal size tailoring by solution volume: **a**, Images of single crystals (Pb-n2) grown in varying precursor solution volumes (from 3 to 10 mL) with a fixed concentration. The final crystal weight (129.7, 195.8, 268.7, 304.9, and 427.5 mg) (**b**), and calculated volume (43.3, 65.3, 89.6, 101.7, and 142.6 mm$^3$) (**c**) scale linearly with the initial solution volume. **d-f,** Crystal size tailoring by initial concentration: Images of single crystals (Pb-n2) grown with varying PEAI masses (500, 450, and 400 mg) in 5 mL MeOH to modulate the initial dissolved solute concentration (**d**). The final crystal weight (**e**) and calculated volume (**f**) show a clear dependence on the PEAI mass.

***Revision:*** Figs. R4-R7 are incorporated in the revised Supplementary Information as Supplementary Figs. 1, 4, 6 and 7.

The following texts (highlighted in yellow) are included in Line 154 of the revised main manuscript: DLS tracking confirms that the precursor maintains a stable molecular state (1-2 nm) for over 22 hours at RT (Supplementary Figs. 4-6). This stability is likely because MeOH keeps the organic $PEA^+$ cations extended, which minimizes heterogeneous nucleation sites and sustains a broad metastable zone. Once a primary nucleus forms, precursor ions preferentially add to this active center due to a lower energetic barrier, effectively inhibiting secondary nucleation to yield a single crystal, demonstrating strong living characteristics (Fig. 1a)[39].

39. De Yoreo, J. J. *et al.* Crystallization by particle attachment in synthetic, biogenic, and geologic environments. *Science* **349**, aaa6760 (2015).

(Line 167) Moreover, adjusting the initial precursor concentration correspondingly modulates the final crystal size, offering two viable strategies for dimensional customization (Fig. 1b and Supplementary Fig. 7).

3. The demonstration of lasing is interesting from exfoliated flakes but has several limitations. First, while the overall improvement of carrier lifetime and diffusion length did support the much higher crystal quality grown by NEIT method, since exfoliation method is implemented, it is not impossible to find an exfoliated crystal from the SCS method that can exhibit optically pumped lasing. Second, the lasing experiments were conducted at 80K, while the possibility of room-temperature optically pumped lasing using n=1 2D perovskites bulk crystals has been demonstrated back in 2018 (Nano Letters, 2018, 18(5), 3221–3228). One angle to improve the authors' results is to study the RT lasing using as-grown bulk crystals since the NEIT-grown crystals seem to have much higher structural and cavity quality. If dimensional control is possible, then RT lasing can be controlled.

***Response:*** Thanks for your suggestion. We have conducted measurements on multiple exfoliated flakes from both NEIT- and SCS-grown Pb-n1. Under identical testing conditions, we observed lasing emission across 7 randomly selected regions on the NEIT-grown flakes. The lasing thresholds of these NEIT samples were at 9.0~17.2 μJ/cm$^2$ (Fig. R8). In contrast, when testing the SCS-grown counterparts under same conditions, we could only detect amplified spontaneous emission (ASE) signals, with a significantly higher threshold of ~100 μJ/cm$^2$ (Fig. R9).

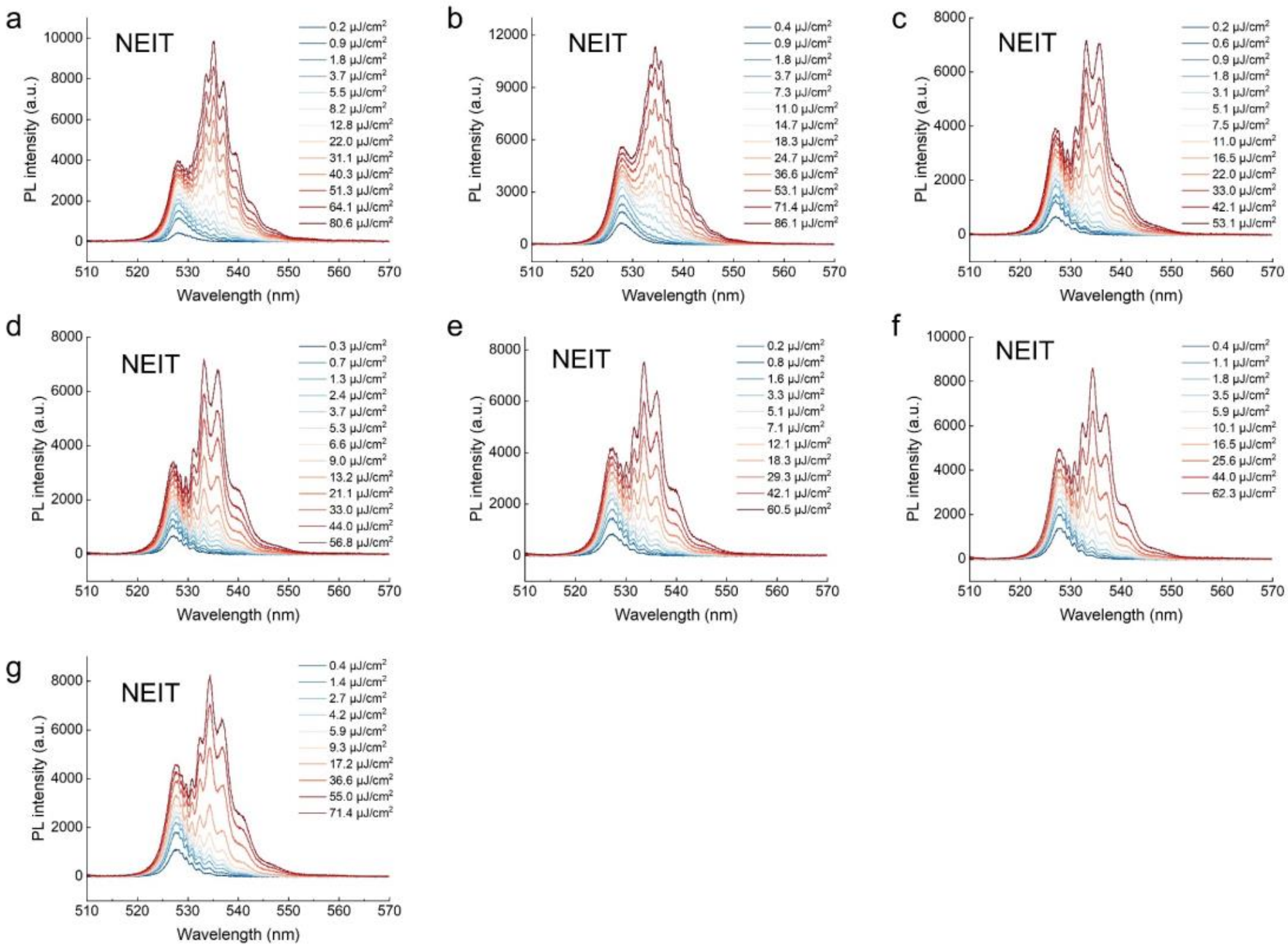


**Fig. R8. Lasing spectra of Pb-n1 exfoliated flakes synthesized from NEIT growth，including a.** Sample 1# with the pump fluence increasing from 0.2 to 80.6 μJ/cm$^2$, the lasing threshold is ~12.8 μJ/cm$^2$. **b.** Sample 2# with the pump fluence increasing from 0.4 to 86.1 μJ/cm$^2$, the lasing threshold is ~14.7 μJ/cm$^2$. **c.** Sample 3# with the pump fluence increasing from 0.2 to 53.1 μJ/cm$^2$, the lasing threshold is ~11.0 μJ/cm$^2$. **d.** Sample 4# with the pump fluence increasing from 0.3 to 56.8 μJ/cm$^2$, the lasing threshold is ~9.0 μJ/cm$^2$. **e.** Sample 5# with the pump fluence increasing from 0.2 to 60.5

μJ/cm$^2$, the lasing threshold is ~12.1 μJ/cm$^2$. **f.** Sample 6# with the pump fluence increasing from 0.4 to 62.3 μJ/cm$^2$, the lasing threshold is ~16.5 μJ/cm$^2$. **g.** Sample 7# with the pump fluence increasing from 0.4 to 71.4 μJ/cm$^2$, the lasing threshold is ~17.2 μJ/cm$^2$.

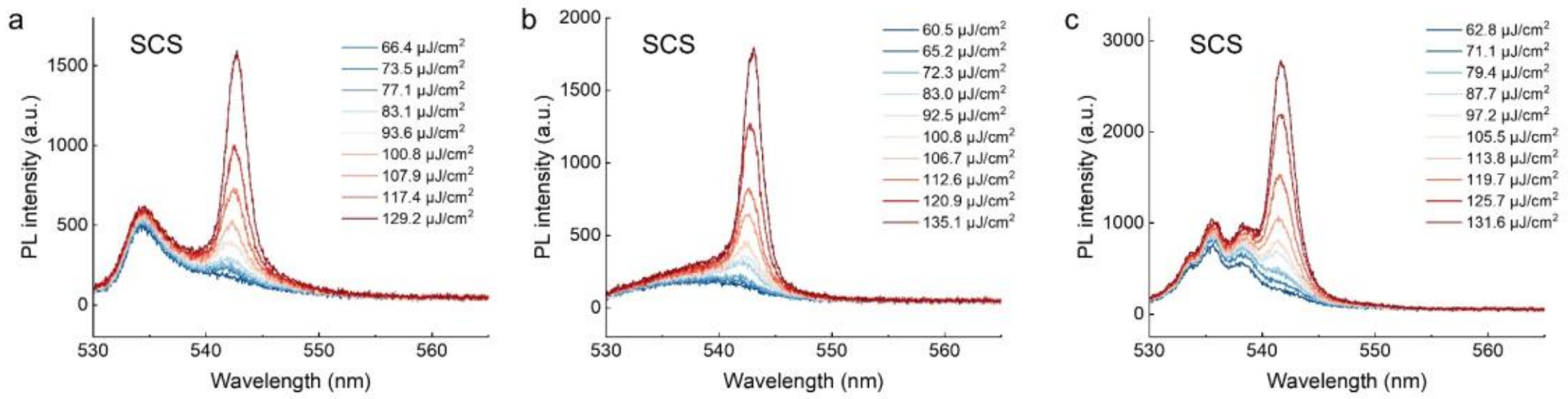


**Fig. R9. PL spectra of multiple Pb-n1 exfoliated flakes synthesized from SCS growth, including a.** Sample 1# with the ump fluence increasing from 66.4 to 129.2 μJ/cm$^2$, the lasing threshold is ~93.6 μJ/cm$^2$. **b.** Sample 2# with the pump fluence increasing from 60.5 to 135.1 μJ/cm$^2$, the lasing threshold is ~100.8 μJ/cm$^2$. **c.** Sample 3# with the pump fluence increasing from 62.8 to 131.6 μJ/cm$^2$, the lasing threshold is ~97.2 μJ/cm$^2$.

RT lasing was reported in bulk n=1 single crystal ($BA_2PbI_4$) (*Nano Lett.* **2018**, 18, 3221-3228). According to our review of the 215 papers citing this work, it was rarely reproduced by any other group in n=1 systems so far, highlighting the exceptionally stringent cavity control required in this study. We attempted to reproduce the RT lasing using our NEIT-grown bulk single crystals under comparable experimental conditions, yet did not observe lasing behavior.

Based on the fundamental understanding, achieving RT lasing in n=1 $PEA_2PbI_4$ bulk single crystal remains extremely challenging due to several intrinsic physical barriers. First, the self-absorption loss in thick bulk crystals is substantial. As n=1 2D perovskites possess the largest exciton binding energy and a small Stokes shift (*Adv. Optical Mater.* **2023**, 11, 2202038; *Mater. Today Chem.* **2023**, 29, 101455), the overlapping of absorption and emission spectra leads to high internal losses that prevent population inversion in bulk crystal. In bulk crystals, self-absorption depletes the optical gain before lasing. Exfoliating the bulk crystals into thin flakes dramatically reduces this, fundamentally bypassing the self-absorption. Second, the thermal management is critical at RT. Thin exfoliated flakes on a substrate benefit from much more efficient heat dissipation compared to bulk crystals, where local heating under high-power pumping can rapidly degrade the optical gain and even the material itself. Lastly, as noted in the literature (e.g., *Adv. Mater.* **2019**, 1903030; *ACS Nano* **2022**, 16, 13082-13091), n=1 lead perovskites suffer from severe Auger recombination and strong exciton-phonon coupling at RT, which collectively suppress the optical gain. The successful observation of lasing from Pb-n1 bulk crystals in 2018 *Nano Letters* work likely benefited from specific whispering-gallery mode (WGM) cavities, which consist of many rectangular plateaus with facets on the crystal surface. In contrast, our bulk single crystals possess flat, continuous surfaces that inherently lack such structure (Fig. R10).

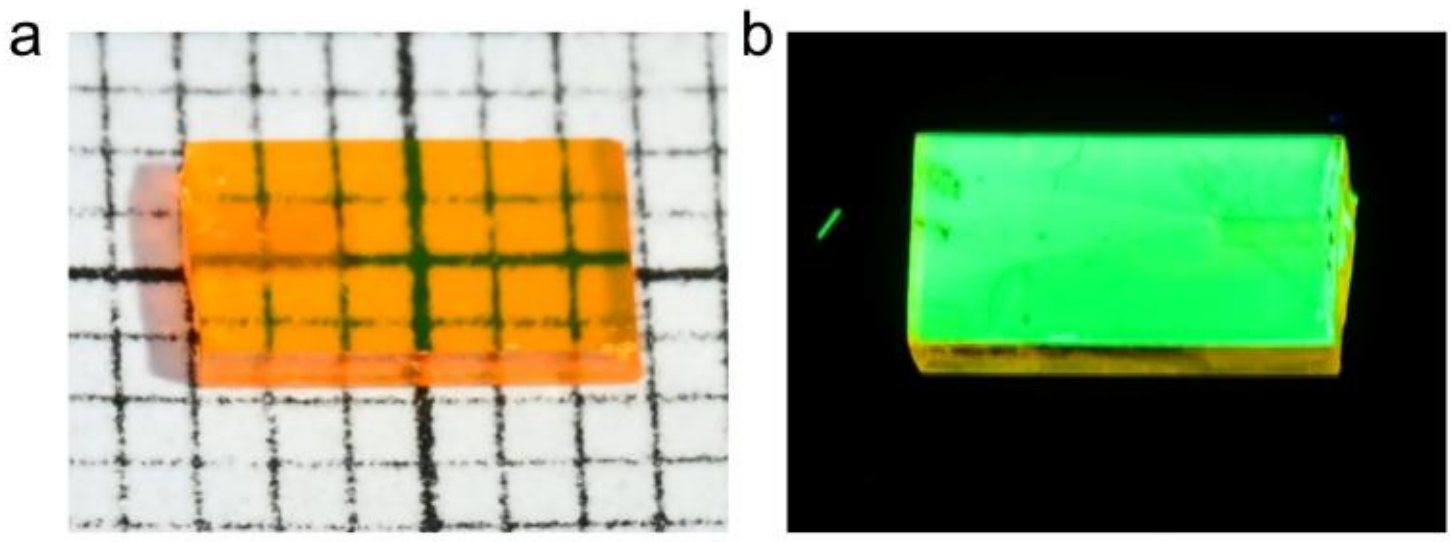


**Fig. R10. Photographs of the Pb-n1 single crystal synthesized via the NEIT method.** (**a**) Optical and (**b**) PL images, illustrating a highly flat surface and uniform internal structure.

***Revision:*** Figs. R8 and R9 are incorporated in the revised Supplementary Information as Supplementary Figs. 20 and 22.

The following texts (highlighted in yellow) are included in Line 229 of the revised main manuscript: We conducted measurements across randomly selected regions with varying flake thicknesses (Supplementary Fig. 20). The NEIT-grown flakes exhibited multimode lasing with threshold of ~15 μJ/cm$^2$ (Fig. 1h and Supplementary Fig. 21). In contrast, exfoliated flakes from SCS-grown crystals tested under identical conditions only displayed amplified spontaneous emission (ASE) with a significantly higher threshold (~100 μJ/cm$^2$) (Supplementary Fig. 22).

Additionally, a few minor issues can be better clarified.

1. It would be a good idea to present shortly how the utilization rates of $PbI_2$ are calculated for both techniques used for comparison in the paper, as the numbers used for the SCS method seem lower than the NEIT method. In particular, the utilization rates of $PbI_2$ for NEIT synthesis can be accurately obtained by calculating the ratio of Pb precursor in the crystal and in the precursor down to the decimal place while that is not the case for the SCS method.

***Response:*** We sincerely thank you for pointing this out. This section clearly presents the calculation details for both the NEIT and SCS methods, as detailed below:

1) The NEIT Method:

For the NEIT method, the $PbI_2$ utilization rate represents the conversion of the dissolved $PbI_2$ precursor into the final Pb-n1 crystal. Since PEAI is fully dissolved in MeOH and only partial $PbI_2$ remains undissolved, the dissolved $PbI_2$ mass is calculated as the total initial $PbI_2$ input mass minus the mass of filtered undissolved $PbI_2$. The equivalent mass of $PbI_2$ within the Pb-n1crystal is determined by its stoichiometric ratio (e.g., for $PEA_2PbI_4$, the $PbI_2$ mass is the final crystal mass multiplied by the molar mass ratio of 461/959). The formula is:

$$PbI_2 \quad Utilization_{NEIT} \ (\%) = \left(\frac{Mass \ of \ equivalent \ PbI_2 \ in \ the \ grown \ crystal}{Mass \ of \ dissolved \ PbI_2 \ in \ the \ solution}\right) \times 100$$

2) The SCS Method:

In the SCS method, all initially added $PbI_2$ is fully dissolved in the solution, so the

utilization rate for SCS be calculated based on the total initial $PbI_2$ added. The equivalent mass of $PbI_2$ within Pb-n1 crystals is identical to that in the NEIT system. The formula is:

$$PbI_2 \quad Utilization_{SCS} \ (\%) = \left(\frac{Mass \ of \ equivalent \ PbI_2 \ in \ the \ grown \ crystal}{Total \ initial \ mass \ of \ PbI_2 \ added}\right) \times 100$$

**Supplementary Table R1. | Raw mass data and $PbI_2$ utilization calculations for $PEA_2PbI_4$ crystal growth through NEIT method.**

| | Precursors of Pb-n1, NEIT | | | Undissolved $PbI_2$ (mg) | Dissolved $PbI_2$ (mg) | $PEA_2PbI_4$ mass (mg) | Equivalent $PbI_2$ in crystal (mg) | $PbI_2$ Utilization (%) |
|---|---|---|---|---|---|---|---|---|
| | PEAI (mg) | $PbI_2$ (mg) | MeOH (mL) | | | | | |
| 1 | 700 | 461 | 5 | 269.9 | 191.1 | 356.4 | 171.3 | 89.7 |
| 2 | 700 | 461 | 5 | 282 | 179 | 327.2 | 157.3 | 87.9 |

**Supplementary Table R2. | Raw mass data and $PbI_2$ utilization calculations for $PEA_2PbI_4$ crystal growth through SCS method.**

| | Precursors of Pb-n1, SCS | | | $PEA_2PbI_4$ mass (mg) | Equivalent $PbI_2$ in crystal (mg) | $PbI_2$ Utilization (%) |
|---|---|---|---|---|---|---|
| | PEAI (mg) | $PbI_2$ (mg) | $HI+H_3PO_2$ (mL) | | | |
| 1 | 300 | 1700 | 9+1 | 458.6 | 220.5 | 13.0 |
| 2 | 300 | 1700 | 9+1 | 479.4 | 230.5 | 13.6 |
| 3 | 300 | 1700 | 9+1 | 419.9 | 201.8 | 11.9 |

***Revision:*** We added a new section titled "Calculation method for $PbI_2$ utilization rates" in the Supplementary information (1. Supplementary Characterizations, Page 5). Supplementary Tables R1-2 are incorporated in the revised Supplementary Information as Supplementary Tables 1-2.

2. The SCLC measurement, data processing, and reproducibility should be better clarified. According to Blakesley et al. (2014), control factors such as film thickness and electrode quality can impact the measurement of trapped density, up to a factor of 3. Corre (2024) also highlighted a variety of potential problems with SCLC measurements such as the presence of mobile charges and contact asymmetry in perovskites.
https://pubs.acs.org/doi/10.1021/acs.jpclett.4c02379
https://pubs.acs.org/doi/10.1021/acsenergylett.9b02720

***Response:*** Thanks for your suggestion. Our devices are based on bulk 2D Ruddlesden-Popper (RP) phase perovskite single crystals. The organic layers in 2D RP perovskites physically block the pathways for halide ion migration. Consequently, the ion migration

in 2D single crystals is suppressed by several orders of magnitude compared to 3D perovskites (*Adv. Mater.* **2024**, 36, 2309588.). To ensure the accuracy of our measurements, we employed a strictly controlled pulsed-voltage SCLC (PV-SCLC) technique (*ACS Energy Lett.* **2020**, 5, 376-384) to suppress any conceivable transient mobile charges. The measurements were conducted at a low scan rate (0 V to 20 V, step size 0.4 V, transient pulse integration time of ~200 ms, and a pulse delay of 2 s). All tests were performed inside a dark electromagnetic shielding box to exclude photo-induced carriers. Additionally, to ensure ideal Ohmic contacts, we engineered an electron-only device configuration: Ag/BCP/C60/perovskite /C60/BCP/Ag.

The accuracy of SCLC fundamentally depends on the precise determination of the active layer thickness ($L$), as the trap density ($n_{trap}$) is calculated by: $n_{trap} = \frac{2\varepsilon\epsilon_0 V_{TFL}}{eL^2}$. To eliminate the geometrical uncertainties mentioned by Blakesley et al., we directly measured the exact cross-sectional thickness ($L$) of each single crystal using optical microscopy. To definitively prove reproducibility, we conducted SCLC measurements on a new batch of crystals with significantly reduced thicknesses (91 μm for NEIT and 42 μm for SCS, compared to the original 225 μm and 78 μm) (Fig. R11). Despite the substantial variations in $L$ and the corresponding shifts in the trap-filled limit voltage ($V_{TFL}$), the derived $n_{trap}$ values remained consistently within the identical order of magnitude ($3.48\times10^9$ $cm^{-3}$ for NEIT and $4.90\times10^{10}$ $cm^{-3}$ for SCS). This thickness-independent consistency robustly rules out geometry-induced artifacts and validates our SCLC reliability.

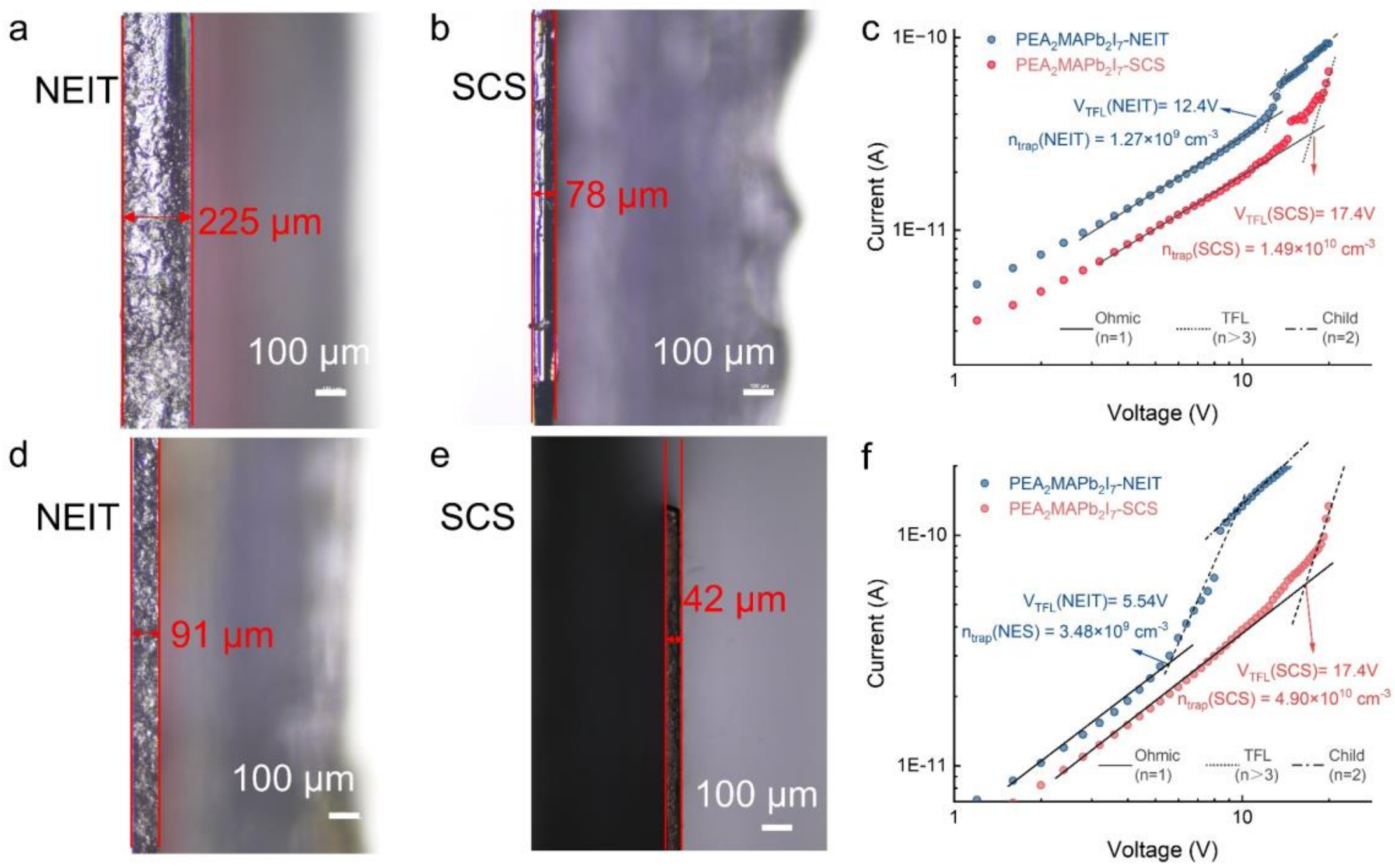


**Fig. R11. Reproducibility of SCLC measurements and cross-sectional thickness determination of $PEA_2MAPb_2I_7$ single crystals. a, b,** Images and thicknesses of the NEIT (**a**, 225 μm) and SCS (**b**, 78 μm) samples in the manuscript. **c.** The corresponding dark current-voltage (I-V) characteristics of the SCLC measurements in **a** and **b**. **d, e,** Thickness of a newly fabricated batch of NEIT (**d**, 91 μm) and SCS (**e**, 42 μm) single crystals. **f.** The corresponding dark current-voltage

(I-V) characteristics in **d** and **e**.

For 2D hybrid perovskites, the average dielectric constant ($\varepsilon_{ave}$) is a volume-weighted composite of the inorganic well ($\omega$) and the organic barrier (b): $\varepsilon_{ave} = \frac{\varepsilon_\omega L_\omega + \varepsilon_b L_b}{L_\omega + L_b}$ (*Proc. Natl. Acad. Sci.* **2019**, 116, 58-66; *ACS Energy Lett.* **2024**, 9, 226-242). $\varepsilon_\omega = 6.1$ and $L_\omega = 12.8$ Å for the inorganic layer were used. Because the organic spacer comprises a bilayer, its effective thickness was doubled, yielding $\varepsilon_b = 3.3$ and $L_b = 6.7$ Å $\times 2$. This precise calculation yields an $\varepsilon_{ave} = 4.7$, which was applied in our $n_{trap}$ calculations.
Finally, our data show the distinct conduction regimes, allowing the precise extraction of $V_{TFL}$ following existing literatures (*Nat. Commun.* **2026**, https://doi.org/10.1038/s41467-026-71490-0; *Nat. Commun.* **2018**, 9, 5302; *ACS Photonics* **2022**, 9, 3529-3539).

***Revision:*** Fig. R11 is incorporated in the revised Supplementary Information as Supplementary Fig. 37.
The following texts (highlighted in yellow) are included in Line 772 of the revised main manuscript: Trap state densities were evaluated using SCLC measurements in electron-only devices with a device structure of Ag (120nm) /BCP (6nm) /$C_{60}$ (35nm) /Perovskite /$C_{60}$ (35nm) /BCP (6nm) /Ag (120nm). Dark current–voltage characteristics were acquired with a Keithley 2636A source meter. To quantify the trap density and suppress potential artifacts from transient mobile charges or contact asymmetry, we employed a strictly controlled pulsed-voltage SCLC technique on symmetric electron-only devices[58]. The SCLC measurements were conducted at a low scan rate (step size 0.4 V, transient pulse integration time of ~200 ms, and a pulse delay of 2 s) inside a dark electromagnetic shielding box. Furthermore, the exact cross-sectional thickness of each measured single crystal was directly determined via optical microscopy.

58. Duijnstee, E. A. *et al.* Toward understanding space-charge limited current measurements on metal halide perovskites. *ACS Energy Lett.* **5**, 376-384 (2020).

**Reviewer #2 (Remarks to the Author):**

Han et al. described a near-equilibrium isothermal growth strategy to improve the quality of 2D metal halide perovskites. The crystals are cm in size and can be applied to several compositions. The crystals demonstrated improved optoelectronic properties, including high PLQY and reported cavity-free lasing. Furthermore, the near-equilibrium growth approach allows formation of vdWs or lateral epitaxial heterostructures. The new structure could broaden potential applications. The results are significant, but several claims and analysis require closer scrutiny and greater scientific rigorous before the manuscript can be considered for acceptance.

1. The authors described the single crystal growth process as near-equilibrium isothermal growth, but additional explanation is required. Why was MeOH chosen as the solvent instead of other candidates? The manuscript also lacks the key explanation for why there is only one nuclei per vial. From the methods section, only two batches of samples were grown using the NEIT route. More statistical evidence of the single nucleation behavior should be provided.

***Response:*** Thanks for your suggestion. We have clarified the solvent selection reason below.

(1) DMF and DMSO have high donor number (DN) values (26.6 for DMF and 29.8 for DMSO) and high dielectric constants (36.7 for DMF and 46.7 for DMSO) (Solvents and Solvent Effects in Organic Chemistry, *Wiley*, **2011**). Their strong Lewis basicity causes stable coordination ([$Pb(Solvent)_n$]$^{2+}$) with $PbI_2$, limiting the formation of lead-halide precursor clusters (*Science* **2022**, 377, 1425-1430; *ACS Appl. Energy Mater.* **2019**, 2, 3400-3409; *ACS Energy Lett.* **2018**, 3, 92-97; *Acc. Chem. Res.* **2016**, 49, 330-338).

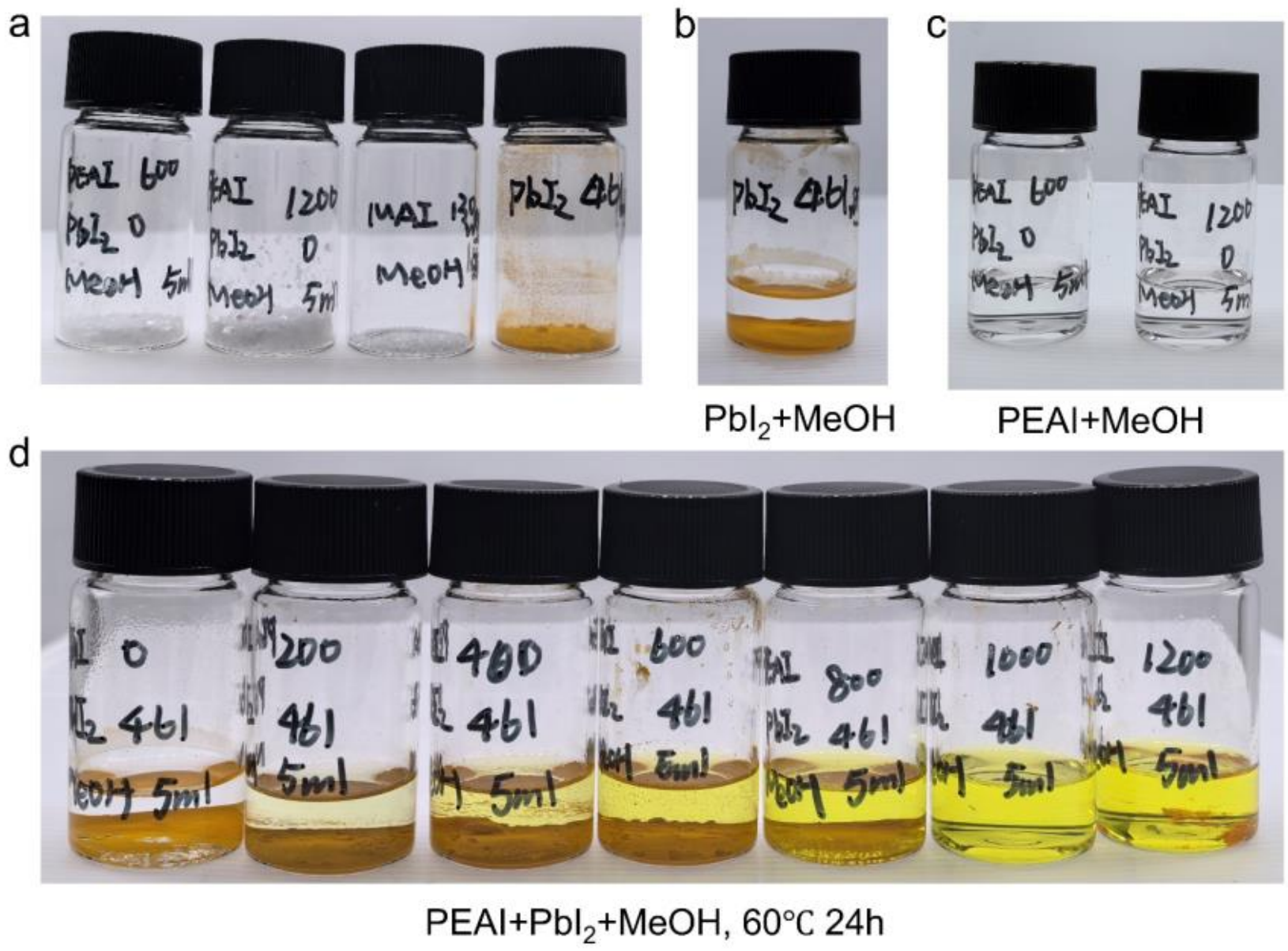


**Fig. R12. Complexation-driven dissolution of $PbI_2$ in MeOH system. a, b,** Pure $PbI_2$ exhibits limited solubility in MeOH (5 mL) even at 60 °C for 24 h. **c.** The PEAI dissolves in MeOH at RT.

**d.** With a fixed amount of $PbI_2$ (461 mg) in 5 mL MeOH at 60 °C, increasing the PEAI (from 0 to 1200 mg) leads to the progressive dissolution of $PbI_2$. (this figure is also incorporated in our response to 2nd comment of Reviewer 1 as Fig. R5).

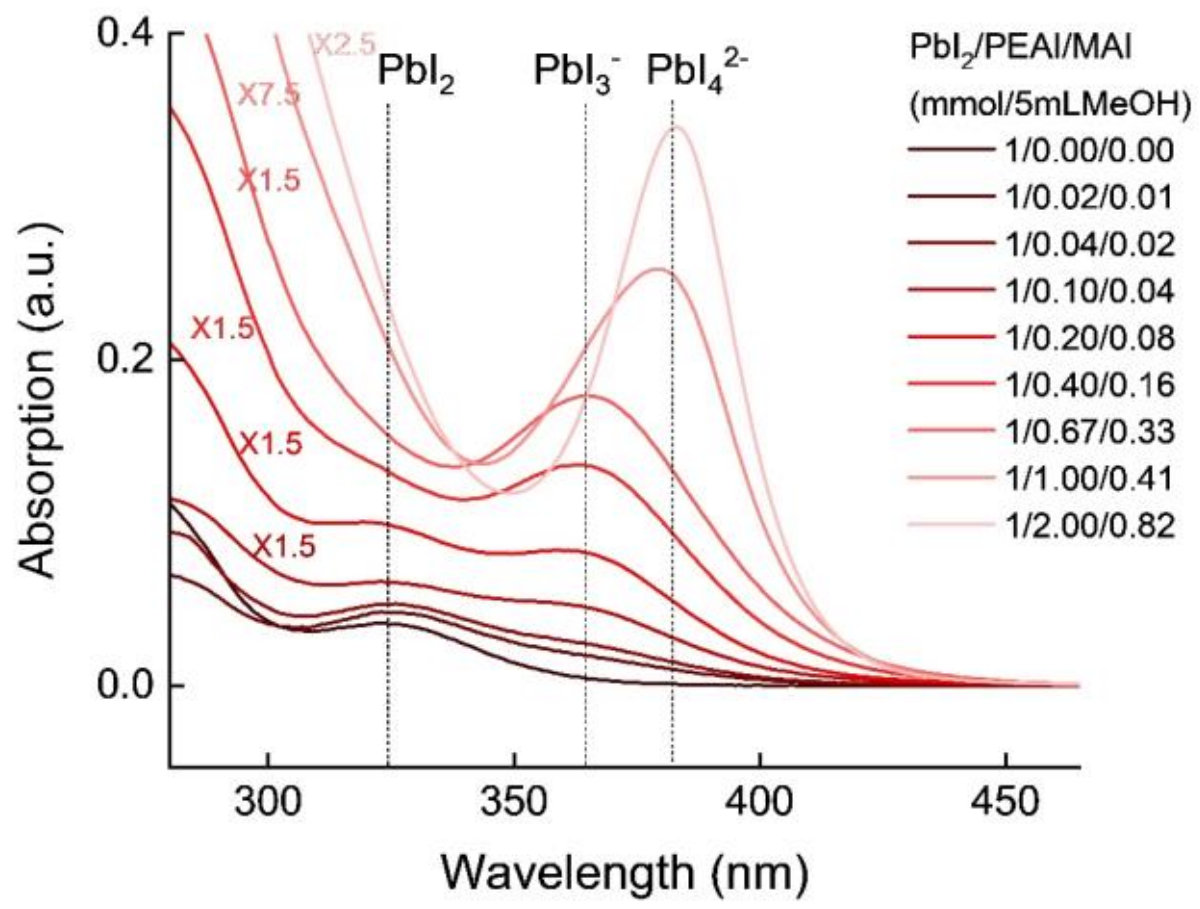


**Fig. R13. UV-Vis spectrum of the perovskite precursor solution in MeOH.** (this figure is also incorporated in our response to 2nd specific comment of Reviewer 1 as Fig. R6).

In contrast, weakly coordinating alcohols possess lower DN (19.0 for MeOH, 19.2 for EtOH and 21.1 for IPA) and cannot dissolve $PbI_2$ through direct solvent coordination (Fig. R12b). We verified this mechanism in the MeOH system: MeOH exhibits ultralow solubility for pure $PbI_2$ (Fig. R12a-b). Incrementally adding PEAI into the MeOH/ $PbI_2$ mixture causes $PbI_2$ to gradually dissolve (Fig. R12c-d). This indicates that the dissolution of $PbI_2$ in MeOH is likely halide-complexation-driven ($PbI_2 + nI^- \rightleftharpoons [PbI_{2+n}]^{n-}$). UV-Vis spectra confirm the evolution from $PbI_2$ to higher-order iodoplumbate complexes, such as $[PbI_3]^-$ and $[PbI_4]^{2-}$, upon adding PEAI. This spectral evolution provides experimental evidence for halide-complexation-driven dissolution. In MeOH, EtOH and IPA, we observed a solvent-dependent solubility. As shown in Fig. R14a-c, while PEAI is dissolved in MeOH, EtOH and IPA, its solubility in IPA is lowest at R. Furthermore, when mixing the same amount of PEAI and $PbI_2$ in different alcohols (Fig. R14d-f), the undissolved precursor residue increases from MeOH to EtOH to IPA. Evaporating the filtered clear supernatants further confirms this: the MeOH supernatant yields abundant yellow $PEA_2PbI_4$ crystals, whereas the EtOH supernatant yields sparse yellow crystals alongside white residues (likely PEAI), and the IPA supernatant yields white PEAI. This indicates that the amount of $PbI_2$ dissolved in EtOH and IPA is very low. We attribute this to the decreasing solvent polarity. As the dielectric constant decreases from MeOH (ε~32.7) to EtOH (ε~24.5) to IPA (ε~18.3) (Solvents and Solvent Effects in Organic Chemistry, *Wiley*, **2010**), the solvent's ability to dissociate PEAI and electrostatically stabilize the charged iodoplumbate complexes (e.g., $[PbI_3]^-$, $[PbI_4]^{2-}$) is progressively reduced (*ACS Energy Lett.* **2018**, 3, 1, 92-97). Consequently, the halide complexation equilibrium ($PbI_2 + nI^- \rightleftharpoons [PbI_{2+n}]^{n-}$) is restricted in lower-dielectric alcohols (EtOH, IPA).

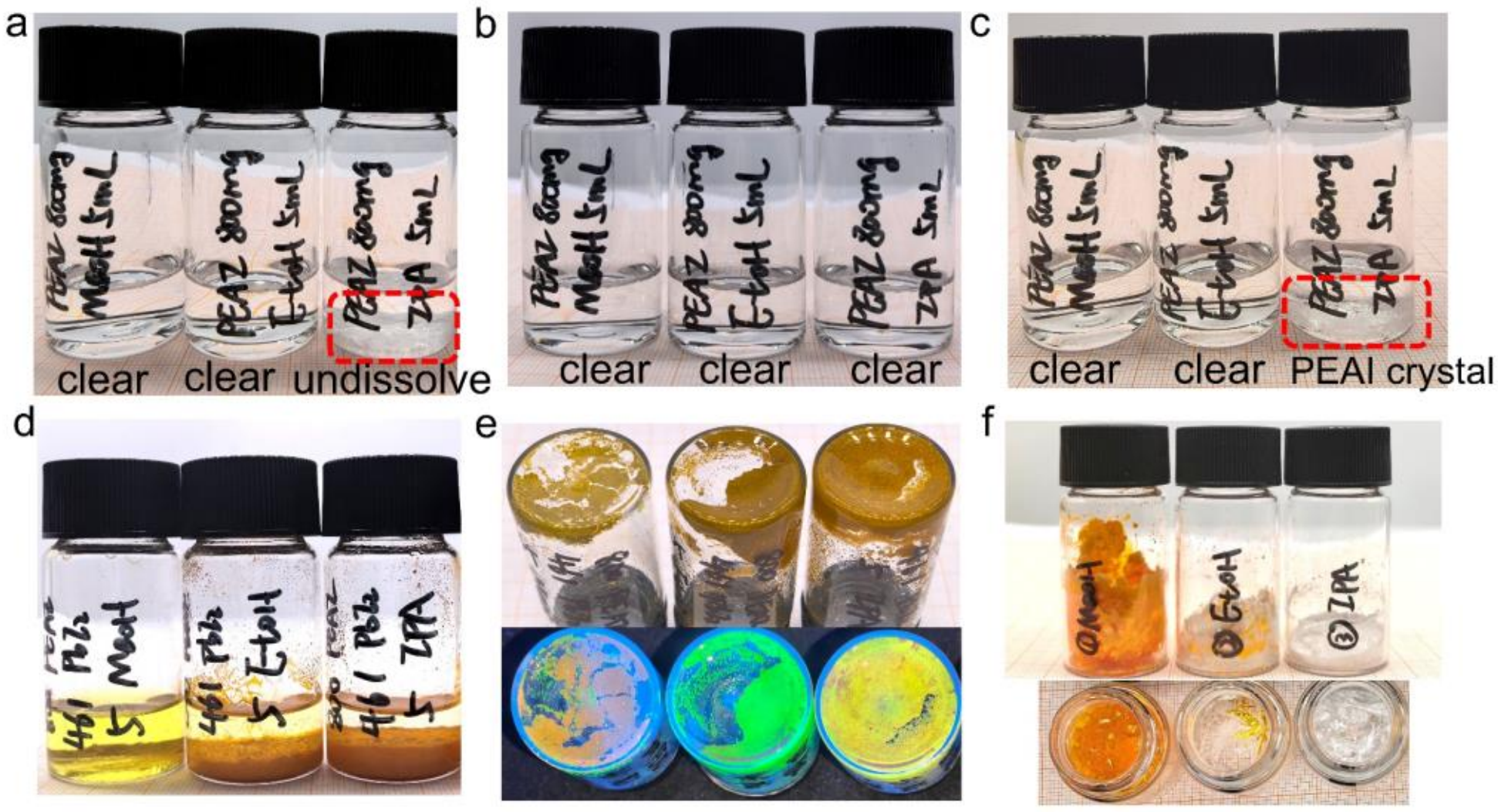


**Fig. R14. Solubility and complexation behaviors of perovskite precursors in different alcohol solvents. a.** PEAI (800 mg) in 5 mL of MeOH, EtOH, and IPA after 5 minutes of stirring at RT. PEAI is readily dissolved in MeOH and EtOH to form clear solutions but remains partially undissolved in IPA. **b.** Complete dissolution of PEAI in all three solvents achieved after continuous heating at 60 °C for 12 hours. **c.** Precipitation behaviors after cooling these solutions to RT. PEAI crystals exclusively precipitate from the IPA solution. **d.** Dissolution states of PEAI (800 mg) and $PbI_2$ (461 mg) mixtures in 5 mL of MeOH, EtOH, and IPA after 12 hours of heating at 60 °C. **e.** Optical and UV-illuminated images of the filtered undissolved residues from the respective mixtures in (**d**). **f.** Solid products obtained by evaporating the filtered supernatants in a vacuum oven at 60 °C.

(2) To understand the single-nucleation behavior in the NEIT (MeOH) strategy, we first examine the high nucleation density in the HI system. Dynamic light scattering (DLS) reveals that slight cooling (100 °C to 90 °C) triggers a transition from a molecular state (~2 nm) to macroscopic aggregates (>1000 nm) (Fig. R15). We attribute this to the rapid hydrophobic aggregation of bulky $PEA^+$ cations, which generates abundant heterogeneous nucleation sites, indicating a narrow metastable zone (defined as the concentration region where a solution is supersaturated but spontaneous nucleation does not occur) during dynamic cooling (Crystallization (Fourth Edition), *Butterworth-Heinemann*, **2001**). Driven by this narrow metastable zone, the HI system tends to undergo uncontrolled multi-nucleation.

In contrast, the NEIT strategy operates RT isothermally in MeOH. As evidenced by DLS tracking (Fig. R16), the precursor solution maintains a stable molecular state (1-2 nm) for over 22 hours at RT, confirming the absence of large aggregates. This is likely because MeOH keeps the $PEA^+$ cations extended, effectively minimizing heterogeneous nucleation sites, leading to homogeneous nucleation. Since the energy barrier for homogeneous nucleation is significantly higher than that of heterogeneous nucleation, spontaneous nucleation is effectively inhibited, thereby sustaining a broad metastable zone. Within this metastable zone, once a primary nucleus forms, it acts as the main active growth center (*Science* **2015**, 348, aaa6760). The precursor ions preferentially add to this existing crystal due to the lower energetic barrier, thus often

yielding only a single crystal in the solution. Furthermore, it is important to note that achieving and sustaining this single-nucleation behavior also strictly requires maintaining stable physical boundary conditions: keeping the growth vessel clean (to exclude potential external heterogeneous sites) and ensuring an environment free from mechanical or thermal disturbances (to prevent localized energy fluctuations that could inadvertently trigger nucleation).

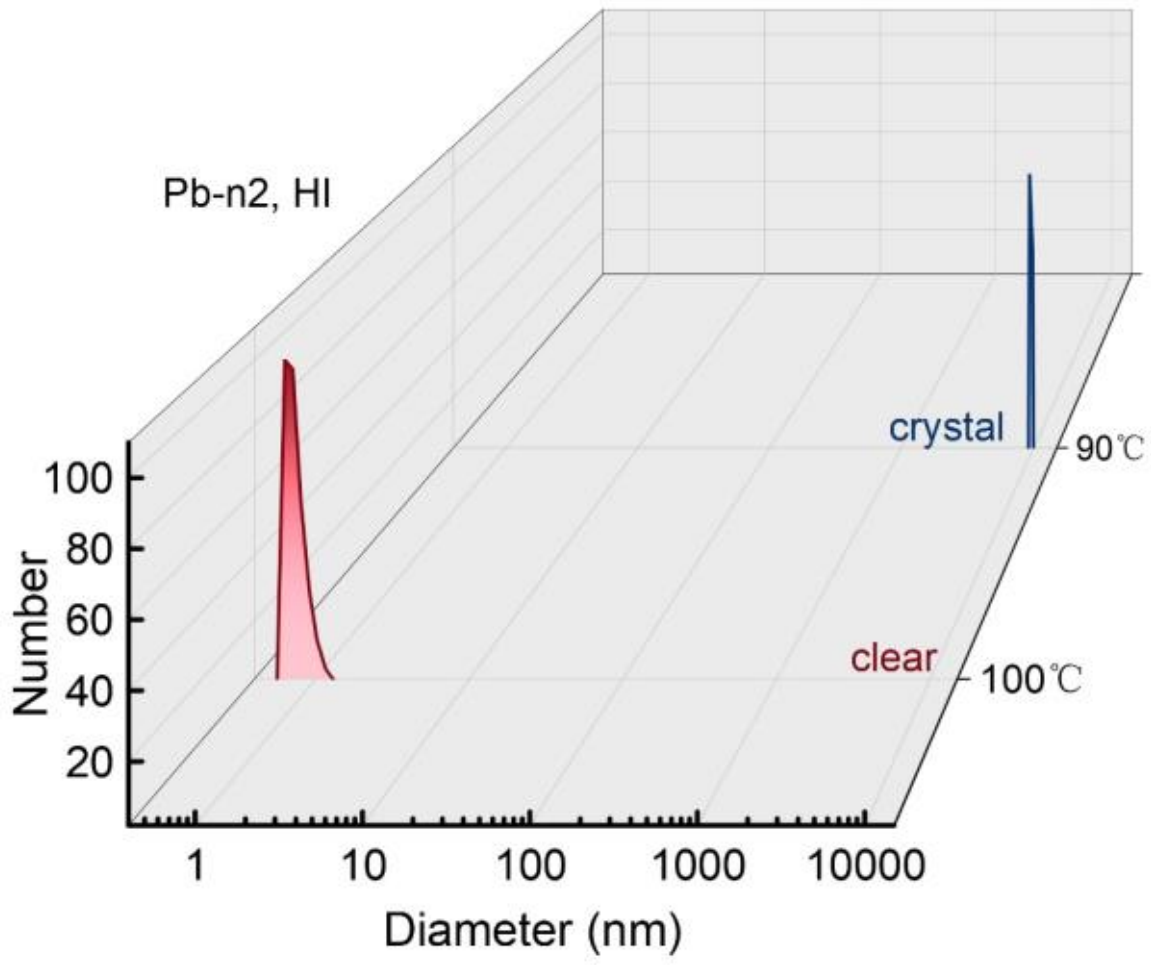


**Fig. R15. Dynamic light scattering (DLS) tracking of precursor solution in the HI system.** Number distribution of the Pb-n2 precursor solution during cooling. A molecular-scale peak (~2 nm) is observed at 100 ℃, while cooling to 90 ℃ triggers the appearance of macroscopic scattering signals (>1000 nm). (this figure is also incorporated in our response to the 1st specific comment of Reviewer 1 as Fig. R1).

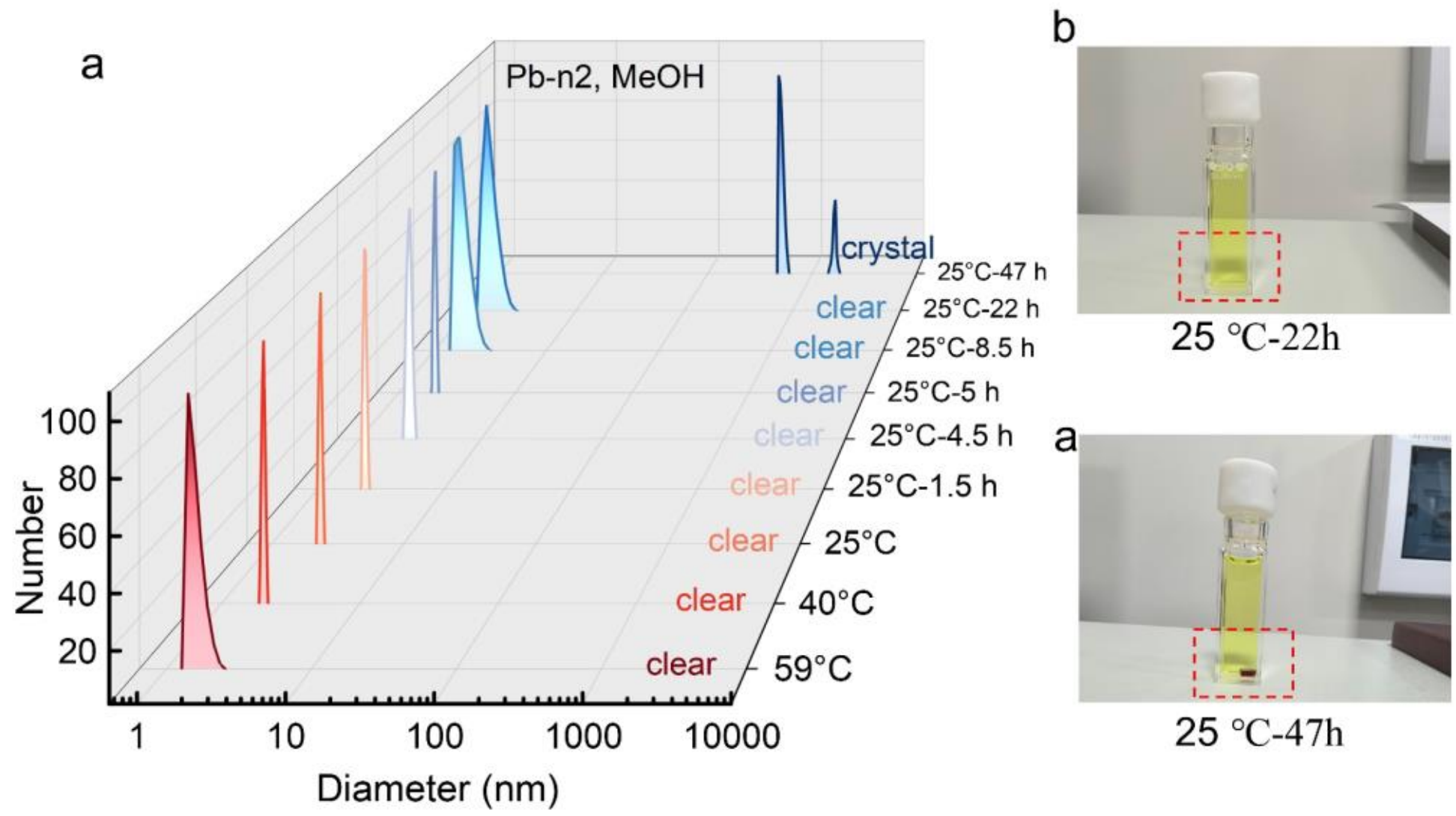


**Fig. R16. DLS tracking and macroscopic observation of the MeOH system. a.** DLS distribution of the Pb-n2 precursor solution during cooling (59 ℃ to 25 ℃) and RT aging at 25 ℃. The profile maintains a peak at ~1-2 nm for over 22 hours. (Note: The initial temperature was set to 59 ℃ due

to the DLS instrument's safety restriction, which must remain below the boiling point of MeOH). **b,** The solution remains visually clear after 22 h at RT. **c,** After 47 h, a crystal at the bottom of the vial. (this figure is also incorporated in our response to the 1st specific comment of Reviewer 1 as Fig. R3).

(3) We wish to clarify that the "two batches" mentioned in the Methods section referred to the specific samples used to calculate the precursor utilization yield. It does not mean we only performed the crystal growth twice. In fact, the high reproducibility of the single-nucleation behavior is demonstrated throughout the manuscript. For example, Fig. 1b shows five independent growths in different precursor volumes (3 to 10 mL), with each yielding a primary single crystal. Supplementary Fig. 34 also tracks the continuous growth of a single crystal.

***Revision:*** Figs. R12-R16 are incorporated in the revised Supplementary Information as Supplementary Figs. 4, 6, 3, 2 and 5 respectively.

The following texts (highlighted in yellow) are included in Line 144 of the revised main manuscript: Strong Lewis base solvents (e.g., DMF, DMSO) are excluded as their stable coordination with $Pb^{2+}$ suppresses the formation of critical precursor clusters [35-37]. Among weakly coordinating alcohols, the decreasing dielectric constant from MeOH to EtOH and IPA progressively restricts the dissolution of $PbI_2$ precursors[36] (Supplementary Fig. 3).

35. Radicchi, E. et al. Understanding the solution chemistry of lead halide perovskites precursors. *ACS Appl. Energy Mater.* **2**, 3400-3409 (2019).
36. Hamill, J. C. et al. Influence of solvent coordination on hybrid organic–inorganic perovskite formation. *ACS Energy Lett.* **3**, 92-97 (2017).
37. Sidhik, S. et al. Deterministic fabrication of 3D/2D perovskite bilayer stacks for durable and efficient solar cells. *Science* **377**, 1425-1430 (2022).

2. In Supplementary Fig.15, no crystals are visible in Day4 picture, but a centimeter sized crystal is shown in the next photo at Day9. The authors should provide more information on the intermediate stage.

***Response:*** We thank the reviewer for pointing this out. We have conducted a new, monitored NEIT growth. By controlling the photographic procedure to minimize disturbance, we successfully captured the continuous macroscopic evolution. As shown in Fig. R17, the intermediate stages from Day 5 to Day 10 are now documented.

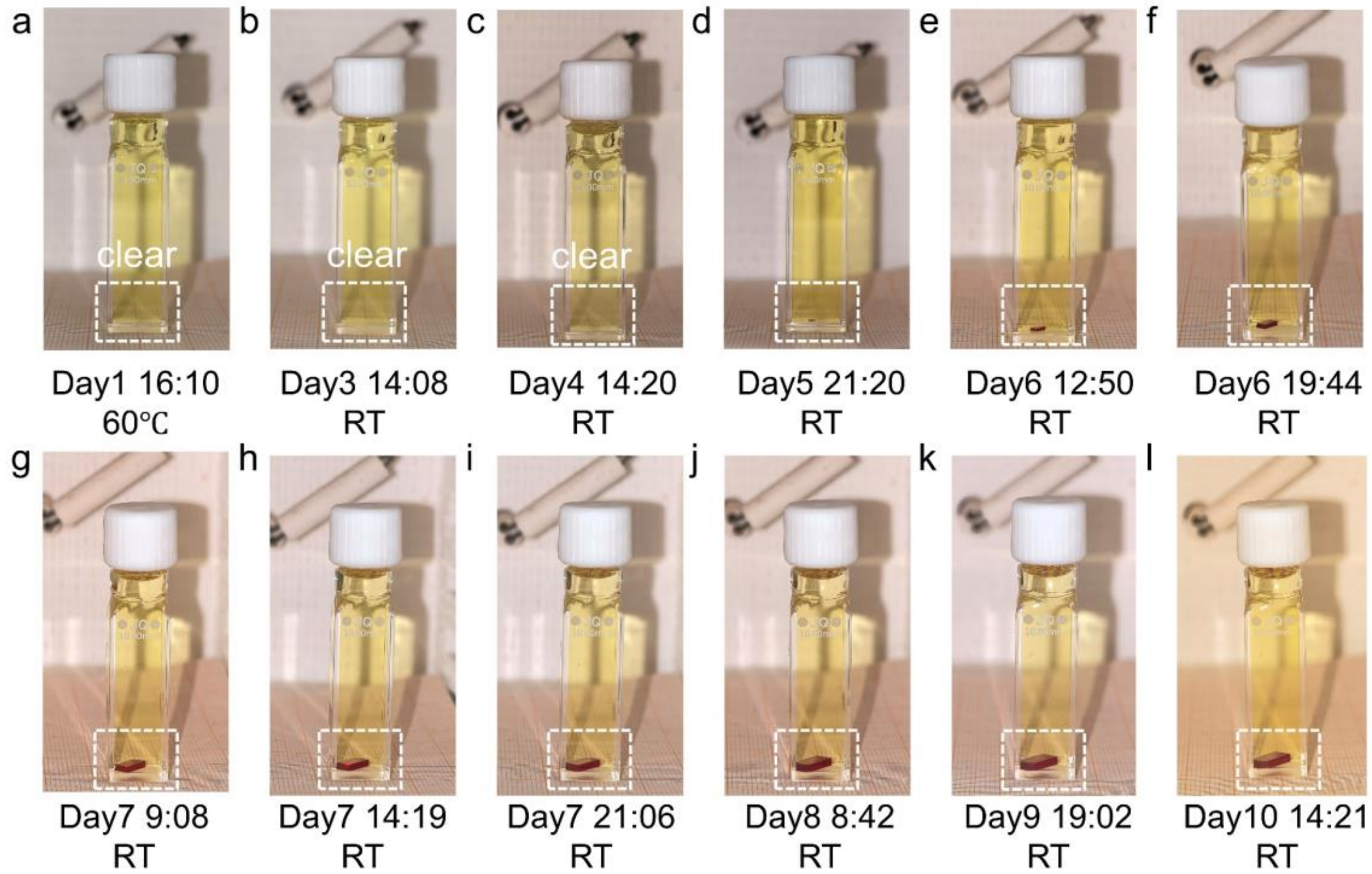


**Fig. R17. Continuous tracking of the living NEIT single crystal growth process of Pb-n2. a.** The precursor solution was initially placed in the muffle furnace at 60 ℃. **b-l.** The solution was cooled down and maintained at RT. The solution remained visibly clear (**b, c**). **d-l.** Continuous image recordings of the growth process from Day 5 to Day 10.

***Revision:*** Fig. R17 is incorporated in the revised Supplementary Information as Supplementary Fig. 35.

3. The authors provided rocking curve and GIXRD results to demonstrate improved crystallinity:
-The GIXRD results appear significantly flawed and can be excluded, along with the claim of a 40% reduction in residue strain. Specifically, the Bragg peak at ~4deg decreased by 0.16deg when sampling from surface to bulk, which would correspond to an implausibly large (a few percent) strain for a bulk single crystal. Alternatively, the authors could exfoliate the surface layer and measured XRD directly of the bulk region to confirm this strain. A more likely explanation is measurement artefact due to misalignment when changing the tilt angle, that caused significant peak shifts at low angles. Also, such big residue strain is not consistent with the narrow FWHM from rocking curve measurements.
-The rocking curve results alone support improved crystallinity. However, the author should make sure that difference in surface roughness is accounted for rocking curve broadening. X-ray reflectivity should provide indication of the surface roughness and can be measured on the same sample using the same instrument. Ideally, exfoliated samples should be used to maintain a flat surface.

***Response:*** We sincerely thank you for this highly constructive comment. Upon re-

evaluating this methodology, we recognized that this strain characterization technique is primarily applied to thin films (perovskite thin films or perovskite oxide thin films) (*Nat. Commun.* **2019**, 10, 815; *Nat. Commun.* **2024**, 15, 2579; *Energy Environ. Sci.* **2025**,18, 2452-2461), and is rarely applied to perovskite bulk single crystals. Following your constructive suggestion, we have deleted the GIXRD results and removed the claim regarding the "40% reduction in residue strain" from the main manuscript.

In addition, we clarify that all rocking curve measurements for both NEIT and SCS crystals were conducted on mechanically exfoliated surfaces. To validate the measurement accuracy, a NEIT Pb-n2 crystal was mechanically exfoliated to expose a clear, mirror-like facet (Fig. R18a). X-ray reflectivity (XRR) analysis confirmed this surface to be ultra-smooth, with a fitted roughness of < 2 nm. Without altering the sample position, subsequent rocking curve measurements were performed (Fig. R18). The acquired profiles align closely with prior data, thereby substantiating the reliability of the initial rocking curve results.

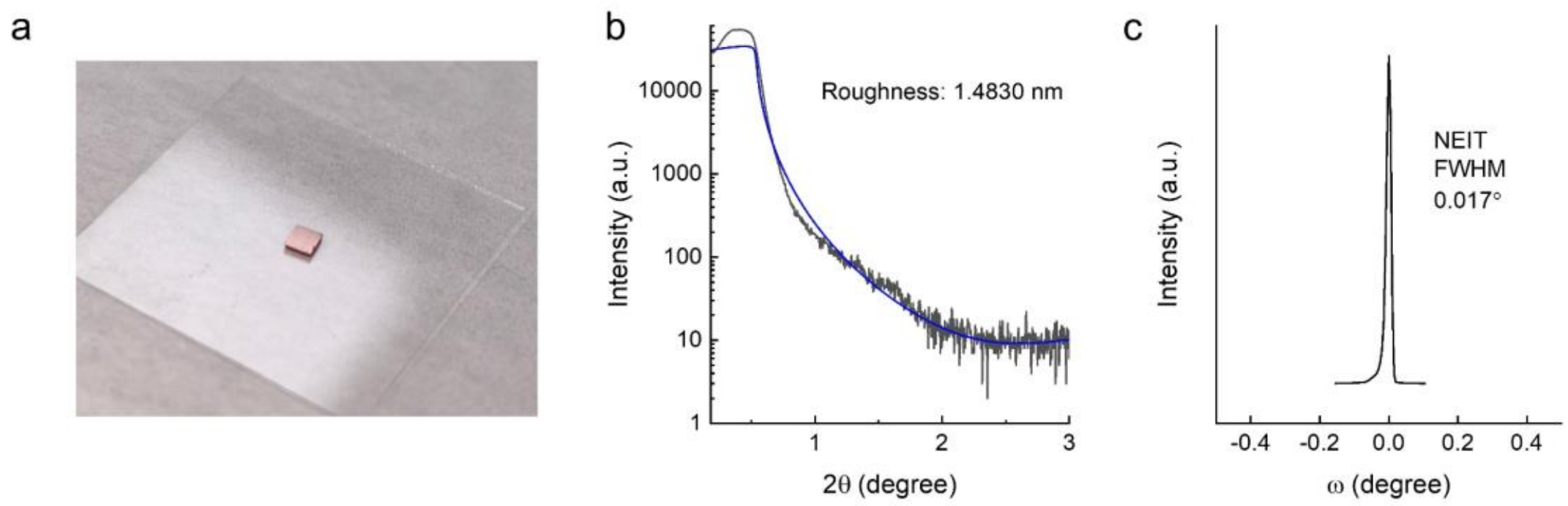


**Fig. R18. Structural characterization of NEIT-grown Pb-n2. a,** Optical image of the exfoliated bulk single crystal, exposing a clear, mirror-like facet. **b,** X-ray reflectivity (XRR) profiles (gray lines) and corresponding fitting curves (blue lines) for NEIT-grown Pb-n2 sample. The extracted surface roughness value is 1.4830 nm. **c,** High-resolution X-ray rocking curve of the (001) diffraction taken on the identical area, exhibiting an ultra-narrow full-width at half-maximum (FWHM) of 0.017°.

***Revision:*** Figs. R18 is incorporated in the revised Supplementary Information as Supplementary Fig. 12.

4. The measurement of 2D perovskite PLQY was not performed in an integration sphere.
-Did the authors consider the anisotropic emission commonly observed in 2D perovskites? Since the optic setup does not collect emission from all directions, assuming a Lambertian profile (which is unclear in the current manuscript) may introduce big error? Back focal plane imaging or angle-resolved PL measurement should confirm the emission profile.
-In addition, what is the sample thickness used for the PLQY measurement? For cm-thick crystals, how much transmission signal is still there for 450 nm excitation? Thick crystals exhibit strong reabsorption effect, therefore, even the reference sample reported

as 55% measured PLQY could have a much larger internal quantum efficiency, which will lower down the claimed difference.

***Response:*** Thanks for your suggestion.

1) To verify the emission profile and ensure our micro-spectroscopy setup does not introduce collection angle errors, we conducted PL polarization and angle-resolved PL measurements on the exfoliated flakes. The circular polar plots for n=1, 2, and 3 perovskites demonstrate that the in-plane PL emission is isotropic (Fig. R19), which is consistent with previous literature (*Sci. Adv.* **2025**, 11, eadx1686). Subsequent angular scanning covering -60° to +60° (Fig. R20) shows that emission intensity and spectral shape exhibit negligible angle dependence for all perovskites (Pb, n=1-3) (*Nat. Mater.* **2023**, 22, 1065-1070). These confirm isotropic emission from our mechanically exfoliated 2D perovskite flakes.

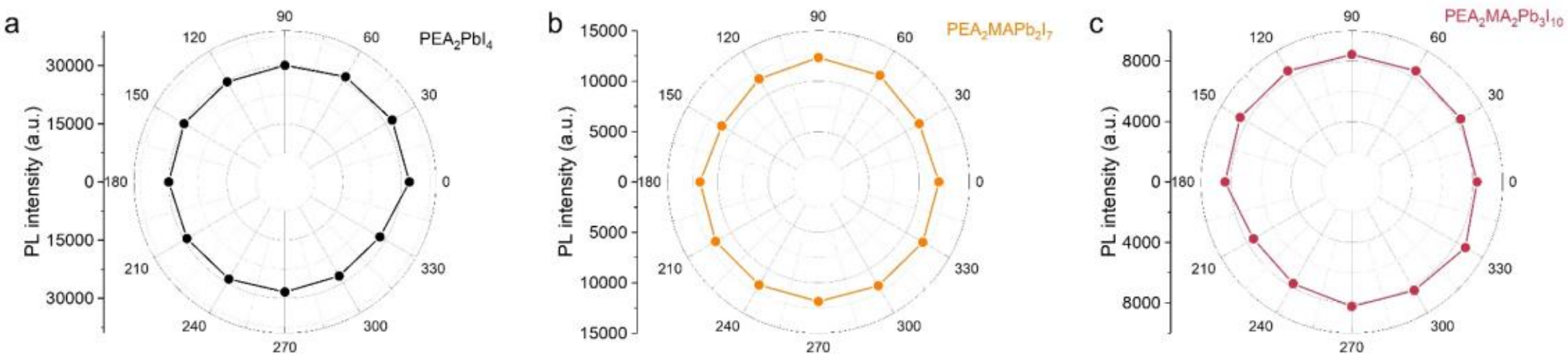


**Fig. R19. In-plane PL polarization of Pb-n1 (a), Pb-n2 (b) and Pb-n3 (c).**

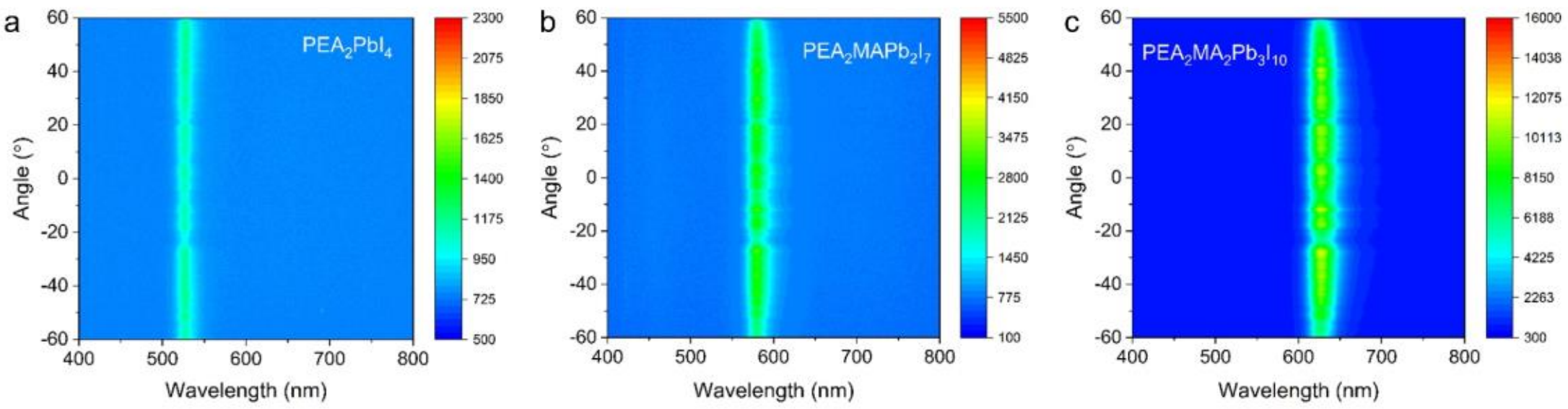


**Fig. R20. Angle-resolved PL spectra of Pb-n1 (a), Pb-n2 (b) and Pb-n3 (c).**

2) To ensure a fair comparison between crystals grown by NEIT and SCS, we selected mechanically exfoliated flakes with similar thicknesses, which exhibit similar self-absorption effect. For example, in SCS, the thickness of n1 nanosheets ranged from 111 to 120 nm, and in NEIT, the n1 nanosheets had a thickness ranging from 109 to 117 nm (Figs. R21-R24). Consequently, the contrast in our measured PLQY values (95% for NEIT vs. 55% for SCS) is reliable. Figs. R21-R24 detail the thickness of the nanoflakes used for our PLQY measurements, along with their absorption, transmission, and reflection values under 454 nm laser excitation. These data confirm that the excitation light can penetrate the sample.

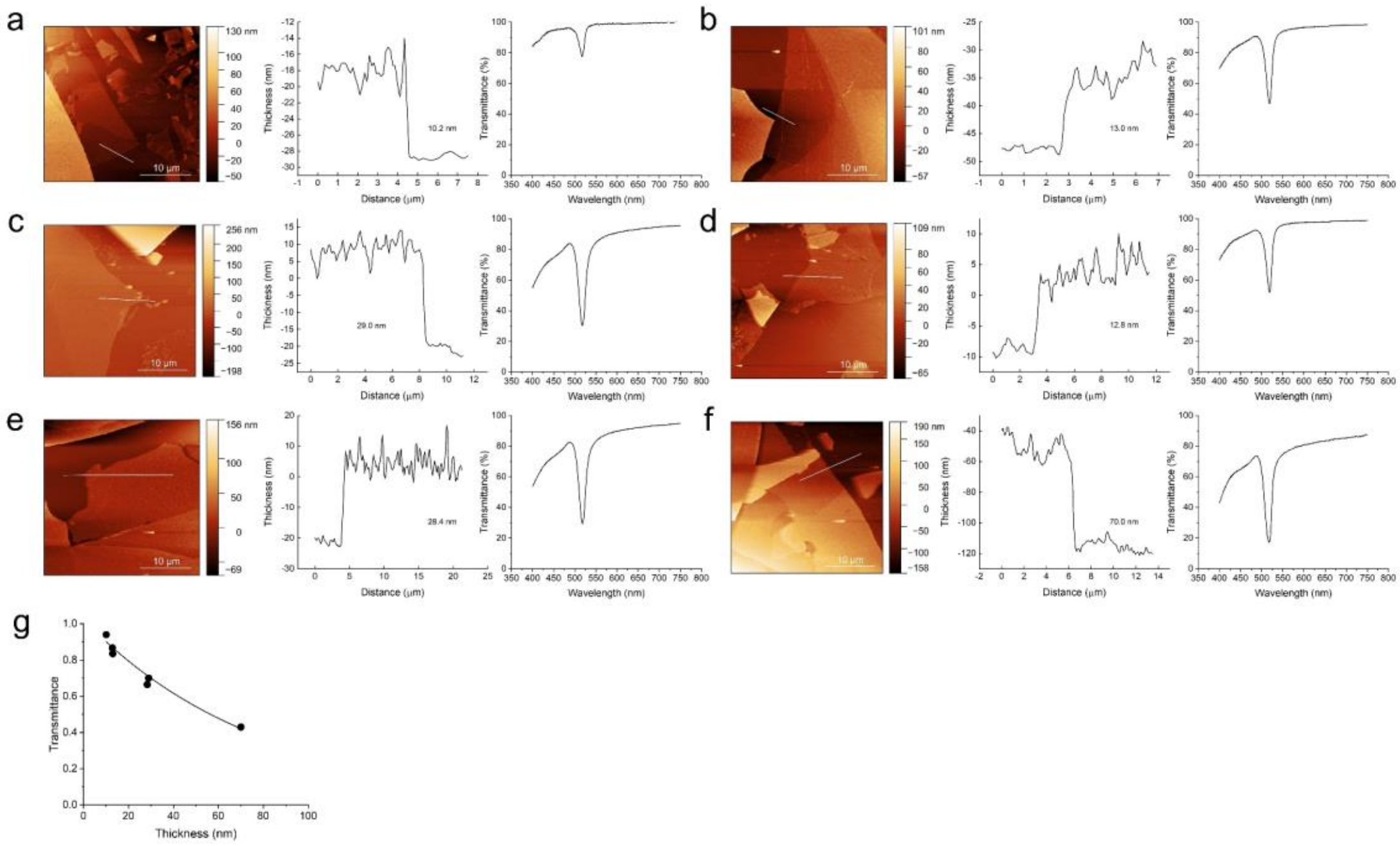

**Fig. R21. Establishment of the thickness-absorbance correlation for exfoliated Pb-n1 flakes. a-f.** Atomic force microscopy (AFM) images (left), corresponding height profiles (middle), and transmittance (right) of mechanically exfoliated Pb-n1 thin flakes with varying thicknesses. The measured thicknesses range from ~10.2 nm to ~70.0 nm, with the corresponding transmittance values extracted at 454 nm. **g**, The extracted correlation between the thickness and transmittance (454 nm).

To quantify the thickness of the flakes utilized in our PLQY measurements, we established thickness-transmittance calibration. We performed AFM and transmittance measurements on a series of exfoliated Pb, n=1-3 flakes with varying thicknesses. In accordance with the Beer-Lambert law, the measured transmittance exhibits a correlation with the AFM-determined thickness. Utilizing this established relationship, the thickness of exfoliated flake can be fitted from its transmittance signal under 454 nm.

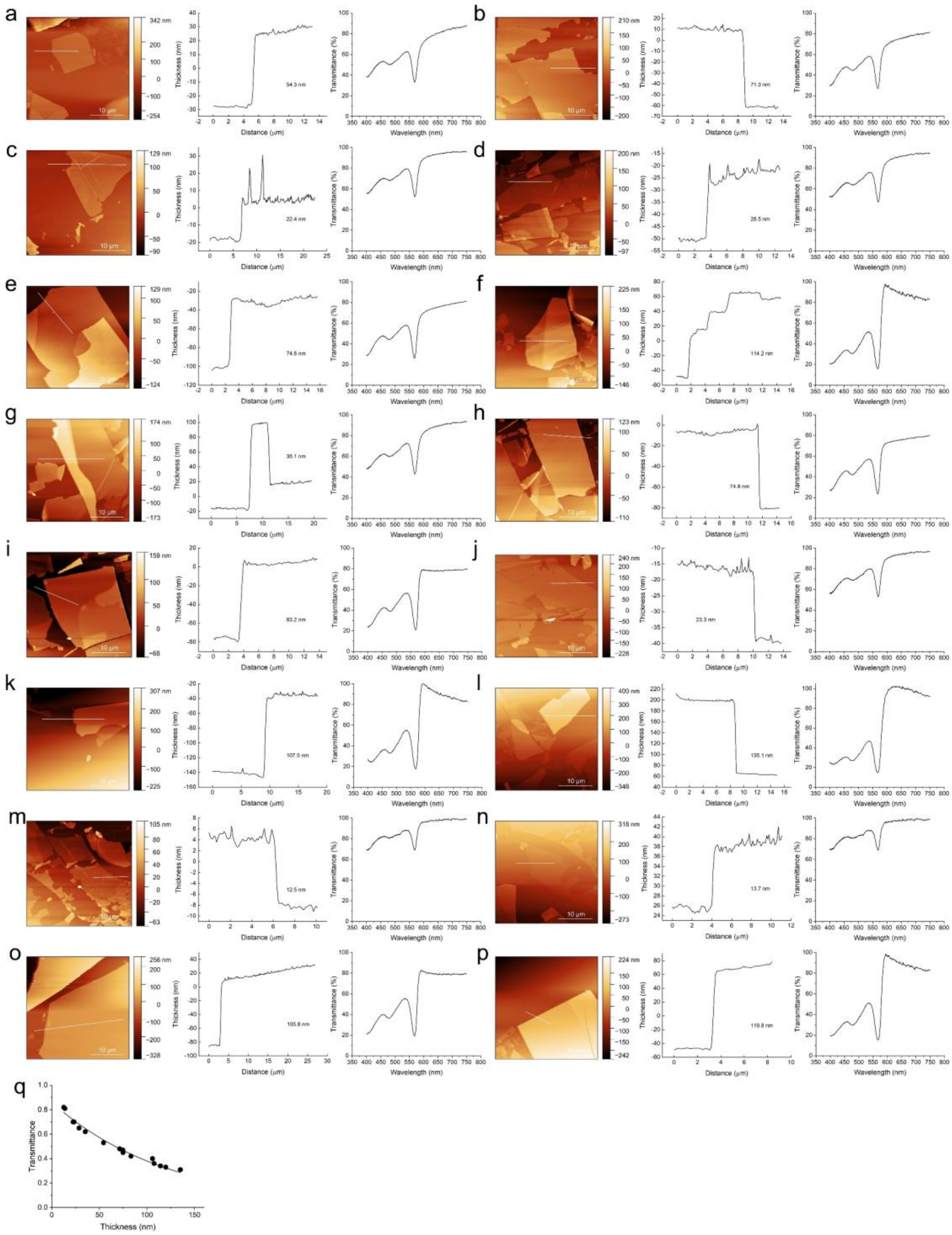

**Fig. R22. Establishment of the thickness-absorbance correlation for exfoliated Pb-n2 flakes. a-p.** Atomic force microscopy (AFM) images (left), corresponding height profiles (middle), and transmittance (right) of mechanically exfoliated Pb-n2 thin flakes with varying thicknesses. The measured thicknesses range from ~12.5 nm to ~135.1 nm, with the corresponding transmittance values extracted at 454 nm. q, The extracted correlation between the thickness and transmittance (454 nm).

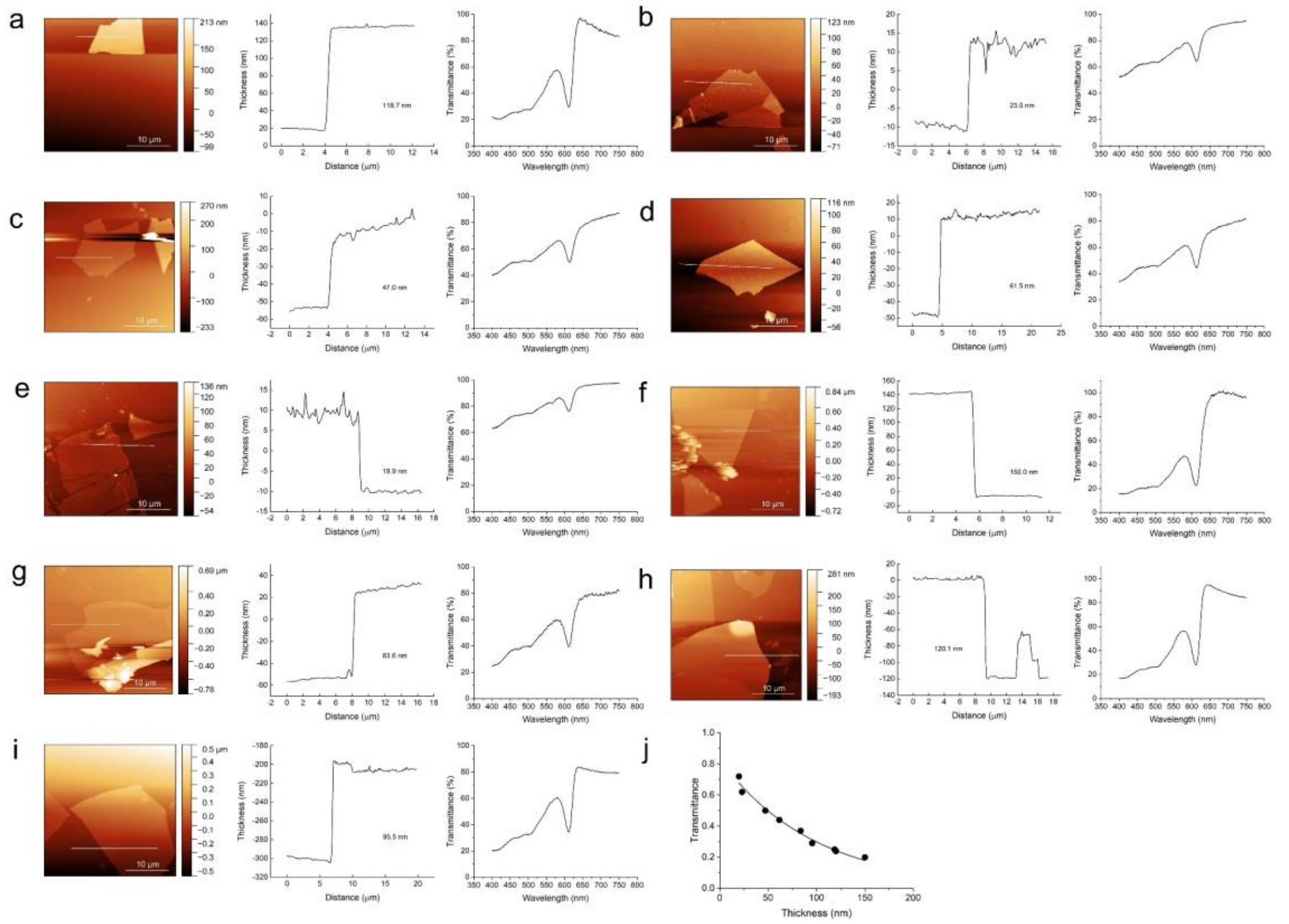

**Fig. R23. Establishment of the thickness-absorbance correlation for exfoliated Pb-n3 flakes. a-i.** Atomic force microscopy (AFM) images (left), corresponding height profiles (middle), and transmittance (right) of mechanically exfoliated Pb-n3 thin flakes with varying thicknesses. The measured thicknesses range from ~19.9 nm to ~150.0 nm, with the corresponding transmittance values extracted at 454 nm. **j**, The extracted correlation between the thickness and transmittance (454 nm).

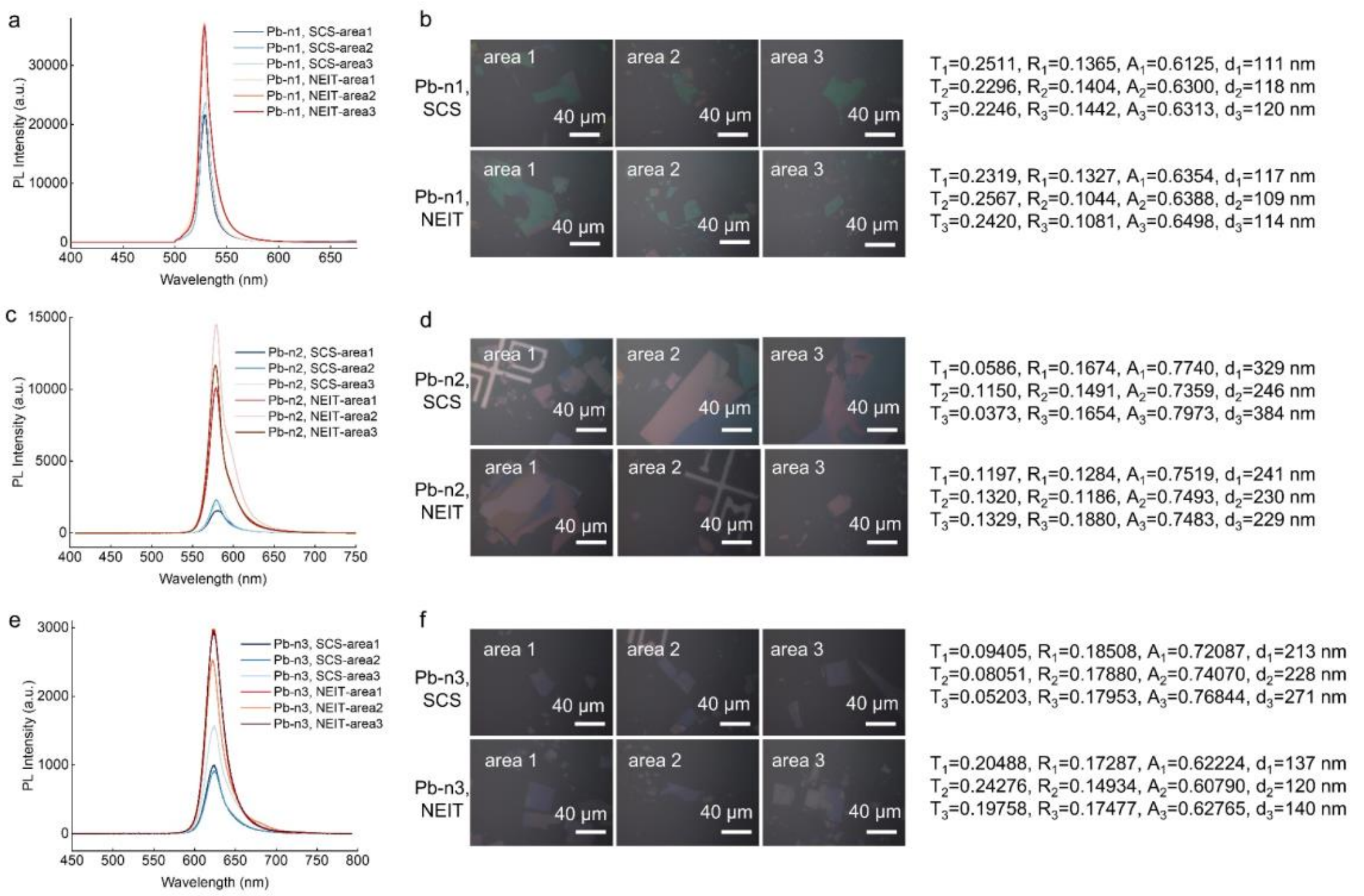


**Fig. R24. PL intensity of 2D perovskites excited under identical laser fluence (454 nm, 320 nW, 40 MHz, ~300 nJ/cm$^2$). a.** PL spectra of Pb-n1 synthesized from SCS and NEIT approaches. **b.** Optical images of Pb-n1 samples synthesized from SCS and NEIT approaches for PL characterization. **c.** PL spectra of Pb-n2 synthesized from SCS and NEIT approaches. **d.** Optical images of Pb-n2 samples synthesized from SCS and NEIT approaches for PL characterization. **e.** PL spectra of Pb-n3 synthesized from SCS and NEIT approaches. **f.** Optical images of Pb-n3 samples synthesized from SCS and NEIT approaches for PL characterization. For each characterized region (area 1-3) in **b, d,** and **f**, the corresponding measured transmittance (T), reflectance (R), calculated absorptance (A) and calculated thickness (d) values are listed on the right, determined according to the relationship A=1-R-T. Substituting the transmittance at 454 nm into the formula established in Figs. 21-23 will obtain the corresponding flake thickness d.

***Revision:*** Figs. R19-24 are incorporated in the revised Supplementary Information as Supplementary Figs. 15-19, and 14, respectively.

We have incorporated the following details into the PLQY testing method of Supplementary Information (1. Supplementary Characterizations, Page 5): All PLQY measurements were performed on mechanically exfoliated nanoflakes. The thickness of each flake was fitted using an established thickness-transmittance calibration curve. Both NEIT and SCS flakes tested possessed comparable nanometer-scale thicknesses.

The following texts (highlighted in yellow) are included in Line 209 of the revised main manuscript: To ensure a fair comparison, these measurements were performed on mechanically exfoliated nanoflakes with similar thicknesses. Furthermore, both polarization-resolved and angle-resolved PL spectroscopy confirmed the highly isotropic emission profile of these flakes (Supplementary Figs. 15-19).

(Line 717) Angle-resolved PL measurements were performed using an Angle-Resolved Micro-Spectrometer (Shanghai Ideaoptics Corp., Ltd). A 405 nm laser was used as the excitation source, and the PL emission signals were collected over an angular range from -60° to +60°.

(Line 720) Polarization-resolved PL spectroscopy was conducted on a WITec Alpha300RAS confocal Raman microscopy system. A linearly polarized light was used as the excitation source. A polarizer was inserted into the signal collection path. The emission signals were recorded at different polarization angles by rotating the polarizer with a step size of 15°.

5. On page 7: the authors attributed the claimed lasing to reduced trap density. However, as the authors stated, Auger recombination, rather than trap-mediated non-radiative recombination, is the main limitation. Under such high power excitation, traps are likely saturated, so attributing threshold behavior primarily to trap density is not justified. Instead, low lasing threshold would be strongly affected by unintentional cavity effects. Thickness of the sample measured should be reported.

-The authors claimed lasing, rather than alternative explanations such as ASE or random lasing, by narrowed peaks, polarization measurements. However, since the 2D perovskite is anisotropic material, polarization is not enough to conclusively establish lasing. Additional evidence of coherence and a feedback mechanism/mode structure is required to refer it as lasing.

-The authors reported FWHM of 0.18 nm. From Figure 1h, it seems the redder part has an overall Gaussian-like shape with FWHM ~2nm, and many periodic fringing features. Perhaps the fringes are where the authors derived the FWHM 0.18nm from. Are the fringes (similarly in Fig. 1h and Fig. S6) an artefact of the spectrometer when averaging over multiple pulses, or from interference in the optical path including windows of the cryostat? By removing the fringes, the feature could be more sensibly explained by ASE, since there was no designed cavity.

***Response:*** We sincerely thank you for this profound physical insight. Under high excitation fluences required for lasing, trap states are saturated, and Auger recombination dominates carrier loss. Our prolonged TRPL (larger $\tau_2$) and SCLC data

indicate a lower bulk trap density in NEIT-grown crystals (*Nat Energy* **2016**, 1, 16149 *J. Am. Chem. Soc.* **2020**, 142, 16254-16264), which is beneficial to lower the lasing threshold by mitigating defect-mediated Auger processes (*Chem. Eng. J.* **2025**, 525, 169849). This improvement facilitates lasing by suppressing non-radiative heating and scattering losses. 1) By minimizing defect-mediated Auger processes, NEIT crystals effectively reduce carrier loss, facilitating population inversion. 2) Highly defective SCS crystals suffer from rapid non-radiative heating and strong optical scattering, which lead to thermal degradation before reaching the threshold and only yield amplified spontaneous emission (ASE) (Fig. R25). The low defect density in NEIT crystals ensures the efficient optical gain. Spectral analysis of 7 randomly selected regions on NEIT flakes (thicknesses of 1249-2441 nm) consistently demonstrated strong lasing (Fig. R26).

As demonstrated in in-plane polarization and angle-resolved PL measurements (Figs. R19 and R20), the PL emission of exfoliated flakes is highly isotropic. Therefore, the emergence of linear polarization above lasing threshold is a classic signature of stimulated emission. Furthermore, coherence time measurements were conducted. The extracted coherence times ($\tau_c$) are 73.8 fs, 332.2 fs, and 424.8 fs for Pb-n1, Pb-n2, and Pb-n3, respectively (Fig. R27).

To exclude optical path artifacts, identical control experiments were conducted on conventional SCS-grown flakes. These SCS samples exhibited ASE without sharp fringing features, further validating the lasing characteristics from our NEIT samples.

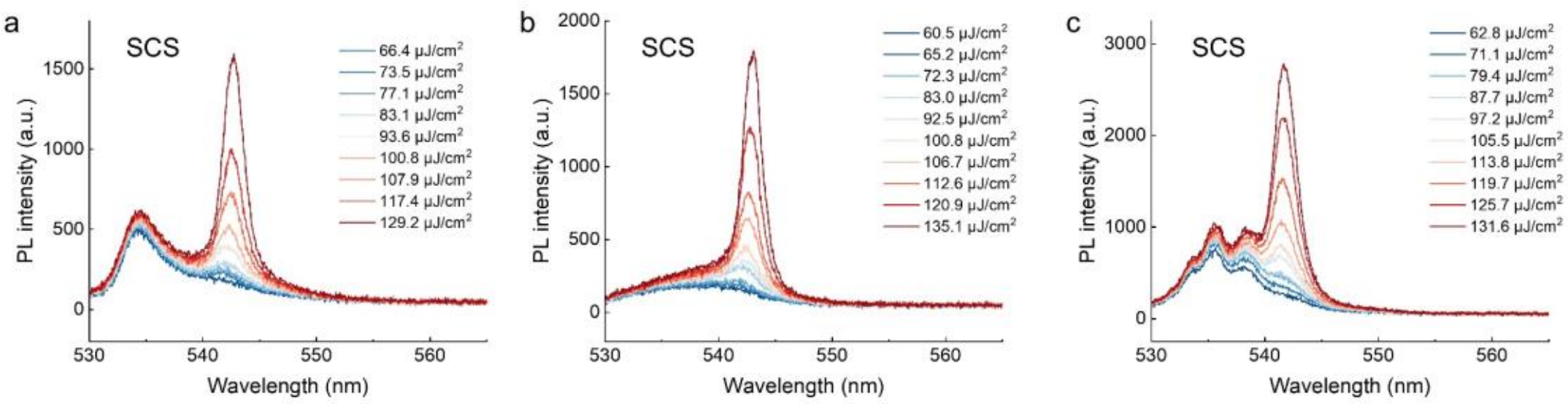


**Fig. R25. PL spectra of multiple Pb-n1 exfoliated flakes synthesized from SCS growth, including a.** Sample 1# with the ump fluence increasing from 66.4 to 129.2 μJ/cm$^2$, the lasing threshold is ~93.6 μJ/cm$^2$. **b.** Sample 2# with the pump fluence increasing from 60.5 to 135.1 μJ/cm$^2$, the lasing threshold is ~100.8 μJ/cm$^2$. **c.** Sample 3# with the pump fluence increasing from 62.8 to 131.6 μJ/cm$^2$, the lasing threshold is ~97.2 μJ/cm$^2$. (this figure is also incorporated in our response to 3$^{nd}$ comment of Reviewer 1 as Fig. R9).

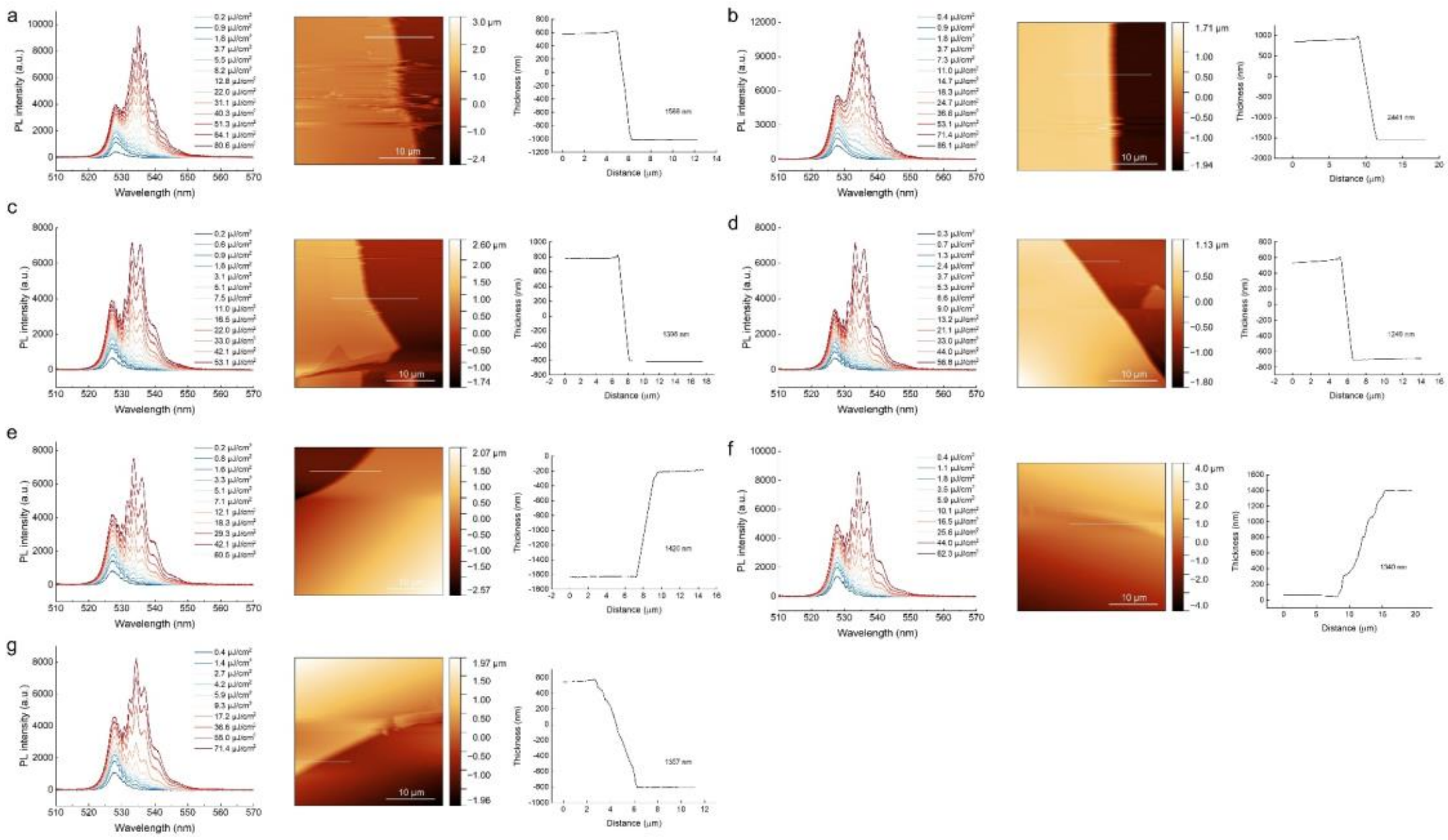

**Fig. R26. Lasing spectra and corresponding thickness of Pb-n1 exfoliated flakes synthesized from NEIT growth, including a.** Sample 1# with the pump fluence increasing from 0.2 to 80.6 μJ/cm$^2$, the lasing threshold is ~12.8 μJ/cm$^2$ and the corresponding thickness is ~1588 nm. **b.** Sample 2# with the pump fluence increasing from 0.4 to 86.1 μJ/cm$^2$, the lasing threshold is ~14.7 μJ/cm$^2$ and the corresponding thickness is ~2441 nm. **c.** Sample 3# with the pump fluence increasing from 0.2 to 53.1 μJ/cm$^2$, the lasing threshold is ~ 11.0 μJ/cm$^2$ and the corresponding thickness is ~1396 nm. **d.** Sample 4# with the pump fluence increasing from 0.3 to 56.8 μJ/cm$^2$, the lasing threshold is ~9.0 μJ/cm$^2$ and the corresponding thickness is ~1249 nm. **e.** Sample 5# with the pump fluence increasing from 0.2 to 60.5 μJ/cm$^2$, the lasing threshold is ~ 12.1 μJ/cm$^2$ and the corresponding thickness is ~1420 nm. **f.** Sample 6# with the pump fluence increasing from 0.4 to 62.3 μJ/cm$^2$, the lasing threshold is ~16.5 μJ/cm$^2$ and the corresponding thickness is ~1340 nm. **g.** Sample 7# with the pump fluence increasing from 0.4 to 71.4 μJ/cm$^2$, the lasing threshold at ~17.2 μJ/cm$^2$ and the corresponding thickness is ~1357 nm.

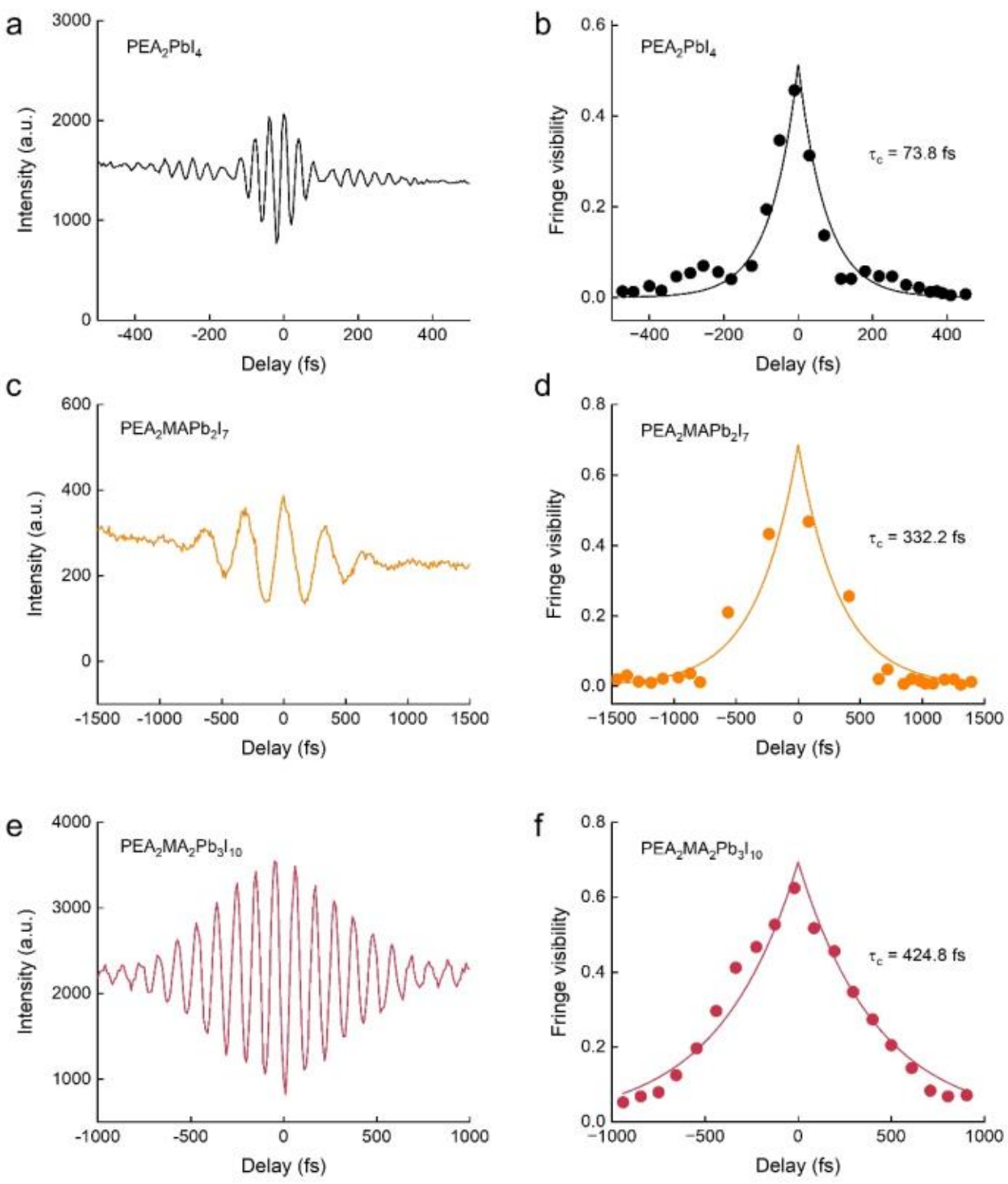


**Fig. R27. Coherence time measurements of the exfoliated Pb-n1, Pb-n2, and Pb-n3 flakes under lasing conditions. a, c, e,** Interference fringe patterns (interferograms) of the emission from NEIT-grown Pb-n1 (a), Pb-n2 (c), and Pb-n3 (e) flakes measured by a Michelson interferometer above the lasing threshold at 80 K. The distinct oscillatory patterns indicate the strong phase correlation of the emitted photon wavepackets. **b, d, f,** The corresponding fringe visibility as a function of the time delay for Pb-n1 (b), Pb-n2 (d), and Pb-n3 (f). The solid lines represent the fitting curves used to extract the temporal coherence times ($\tau_c$) (*Nature* **2023**, 617, 79–85). The coherence times are quantified as 73.8 fs, 332.2 fs, and 424.8 fs for n=1, 2, and 3 perovskites, respectively. These extended coherence times and clear interference profiles provide unambiguous evidence of the temporal coherence of the optical emission, proving the true lasing.

***Revision:*** Figs. R25-R27 are incorporated in the revised Supplementary Information as Supplementary Figs. 22, 20 and 24, respectively.

The following texts (highlighted in yellow) are included in Line 224 of the revised main manuscript: Under the high optical excitation fluences required for lasing, trap states are generally saturated, and Auger recombination dominates the carrier loss[46,47]. Nevertheless, the reduced trap density in NEIT-grown crystals inherently could lower the defect-mediated Auger recombination rate and minimizes non-radiative thermal degradation, effectively facilitating population inversion[48]. We conducted measurements across randomly selected regions with varying flake thicknesses (Supplementary Fig. 20). The NEIT-grown flakes exhibited multimode lasing with threshold of ~15 μJ/cm$^2$ (Fig. 1h and Supplementary Fig. 21). In contrast, exfoliated flakes from SCS-grown crystals tested under identical conditions only displayed amplified spontaneous emission (ASE) with a significantly higher threshold (~100 μJ/cm$^2$) (Supplementary Fig. 22).

48. Cai, Q. *et al.* Unravel the impacts of deep-level defects on Auger recombination in nanocrystals for realizing low-threshold blue amplified spontaneous emission. *Chem. Eng. J.* **525**, 169849 (2025).

(Line 240) Furthermore, we observed distinct interference fringes and extracted coherence times ($\tau_c$) of 73.8 fs, 332.2 fs, and 424.8 fs for the Pb-n1, n2, and n3 perovskites, respectively (Supplementary Fig. 24). The temporal coherence signatures, combined with linear polarization above the threshold, confirm the lasing emission.
(Line 767) The coherence time of the lasing emission was measured using a custom-built Michelson interferometer optical setup. The resulting interferometric signals were collected and detected by a Princeton Instruments (PI) spectrometer.

6. Figure 4e-f are hard to follow and the authors should label the crystal orientation. Figure 4e seems to be cross-section of the vdWs heterostructure. How was the cross-section section prepared? In line 388 the authors also mentioned SEM of lateral heterostructure but no figures are referred to. In figure S20b, the EDS spectra for the Pb and Sn regions should be shown to confirm that they are not alloyed.

***Response:*** We appreciate the constructive suggestions to improve the clarity of our heterostructure characterization.
1) The [001] direction of the Pb-Sn, n2 vdW heterostructure are labeled in Fig. 4e (Fig. R28).
2) The cross-section of the Pb, n2-n1 vdW heterostructure was prepared by mechanical cut the heterostructure with a sharp blade perpendicular to the (00k) plane.
3) The reference to the SEM image of Pb-Sn, n2 lateral heterostructures has been updated in the revised manuscript, and the image is included as Supplementary Fig. S41b.
4) To confirm that the Pb-n2 and Sn-n2 regions are not alloyed in the lateral heterostructures, we performed EDS line scans (Fig. R29). The elemental profiles of Pb and Sn show a sharp transition at the boundary, with no obvious intermixing zone.

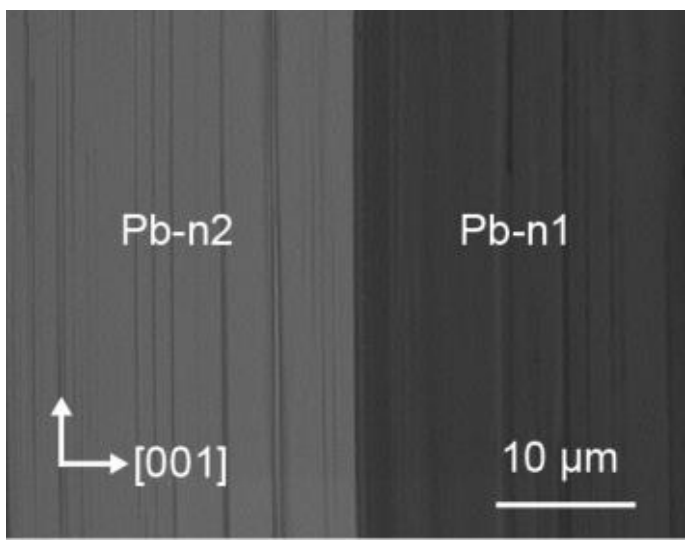


**Fig. R28. SEM image of Pb, n2-n1 vdW heterostructure.**

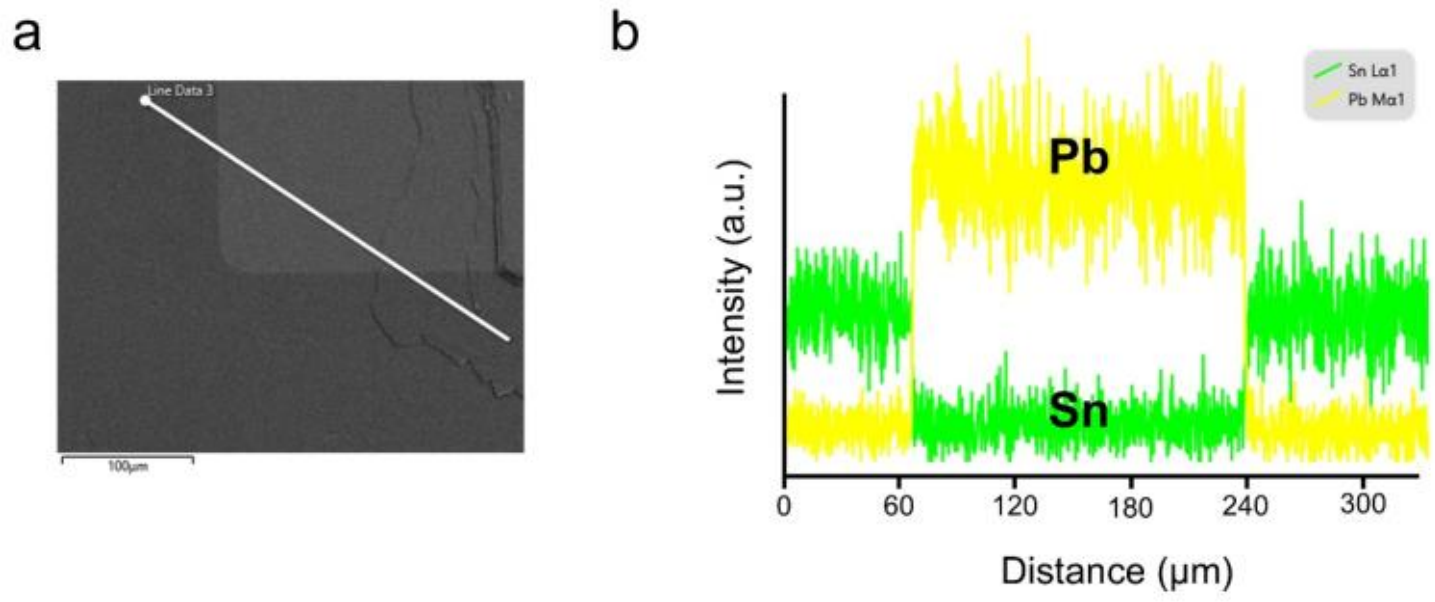


**Fig. R29. SEM image (a) of the Pb-Sn, n2 lateral heterostructure and the corresponding EDS line scan profiles (b) of Pb and Sn elements along the path indicated in (a).**

***Revision:*** We have labeled the [001] direction of the Pb-Sn, n2 lateral heterostructure in Fig. 4e, and also included the EDS line scan results in Supplementary Fig. 41.
The following texts (highlighted in yellow) are included in Line 439 of the revised main manuscript: In addition, the sharp interface was corroborated in lateral heterostructures by clear SEM domain contrast and energy-dispersive X-ray spectroscopy (EDS) line scans. The EDS profiles show an abrupt transition between Pb and Sn domains, with no discernible intermixing zone, definitively ruling out interfacial alloying (Supplementary Fig. 41).
(line 749) For the cross-sectional analysis, heterostructures were mechanically cut perpendicular to the (00k) plane using a sharp blade.

7. There is a claimed lattice mismatch of <1.6% between the Pb-Sn lateral heterojunction. The author should explain how the heterostructure accommodate the high mismatch without cracking or significant defects that affect photodetector performance.

***Response:*** Thanks for your suggestion. The Pb-Sn lateral heterostructure accommodates the ~1.6% lattice mismatch without significant performance degradation owing to following key factors: Firstly, the ionic nature of halide perovskites results in a soft crystal lattice (*Matter* **2021**, 4, 3874-3896). This structural flexibility offers higher tolerance to lattice mismatch than conventional covalent semiconductors, accommodating strain without cracking. Secondly, as reported in the literature, perovskite lateral heterostructure with larger mismatches (e.g., 5-6% in $(2T)_2PbI_4$ and $(2T)_2PbBr_4$) relax strain via interfacial edge dislocations and out-of-plane ripple formation (*Nature* **2020**, 580, 614-620). In this regard, the ~1.6% mismatch in our Pb-Sn system is reasonable, which would not induce cracking or significant defects that affect photodetector performance.

.

***Revision:*** The following texts (highlighted in yellow) are included in Line 425 of the revised main manuscript: Benefiting from the soft ionic lattice of halide perovskites, the ~1.6% lattice mismatch is accommodated without cracking or significant defects that affect device performance[6].

8. The authors reported vdWs epitaxy and lateral epitaxy in the heterostructure. More crystallographic evidence is needed to support the aligned in-plane orientation. A single peak of (110) is shown for the vdWs heterostructure but not for lateral heterostructure. Importantly, the single (110) peak cannot exclude certain misoriention when the Bragg peak from the second crystal could be rotated such that they are not accessible in the current 1D line scan geometry. Reciprocal space maps showing in-plane signals from both layers can confirm epitaxy since the two layers do have different lattice parameters.

***Response:*** We fully agree that 2D reciprocal space mapping (RSM) is critical to evaluate the in-plane epitaxial registry.

We have now acquired high-resolution 1D XRD and 2D RSM of the (110) plane for the lateral heterostructure. Because the (110) plane lies in an inaccessible region for conventional coplanar geometry relative to the main (001) orientation, a skew scan geometry was utilized to facilitate the measurement (Fig. R30). Consequently, the acquired data need to be transformed. The 1D XRD pattern of the (110) plane resolves a peak at 2θ~14.35° (Pb-n2) and a secondary peak at 2θ~14.42° (Sn-n2), closely matching the theoretical difference in their lattice parameters. Furthermore, in the (110) RSM, we successfully capture distinct reflections from both the Pb-n2 and Sn-n2 domains within the identical reciprocal scattering plane. The simultaneous observation of both (110) reflections in the same coordinate frame provides evidence that the Sn-n2 lattice likely maintains an orientation with the Pb-n2.

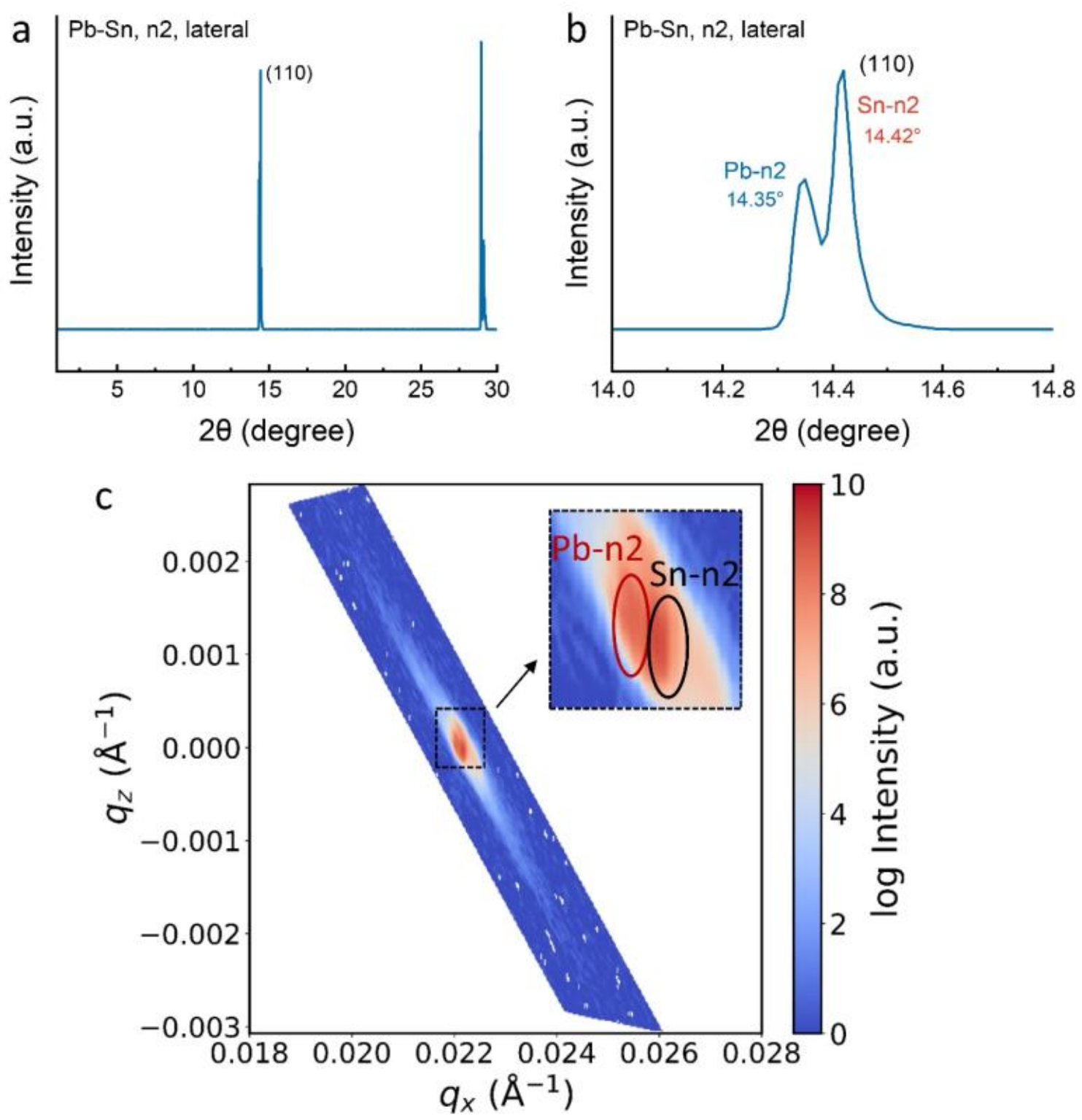


**Fig. R30. Crystallographic evidence for the in-plane orientational epitaxy of Pb-Sn, n2 lateral heterostructure. a.** High-resolution 1D XRD pattern of Pb-Sn, n2 lateral heterostructure in (110)

plane. **b.** The magnified region of a at around the (110) diffraction peak reveals 2 distinct peaks corresponding to Pb-n2 and Sn-n2. **c.** The corresponding 2D reciprocal space map (RSM) of the (110) plane  showing distinct reflections.

***Revision:*** Fig. R30 is incorporated in the revised Supplementary Information as Supplementary Fig. 42.
The following texts (highlighted in yellow) are included in Line 453 of the revised main manuscript: Furthermore, HRXRD 1D scan and 2D reciprocal space mapping (RSM) of the (110) plane reveal distinct reflections from both Pb-n2 and Sn-n2 domains, verifying the aligned in-plane orientation of the Pb-Sn, n2 lateral heterostructure (Fig. 4g and Supplementary Fig. 42).

**Format and terminology:**

**1.** The manuscript lacks sufficient methodological detail for reproducibility of characterizations. For example, rocking curve measurements including beam size, slit settings and beam optics; accelerating voltage for SEM and EDS; excitation power of PLQY, device geometry of the SCLC electron-only device; etc.

***Response:*** Thanks for your suggestion. Following your suggestions, we have included all the specified parameters and instrument settings in the revised main manuscript.
***Revision:*** (Line 700): All high-resolution X-ray measurements, including rocking curve, ~~Grazing-incidence XRD (GIXRD) and~~ 2θ/ω scan, reciprocal space mapping (RSM), and X-ray reflectivity (XRR) were conducted on a Bruker D8 Discover diffractometer equipped with a point-focused Cu Kα source (beam diameter: 0.5 mm).
(Line 746): SEM imaging was conducted at an accelerating voltage of 3 kV with a probe current of 200 pA, while EDS mapping was performed at a higher accelerating voltage of 10 kV and probe current of 500 pA to ensure sufficient X-ray excitation.
(Line 772): Trap state densities were evaluated using SCLC measurements in electron-only devices with a device structure of Ag (120nm) /BCP (6nm) /$C_{60}$ (35nm) /Perovskite /$C_{60}$ (35nm) /BCP (6nm) /Ag (120nm).
The PLQY test information has been added to the caption of Supplementary Fig. 14: PL intensity of 2D perovskites excited under identical laser fluence (320 nW, 40 MHz, ~300 nJ/cm$^2$).

2. In Supplementary Fig. 1, enlarging a crystal should be described as seeded growth, not epitaxial growth.

***Response and revision:*** We thank you for pointing out this terminology inaccuracy. We have changed epitaxial growth to seeded growth in Supplementary Fig. 8.

3. On page 2 line 61, suggest change 'bright' to 'efficient'. Confined perovskite systems are advantageous in efficient LEDs, but to achieve high brightness, these systems (and the reference cited) are less competitive in maintaining high efficiency at high current

density.

***Response and Revision:*** Thanks for your suggestion. We have accordingly changed “bright” to “efficient” in the revised main manuscript:

(Line 71) directly limiting their application in high-efficiency solar cells and ~~bright~~ efficient LEDs.

4. Supplementary Fig.4b, the y-axis label should not be PL intensity if the measurement is reflection and transmission.

***Response and Revision:*** We thank you for catching this labeling error. We have corrected the y-axis label in Supplementary Fig. 4b to Intensity (a.u.) in the revised Supplementary Fig. 13.

5. Supplementary Fig 22c, the reported linear dynamic range is not supported. In linear dynamic range, the photoresponsivity is constant against input power, instead here, photoresponsivity decreased from the very beginning.

***Response:*** Thanks for your suggestion. We agree that the determination of linear dynamic range (LDR) requires a constant photoresponsivity at low incident powers. The decreasing trend of photoresponsivity in our initial large-area photodetector device likely originates from an electrical edge effect (*Joule* **2022**, 6, 1904-1917).
To accurately evaluate the intrinsic LDR and eliminate this electrical edge effect, we fabricated mechanically exfoliated Pb-Sn, n2 lateral heterostructure micro-size devices. As shown in Fig. R31, the photoresponsivity remains nearly constant across a wide-range illumination from $2.2\times10^{-4}$ to 20.7 mW/cm2, which is used to extract the LDR value.

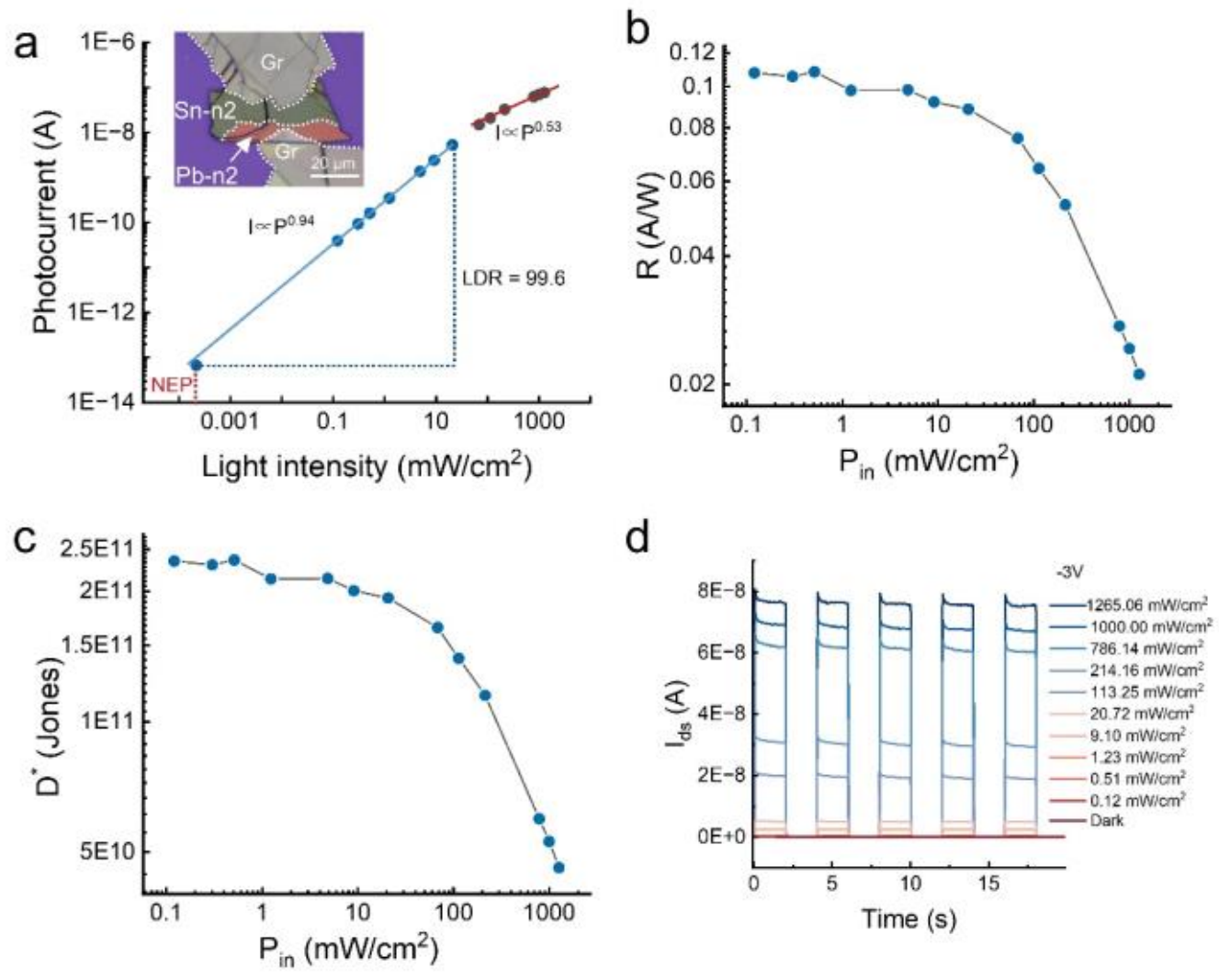


**Fig. R31. Photodetection performance and linear dynamic range of the micro-size exfoliated lateral heterostructure. All measurements were conducted under a bias voltage of -3 V. a.** The

illumination power-dependent photocurrents. The device exhibits a linear dynamic range ($I \propto P^{0.94}$) yielding an LDR of 99.6 dB, followed by a sublinear transition ($I \propto P^{0.53}$) at high power densities. **b, c,** Responsivity (R) and specific detectivity (D*) as functions of irradiation power intensity ($P_{in}$) at –3 V bias. **d.** Photocurrent switching behavior under illumination power densities ranging from 0.12 to 1265.06 mW/cm$^2$, recorded at a bias voltage of –3 V (Pb-n2 grounded, Sn-n2 biased).

***Revision:*** Fig. R31 is incorporated in the revised Supplementary Information as Supplementary Fig. 44.

Reviewer #3 (Remarks to the Author):

This work reports a near-equilibrium isothermal (NEIT) growth strategy for two-dimensional perovskite single crystals, which achieves ultra-low nucleation density and produces centimeter-sized single crystals of $PEA_2PbI_4$ (Pb-n1), $PEA_2MAPb_2I_7$ (Pb-n2), and $PEA_2MA_2Pb_3I_{10}$ (Pb-n3). The NEIT strategy establishes a versatile and scalable platform for synthesizing high-quality, large-area 2D perovskite single crystals and their heterostructures, representing a significant advance in the material foundation for quantum-confined semiconductor devices. The findings are compelling and the methodology is highly innovative. However, before the acceptance in Nature Communications, the authors are required to make minor revisions to address the following comments to further strengthen the rigor and impact of the work.

1. In Fig. 1g, the PLQY is plotted on a normalized scale (0–1), while the text reports values as percentages (e.g., 95 ± 2%). The y-axis of Fig. 1g should be relabeled to 0–100% to maintain consistency and avoid reader confusion.

***Response and revision:*** We thank you for pointing out this inconsistency. We have revised the y-axis of Fig. 1g in the main manuscript to display a 0-100% scale, aligning with the percentage values (e.g., 95 ± 2%).

2. The Pb-n2 crystals grown by the NEIT method exhibit high transparency in the presented images. As optical transparency correlates directly with crystalline quality and long-range uniformity, it is highly recommended to provide a quantitative comparison of optical clarity, for example via transmittance spectroscopy, between crystals synthesized using the NEIT and SCS approaches.

***Response:*** Thanks for your suggestion. We completely agree that macroscopic optical transparency is a highly reliable and direct indicator of internal crystalline quality, as it is directly correlated with the suppression of internal optical scattering centers. Transmittance spectroscopy was performed on distinct regions of both NEIT- and SCS-grown Pb-n2 single crystal. The NEIT-grown Pb-n2 crystal exhibits a consistently high transmittance of approximately 85% with good spatial uniformity across all 23 sampled spots, despite a thickness of 619 μm. The SCS-grown counterpart yields a lower transmittance (47%-79%) and shows significant variability among the 13 tested regions, even though the sample is 68 μm thick (Fig. R32).

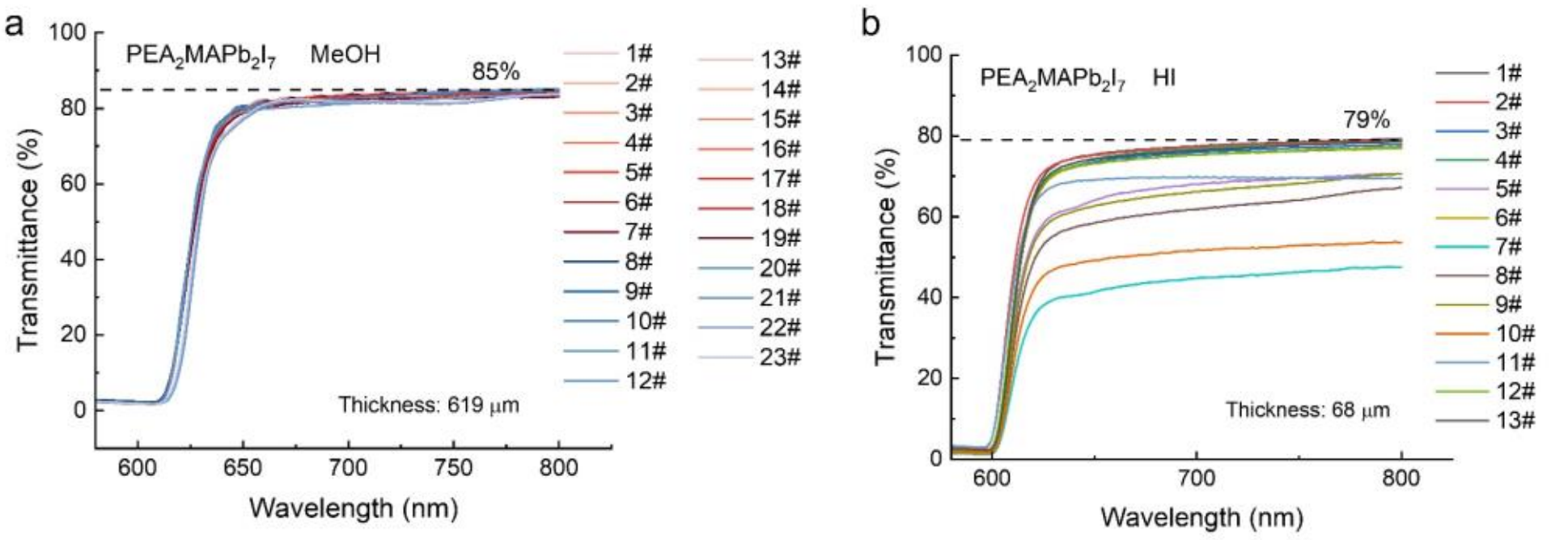


**Fig. R32. Optical transparency of NEIT- and SCS-grown Pb-n2 single crystals. a.** Transmittance spectra of a NEIT-grown Pb-n2 single crystal with a measured thickness of 619 μm. **b.** Transmittance spectra of an SCS-grown Pb-n2 single crystal with a thickness of 68 μm.

***Revision:*** Fig. R32 is incorporated in the revised Supplementary Information as Supplementary Fig. 10.

The following texts (highlighted in yellow) are included in Line 182 of the revised main manuscript: Quantitative transmittance spectroscopy confirms the superior macroscopic quality of the NEIT-grown crystals. The NEIT-grown Pb-n2 crystal maintains consistent transmittance of ~85% with good spatial uniformity (thickness of 619 μm), compared to highly variable transmittance (47%–79%) for the SCS-grown counterpart (thickness of 68 μm) (Supplementary Fig. 10).

3. In Fig. 1e, the FWHM for the (001) diffraction peak is labeled, but the corresponding value for the (110) peak is absent. For completeness, the (110) peak FWHM should also be indicated in the figure or caption.

***Response and revision:*** Thanks for your suggestion. We have updated Fig. 1e in the revised manuscript to explicitly label the FWHM values for the (110) diffraction peaks.

4. In Fig. 2, the carrier lifetime of the Pb-n1, n2 and n3 single crystals synthesized from NEIT and SCS approaches are presented as single measurements for each crystal. Reporting results from multiple samples or repeated measurements would enhance the statistical reliability of these data.

***Response:*** Thanks for your suggestion. We fully agree that providing statistical data from multiple measurements is essential to establish the robustness and reproducibility of our findings. To confirm the reliability of the prolonged carrier lifetimes, we have performed repeated time-resolved photoluminescence (TRPL) measurements on multiple randomly selected flakes exfoliated from Pb-n1, Pb-n2, and Pb-n3 crystals (both NEIT and SCS grown). As shown in Figs. R33-R35, the TRPL decay kinetics are highly reproducible across multiple tests. These repeated measurements confirm that the prolonged carrier lifetime in NEIT-grown crystals is not an isolated or stochastic occurrence.

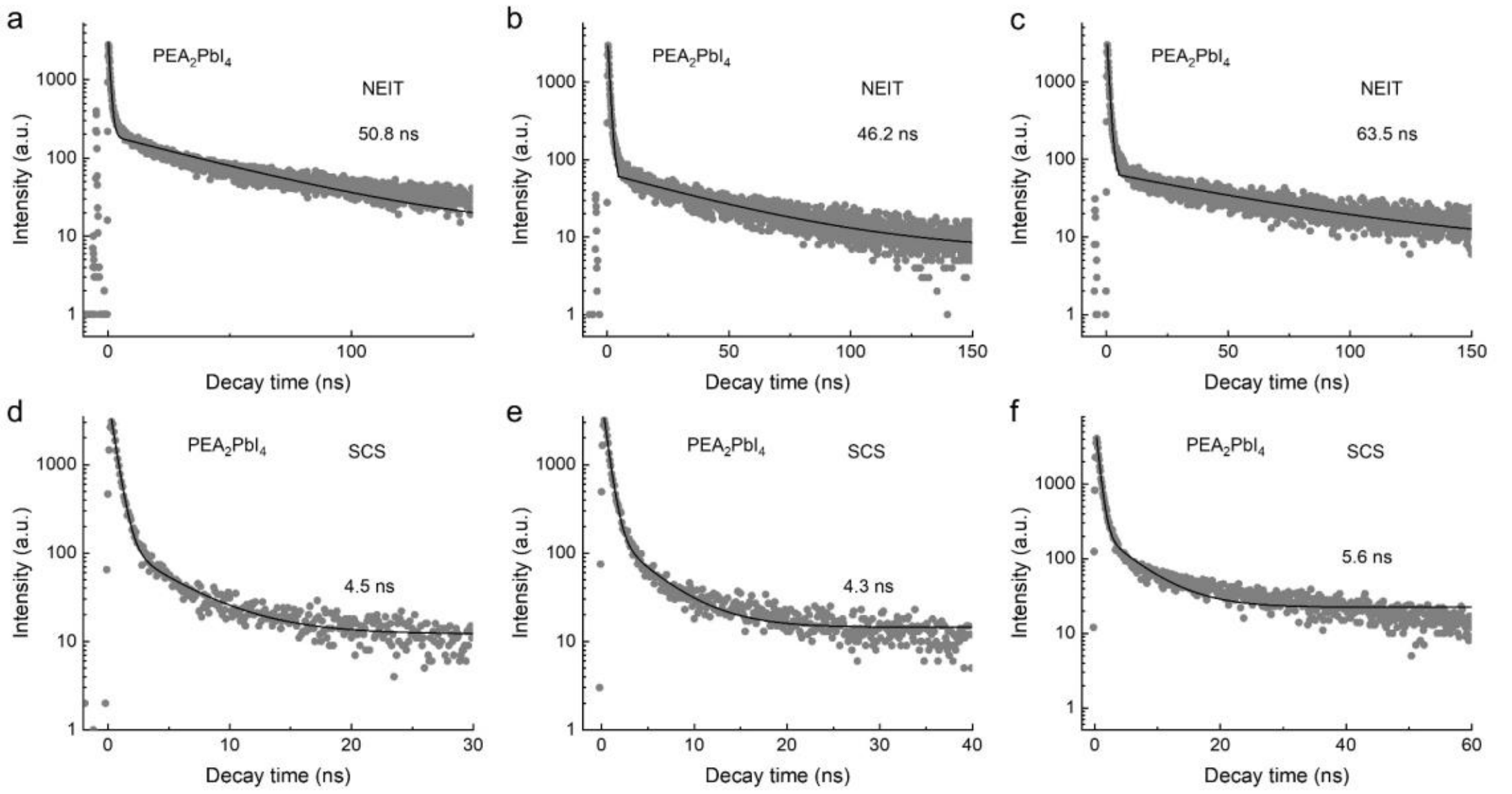


**Fig. R33. Reproducibility of carrier lifetime measurements for Pb-n1 single crystals. a-c.** Repeated TRPL decay spectra and bi-exponential fitting curves of Pb-n1 flakes exfoliated from NEIT-grown single crystals, yielding consistently prolonged $\tau_2$ lifetimes of 50.8 ns, 46.2 ns, and 63.5 ns. **d-f.** Repeated TRPL decay spectra of Pb-n1 flakes from conventional SCS-grown crystals under identical testing conditions, showing short lifetimes of 4.5 ns, 4.3 ns, and 5.6 ns.

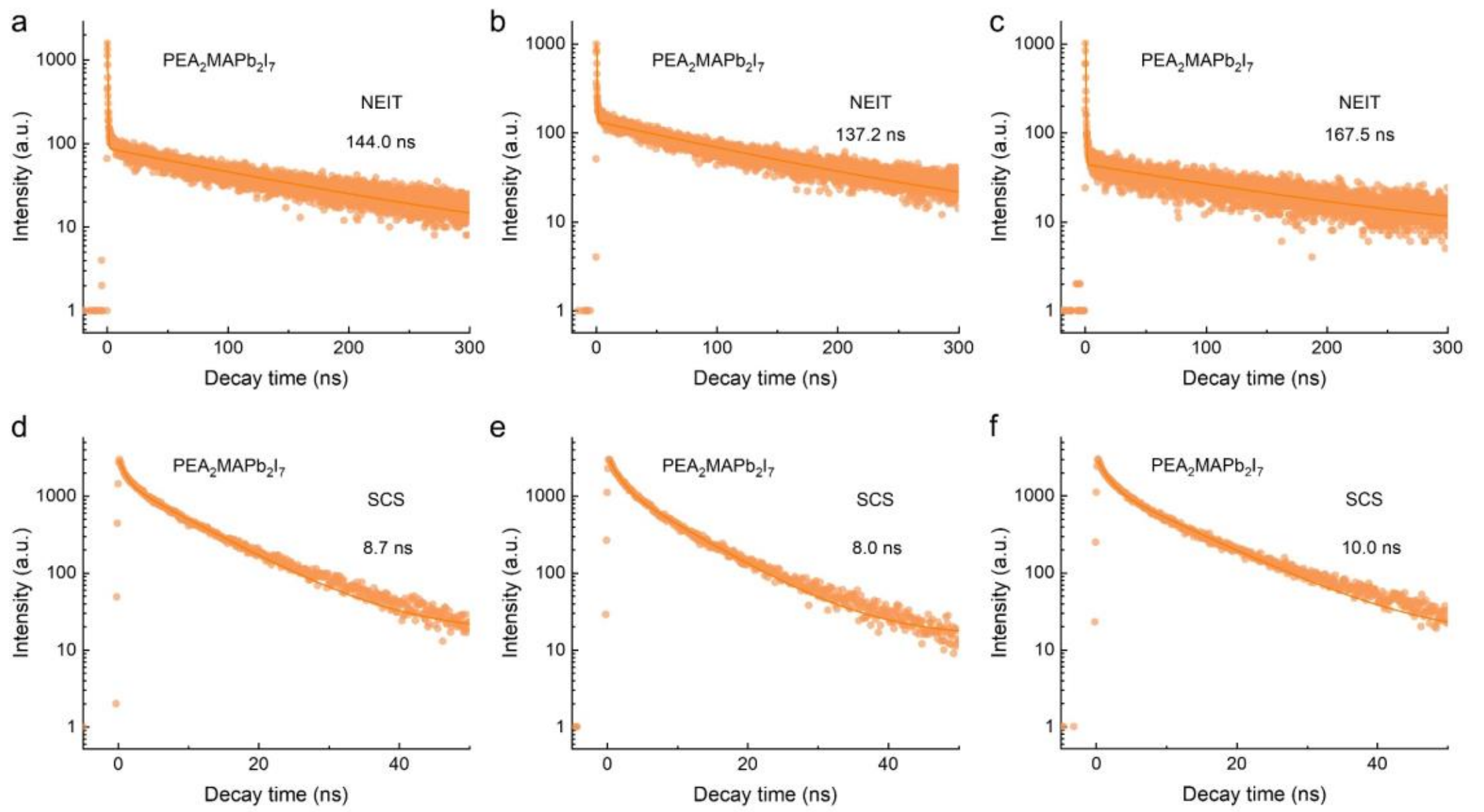


**Fig. R34. Reproducibility of carrier lifetime measurements for Pb-n2 single crystals. a-c.** Repeated TRPL decay spectra and fitting curves of NEIT-grown Pb-n2 flakes, demonstrating long $\tau_2$ lifetimes of 144.0 ns, 137.2 ns, and 167.5 ns. **d-f.** Repeated TRPL decay spectra of SCS-grown Pb-n2 flakes, showing consistently short lifetimes of 8.7 ns, 8.0 ns, and 10.0 ns.

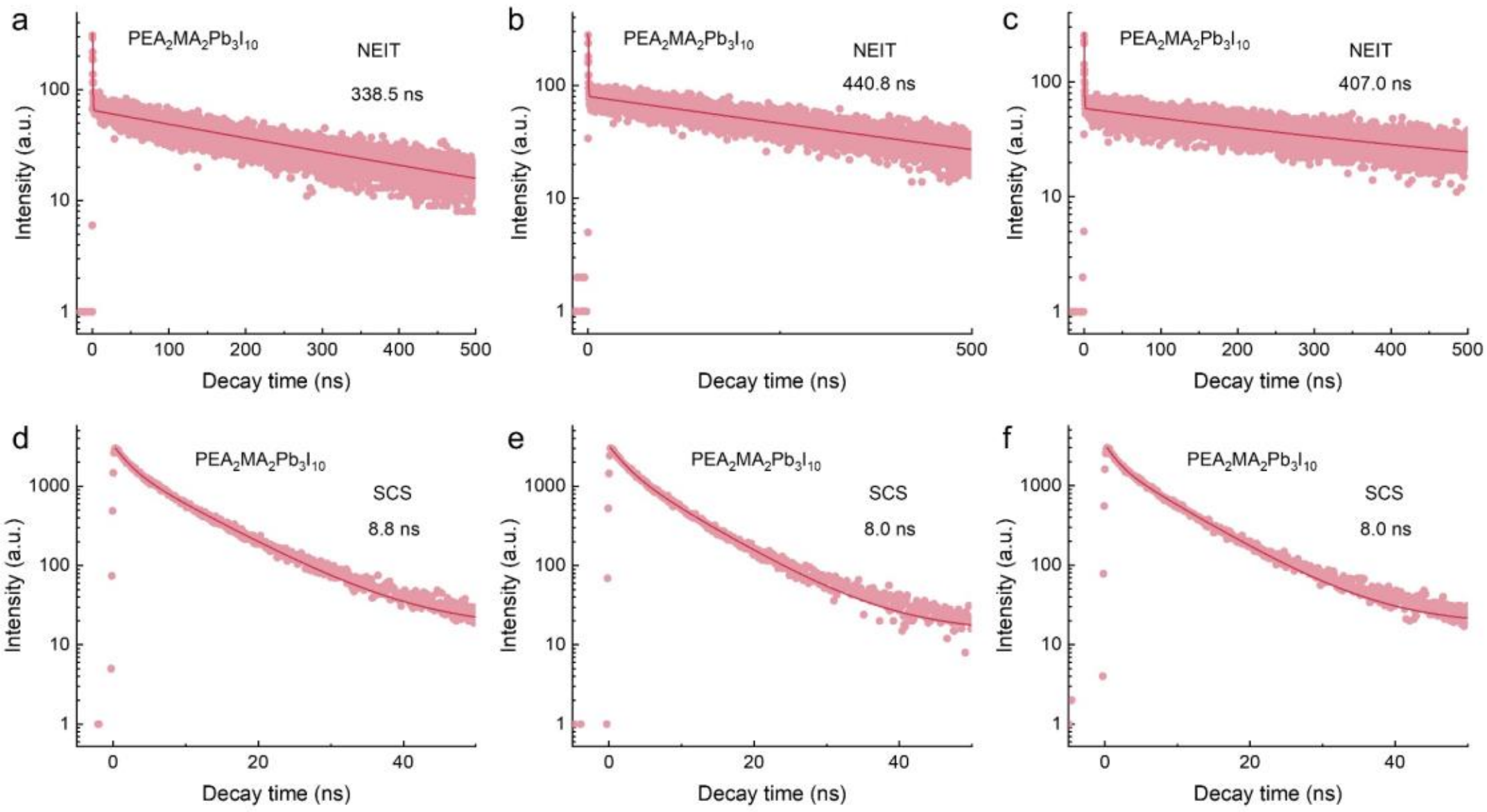


**Fig. R35. Reproducibility of carrier lifetime measurements for Pb-n3 single crystals. a-c.** Repeated TRPL decay spectra and fitting curves of NEIT-grown Pb-n3 flakes, demonstrating long $\tau_2$ lifetimes of 338.5 ns, 440.8 ns, and 407.0 ns. **d-f.** Repeated TRPL decay spectra of SCS-grown Pb-n3 flakes, showing short lifetimes of 8.8 ns, 8.0 ns, and 8.0 ns.

***Revision:*** Figs. R33-R35 are incorporated in the revised Supplementary Information as Supplementary Figs. 25-27.

5. A discrepancy is noted between the lifetime of Pb-n2 reported in Fig. 2b (151.9 ns) and that from FLIM in Fig. 2e (66.7 ns). The origin of this difference, whether due to measurement conditions or regional variation, should be clarified and discussed.

***Response:*** We sincerely thank you for the comment. The lifetime difference between TRPL and FLIM arises from distinct excitation power densities. To extract the intrinsic lifetime, TRPL was performed using an expanded laser spot (1000 $\mu m^2$), maintaining a low excitation fluence of ~5 $nJ/cm^2$. FLIM utilizes a microscope-focused spot (with a repetition frequency of 4 MHz, laser power of $5.4\times10^{-3}$ μW, and calculated Airy disk radius of 218.34 nm), resulting in a much higher power density of ~900 $nJ/cm^2$. As demonstrated by our power-dependent TRPL measurements (Fig. 2d, Supplementary Fig. 8), the carrier decay kinetics are highly sensitive to excitation fluence. The increased power density accelerates the decay kinetics, thereby reducing the lifetime.

***Revision:*** The following texts (highlighted in yellow) are included in Line 286 of the revised main manuscript: It is worth noting that the shorter lifetime measured by FLIM (~66.7 ns) compared to TRPL (~151.9 ns) arises from the different excitation power densities (~900 $nJ/cm^2$ in FLIM versus ~5 $nJ/cm^2$ in TRPL).
(Line 740) The samples were excited using a 405 nm laser with a repetition rate of 4 MHz and laser power of $5.4 \times 10^{-3}$ μW. The diffraction-limited Airy disk radius was calculated to be 218.34 nm, yielding a high localized excitation fluence of ~900 $nJ/cm^2$.

**A List of All Revisions and Responses to Referees' Comments for *Nature Communications* Manuscript: NCOMMS-26-000453A**

**Note**: The revision is yellow highlighted in the main text. Response to the referees' comments is marked in blue.

**REVIEWER COMMENTS**

Reviewer #1 (Remarks to the Author):

The response letter is well-organized, scientifically detailed, and addresses most reviewer concerns substantively. Particularly, DLS tracking experiments are highly persuasive evidence for the metastable zone difference between HI and MeOH systems. One remaining issue to address is that authors should try to capture the gradual increase of particle size under DLS (during cooling in SLS method and during steady growth at RT in MeOH system.) The current DLS results only capture the beginning and the end.

***Response:*** We appreciate your insightful comment. We agree that capturing the intermediate dynamic states during crystallization is crucial. To address this, we have performed in-situ continuous DLS tracking for both the Pb-n2, SCS and NEIT systems (Figs. R1 and R2).

As shown in Fig. R1, we continuously monitored the Pb-n2, SCS solution immediately during natural cooling. The DLS signal changes rapidly, reflecting the burst nucleation behavior associated with the narrow metastable zone in the SCS system. The transition from a clear solution to massive crystallization occurs rapidly. Two independent experiments exhibit a consistent trend of rapid changes in DLS signal, revealing high-density occurrence of rapid nucleation (Fig. R1b and d).

For the NEIT system (Fig. R2), we conducted undisturbed in-situ tracking (the solution remained inside the instrument). During the initial cooling (first 20 min) and the subsequent prolonged room temperature maintenance stage (monitored for up to 36 h in Fig. R2a, and 660 min in Fig. R2c), the precursor size remained at the molecular scale (~1-2 nm). We did not observe a gradual size increase into intermediate clusters prior to macroscopic crystallization. The macroscopic scattering signals (>1000 nm) observed in our previous round of data were likely induced by mechanical disturbance. In our previous quasi-in situ setup, the cuvettes were repeatedly moved in and out of the instrument, generating physical perturbations. To prove this, we intentionally shook the crystallized solution, which instantly generated massive ~10000 nm scattering signals (Fig. R2e).

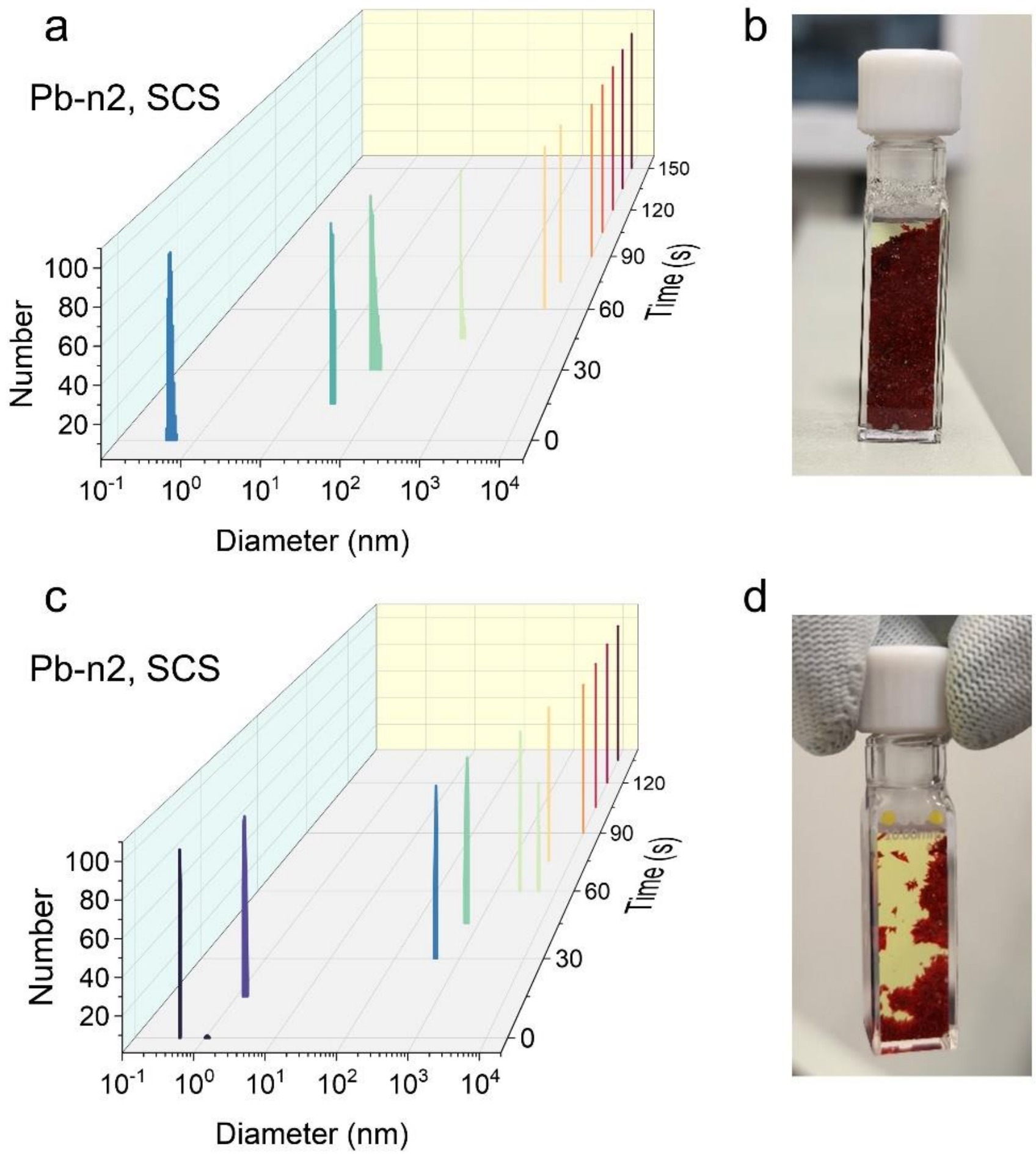


**Fig. R1. In-situ DLS tracking of the Pb-n2, SCS system.** In situ DLS distribution of the Pb-n2, SCS precursor solution during natural cooling. Due to the rapid crystallization kinetics of the SCS method, measurements were initiated immediately upon transferring the hot, clear solution into the instrument (duration: 15 s; interval: 0 s). **a, c.** Two independent continuous in situ tracking profiles showing the rapid emergence of macroscopic scattering signals. **b, d.** Images of the corresponding solution immediately after the DLS measurements, revealing massive and rapid crystallization.

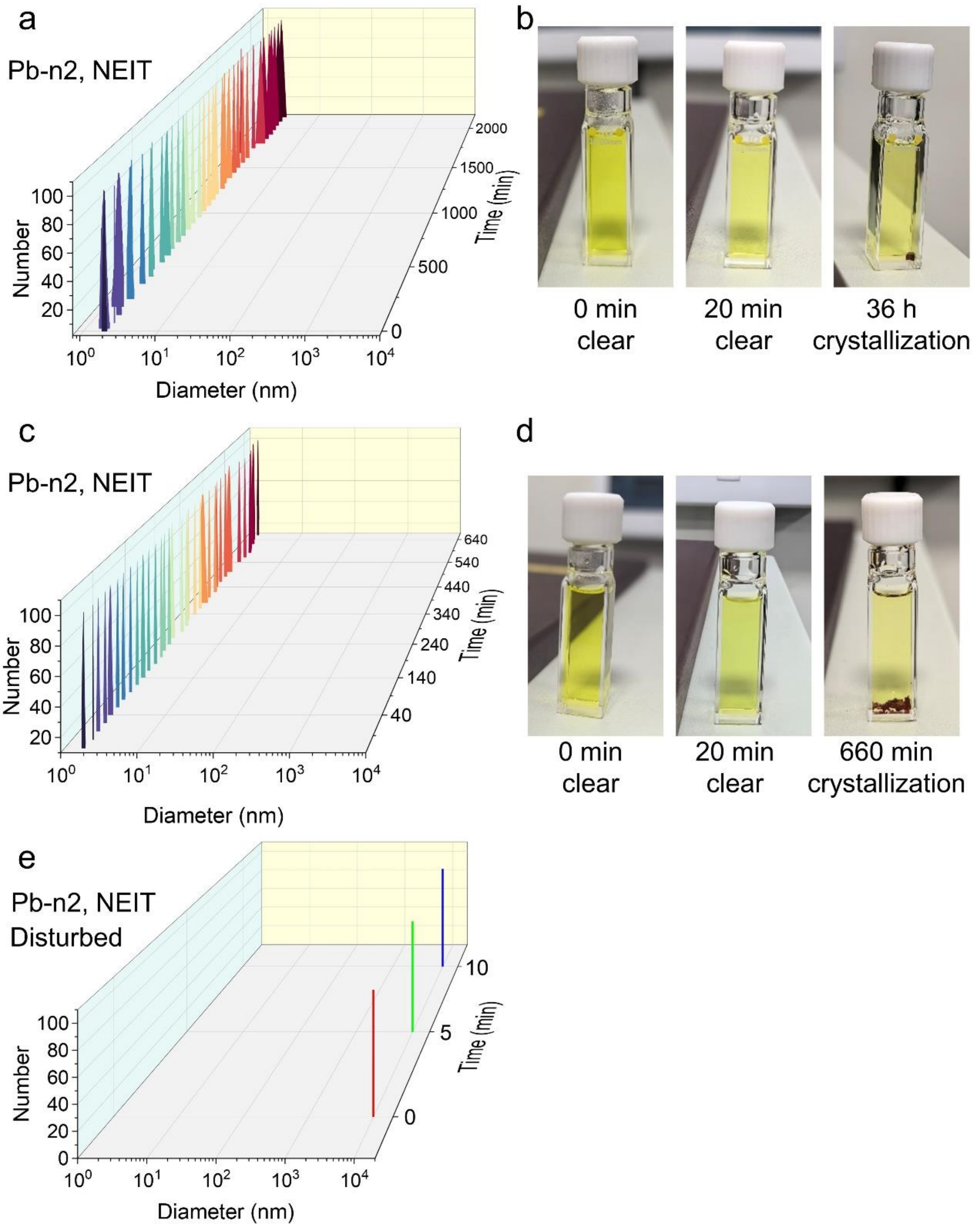


**Fig. R2. In-situ DLS tracking of the Pb-n2, NEIT system. a.** Continuous tracking during the initial cooling (first 20 min) and subsequent RT (measured hourly). The profile maintains peak at ~1-2 nm throughout the entire 36 h process until crystallization. **b.** Images corresponding to (a) at 0 min (hot clear solution), 20 min (cooled to RT, clear), and 36 h (crystallization). **c.** DLS tracking of another sample to monitor accelerated growth. After maintaining a ~1-2 nm peak during the initial 20 min cooling, the solution was intentionally shaken to accelerate nucleation and measured every 20 min. The signal persistently remained at ~1-2 nm up to 660 min. **d.** Images corresponding to (c) at 0 min (hot clear solution), 20 min (cooled to RT, clear), and 660 min (crystallization). **e.** DLS profile of the crystallized solution from (c) measured immediately after intentional mechanical

disturbance (shaking), which instantly triggered the appearance of macroscopic scattering signals (~10000 nm). Note: Unlike previous quasi-in situ measurements where sample transfer caused mechanical disturbance, the steady-growth tracking in a and c was strictly in situ and undisturbed.

***Revision:*** Figs. R1 and R2 are incorporated in the revised Supplementary Information as Supplementary Figs. 3 and 7.

Additionally, SI Fig 22 "ump fluence" should be "pump fluence"

***Response:*** We apologize for this typographical error and sincerely thank you for careful reading. We have corrected "ump fluence" to "pump fluence" in the revised Supplementary Fig. 28.

Reviewer #2 (Remarks to the Author):

The authors have done a considerable effort to improve the manuscript to be published in the journal. I have three questions prior to recommend for publication:
(1) The authors showed angular-resolved PL is uniform for the 2D perovskite single crystal. I appreciate the experimental validation in Fig. S16, but could the author comment on why the strong structure anisotropy did not lead to optical anisotropy? It has been previously demonstrated both theoretically and experimentally that the emission intensity is angular dependent (stronger in the out-of-plane direction) in 2D perovskites (https://doi.org/10.1021/acs.jpclett.0c00901, https://doi.org/10.1016/j.jmat.2023.01.014). This discrepancy may affect an over-estimated PLQY value.

***Response:*** We sincerely thank you for this highly constructive and insightful comment. We completely agree that the intrinsic structural anisotropy of 2D layered perovskites can lead to an orientation-dependent emission profile (i.e., discrepancies between the in-plane and out-of-plane emission characteristics). Therefore, simply assuming optical isotropy in all directions would indeed lead to an overestimation of the overall photoluminescence quantum yield (PLQY). To avoid this overestimation caused by ignoring the PL anisotropy, it is necessary to collect both the in-plane and out-of-plane PL emissions across a full 0° to 90° range for accurate PLQY correction. However, our angle-dependent PL system utilizes an objective lens with a numerical aperture (NA) of 0.9, which dictates a theoretical collection limit of ~64° and only provides reliable data within the 0° to 62° range. To overcome this limitation and capture the complementary high-angle (62° to 90°) PL emission, a circularly polarized luminescence spectrometer equipped with a molecular orientation unit was employed to measure the angle-dependent PL emission within the 62° to 90° range. The schematic of this combined low-angle (0° to 62°) and high-angle (62° to 90°) angle-dependent PL measurement is illustrated in Fig. R3.

**1) Verification of isotropic low-angle (0° to 62°) emission:**

To ensure reliability, we randomly selected eight distinct areas on the Pb-n1, Pb-n2, and Pb-n3 single crystals and conducted angle-dependent PL measurements (Figs. R4-R6). Within the objective's reliable measurable range from -62° to +62°, the PL emission intensity remained uniform across all sampled areas. This direct experimental evidence confirms that for these PEA-based 2D perovskite single crystals, the low-angle (0° to 62°) PL emission is isotropic. The sharp decrease in PL intensity observed near 0° is an instrumental artifact originating from the optical measurement setup.

**2) Characterization of anisotropic high-angle (62° to 90°) PL emission:**

As the reviewer pointed out, for 2D layered perovskites, the in-plane emission profile is distinct from the out-of-plane distribution. To investigate the in-plane emission characteristics, the single crystals were rapidly dissolved and immediately spin-coated into thin films. Although these thin films present certain differences compared to the single crystals, they serve as a suitable platform for angle-dependent PL analysis (*J. Phys. Chem. Lett.* 2020, 11, 3458–3465; *J. Materiomics.* 2023, 9, 762-767; *Adv. Mater.* 2021, 33, 2007176). Utilizing the aforementioned molecular orientation spectrometer, the emission behavior at high angles (62° to 90°) was extracted (Fig. R7).

By integrating these two sets of angle-dependent PL profiles (low-angle and high-angle) over the full angular range, an anisotropy correction factor was derived (0.804 for Pb-n1, 0.827 for Pb-n2, and 0.780 for Pb-n3). By multiplying the initial values by this factor, the PLQY values for all 2D perovskite single crystals were recalibrated (Fig. R8), thereby correcting the previous overestimation. Furthermore, the primary objective of our comparative PLQY measurements is to demonstrate the enhanced crystal quality of the NEIT-grown single crystals. While instrumental limits may introduce minor systemic deviations in absolute PLQY values, all samples (including both NEIT-grown crystals and SCS-grown controls) were evaluated under identical conditions. This ensures a constant systemic deviation, preserving the strict comparability of our dataset. Consequently, our core conclusion remains unchanged: the NEIT strategy effectively improves single crystal quality.

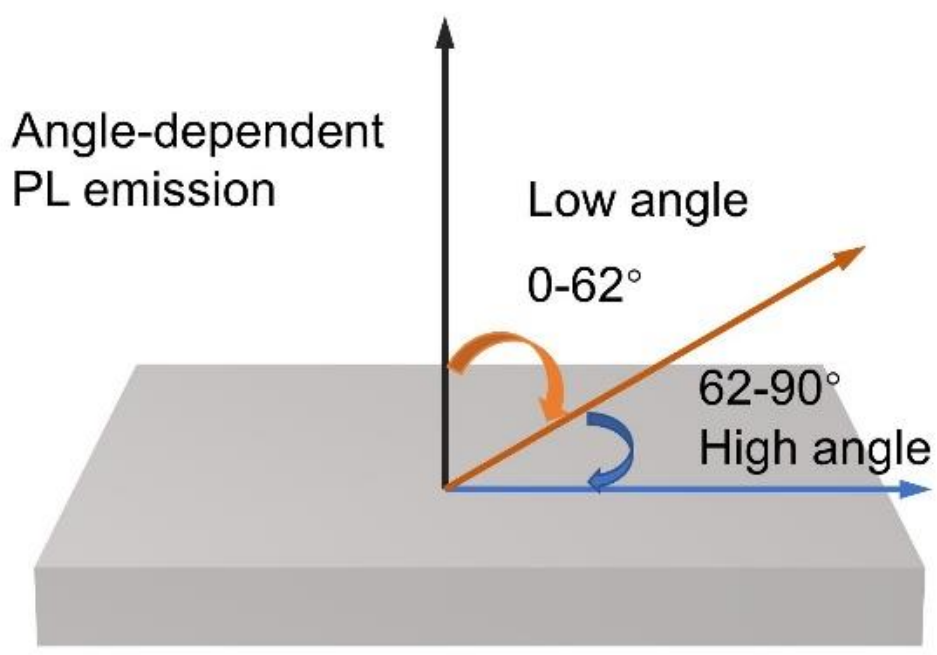


**Fig. R3. Schematic illustration of the angle-dependent PL measurements. The angles are defined relative to the surface normal, comprising a low-angle region (0° to 62°) and a complementary high-angle region (62° to 90°).**

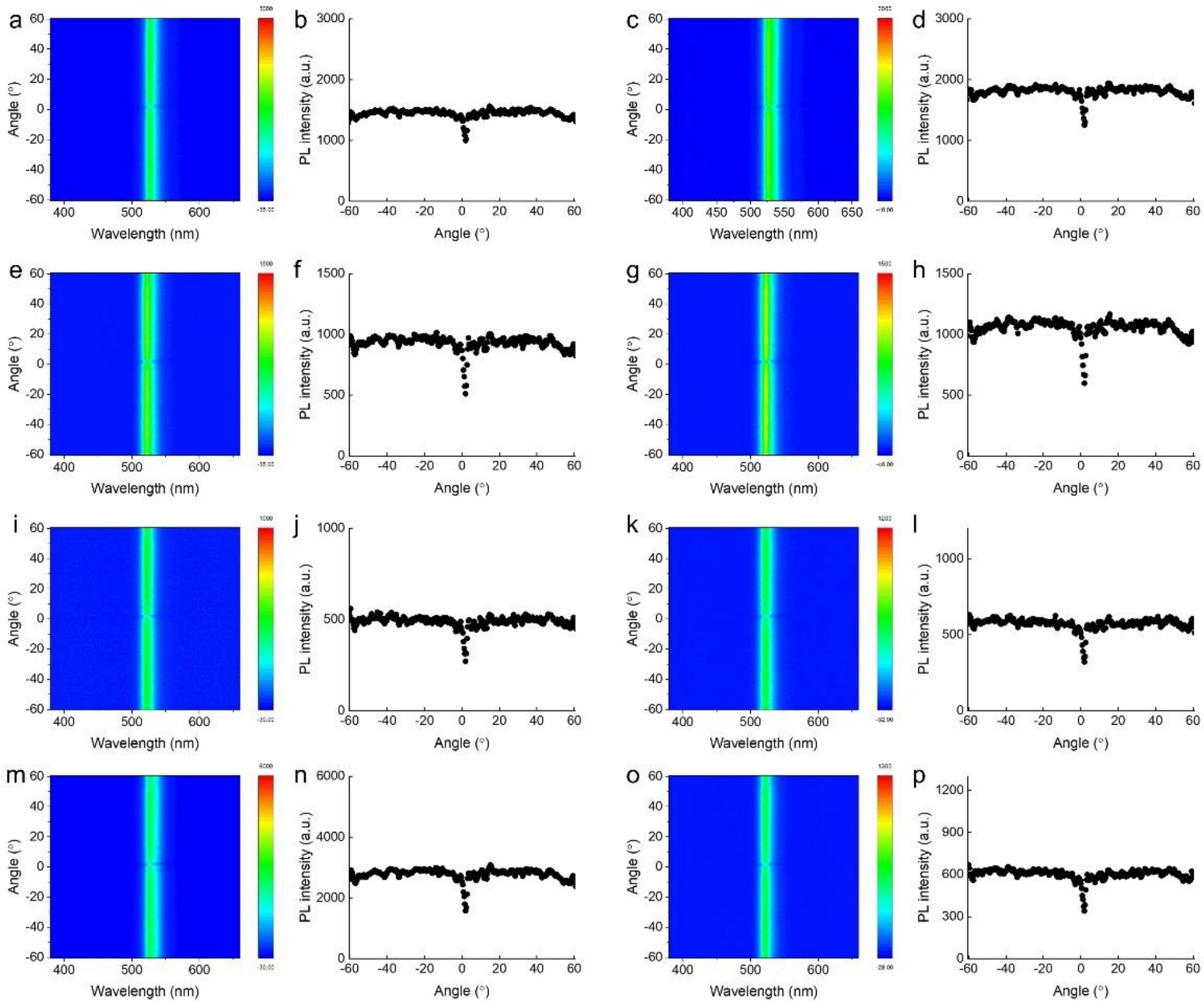

**Fig. R4. Low-angle angle-dependent PL of Pb-n1 single crystals. a, c, e, g, i, k, m, o,** 2D pseudo-color maps of micro-area angle-resolved PL spectra collected from eight randomly selected regions on the Pb-n1 single crystal. **b, d, f, h, j, l, n, p,** Corresponding extracted peak PL intensities recorded at different emission angles. The sharp decrease in PL intensity observed near 0° is an instrumental artifact originating from the optical measurement setup.

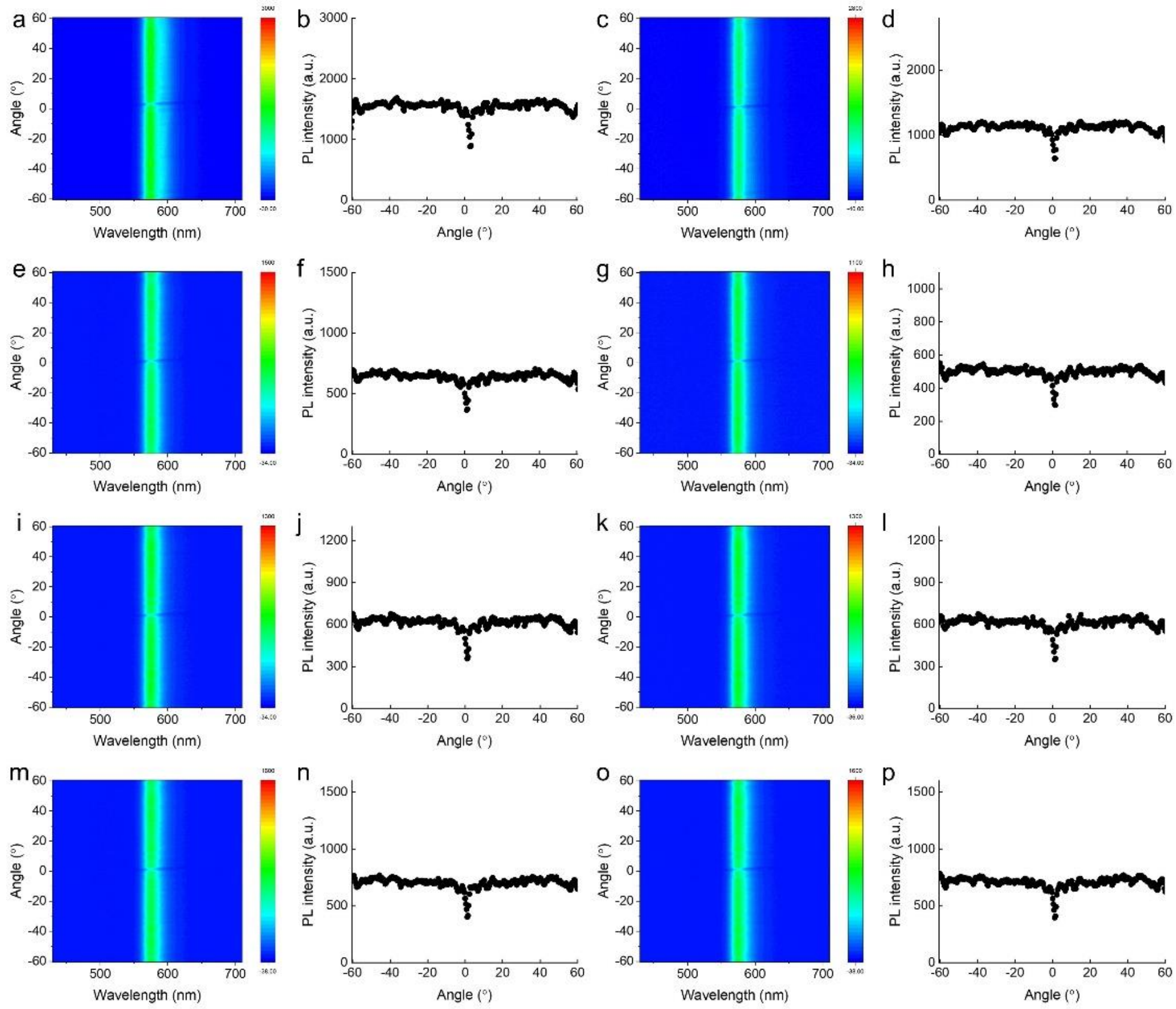


**Fig. R5. Low-angle angle-dependent PL of Pb-n2 single crystals. a, c, e, g, i, k, m, o,** 2D pseudo-color maps of micro-area angle-resolved PL spectra collected from eight randomly selected regions on the Pb-n2 single crystal. **b, d, f, h, j, l, n, p,** Corresponding extracted peak PL intensities recorded at different emission angles. The sharp decrease in PL intensity observed near 0° is an instrumental artifact originating from the optical measurement setup.

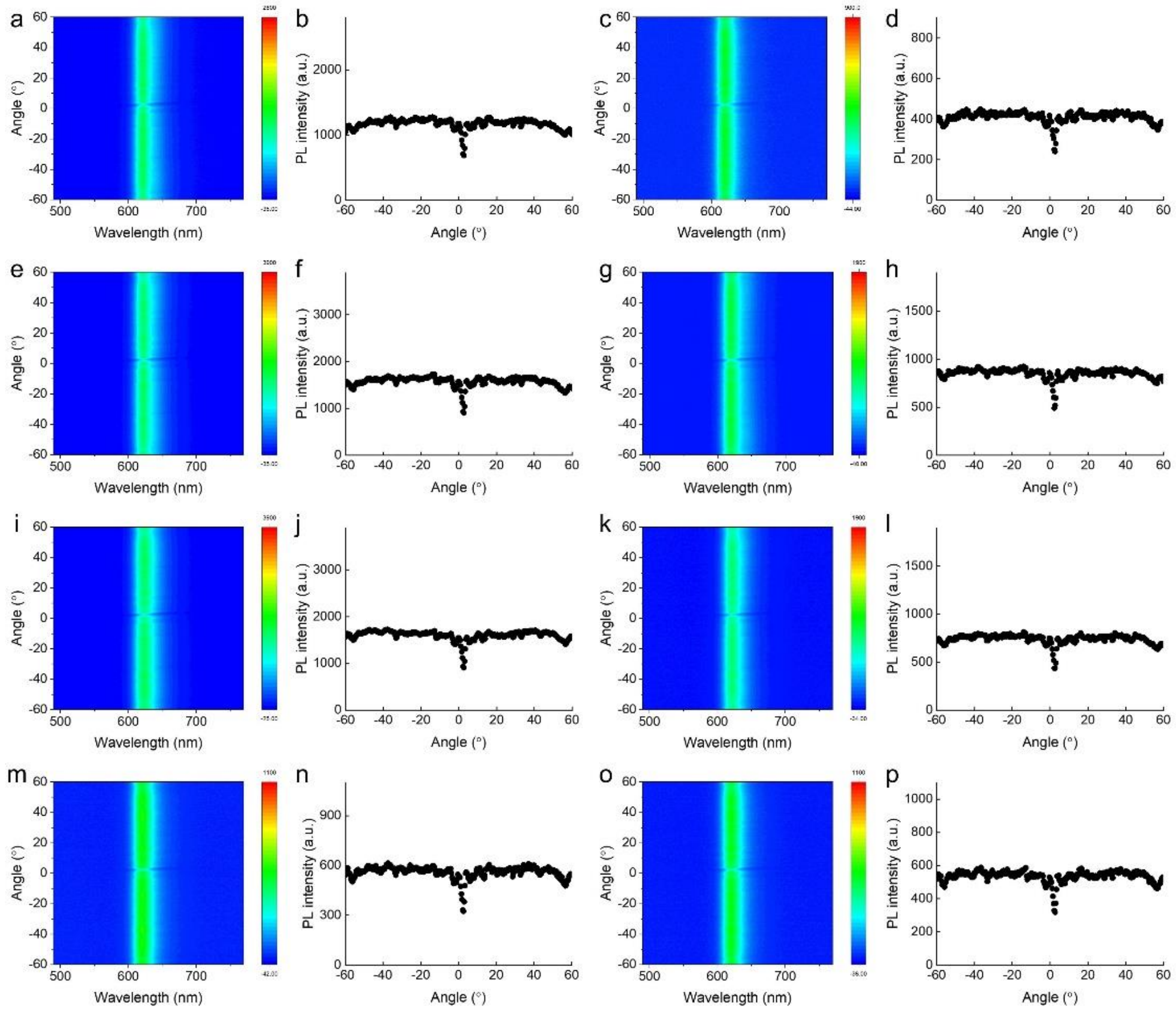

**Fig. R6. Low-angle angle-dependent PL of Pb-n3 single crystals. a, c, e, g, i, k, m, o,** 2D pseudo-color maps of micro-area angle-resolved PL spectra collected from eight randomly selected regions on the Pb-n3 single crystal. **b, d, f, h, j, l, n, p,** Corresponding extracted peak PL intensities recorded at different emission angles. The sharp decrease in PL intensity observed near 0° is an instrumental artifact originating from the optical measurement setup.

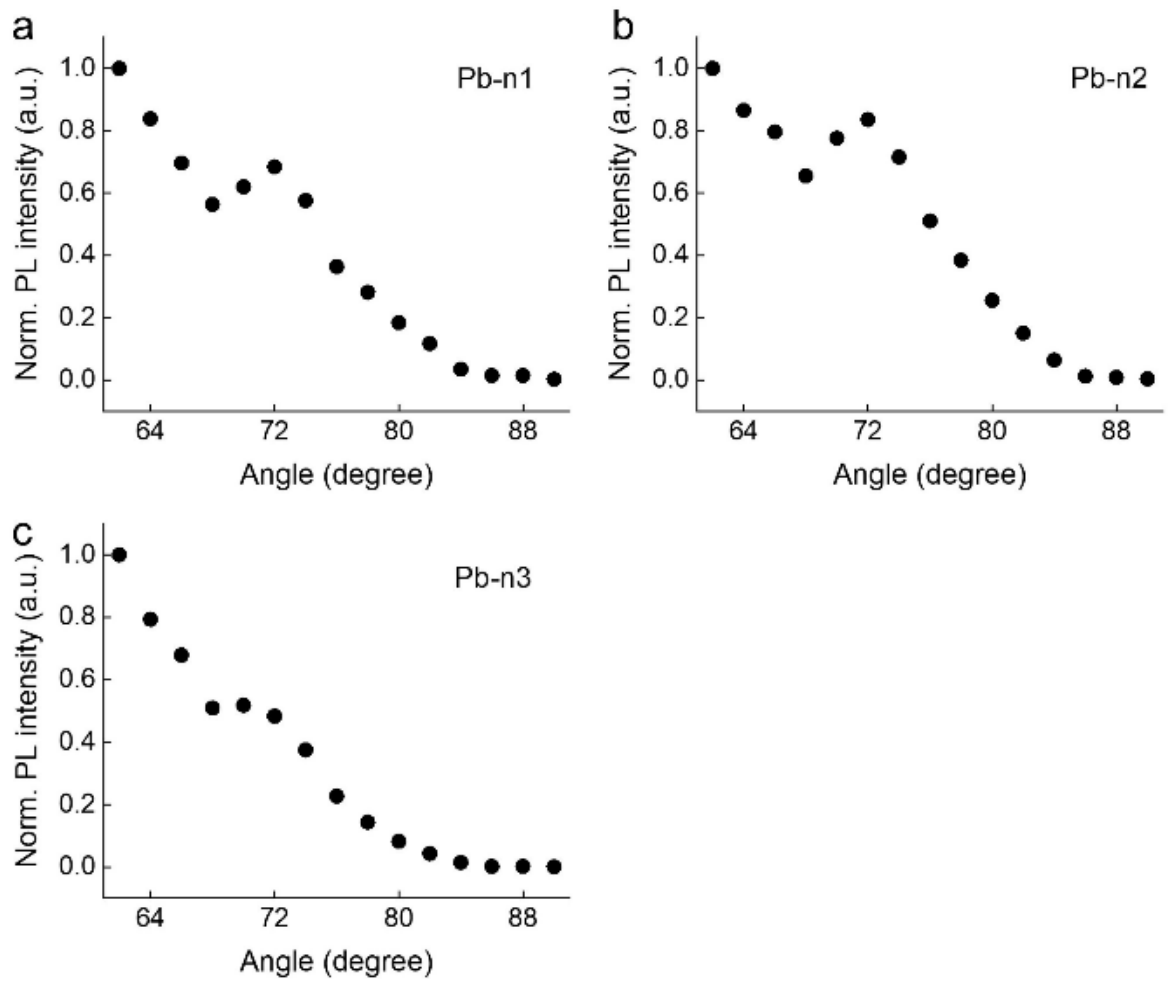


**Fig. R7. High-angle angle-dependent PL profiles of 2D perovskites.** Normalized peak PL intensities recorded at high emission angles (ranging from 62° to 90°) for Pb-n1 (a), Pb-n2 (b), and Pb-n3 (c). Data were collected using a molecular orientation spectrometer on spin-coated thin films, which were prepared by dissolving the respective single crystals. In contrast to the out-of-plane isotropic emission, the pronounced attenuation of PL intensity towards 90° clearly demonstrates the in-plane optical anisotropy dictated by the 2D layered structure. These high-angle emission profiles were utilized to comprehensively recalibrate the PLQY values.

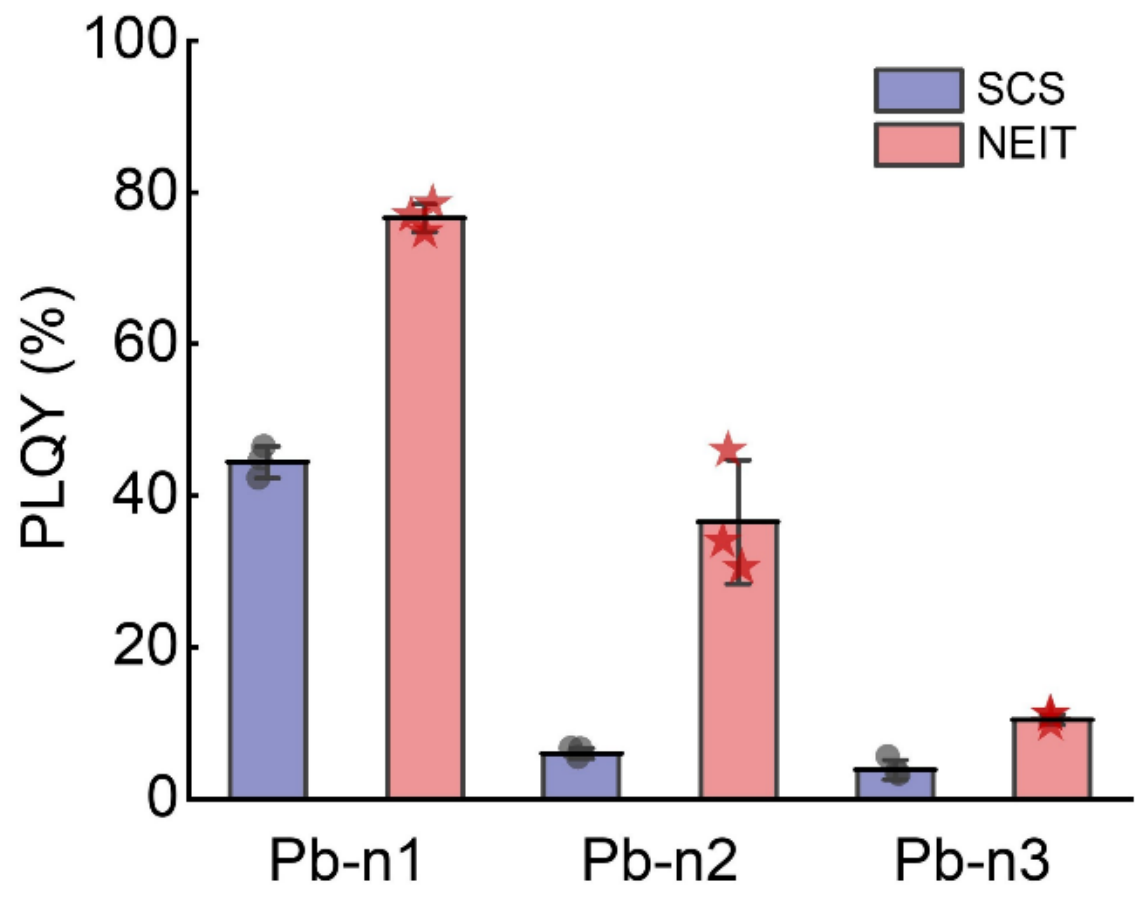


**Fig. R8. PLQYs of Pb-n1, n2 and n3 single crystals synthesized from NEIT and SCS methods.**

***Revision:*** Figs. R3-R7 are incorporated in the revised Supplementary Information as Supplementary Figs. 18-22. Fig. R8 is incorporated in the revised Fig. 1g.

The following texts (highlighted in yellow) are included in Line 197 of the revised main text: NEIT-grown crystals exhibit much brighter PL emissions compared to SCS grown crystals at identical emission peaks (~530 nm for Pb-n1, ~580 nm for Pb-n2, ~620 nm for Pb-n3; Fig. 1f), culminating in a high PLQY of 77 ± 2% for NEIT-grown Pb-n1 crystal—surpassing SCS counterpart (44 ± 2%) and establishing a new benchmark for all reported Ruddlesden-Popper perovskite single crystals [20,24,42-44]. Similarly, PLQYs for Pb-n2 and Pb-n3 reach 37± 8% and 10 ± 1%, representing 6.3-fold and 2.6-fold enhancement over conventional SCS methods (6 ± 1% and 4 ± 1% for SCS-grown Pb-

n2 and Pb-n3 crystals, Fig. 1g and Supplementary Figs. 15, 16).

(Line 204) Furthermore, both polarization-dependent and low-angle (0-62°) angle-dependent PL spectroscopy confirmed the isotropic out-of-plane emission profile of these 2D perovskite flakes. However, high-angle (62-90°) angle-dependent PL revealed that the in-plane emission is weaker than the out-of-plane emission. By combining the PL profiles from both angular ranges, the final PLQY values were recalibrated (Supplementary Figs. 17-25).

(Line 688) Angle-dependent PL measurements were performed using two complementary systems to cover the full emission profile. The low-angle emission (0 to ±62°) was collected on a home-built system consisting of a 100× objective (NA = 0.9), a Princeton Instruments spectrometer, and a PIXIS-1024B detector. The high-angle emission (62° to 90°) was acquired using a circularly polarized luminescence spectrometer equipped with a molecular orientation unit (JASCO CPL-300).

We have incorporated the following details into the PLQY testing method of Supplementary Information (1. Supplementary Characterizations, Page 3): Considering the intrinsic optical anisotropy of 2D layered perovskites, the initial PLQY values were recalibrated. Isotropic low-angle emission within the objective's collection limit (-62° to +62°) was confirmed using a home-built angle-dependent PL system (100× objective, NA = 0.9, Princeton Instruments spectrometer, PIXIS-1024B detector). The high-angle (62° to 90°) emission profile was extracted on thin films fabricated by immediately spin-coating the dissolved single crystals (n=1~3) using a JASCO CPL-300 spectrometer equipped with a molecular orientation unit[2-4]. Integrating these two sets of emission profiles (low-angle and high-angle) over the full angular range yielded specific anisotropy correction factors (0.804 for Pb-n1, 0.827 for Pb-n2, and 0.780 for Pb-n3). By multiplying the initial values by these factors, the PLQY values for all 2D perovskite single crystals were recalibrated, thereby correcting the overestimation induced by structural confinement.

2 Walters, G. *et al.* Directional light emission from layered metal halide perovskite crystals. *J. Phys. Chem. Lett.* **11**, 3458-3465 (2020).

3 Qi, Y. *et al.* Anisotropic emission of orientation-controlled mixed-dimensional perovskites for light-emitting devices. *J. Materiomics.* **9**, 762-767 (2023).

4 Sidhik, S. *et al.* Memory seeds enable high structural phase purity in 2D perovskite films for high-efficiency devices. *Adv. Mater.* **33**, 2007176 (2021).

(2) The authors stated a mismatch value of 1.6% between lateral Pb-Sn epitaxial heterostructure. The claim of that mismatch value 'is accommodated without cracking or significant defects' is not substantiated with existing data. It is hard to validate whether small cracks form that are not yet being captured in the current resolution, and characterization of defects is not provided. Moreover, it is less likely that the 1.6% mismatch did not show any impact at all. In the 5% lattice mismatch report the author quoted, the strain can be seen relaxed through ripples and dislocations.

***Response:*** We appreciate your insightful comment and agree with your assessment.

While the soft ionic lattice and flexible organic spacers of 2D perovskites elastically accommodate minimal mismatch to prevent macroscopic cracking, the residual strain at the micro/nanoscale is likely released through dislocations or other structural defects.

Furthermore, we would like to clarify that the ~1.6% lattice mismatch mentioned in the manuscript is specifically calculated for the Pb, n2-n1 vdW heterostructure. For the lateral Pb-Sn, n2 lateral heterostructure, the mismatch was initially stated as < 2%. Furthermore, the newly supplemented RSM data, acquired following your suggestion, demonstrate that the lattice mismatch of the Pb-Sn, n2 lateral heterostructure is ~ 0.6% (< 1%).

***Revision:*** The following texts (highlighted in yellow) are included in Line 400 of the revised main text: Benefiting from the soft ionic lattice and flexible organic spacers of 2D halide perovskites, the < 1% lattice mismatch is accommodated without observable macroscopic cracking, while the localized stress is likely released through the formation of interfacial dislocations [6].

(3) While the mismatch value of 1.6% is quoted, in the (110) plane XRD data (Fig. S42) the two peaks should be expected to have q value difference of 1.6%. As the q value is ~1 A-1, the difference would be 0.01-0.02 A-1. The data in Fig.S42a shows less than 0.004 A-1 and even less than 0.0005 A-1 q value difference in Fig. S42c. It is therefore unclear whether the second signal belongs to Sn-n2, which shed question on whether the epitaxial relationship is proven. Would the author provide reference pure Sn-n2 samples?

***Response:*** We appreciate your insightful comment. The misunderstanding of the q value in the (110) plane RSM originated from our previous data processing method.

To clarify this, we conducted high-resolution 1D XRD (for the (00k) and (110) planes) and 2D RSM measurements (for the (110) plane) on the Pb-n2, Sn-n2 single crystals, and Pb-Sn, n2, lateral heterostructure. Our measurement began with 2theta-omega scans along the (00k) direction (Fig. R9a, R10a, R11a). For the Pb-Sn, n2, lateral heterostructure, reflections from the (001) to (003) planes exhibit overlapping signals. The (004) plane is the first crystallographic plane where the distinct peaks of the two constituent phases can be clearly resolved. Based on the single-crystal structure, the interplanar angle (psi) between the (004) and (110) plane normals was calculated to be approximately 82°. The instrument was subsequently rotated to the target (110) plane. Because the conventional scanning mode lies in forbidden zone, a skew scan geometry was employed.
Under this skew mode, the $q_x$ and $q_z$ axes converted by the LEPTOS software are tilted. Previously, to improve the visual presentation, we applied a custom model to orthogonalize the $q_x$-$q_z$ coordinates. However, this process inadvertently led to confusion. To eliminate ambiguity, we now present the raw $q_x$ and $q_z$ RSM data directly exported from LEPTOS (Fig. R12). The horizontal coordinates of the heterostructure

align consistently with those of the corresponding individual single crystals, confirming epitaxial growth.

Based on these raw RSM results, we now further correct the lattice mismatch value reported in the manuscript. Prior to these detailed measurements, we calculated the theoretical lattice mismatch based on potential epitaxial alignments. Since Sn-n2 could theoretically grow on either the Pb-n2 (110) or ($1\bar{1}0$) planes, there are four potential matching configurations:(1) Pb-n2 (110) // Sn-n2 (110) with a lattice mismatch of 0.6%,(2) Pb-n2 (110) // Sn-n2 ($1\bar{1}0$) with a lattice mismatch of 0.6%,(3) Pb-n2 ($1\bar{1}0$) // Sn-n2 (110) with a lattice mismatch of 1.8%, (4) Pb-n2 ($1\bar{1}0$) // Sn-n2 ($1\bar{1}0$) with a lattice mismatch of 0.6% (as detailed in the Tables R1 and R2). This is why our original manuscript quoted a lattice mismatch of less than 2%. However, the RSM data demonstrates that the actual epitaxial alignment is Pb-n2 (110) // Sn-n2 (110) (Figs. R11 and R12, Table R1). This specific alignment possesses an exceptionally small intrinsic lattice mismatch of only 0.6%. Accordingly, we have implemented corresponding revisions in the main text, updating the lattice mismatch of the Pb-Sn, n2 lateral heterostructure to <1%.

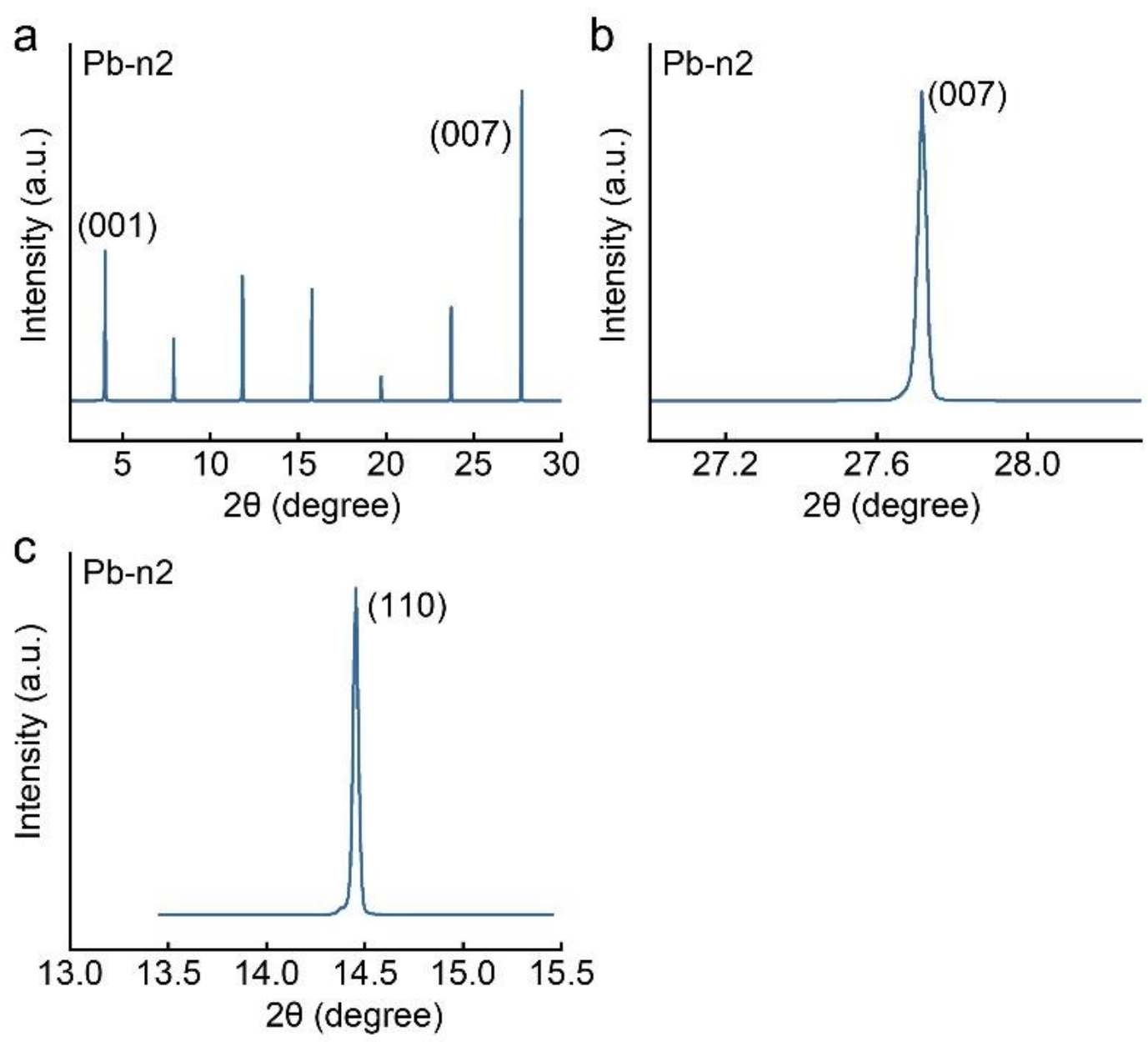


**Fig. R9. High-resolution 1D XRD pattern of Pb-n2 single crystal. a.** High-resolution 1D XRD pattern of Pb-n2 single crystals in (00k) plane. **b.** The magnified region of (a) around the (007) diffraction peak. **c.** High-resolution 1D XRD pattern of Pb-n2 single crystals in (110) plane.

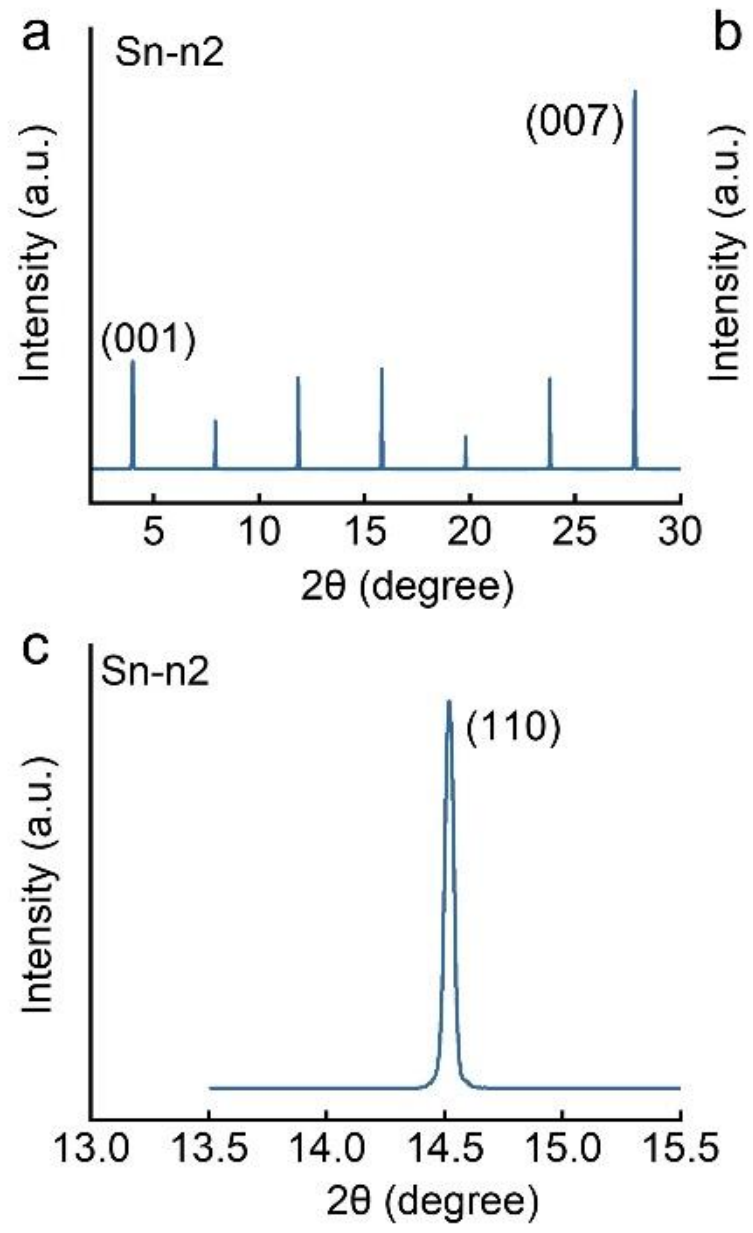


**Fig. R10. High-resolution 1D XRD pattern of Sn-n2 single crystal. a.** High-resolution 1D XRD pattern of Sn-n2 single crystals in (00k) plane. **b.** The magnified region of (a) around the (007) diffraction peak. **c.** High-resolution 1D XRD pattern of Sn-n2 single crystals in (110) plane.

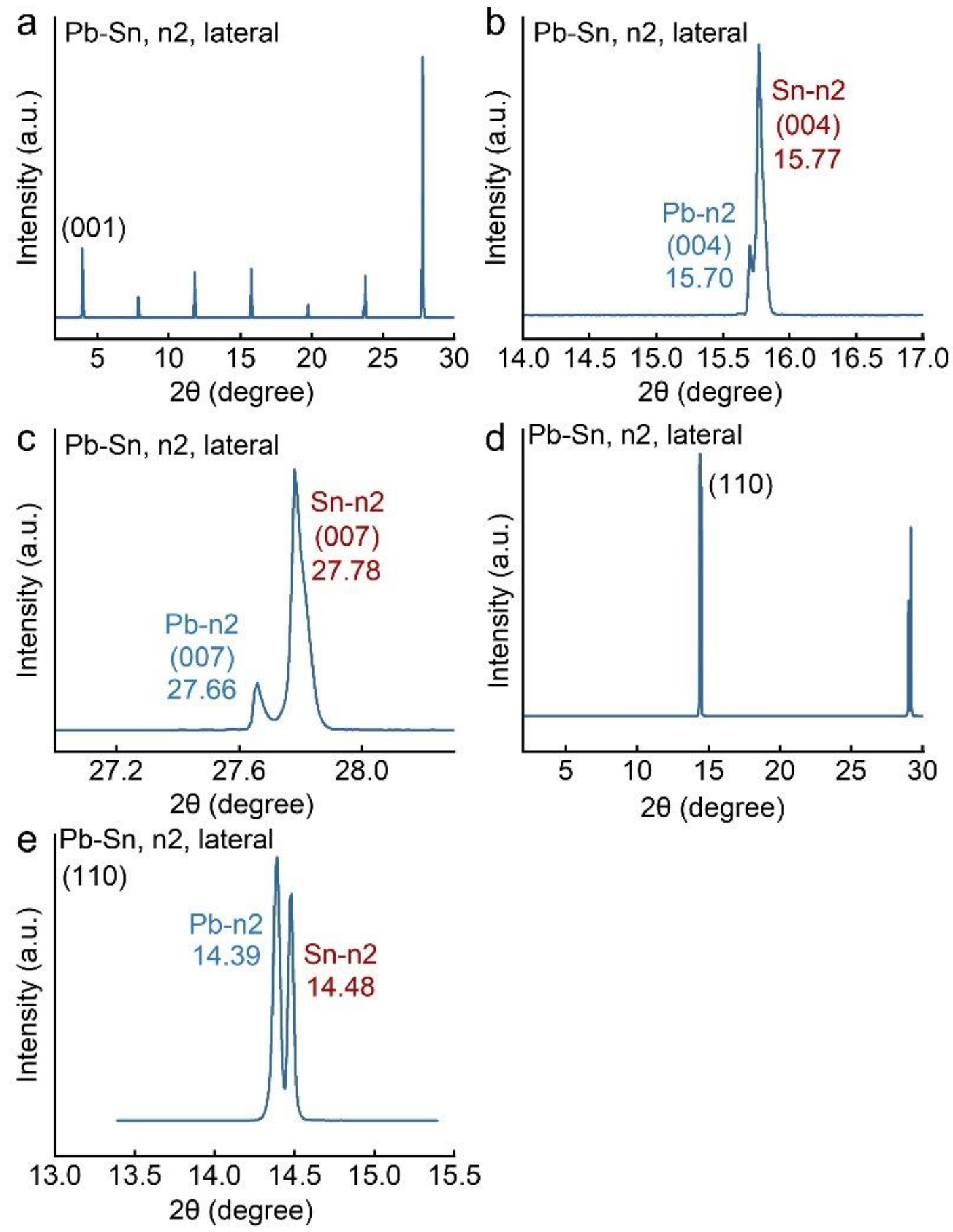

**Fig. R11. High-resolution 1D XRD pattern of Pb-Sn, n2 lateral heterostructure. a.** High-resolution 1D XRD pattern of Pb-Sn, n2 lateral heterostructure in (00k) plane. **b, c.** The magnified region of (a) around the (004) and (007) diffraction peaks reveal 2 distinct peaks corresponding to Pb-n2 and Sn-n2. **d.** High-resolution 1D XRD pattern of Pb-n2 single crystals in (110) plane. **e.** The fine scan of (110) plane reveal 2 distinct peaks corresponding to Pb-n2 and Sn-n2.

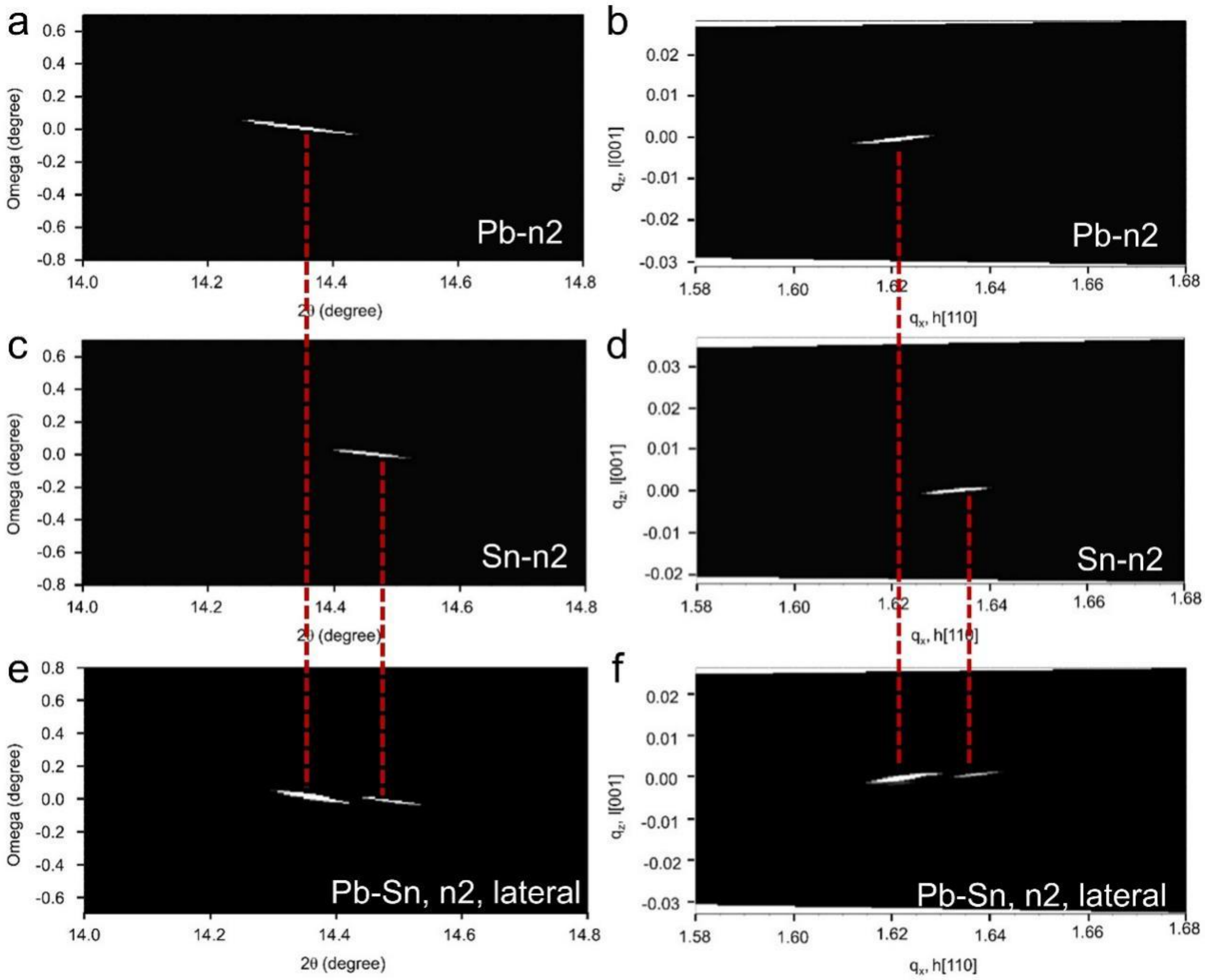


**Fig. R12. Crystallographic evidence for the in-plane orientational epitaxy of Pb-Sn, n2 lateral heterostructure.** Raw RSM data of the (110) planes for pure Pb-n2 single crystal (**a**), pure Sn-n2 single crystal (**c**), and the Pb-Sn n2 lateral heterostructure (**e**). RSM of the (110) planes converted by the LEPTOS software for the pure Pb-n2 (**b**), pure Sn-n2 (**d**), and the heterostructure (**f**) samples. Both the raw data and RSM maps reveal that the diffraction peaks of the pure Pb-n2 and Sn-n2 single crystals align with those observed in the heterostructure.

Table R1. Theoretical crystallographic parameters (d-spacing and 2θ) for the (110) and ($1\bar{1}0$) planes of Pb-n2 and Sn-n2 single-crystal.

| Crystal | Plane | d-spacing (Å) | 2θ (°) |
|---|---|---|---|
| Pb-n2 | (110) | 6.15595 | 14.3766 |
| Pb-n2 | ($1\bar{1}0$) | 6.23147 | 14.2014 |
| Sn-n2 | (110) | 6.11915 | 14.4635 |
| Sn-n2 | ($1\bar{1}0$) | 6.19421 | 14.3221 |

Table R2. Calculated lattice mismatch for various potential epitaxial alignments between the in-plane facets of Pb-n2 and Sn-n2.

| Pb-n2 Plane | d-spacing (Å) | Sn-n2 Plane | d-spacing (Å) | Lattice Mismatch |
|---|---|---|---|---|
| Pb-n2 (110) | 6.15595 | Sn-n2 (110) | 6.11915 | 0.6% |
| Pb-n2 (110) | 6.15595 | Sn-n2 ($1\bar{1}0$) | 6.19421 | 0.6% |
| Pb-n2 ($1\bar{1}0$) | 6.23147 | Sn-n2 (110) | 6.11915 | 1.8% |
| Pb-n2 ($1\bar{1}0$) | 6.23147 | Sn-n2 ($1\bar{1}0$) | 6.19421 | 0.6% |

***Revision:*** Figs. R9-R12 are incorporated in the revised Supplementary Information as Supplementary Figs. 48-51.

The following texts (highlighted in yellow) are modified in Line 399 of the revised main text: Seeded epitaxy in $PEA_2MASn_2I_7$ (Sn-n2) solution generates both vdW-stacked and lateral heterostructures of $PEA_2MAPb_2I_7$-$PEA_2MASn_2I_7$ (Pb-Sn, n2), enabled by minimal lattice mismatch (< 1%) along the (110) crystallographic orientation (Fig. 4a, 4c-4d and Supplementary Figs. 46, 47).

(Line 431) Furthermore, HRXRD 1D scan and 2D reciprocal space mapping (RSM) of the (110) plane reveal that the diffraction peaks of the pure Pb-n2 and Sn-n2 single crystals align with those observed in the heterostructure, verifying the aligned in-plane orientation of the Pb-Sn, n2 lateral heterostructure (Fig. 4g and Supplementary Figs. 48-51).
